# Global impact of the first year of COVID-19 vaccination: a mathematical modelling study

**DOI:** 10.1016/S1473-3099(22)00320-6

**Published:** 2022-09

**Authors:** Oliver J Watson, Gregory Barnsley, Jaspreet Toor, Alexandra B Hogan, Peter Winskill, Azra C Ghani

**Affiliations:** a MRC Centre for Global Infectious Disease Analysis, Imperial College London, London, UK

## Abstract

**Background:**

The first COVID-19 vaccine outside a clinical trial setting was administered on Dec 8, 2020. To ensure global vaccine equity, vaccine targets were set by the COVID-19 Vaccines Global Access (COVAX) Facility and WHO. However, due to vaccine shortfalls, these targets were not achieved by the end of 2021. We aimed to quantify the global impact of the first year of COVID-19 vaccination programmes.

**Methods:**

A mathematical model of COVID-19 transmission and vaccination was separately fit to reported COVID-19 mortality and all-cause excess mortality in 185 countries and territories. The impact of COVID-19 vaccination programmes was determined by estimating the additional lives lost if no vaccines had been distributed. We also estimated the additional deaths that would have been averted had the vaccination coverage targets of 20% set by COVAX and 40% set by WHO been achieved by the end of 2021.

**Findings:**

Based on official reported COVID-19 deaths, we estimated that vaccinations prevented 14·4 million (95% credible interval [Crl] 13·7–15·9) deaths from COVID-19 in 185 countries and territories between Dec 8, 2020, and Dec 8, 2021. This estimate rose to 19·8 million (95% Crl 19·1–20·4) deaths from COVID-19 averted when we used excess deaths as an estimate of the true extent of the pandemic, representing a global reduction of 63% in total deaths (19·8 million of 31·4 million) during the first year of COVID-19 vaccination. In COVAX Advance Market Commitment countries, we estimated that 41% of excess mortality (7·4 million [95% Crl 6·8–7·7] of 17·9 million deaths) was averted. In low-income countries, we estimated that an additional 45% (95% CrI 42–49) of deaths could have been averted had the 20% vaccination coverage target set by COVAX been met by each country, and that an additional 111% (105–118) of deaths could have been averted had the 40% target set by WHO been met by each country by the end of 2021.

**Interpretation:**

COVID-19 vaccination has substantially altered the course of the pandemic, saving tens of millions of lives globally. However, inadequate access to vaccines in low-income countries has limited the impact in these settings, reinforcing the need for global vaccine equity and coverage.

**Funding:**

Schmidt Science Fellowship in partnership with the Rhodes Trust; WHO; UK Medical Research Council; Gavi, the Vaccine Alliance; Bill & Melinda Gates Foundation; National Institute for Health Research; and Community Jameel.

## Introduction

The first COVID-19 vaccine was delivered outside of a clinical trial setting on Dec 8, 2020.^1^ By Dec 8, 2021, 55·9% of the global population was estimated to have received at least one dose of a COVID-19 vaccine, 45·5% estimated to have received two doses, and 4·3% estimated to have received a booster dose.^2^ Despite the incredible speed with which COVID-19 vaccines were developed in 2020 and subsequently distributed during 2021, more than 3·5 million deaths due to COVID-19 have been reported globally since the first vaccine was administered.^2^

Understanding the global impact of vaccination on the course of the COVID-19 pandemic is challenging given the heterogeneous access to vaccines coupled with different levels of transmission and ongoing non-pharmaceutical interventions across countries. In the early months of 2021, the impact of vaccination would have been minimal because of the delay in developing the infrastructure for a widespread vaccination campaign, the need for a delayed two-dose regimen in some jurisdictions to ensure maximum protection,^3^ and the delay in the development of antibodies following vaccination. Additionally, as vaccine supply was constrained, most countries opted to prioritise vaccination in high-risk populations, including health-care workers and older people. Such strategies would have generated direct protection but would have had comparatively less impact on SARS-CoV-2 transmission. However, from mid-2021 onwards those countries with access to plentiful vaccine supply opted for mass vaccination of the adult population, later including children and subsequent boosting to maintain high levels of protection given the waning in vaccine efficacy and the emergence of new variants of concern. This approach has resulted in vast inequalities in global vaccine distribution.^4^

**Research in context**

**Evidence before this study**

We searched PubMed up to April 26, 2022, without any date limits or language restrictions, using the search terms “vaccin* AND impact AND (death* OR live*) AND (estimat* OR evaluat*) AND (COVID-19 OR SARS-CoV-2)”. We found eight published studies that estimated the impact of COVID-19 vaccination, including deaths averted from vaccination. None of the studies considered the global impact of COVID-19 vaccination, focusing instead on specific regions (Italy, California, North Carolina, Stockholm, subsets of states in the USA, New York City, and the WHO European Region). Furthermore, the study focusing on the WHO European region only quantified the direct impact of vaccination and did not estimate the indirect effects (ie, decreasing infection risk of both vaccinated and unvaccinated susceptible individuals).

**Added value of this study**

This mathematical modelling study advances previous work both in terms of scale (number of regions modelled) and in terms of quantifying both the direct and indirect impact of COVID-19 vaccination globally. We estimated the impact of vaccination up to Dec 8, 2021, by fitting COVID-19 transmission models to both reported deaths and excess mortality during the pandemic as a proxy for deaths due to COVID-19. This study is, to the best of our knowledge, the first to use excess mortality estimates in this way, allowing for the impact of COVID-19 vaccination to be estimated more accurately in countries with weaker surveillance systems.

**Implications of all the available evidence**

The results highlight the substantial impact that vaccination has had on the trajectory of the COVID-19 pandemic. They also illustrate the broader impact of COVID-19 vaccination in terms of allowing countries with high vaccine coverage to relax interventions. Furthermore, the findings highlight the importance of equitable access to vaccines, particularly in low-income regions, where substantially more lives could have been saved if the vaccination targets set out by the COVID-19 Vaccines Global Access (COVAX) Facility (20% coverage in COVAX Advance Market Commitment countries by the end of 2021) and WHO (40% coverage in each country by the end of 2021) had been reached.

To reduce inequality, a fair allocation mechanism for COVID-19 vaccines was developed through the COVID-19 Vaccines Global Access (COVAX) facility, with a key target of achieving 20% vaccine coverage for the countries covered by its Advance Market Commitment (AMC) through COVAX-secured doses by the end of 2021.^5^ WHO expanded this target by setting a global strategy to achieve 70% coverage in all countries by mid-2022, with an interim target of 40% coverage by the end of 2021.^7^ However, as a result of numerous challenges, particularly the constrained vaccine supply to COVAX (exacerbated by some countries obtaining a greater proportion of the global vaccine supply, pharmaceutical companies not meeting their contractual obligations to COVAX, and unpredictable delays in supply including vaccines with brief expiry windows), these targets were not reached in many lower-middle-income countries and low-income countries.^6^ Vaccine uptake has also been suboptimal in many countries because of vaccine hesitancy.^7^ This considerable heterogeneity in vaccination coverage has resulted in continued reliance on non-pharmaceutical interventions for pandemic management in some countries^8^ but concomitantly enabled other nations to relax interventions as a route out of the pandemic.^9^

Quantifying the impact of vaccination is further challenged by the incomplete picture of the COVID-19 pandemic that is obtained from reported deaths. In many countries, vital registration systems are incomplete and therefore only a fraction of deaths are routinely reported. However, even in countries with complete vital registration systems, it is difficult to accurately define the cause of death in individuals who present with multiple morbidities. Excess all-cause mortality (the difference between the observed and expected number of deaths in non-pandemic years) has therefore been used to quantify the impact of the COVID-19 pandemic.^10^ Although the exact contribution of COVID-19 to excess mortality is unknown, the strong temporal correlation observed globally between reported COVID-19 mortality and excess mortality provides evidence that excess mortality is an informative indicator of pandemic-related mortality.^11^ Robust vital registration systems do not exist in many parts of the world, with WHO estimating that 40% of global deaths that occurred in 2020 were unregistered,^12^ and therefore data on excess mortality are not available for every country. Model-based estimates have therefore been developed to obtain a more complete estimate of the pandemic to date. One set of estimates produced by *The Economist* uses a range of socioeconomic and epidemiological data to infer excess mortality.^13^ Although the precise estimates differ between research groups^14^ and WHO,^15^ they all suggest a substantially larger number of COVID-19 deaths than have been reported to date.

We aimed to quantify the global impact of the first year of COVID-19 vaccination and estimate the number of deaths from COVID-19 averted in 185 countries and territories, both from the direct protection of vaccinated individuals and from the indirect protection of all individuals living in vaccinated environments due to the reduction in risk of infection. Additionally, we aimed to quantify the impact that a more equitable global vaccination campaign, meeting the vaccination targets set by COVAX of 20% vaccination coverage of the eligible population by the end of 2021, could have had in COVAX AMC countries. We also aimed to quantify the impact of achieving the WHO target of 40% coverage by the end of 2021 in all countries.

## Methods

### Transmission model fitting

For this mathematical modelling study, we used a previously published COVID-19 transmission model^16^, ^17^ and fitting framework^18^ to obtain profiles of the COVID-19 pandemic in each country and thus estimate the counterfactual scenario in which vaccines are not delivered. Briefly, the model is a population-based, age-structured susceptible-exposed-infectious-recovered-susceptible (SEIRS) model, which explicitly captures disease severity, passage through different indicated health-care levels, and the roll-out of vaccination. We incorporated country-level data on demography, age-based mixing patterns, and health-care capacity. We fit the model to officially reported COVID-19 deaths in each country, resulting in an inferred time-varying level of transmission, *R*_t_, denoting the mean number of secondary infections in the absence of both infection-induced and vaccine-derived immunity. By fitting directly to mortality, we indirectly captured the impact that non-pharmaceutical interventions have had over the course of the COVID-19 pandemic.

Vaccination rates for first and second doses in each country were taken from Our World in Data^2^ and the WHO dashboard. We assumed a vaccination strategy that first targets those most at risk (including health-care workers) and then iteratively distributes vaccines in descending age order. Vaccination was assumed to confer protection against SARS-CoV-2 infection and the development of severe disease requiring hospital admission,^3^ and to reduce transmission from vaccine breakthrough infections (ie, we assumed vaccinated individuals who develop infection would be less infectious than unvaccinated individuals).^19^ We inferred vaccine efficacy for each country on the basis of vaccine types known to be predominantly used in each country. We explicitly modelled the emergence of the delta (B.1.617.2) variant and its impact on vaccine efficacy, hospital admissions, and immune escape.^20^, ^21^ Any epidemiological differences associated with previous variants were assumed to be reflected by their effects on mortality,^22^ which were subsequently captured by the estimated *R*_t_ trend. We fit the model to COVID-19 mortality in a Bayesian framework using a Metropolis-Hastings Markov Chain Monte Carlo-based sampling scheme. We used the resulting fit to estimate the time-varying reproductive number, *R*_t_, and its associated uncertainty.

Complete details of the model, vaccination, variants, and model fitting are given in the appendix (pp 2–10). No ethical concerns were noted for this study, with all mortality data used based on nationally aggregated statistics; all datasets used were publicly available.

### Excess mortality and COVID-19 mortality data

Because of the heterogeneity in death registration and certification worldwide, we also fit the model to all-cause excess mortality. For countries and time periods for which excess mortality had not been reported, we used model-based estimates of all-cause excess mortality, first produced by *The Economist*.^13^ More details of the methodology are given in the appendix (p 2). Given the wide uncertainty in these model-based estimates of excess mortality in many parts of the world, we also presented the deaths averted as estimated by fitting to official reported COVID-19 deaths from the Johns Hopkins University COVID-19 Data Repository (appendix p 2). Importantly, these estimates based on official reported COVID-19 deaths represent the lower bound of deaths averted at the global level due to the considerable levels of under-reporting of COVID-19 mortality documented worldwide.^23^

### Estimating deaths averted due to vaccination

The first vaccination outside a clinical trial setting was given on Dec 8, 2020. We introduced vaccination from this point onwards in the model and explored the impact of the first year of vaccination up to Dec 8, 2021. To quantify the impact of vaccination and its associated uncertainty, we took 100 draws from the estimated distribution of *R*_t_ and vaccine efficacy estimates for each country and simulated a counterfactual scenario in which no vaccines are available and the epidemic in each country follows the same *R*_t_ trend since the start of the pandemic; a counterfactual in which vaccines are delivered but there are no indirect effects (ie, they do not reduce SARS-CoV-2 transmission); and the observed scenario in which vaccines were delivered at the rates reported. The third scenario generated an estimate of the trajectory of the epidemic for our fitted model and hence closely matched reported COVID-19 or excess deaths or estimated excess deaths in each country. We calculated the deaths averted as a result of vaccination by subtracting the estimated COVID-19 deaths from the simulation with vaccines included (the observed scenario) from the estimated COVID-19 deaths under the first counterfactual scenario. This process is illustrated in the appendix (p 18), which shows the estimated deaths averted for the USA. Because of the difficulty in predicting how governments and populations would have responded, and how viral evolution would have progressed if vaccines had not been available, we made no attempt to adjust the *R*_t_ trends for further non-pharmaceutical interventions, changes in mobility, or development of variants that probably would have occurred differently in the absence of vaccination. To explore the impact of key model parameters on estimates of deaths averted, we did additional sensitivity analyses. These included characterising the effects of the assumed relationship between the infection fatality ratio (IFR) and age (appendix p 10), as well as the assumed degree of immune evasion exhibited by the delta variant (appendix p 7).

We also explored the impact of increasing vaccine distribution to meet WHO and COVAX targets. We modelled two scenarios in which the targets set by WHO to fully vaccinate 40% of the eligible population in each country and administrative region, and by COVAX to fully vaccinate 20% of the eligible population in AMC countries, by the end of 2021 had been reached. To do so, for countries in which these targets had not been met, we scaled the roll-out of vaccines across the year by a constant factor such that exactly the targeted amount of the population had received their second vaccine dose by our end date (Dec 8, 2021).

### Statistical analysis

All analyses were done with R software (version 4.1.3), with all data, code, packages, and versions used available online at GitHub. This analysis covered 185 countries and territories with a population greater than 90 000 as reported in *World Population Prospects 2019*,^24^ and that reported at least one death due to COVID-19 or 1 week of positive estimated excess mortality. We excluded China from our estimates because of its unique position as the origin of the detected epidemic and its large influence on estimates of deaths averted stemming from its population size.

### Role of the funding source

The sponsors of the study had no role in study design, data collection, data analysis, data interpretation, or writing of the report.

## Results

Based on our model fit to officially reported COVID-19 deaths, we estimated that 18·1 million (95% credible interval [CrI] 17·4–19·7) deaths due to COVID-19 would have occurred without vaccinations worldwide during the first year of the COVID-19 vaccination programme (Dec 8, 2020, to Dec 8, 2021). Of these, we estimated that vaccination prevented 14·4 million (95% CrI 13·7–15·9) deaths due to COVID-19, representing a global reduction of 79% of deaths (14·4 million of 18·1 million) during the first year of COVID-19 vaccination (table 1). These estimates of vaccine impact do not account for the potential under-ascertainment of deaths related to COVID-19.

**Table 1:** Estimated deaths averted in the first year of COVID-19 vaccinations worldwide based on fits to officially reported COVID-19 deaths

		**Total COVID-19 deaths**	**Vaccination coverage (%)**	**Estimated deaths averted by vaccinations**		
				Total	Per 10 000 people	Per 10 000 vaccines
Worldwide		5 469 000 (5 339 000–5 613 000)	38·30%	14 400 000 (13 650 000–15 900 000)	22·81 (21·63–25·18)	25·99 (24·64–28·69)
World Bank income group						
	High-income countries	1 956 000 (1 892 000–2 032 000)	68·80%	6 353 000 (6 105 000–6 604 000)	52·6 (50·54–54·67)	36·67 (35·23–38·11)
	Upper-middle-income countries	2 287 000 (2 220 000–2 355 000)	50·10%	2 914 000 (2 785 000–3 047 000)	25·6 (24·47–26·77)	23·36 (22·33–24·43)
	Lower-middle-income countries	1 188 000 (1 099 000–1 302 000)	29·80%	5 083 000 (4 379 000–6 628 000)	15·27 (13·16–19·91)	20·39 (17·57–26·59)
	Low-income countries	36 520 (33 390–40 410)	3·57%	20 380 (17 680–23 870)	0·3188 (0·2766–0·3733)	2·965 (2·572–3·472)
WHO region						
	African region	153 800 (145 100–164 700)	5·48%	97 190 (88 420–107 400)	0·8677 (0·7894–0·9589)	5·958 (5·420–6·584)
	Region of the Americas	2 492 000 (2 418 000–2 576 000)	58·30%	3 813 000 (3 624 000–3 987 000)	37·46 (35·6–39·17)	29·28 (27·83–30·62)
	Eastern Mediterranean region	318 700 (307 200–331 500)	28·10%	639 200 (581 600–707 700)	8·746 (7·958–9·684)	13·50 (12·28–14·95)
	European region	1 628 000 (1 589 000–1 673 000)	56·50%	4 334 000 (4 214 000–4 487 000)	46·77 (45·48–48·42)	39·52 (38·43–40·92)
	South-East Asian region	713 800 (635 900–807 000)	35·40%	3 913 000 (3 234 000–5 491 000)	19·61 (16·21–27·52)	21·63 (17·88–30·36)
	Western Pacific region	149 000 (120 100–234 400)	62·40%	1 574 000 (1 267 000–1 839 000)	30·14 (24·26–35·21)	22·58 (18·18–26·38)

Deaths averted are presented as medians with 95% credible intervals, with values also presented per 10 000 total population and per 10 000 vaccinations (first or second dose). Vaccination coverage is the proportion of the population with a full dose in the modelled countries by Dec 8, 2021. Total deaths are all modelled deaths in the presence of vaccinations when fitted to reported deaths from the start of the pandemic up to Dec 8, 2021.

Using our model fit to predicted and reported excess mortality (appendix p 23), we estimated that 31·4 million (95% CrI 30·6–32·1) deaths due to COVID-19 would have occurred without vaccinations during the first year of COVID-19 vaccination, with 19·8 million (95% CrI 19·1–20·4) deaths averted, corresponding to 63% (19·8 million of 31·4 million) of total deaths (table 2). The difference between vaccine impact estimates based on excess mortality and official deaths due to COVID-19 was greatest in low-income regions, with approximately ten times more deaths estimated to have been averted in low-income countries when relying on excess mortality estimates (appendix pp 13, 19).

**Table 2:** Estimated deaths averted in the first year of COVID-19 vaccinations worldwide based on fits to excess mortality

		**Total excess deaths**	**Estimated deaths averted by vaccinations**		
			Total	Per 10 000 people	Per 10 000 vaccines
Worldwide		17 990 000 (17 610 000–18 530 000)	19 810 000 (19 130 000–20 380 000)	31·21 (30·14–32·1)	35·68 (34·47–36·71)
World Bank income group					
	High-income countries	2 503 000 (2 412 000–2 609 000)	8 004 000 (7 644 000–8 438 000)	66·18 (63·20–69·77)	46·14 (44·07–48·64)
	Upper-middle-income countries	4 717 000 (4 611 000–4 827 000)	4 230 000 (4 051 000–4 384 000)	36·97 (35·40–38·31)	33·71 (32·28–34·94)
	Lower-middle-income countries	9 688 000 (9 329 000–10 170 000)	7 401 000 (6 841 000–7 655 000)	22·23 (20·55–23·00)	29·69 (27·44–30·71)
	Low-income countries	1 087 000 (1 068 000–1 106 000)	180 300 (171 400–188 900)	2·711 (2·576–2·840)	26·23 (24·93–27·48)
WHO region					
	African region	1 614 000 (1 580 000–1 652 000)	466 400 (446 300–487 000)	4·164 (3·985–4·348)	28·59 (27·36–29·85)
	Region of the Americas	3 354 000 (3 260 000–3 456 000)	4 469 000 (4 233 000–4 728 000)	43·89 (41·57–46·43)	34·31 (32·50–36·29)
	Eastern Mediterranean region	2 310 000 (2 248 000–2 376 000)	992 800 (938 800–1 066 000)	13·58 (12·85–14·59)	20·97 (19·83–22·52)
	European region	3 448 000 (3 347 000–3 568 000)	5 811 000 (5 551 000–6 187 000)	62·30 (59·51–66·33)	52·63 (50·28–56·04)
	South-East Asian region	6 741 000 (6 398 000–7 247 000)	5 658 000 (5 114 000–5 858 000)	27·99 (25·3–28·98)	31·29 (28·28–32·39)
	Western Pacific region	518 700 (489 200–547 800)	2 429 000 (2 266 000–2 617 000)	46·31 (43·21–49·91)	34·74 (32·42–37·44)

Deaths averted are presented as medians with 95% credible intervals, with values also presented per 10 000 total population and per 10 000 vaccinations (first or second dose). Total deaths are all modelled deaths in the presence of vaccinations when fitted to excess mortality from the start of the pandemic up to Dec 8, 2021.

**Figure 1:**
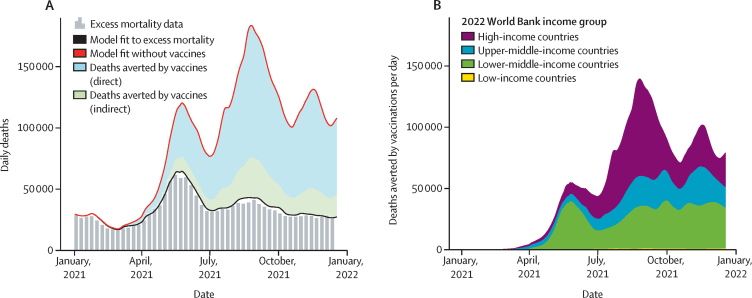
Global COVID-19 deaths averted due to vaccination based on excess mortality (A) Median number of daily COVID-19 deaths based on excess mortality estimates (grey vertical bars) in the first year of vaccination. The baseline estimate of daily COVID-19 deaths from the model fit to excess mortality is plotted with the solid black line and the counterfactual scenario without vaccines is plotted with a red line. The gap between the red and black line indicates the deaths averted due to vaccination, with the proportion of total deaths averted by direct protection conferred by vaccination shown in blue and indirect protection shown in green. (B) Median number of daily deaths averted per day as per 2022 World Bank income group.

Using our model fit to excess mortality, we estimated that most deaths averted were due to the high levels of individual-level direct protection conferred by vaccination, with 79% (15·5 million of 19·8 million) of deaths averted through direct protection (figure 1A). Vaccine impact was also conferred through reducing the levels of burden placed on health-care systems, reducing the number of days that health-care capacity would have been exceeded and therefore contributing to an overall lower fatality rate from infection (appendix p 20). Throughout 2021, vaccine impact changed over time and space. Vaccine impact was initially concentrated in lower-middle-income countries (figure 1B), resulting from the significant epidemic wave in India as the delta variant emerged. This was subsequently followed by vaccine impact being concentrated in high-income countries that were then either able to relax interventions due to high vaccination coverage (eg, the UK), or that did not implement further restrictions despite the spread of the more virulent delta variant in the second half of 2021.

**Figure 2:**
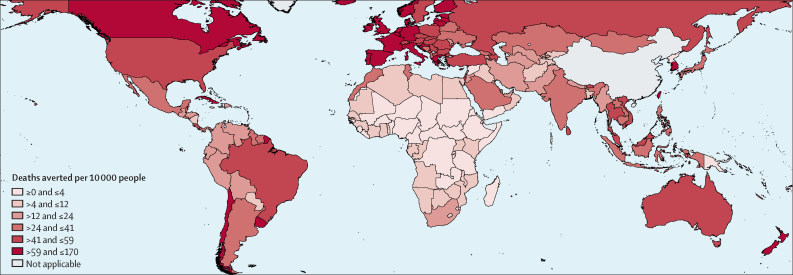
Median deaths averted by vaccinations per 10 000 people by country in the first year of COVID-19 vaccination Estimates of deaths averted were based on model fits to excess mortality and were binned within seven equal quantiles starting at 0 deaths averted. Deaths averted listed as not applicable for China because of its exclusion from our analysis, due to its unique position as the origin of the detected epidemic and large influence on estimates of deaths averted stemming from its population size.

Overall, estimated deaths averted per capita were highest in high-income countries, reflecting the earlier and wider roll-out of vaccination campaigns (table 2, figure 2; appendix p 13). We estimated that substantially more deaths were averted in the WHO European region. This was due to both the greater number of vaccinations administered in these regions and the higher levels of vaccine coverage achieved before the arrival of the delta variant.

**Figure 3:**
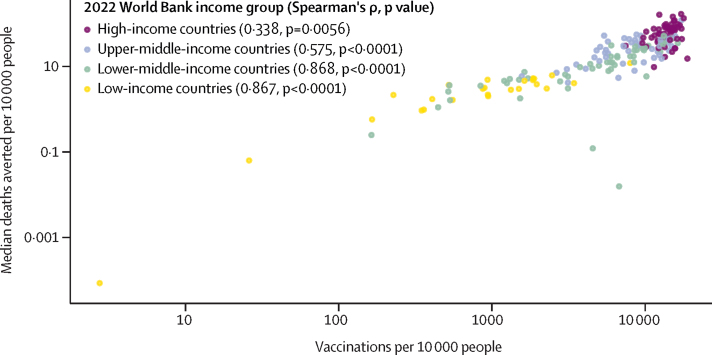
Median deaths averted by vaccinations per 10 000 against vaccinations per 10 000 for each country All measures are on the log-scale. Spearman's rank correlation coefficient (Spearman's ρ) is also given for each income group with a p value based on the Z score against a null hypothesis of no correlation. Countries that did not deliver any vaccinations or had no deaths averted are not included.

The estimated number of deaths averted per vaccine administered was notably higher in high-income countries and upper-middle-income countries, in part due to greater access to the more efficacious mRNA vaccines (table 2; appendix p 14). Across the geographical regions, the estimated number of deaths averted per vaccine administered was estimated to be significantly higher in the European region and significantly lower in the Eastern Mediterranean region, reflecting disparities in access to different vaccine types (appendix p 14) coupled with very high predicted excess mortality in several countries in the Eastern Mediterranean region (table 2). The disparity in the number of deaths averted per vaccine between the European region and the Western Pacific region, despite access to similar vaccine types, reflects the zero-COVID strategy adopted by some countries in the Western Pacific region, such as New Zealand, which resulted in smaller epidemics predicted in the no-vaccine counterfactual (table 2; appendix p 13). Conversely, we estimated the greatest vaccine impact to have occurred in high-income countries that did not pursue a zero-COVID strategy (appendix p 13), reflecting how maximising vaccination coverage was leveraged to re-open the economy, resulting in increased transmission and subsequently higher inferred *R*_t_ trends. When viewed across income strata, a linear log–log relationship was observed between per-capita deaths averted and vaccines administered (figure 3), with low-income countries estimated to have a lower vaccine impact resulting from lower vaccine coverage. This relationship was weakest within high-income countries, as all high-income countries had high levels of vaccinations per capita, with the variation in deaths averted explained by other heterogeneities in their epidemics, such as pursuing zero-COVID strategies.

For the 83 COVAX AMC countries modelled, using our model fit to excess mortality, we estimated that 17·9 million (95% CrI 17·2–18·5) deaths due to COVID-19 would have occurred without vaccinations during the first year of COVID-19 vaccination. We estimated that vaccinations averted 7·4 million (95% Crl 6·8–7·7) deaths, 41% (7·4 million of 17·9 million) of the deaths that would have occurred in COVAX AMC countries. Notably, the shortfall of the COVAX target in several regions was estimated to have resulted in an additional 156 900 (95% CrI 147 800–165 400) deaths (table 3). Although these deaths constituted a small proportion of the total deaths averted globally, these avertable deaths were concentrated in 25 low-income countries, which we predict would have averted an additional 81 750 (95% CrI 75 430–88 200) deaths across low-income countries by reaching 20% coverage, representing an additional 45% of deaths averted (table 3).

**Table 3:** Estimated increase in deaths averted in the first year of COVID-19 vaccinations worldwide based on fits to excess mortality had all countries met either of the COVAX or WHO vaccination targets

		**COVAX target (20% of eligible population in COVAX Advance Market Commitment countries fully dosed)**				**WHO target (40% of eligible population fully dosed)**			
		Countries failing target	Increased vaccine coverage (%)	Additional deaths averted	Additional deaths averted* (%)	Countries failing target	Increased vaccine coverage (%)	Additional deaths averted	Additional deaths averted* (%)
Worldwide		41	4·15%	156 900 (147 800–165 400)	0·792% (0·744–0·843)	96	27·8%	599 300 (577 700–622 400)	3·03% (2·89–3·17)
World Bank income group									
	High-income countries	..	..	..	..	1	0·00191%	20 (20–30)	0·000298% (0·000243–0·000342)
	Upper-middle-income countries	..	..	..	..	27	6·1%	51 110 (47 860–66 690)	1·21% (1·12–1·58)
	Lower-middle-income countries	16	4·28%	75 540 (68 640–80 380)	1·02% (0·923–1·13)	41	39·7%	347 500 (330 300–363 300)	4·71% (4·43– 5·11)
	Low-income countries	25	253%	81 750 (75 430–88 200)	45·2% (42·0–49·3)	27	1060%	200 000 (187 900–211 900)	111% (105–118)
WHO region									
	African region	31	134%	132 700 (123 800–141 300)	28·4% (26·5–30·4)	44	631%	348 900 (330 200–370 000)	74·9% (70·7–78·8)
	Region of the Americas	1	0·248%	1080 (850–1390)	0·0241% (0·0186–0·0308)	14	1·66%	6330 (5870–6840)	0·141% (0·129–0·155)
	Eastern Mediterranean region	6	5·56%	20 850 (18 860–22 710)	2·09% (1·86–2·32)	13	61·6%	126 800 (118 900–134 600)	12·7% (11·6–13·7)
	European region	..	..	..	..	13	3·05%	41 760 (38 110–46 160)	0·715% (0·644–0·799)
	South-East Asian region	1	0·586%	1410 (50–2960)	0·0254% (0·000914–0·0532)	7	17·5%	70 420 (64 300–75 890)	1·25% (1·15–1·39)
	African region	2	0·302%	900 (610–1200)	0·0366% (0·0250–0·0492)	5	2·59%	4990 (4390–5730)	0·205% (0·178–0·237)

Data are n (95% credible interval [CrI]) or % (95% CrI). All percentages are reported to 3 significant figures. Increased vaccination coverage is defined as the percentage increase in the proportion of the population with a full dose in all modelled countries when meeting the respective targets. Countries are grouped by 2022 World Bank income group and WHO region. COVAX=COVID-19 Vaccines Global Access.

* In proportion to total deaths averted by vaccines, as shown in table 2.

We found that 96 countries and administrative regions were below the WHO target of 40% vaccination coverage by the end of 2021. Had this target been met, we estimated that 599 300 (95% CrI 577 700–622 400) additional deaths would have been averted (table 3). The majority of these deaths occurred in lower-middle-income countries and the African and Eastern Mediterranean regions, although the largest proportional increase was seen in low-income countries, with the averted deaths making up a 111% increase in estimated deaths averted by vaccinations (table 3).

Our vaccine impact estimates were dependent on the assumed level of immune escape shown by the delta variant and the assumed relationship between age and the IFR. In the scenario in which the epidemic wave caused by the delta variant was comparable to previous waves and neither reached herd immunity nor resulted in health-system capacity being breached, our estimates of vaccine impact were unchanged regardless of the assumed level of immune evasion associated with the delta variant (appendix p 21). However, in scenarios in which the introduction of the delta variant produced a significantly larger wave that resulted in herd immunity being reached in the counterfactual, increased immune escape associated with the delta variant resulted in an increased number of averted deaths due to the larger effective size of the susceptible population. In sensitivity analyses in which the relationship between age and IFR was changed, we estimated that vaccine impact would be greater in scenarios with higher IFRs, reflecting the higher number of deaths that could be averted by vaccination (appendix p 22).

## Discussion

The high individual-level protection against severe disease and mortality due to COVID-19, as well as the population-level benefit afforded by mild protection against SARS-CoV-2 infection (before the emergence of the omicron [B.1.1.529] variant), conferred by vaccination, has fundamentally altered the course of the COVID-19 pandemic. Directly measuring the impact of vaccination programmes on COVID-19 mortality is not possible as the counterfactual (ie, without vaccinations) cannot be observed. Mathematical models are a valuable tool for quantifying the impact of vaccination campaigns on epidemic dynamics.^25^ We evaluated the impact of the first year of COVID-19 vaccination, revealing how vaccinations have more than halved the potential global death toll due to COVID-19, with an estimated 19·8 million deaths from COVID-19 averted as a result of vaccination, based on excess mortality estimates of the impact of the pandemic. These reductions were concentrated in high-income countries that relied on their vaccination programmes to relax interventions and allow SARS-CoV-2 transmission to increase as they moved into a new stage of the pandemic.

In low-income countries, particularly countries that did not reach the 20% targets set out by COVAX, vaccine impact was substantially lower, with vaccine impact estimated to have been almost doubled if the targets had been reached. If the 40% target, per country, from WHO had been met, we estimated a further increase in deaths averted, mainly focused in lower-middle-income countries and low-income countries. A limitation of our assessment of the COVAX and WHO targets is the timeframe of our analysis, as these targets were set to be reached by the end of 2021, whereas our modelling endpoint was Dec 8, 2021, to align with 1 year since the start of public vaccination. Hence, some countries might have moved closer to achieving the targets, or achieved them, by the end of the year. However, any recent vaccination drives would have had consequently negligible impact given the delay in developing protection and insufficient impact on COVID-19 dynamics.

Deriving estimates of vaccine impact is heavily dependent on the counterfactual scenario chosen. In our counterfactual, we assumed the same time-varying levels of SARS-CoV-2 transmission as estimated in our model fits. Consequently, the largest impact was observed in countries that delivered the most vaccinations to date and simultaneously relaxed interventions, allowing SARS-CoV-2 transmission to increase. However, several countries with slower vaccination roll-out as well as countries adopting a zero-COVID strategy maintained stronger interventions to suppress transmission and thus observed smaller impacts of their vaccination programmes as a result. As these countries start to reopen, we predict that vaccine impact estimates would increase in line with increasing levels of SARS-CoV-2 transmission.

Under-ascertainment of COVID-19 mortality is a known issue that has hindered our understanding of the pandemic.^23^ In this analysis, we consequently focused on fitting to all-cause excess mortality, which provides a more complete description of the pandemic.^15^ However, even when relying on model fits based on reported COVID-19 deaths, we estimated that more than 14 million deaths were averted by COVID-19 vaccination. The discrepancy between vaccine impact estimates based on excess mortality and COVID-19 deaths was concentrated in settings with lower death registration and certification. This substantial discrepancy underpins the crucial need for continued investment in civil registration and vital statistics to prevent biases in mortality reporting further minimising the perceived impact and necessity of vaccination in settings with lower reporting of deaths. In countries with more complete reporting systems, our estimates were broadly comparable to other endeavours focused on officially reported COVID-19 deaths and on understanding the direct impact of vaccination on people older than 60 years in Europe.^26^ We identified one study that estimated both the indirect and direct impact of vaccination, which again yielded estimates for vaccine impact in the USA that were similar to our impact estimates based on reported COVID-19 deaths.^27^

In our effort to provide impact estimates globally, we introduced various assumptions into our model. We were hindered by the global disparities in SARS-CoV-2 genomic surveillance and the absence of detailed vaccination data for the majority of countries. Consequently, key model inputs had to be created from working assumptions on which vaccines were delivered, how they were delivered, and when new variants of concern spread worldwide. We also assumed that the relationship between age and IFR was the same for each country. These assumptions would have affected our estimates of deaths averted, with sensitivity analyses showing that higher overall IFRs will increase the number of deaths that could be averted by vaccination. Our impact estimates were also limited by the inherent uncertainty in model-based estimates of excess mortality.^13^ These estimates are likely to have underestimated or overestimated COVID-19 death tolls in many countries. Notably, our model fits were unable to recreate excess mortality death tolls in recent epidemic waves in Iraq and Sudan because of the depletion of the susceptible population. These discrepancies could have been due to multiple reasons, including overestimated excess mortality, proportions of excess mortality not due to COVID-19,^28^ higher infection fatality rates by age in low-income settings than those estimated from high-income countries,^29^ and lower vaccine effectiveness than assumed in our framework. Last, our impact estimates were dependent on the assumed degree of immune escape that each variant of concern exhibits.^20^ If immune escape was higher than we assumed, more of the population would have been susceptible to re-infection and consequently more deaths from COVID-19 could have been averted by vaccination.

More broadly, our estimates should be considered in light of the considerable uncertainty inherent in estimating vaccine impact. Uncertainty in the true death toll of the pandemic, the circulating variants of concern and their immunological phenotypes, and the vaccines themselves administered in many countries vastly complicate efforts to derive accurate estimates of the impact of COVID-19 vaccines. However, the results of this analysis still provide a comprehensive and thorough assessment of the impact of COVID-19 vaccination, revealing the substantial impact that vaccines have had and the millions of lives that are likely to have been saved during the first year of vaccination. Despite this, more lives could have been saved if vaccines had been distributed more rapidly to many parts of the world and if vaccine uptake could have been strengthened worldwide. Reaching vaccination coverage targets and improving vaccine coverage globally is dependent on multiple factors and not solely dependent on improving vaccine donations.^30^ Vaccine intellectual property needs to be shared more quickly in the future, with more open technology and knowledge transfer surrounding vaccine production and allocation. Vaccine distribution and delivery infrastructure also needs to be scaled up worldwide and misinformation combatted to improve vaccine demand. Improvements must be made in all these areas to reach current vaccine targets and help ensure that vaccines are more equitably distributed in the future.

## Data sharing

All data, codes, and supplementary tables used and generated by this study are available in a GitHub repository (version 1.0.1) or the Zenodo open repository. All estimates of deaths averted from vaccination are available in the appendix (p 13).

## Declaration of interests

ACG has received personal consultancy fees from HSBC, GlaxoSmithKline, and WHO related to COVID-19 epidemiology and from The Global Fund to Fight AIDS, Tuberculosis and Malaria for work unrelated to COVID-19. ACG is a non-remunerated member of scientific advisory boards for Moderna and the Coalition for Epidemic Preparedness. ABH and PW have received personal consultancy related to COVID-19 work from WHO. All other authors declare no competing interests.
