# Supplementary material for: Global impact of the first year of COVID-19 vaccination: a mathematical modelling study
Source: Lancet Infect Dis. 2022 Sep;22(9):1293–302. doi: 10.1016/S1473-3099(22)00320-6 (PMC9225255; doi:10.1016/S1473-3099(22)00320-6)
Supplement: Supplementary appendix [file mmc1.pdf]

THE LANCET
Infectious Diseases

Supplementary appendix

This appendix formed part of the original submission and has been peer reviewed. We post it as supplied by the authors.

Supplement to: Watson OJ, Barnsley G, Toor J, et al. Global impact of the first year of COVID-19 vaccination: a mathematical modelling study. *Lancet Infect Dis* 2022; published online June 23. https://doi.org/10.1016/S1473-3099(22)00320-6.

Supplementary appendix: Global Impact of COVID-19 vaccination: a mathematical modelling study

Supplementary appendix: Global Impact of COVID-19 vaccination: a mathematical modelling study 1
- Supplementary Methods: 2
  - Data sources: 2
    - COVID-19 mortality and excess mortality 2
    - Vaccination 3
  - Model: 4
    - COVID-19 Transmission Model Overview 4
    - Vaccination Effectiveness 5
    - Variants of Concern 6
  - Model Fitting: 8
  - Impact of Healthcare Burden on Deaths Averted 9
  - Deaths Averted based on Reported Deaths 9
  - Infection Fatality Ratio Sensitivity Analysis 10
- Supplementary Tables 11
- Supplementary Figures 18
- Supplementary References 24
- National Excess Mortality Fits 28

1. Supplementary Methods:

1.1. Data sources:

1.1.1. COVID-19 mortality and excess mortality

Estimates of excess mortality are sourced from *The Economist* excess mortality model.[1] This model uses total mortality figures, where available, from *The Economist* Excess Death tracker[2] and the World Mortality Database,[3] and where unavailable, uses a model estimate. Such model estimates are made with a boosted regression tree machine learning method,[4] with excess deaths per 100,000 population from the aforementioned sources as the dependent variable, and a wide range of covariates as independent variables. These covariates include demographic, economic, geographical and political data, data on mobility based on cell phones, information on government policy responses to COVID-19, as well as available COVID-19 data on confirmed cases, confirmed deaths, and testing. Socio-economic datasets used are the World Development Indicators,[5] Varieties of Democracy Project,[6] Boix-Miller-Rosato Dichotomous Coding of Democracy,[7] Freedom in the World Report[8] and the World Health Organization (WHO) Global Health Workforce Statistics.[9] COVID-19 related deaths and infections are taken from the COVID-19 Data Repository (John Hopkins University),[10] data on vaccinations and testing were from Our World in Data,[11,12] and information on mobility and interventions are taken from Google COVID-19 Community Mobility Reports[13] and Oxford COVID-19 Government Response Tracker.[14] COVID-19 serology is taken from SeroTracker.[15] Additionally, mortality estimates for HIV,[16] Malaria,[17] and Tuberculosis[18] are included, all sourced from the WHO Global Health Observatory.[19]. Demographic data was sourced from the United Nations World Population Prospects database [20], which were combined with age-dependent estimates of IFR[21] to derive population-level IFRs. Geographical covariates include coordinates of the largest and capital cities,[22] mean elevation, percent of population in the tropics and coastal distances,[23] and mean distances between countries.[24]

For missing covariates an indicator variable is added to allow the regression tree to condition on them, as a missing covariate can be as informative as the data itself. Additionally, averages of daily COVID-19 cases and deaths, excess mortality, and serology by region, sub-region, continent, income group, and for adjacent countries and weighted by distance are included.

Interactions for the number of vaccinations and COVID-19 related deaths and cases are also included. Dimensionality reduction is made by moving to weekly data and removing covariates with more than 99% correlation. Where available for LMIC countries, the model is also trained on subregion data. Excess mortality estimates are updated daily and reported on a 7-day average basis (including additionally, if not part of the 7-day cadence, the current date). All model code and data sources for the Economist analysis are available at https://github.com/TheEconomist/covid-19-the-economist-global-excess-deaths-model.[1]

For our model fitting, we use the central estimates of excess deaths produced by *The Economist* model. The estimates used in this paper are taken from the model on the 7th of December 2021, with the exception of the fit for Curacao, which is taken from the model on the 20th of January 2022, to take advantage of improved estimates.

1.1.2. Vaccination

Vaccination data are taken from Our World in Data[12] and the WHO's Dashboard.[25] Where possible the number of people receiving first doses and the number receiving second doses each day are used or calculated from the total number of vaccinations, if needed. Preference is given to the smoothed rate of new vaccinations. Due to delays in the creation of frameworks for reporting vaccination data, many countries exhibit an anomalously large number of vaccinations on the first day of vaccination being reported. To account for such reporting delays, we linearly distribute the doses administered on the first day of vaccination over the previous 14 days, assuming the vaccination rate is comparable to the vaccination rate observed in the following 14 days. Additionally, second doses are adjusted so there are no second doses within the first 18 days of the start of the vaccination campaign in line with manufacturer recommended delays between first and second doses.

Missing values are interpolated such that the cumulative number of vaccinated people rises linearly. Where first dose and second dose data are lacking, the missing values are imputed such that the previous ratio of first dose vaccinated to second dose vaccinated is maintained. For countries without time-series data, we set up a reasonable schedule of vaccinations using the dates vaccinations become available and the doses recorded from the WHO vaccination dataset, with the number of second doses set to 0 for the first 18 days and then building to the

reported percentage by the end of the next 21 days. To extend the vaccinations up to the current day we assume that the doses given out each day remain at the final weekly average, with the proportion that are second doses also remaining at its weekly average, adjusted so that the number of second doses given out does not exceed the number of people with only first doses. As a simplifying assumption and where possible, single dose vaccines were assumed to have the same efficacy as the first dose of a two-dose vaccine schedule.

1.2. Model:

We use a previously published COVID-19 transmission model[26,27] and fitting framework[28] to fit the weekly estimated excess deaths in each country. In overview, the model is a population-based age-structured Susceptible-Exposed-Infected-Recovered model, which explicitly represents disease severity, passage through different healthcare levels and the roll out of vaccination. The full model is described in Hogan et al,[27] which we describe in overview here before detailing the specific extensions we have made to fit the model to national excess mortality data.

1.2.1. COVID-19 Transmission Model Overview

Hogan et al. extended a previously developed age-structured deterministic SEIR-type compartmental model of SARS-CoV-2 transmission[26] to include vaccination. This model explicitly incorporates the clinical pathway for those requiring hospitalisation, allowing estimates of the need for oxygen and/or intensive care support. Transmission depends on age-based contact matrices and a constant transmission rate per contact. Other risk groups or settings (such as healthcare workers or care homes) are not included. The model incorporates a latent period between infection and becoming infectious as well as age-dependent probabilities of hospitalisation and disease severity. The model also captures the loss of infection-derived immunity, assuming this follows an Erlang distribution with a mean duration of one year. The model assumes that all COVID-19 related deaths occur in those requiring hospitalisation and incorporates a limited healthcare capacity, causing increased mortality rates in those deprived of hospital or ICU beds. The vaccination pathway incorporates a delay in the development of protection and waning, both assumed to follow an Erlang distribution with a shape of two. Susceptible, latent, or recovered individuals can be vaccinated. Importantly, because latent individuals can be vaccinated, it is possible for latent individuals to develop vaccine derived

protection before realising their infection. However, due to the considerably shorter mean duration of a latent infection (4.6 days) compared to the delay for vaccine protection to be conferred (14 days), this effect will be minor.

Once vaccination is introduced into a population, the model vaccinates all eligible individuals at a rate determined by the time series of first doses as previously calculated. Once vaccination commences, a proportion of the entire adult population is vaccinated first to represent high-risk individuals. Then, vaccine prioritisation is assumed to be age-based, with 5-year age groups sequentially vaccinating beginning with the oldest age group, the 80+ year age group, then 75-80 years, and so on. Once 80% of the prioritised population within the target group is vaccinated, the next group is targeted. In countries with reported vaccination levels greater than 80% of the eligible population a final prioritisation stage increases maximum coverage to 95% in all groups. See Hogan et al.[27] and Walker et al.[26] for the mathematical details of the model.

1.2.2. Vaccination Effectiveness

We model three forms of vaccine efficacy: 1) efficacy against infection, 2) efficacy against severe disease (reducing risk of hospitalisation) in breakthrough infections and 3) reducing onward infectiousness of vaccinated individuals who become infected. We assume that protection is partial and that vaccine efficacy is the same for susceptible and immune individuals. For those protected by both vaccine-derived and infection-derived immunity, we assume that the most protective effect is dominant. For 3), we assume that all vaccinated individuals have a 50% reduction in infectiousness for breakthrough infections.[29] Both 1) and 2) have been shown to be dependent on the vaccine type, number of doses received, the time since first vaccination, the time between first dose and booster dose and the variants of concern circulating in the population. However, this information is largely absent for most countries. In addition, our compartmental framework models the vaccinated population as a whole and as such we use a weighting approach.

Given the absence of accurate data on the specific vaccine doses given in each country, we model the most commonly distributed known vaccine type in each country (Supplementary Table 5) as documented in the Unicef COVID-19 Vaccine Market Dashboard.[30] However, the majority of countries have used a combination of multiple types of vaccines and often the major

vaccine type is still Unknown (Supplementary Table 5). Consequently, the efficacy of the most commonly distributed vaccine is unlikely to reflect the population average vaccine efficacy. To account for this uncertainty, we allow vaccine efficacy to scale between chosen central values (reflecting the efficacy of the known dominant vaccine type) and the maximum and minimum vaccine efficacy across all vaccine types that have been used in that country. For example, in the United Kingdom our scaling parameter is centred on the efficacy for the mRNA vaccines (representing Pfizer, which was the dominant vaccine used in the UK by 8 Dec 2021) but can scale to the adenovirus values (representing AstraZeneca). The efficacy values for each vaccine type are shown in Supplementary Table 1. We have chosen to model vaccine types, e.g. grouping Moderna and Pfizer in the mRNA vaccine type, rather than representing specific vaccines, given the many unknowns in global vaccine roll out. The resultant efficacy values broadly reflect the range of estimated vaccine efficacies observed in response to wild-type and non-immune escape variants of concern.[31]

1.2.3. Variants of Concern

To capture the impact of variants of concern, we source the timing of new variants in each country where available directly from GISAID,[32] as compiled in CoVariants.[33] For countries without sequence data available on the start date of Delta, we use the median of countries in the same UN subregion, or in the same UN region if no sequences are available within the subregion. We assume that the timing of the Delta variant's arrival is related to the locality of the country and the average arrival times in the UN regions and subregions reflect transmission to neighbouring countries.

We model the impact of new variants through three model mechanisms each occurring over a 60-day period once Delta has been introduced:

- Infection hospitalisation rate increases linearly to 145% of the age specific hospitalisation rates for wild-type.[34,35]
- Vaccine effectiveness is linearly scaled down to the efficacies estimates against delta over this time to reflect lower protection against infection, see Supplementary Table 1.[36–38]

- Immune evasion for infection-derived immunity occurs for 27%[39] of the previously infected population. This is implemented by temporarily increasing the rate of transition from the recovered compartments to the susceptible compartments during the 60-day period.

Any increase in transmissibility due the Delta variant or any other VOC will be accounted for by the fitted fortnightly effects on the reproduction number.

We explored the possible impact of the mechanism of immune evasion from infection-derived immunity by conducting the sensitivity analyses shown in Supplementary Figure 4. To do this, we used synthetic data to explore two distinct scenarios. In the first scenario we assume a relatively low number of deaths distributed approximately uniformly across three epidemic waves. We assume that the Delta variant becomes dominant on the eve of the third wave and introduce the immune escape over this time. We then fit the model to the data with the MCMC approach and then estimate the deaths averted by vaccinations with different immune escape percentages ranging from 0% to 80%. This scenario is shown in Supplementary Figure 4c. In the second scenario, we explored a scenario with a large Delta wave and only one single small earlier wave (Supplementary Figure 4d). To simplify the model parameterisation, in both epidemics we assume a constant vaccine efficacy of 60% against infection and 90% against disease with vaccine roll out at a constant rate such that 80% of the population is vaccinated by the end of the epidemic.

The number of deaths averted across immune escape levels in the first scenario remained stable (Supplementary Figure 4a), beyond the noise explainable by the MCMC fitting process. However, we observed a positive relationship between the level of immune escape and deaths averted in the large Delta wave scenario (Supplementary Figure 4b). A potential explanation is that in the first scenario the counterfactual deaths do not overwhelm the healthcare capacity of the country before reaching herd immunity hence any potential in transmission from the immune escape is controlled by a reduction in R_t from the fitting process. However, in the second scenario the counterfactual deaths exceed healthcare capacity and hence the mortality rate increases further beyond the amount controlled for by the model fitting process. Therefore, we only expect immune escape to have an impact on deaths averted where we see large amounts

of deaths associated with the Delta wave. For these situations when fitting to the excess mortality fits we often require the immune escape to accurately match the data.

1.3. Model Fitting

For the fits to excess-mortality we fit the model to the weekly time series of estimated excess deaths, D_t, or the reported weekly COVID-19 deaths, whichever is higher. For the fits based upon reported deaths, D_t is the daily number of deaths. We assume the likelihood is described by a Negative Binomial distribution, which is given by:

$$D_t = NB(\mu, \sigma)$$

where NB is the Negative Binomial distribution, with standard deviation σ and mean μ, the model estimate of deaths in the given time-period. The standard deviation σ can be expressed as $\sqrt{\mu + \mu^2/r}$, where r is the dispersion parameter and assumed to be equal to 7 to account for overdispersion. Additionally, the total number of deaths related to the epidemic is assumed to be described by the Negative Binomial distribution with a dispersion parameter of 40. The dispersion parameters chosen here (7 for weekly data and 40 for cumulative total deaths) were chosen to capture the reduced variance when moving from daily reported death date (previously estimated to approximately capture the variance in daily reported deaths.[40])

The model is fit to D_t by inferring the epidemic start date, the reproduction number at the start of the epidemic, R_0, the vaccine efficacy scaling parameter, and the time varying reproduction number, R_t, updated every two weeks.The last change in R_t is maintained for the last 14-28 days prior to the current day to reflect our inability to estimate the effect size of this parameter due to the approximate 21-day delay between infection and death.

Model fitting was carried within a Bayesian framework, using a Metropolis-Hastings Markov Chain Monte Carlo (MCMC) based sampling scheme with parallel tempering using at least 12 temperature rungs, implemented in the R package *drjacoby*.[41] All parameter inference results

reported here are based on 100 samples from the posterior with effective samples sizes assessed to check that chains had suitably explored the parameter space. The prior distributions and ranges for each parameter estimated used are given in Supplementary Table 2.

1.4. Impact of Healthcare Burden on Deaths Averted

By comparing deaths in the baseline model and counterfactuals with no vaccinations or no protection against transmission and infection we can explore the deaths averted by direct and indirect effects of vaccination. However, as healthcare capacity can be exceeded in this model this too has an impact on deaths, with an estimated 2.77 million (2.63-2.96 million) deaths caused by exceeding healthcare capacity in the excess mortality-based estimates. Expanding the counterfactual modelling framework to include counterfactuals with no limit to healthcare capacity we can split the cause of deaths averted into those by increased access to healthcare (Supplementary Figure 3). Direct protection remains the main driver of deaths averted and very few deaths are averted by the effects of the indirect protection on healthcare capacity. On average, more deaths are averted by the reduction in healthcare capacity from direct protection, 4.44 million, than are averted by indirect protection, 3.27 million.

1.5. Deaths Averted based on Reported Deaths

We also estimate deaths averted by vaccinations based on reported deaths (Table 1). This analysis includes all countries and administrative regions modelled in squire that have reported at least one COVID-19 death, excluding China and countries/regions that have less than a population of less than 90,000 in the UN World Population Prospects 2019. This model is fitted to daily reported COVID-19 death data from John Hopkins[10] and Worldometer.[42] As such the model likelihood is modified to have a lower variance with dispersion parameter equal to 2, which has been estimated previously to approximately capture the variance in daily reported deaths.[40]

Comparing deaths averted across fits included in both analyses, that the smallest difference occurs in HIC countries (Supplementary Figure 2). This is expected as HIC have greater testing capacities and so excess mortality curves are similar to the reported death curves. UMIC and LMIC countries represent the majority of the difference in deaths averted between the two global

estimates. LIC countries also have a proportionally large increase in deaths averted however with limited access to vaccines the number of deaths averted is low.

1.6. Infection Fatality Ratio Sensitivity Analysis

To explore the impact of the assumed IFR on the number of deaths averted, we conducted a sensitivity analysis on synthetic data for two archetypal epidemics. The two scenarios used where a death curve with three small peaks and a high death scenario with a large delta wave, similar to those depicted in supplementary figure 1c and 1d, though with the high death scenario scaled down to allow fitting at the lowest end of the IFR ranges, supplementary figure 4c and d.

We explore a range of IFRs across the age specific 95% confidence intervals reported in Brazeau et al. 2020.[21] Using a scaling parameter we explored these values symmetrically around the central estimate used in this paper. To match our desired IFR we scale the probability of hospitalisation for the infected, and refit the model to the scenario death curves, shown in supplementary figure 4c and d. For this analysis we assume no immune escape with the Delta variant, and allow vaccine efficacy to vary in the fitting process.

We find evidence of a positive trend between the infection fatality ratio and the number of deaths averted by vaccinations, in both scenarios see supplementary figure 4a and b. This is because higher IFR leads to a model fit with low levels of naturally acquired immunity and so the immunity acquired from vaccinations becomes more impactful.

Lastly, the relationship between IFR and age is maintained constant throughout simulations. However, we do not model a changing population age over time and we do not model births in our framework because the 2 year model duration will make this impact negligible. As a result, when individuals die in the model, they are not replaced as would happen in a constant population size model. Consequently, after significant epidemic waves, the average population age will decrease as a result of the higher mortality due to COVID-19 in older age groups resulting in the population average IFR decreasing over time.

2. Supplementary Tables

Supplementary Table 1: Assumed central vaccine efficacies against infection and disease for each Vaccine and Variant Type.[31,38,43–47]

Vaccine Type	Variant	Protection against Infection		Protection against Disease	
		First Dose	Second Dose	First Dose	Second Dose
Adenovirus	Wild Type (Non-Delta)	64%	77%	79%	92%
	Delta	30%	67%	71%	92%
Johnson & Johnson	Wild Type (Non-Delta)	66%	-	83%	-
	Delta	50%	-	74%	-
mRNA	Wild Type (Non-Delta)	63%	86%	83%	95%
	Delta	36%	88%	83%	93%
Subunit	Wild Type (Non-Delta)	54%	86%	83%	96%
	Delta	30%	71%	68%	86%
Whole Virus	Wild Type (Non-Delta)	50%	67%	50%	79%
	Delta	10%	60%	14%	70%

Supplementary Table 2: Prior distributions and ranges

Parameter	Distribution	Prior	Range	Notes
R_0	Uniform	-	[1, 10]	-
R_t/R_{t-1}	F	$d_1 = 40$, $d_2 = 40$	[0, 10] (on Rt)	Reflects expectation of similarity between Rt value and subsequent Rt values and maintains near symmetry in increases/decreases
Start Date	Uniform	-	10 - 55 days before first reported death	-
Vaccine Efficacy Scaling	Normal	Mean = 1/2, sd = 0.1	[0, 1]	Calculated so that range is equivalent to 95% confidence levels

Supplementary Table 3: Country specific deaths averted for the excess mortality fits.

Estimates of baseline deaths and deaths averted for each country used in the analysis. Reduction in deaths if the COVAX and WHO targets are met are also presented. All estimates are made using the same set of parameter draws in each country. All figures and values related to excess mortality in this paper are drawn from the results summarised here.

Table available to download at:

https://github.com/mrc-ide/covid-vaccine-impact-orderly/releases/download/v1.0.1/excess_mortality_summary_table.csv [48]

Supplementary Table 4: Country specific deaths averted for the reported deaths fits.

Estimates of baseline deaths and deaths averted for each country used in the analysis. All estimates are made using the same set of parameter draws in each country. All figures and values related to reported deaths in this paper are drawn from the results summarised here.

Table available to download at:

https://github.com/mrc-ide/covid-vaccine-impact-orderly/releases/download/v1.0.1/reported_death_summary_table.csv [48]

Supplementary Table 5: Dominant vaccine types in each country.

The most commonly distributed vaccine type in each country as documented in the Unicef COVID-19 Vaccine Market Dashboard,[30] which were used to define the central vaccine efficacy estimate for each country. For a number of countries, the major vaccine type is Unknown, in which case the most commonly distributed known vaccine type was assumed.

Table also available to download at:

https://github.com/mrc-ide/covid-vaccine-impact-orderly/releases/download/v1.0.1/dominant_vaccines.csv [48]

ISO3C	Country	Dominant Vaccine Type	Dominant Vaccine Type Excluding Unknown
AFG	Afghanistan	Janssen – Ad26.COV 2.S	Janssen – Ad26.COV 2.S
ALB	Albania	Unknown	Pfizer BioNTech – Comirnaty
DZA	Algeria	Sinovac – CoronaVac	Sinovac – CoronaVac
AGO	Angola	Pfizer BioNTech – Comirnaty	Pfizer BioNTech – Comirnaty
ATG	Antigua & Barbuda	Pfizer BioNTech – Comirnaty	Pfizer BioNTech – Comirnaty
ARG	Argentina	Sinopharm (Beijing) – BBIBP-CorV	Sinopharm (Beijing) – BBIBP-CorV
ARM	Armenia	Moderna – Spikevax	Moderna – Spikevax
ABW	Aruba	Unknown	Pfizer BioNTech – Comirnaty
AUS	Australia	Pfizer BioNTech – Comirnaty	Pfizer BioNTech – Comirnaty
AUT	Austria	Pfizer BioNTech – Comirnaty	Pfizer BioNTech – Comirnaty
AZE	Azerbaijan	Unknown	Pfizer BioNTech – Comirnaty
BHS	Bahamas	Pfizer BioNTech – Comirnaty	Pfizer BioNTech – Comirnaty
BHR	Bahrain	Unknown	Sinopharm (Beijing) – BBIBP-CorV
BGD	Bangladesh	Sinopharm (Beijing) – BBIBP-CorV	Sinopharm (Beijing) – BBIBP-CorV
BRB	Barbados	AstraZeneca – Vaxzevria	AstraZeneca – Vaxzevria
BLR	Belarus	Sinopharm (Beijing) – BBIBP-CorV	Sinopharm (Beijing) – BBIBP-CorV
BEL	Belgium	Pfizer BioNTech – Comirnaty	Pfizer BioNTech – Comirnaty
BLZ	Belize	AstraZeneca – Vaxzevria	AstraZeneca – Vaxzevria
BEN	Benin	Janssen – Ad26.COV 2.S	Janssen – Ad26.COV 2.S
BTN	Bhutan	SII – Covishield	SII – Covishield
BOL	Bolivia	Sinopharm/Beijing	Sinopharm (Beijing) – BBIBP-CorV
BIH	Bosnia & Herzegovina	AstraZeneca – Vaxzevria	AstraZeneca – Vaxzevria
BWA	Botswana	Janssen – Ad26.COV 2.S	Janssen – Ad26.COV 2.S
BRA	Brazil	Pfizer BioNTech – Comirnaty	Pfizer BioNTech – Comirnaty
BRN	Brunei	AstraZeneca – Vaxzevria	AstraZeneca – Vaxzevria
BGR	Bulgaria	Pfizer BioNTech – Comirnaty	Pfizer BioNTech – Comirnaty
BFA	Burkina Faso	Janssen – Ad26.COV 2.S	Janssen – Ad26.COV 2.S
BDI	Burundi	Sinopharm (Beijing) – BBIBP-CorV	Sinopharm (Beijing) – BBIBP-CorV
KHM	Cambodia	Sinovac – CoronaVac	Sinovac – CoronaVac
CMR	Cameroon	Janssen – Ad26.COV 2.S	Janssen – Ad26.COV 2.S
CAN	Canada	Pfizer BioNTech – Comirnaty	Pfizer BioNTech – Comirnaty
CPV	Cape Verde	AstraZeneca – Vaxzevria	AstraZeneca – Vaxzevria
CAF	Central African Republic	Janssen – Ad26.COV 2.S	Janssen – Ad26.COV 2.S
TCD	Chad	Janssen – Ad26.COV 2.S	Janssen – Ad26.COV 2.S
CHL	Chile	Sinovac – CoronaVac	Sinovac – CoronaVac
COL	Colombia	Sinovac – CoronaVac	Sinovac – CoronaVac

COM	Comoros	Unknown	Sinopharm (Beijing) – BBIBP-CorV
NA	Congo - Brazzaville	Janssen – Ad26.COV 2.S	Janssen – Ad26.COV 2.S
COD	Congo - Kinshasa	Janssen – Ad26.COV 2.S	Janssen – Ad26.COV 2.S
CRI	Costa Rica	Pfizer BioNTech – Comirnaty	Pfizer BioNTech – Comirnaty
CIV	Côte d'Ivoire	Pfizer BioNTech – Comirnaty	Pfizer BioNTech – Comirnaty
HRV	Croatia	Pfizer BioNTech – Comirnaty	Pfizer BioNTech – Comirnaty
CUB	Cuba	Unknown	Abdala (Subunit like novavax)
CUW	Curaçao	Moderna – Spikevax	Moderna – Spikevax
CYP	Cyprus	Pfizer BioNTech – Comirnaty	Pfizer BioNTech – Comirnaty
CZE	Czechia	Pfizer BioNTech – Comirnaty	Pfizer BioNTech – Comirnaty
DNK	Denmark	Pfizer BioNTech – Comirnaty	Pfizer BioNTech – Comirnaty
DJI	Djibouti	Janssen – Ad26.COV 2.S	Janssen – Ad26.COV 2.S
DOM	Dominican Republic	Sinovac – CoronaVac	Sinovac – CoronaVac
ECU	Ecuador	Sinovac – CoronaVac	Sinovac – CoronaVac
EGY	Egypt	AstraZeneca – Vaxzevria	AstraZeneca – Vaxzevria
SLV	El Salvador	Sinovac – CoronaVac	Sinovac – CoronaVac
GNQ	Equatorial Guinea	Sinopharm (Beijing) – BBIBP-CorV	Sinopharm (Beijing) – BBIBP-CorV
ERI	Eritrea	NA	NA
EST	Estonia	Pfizer BioNTech – Comirnaty	Pfizer BioNTech – Comirnaty
SWZ	Eswatini	Janssen – Ad26.COV 2.S	Janssen – Ad26.COV 2.S
ETH	Ethiopia	Janssen – Ad26.COV 2.S	Janssen – Ad26.COV 2.S
FJI	Fiji	AstraZeneca – Vaxzevria	AstraZeneca – Vaxzevria
FIN	Finland	Pfizer BioNTech – Comirnaty	Pfizer BioNTech – Comirnaty
FRA	France	Pfizer BioNTech – Comirnaty	Pfizer BioNTech – Comirnaty
PYF	French Polynesia	Unknown	Janssen – Ad26.COV 2.S
GAB	Gabon	Janssen – Ad26.COV 2.S	Janssen – Ad26.COV 2.S
GMB	Gambia	Janssen – Ad26.COV 2.S	Janssen – Ad26.COV 2.S
GEO	Georgia	Sinopharm (Beijing) – BBIBP-CorV	Sinopharm (Beijing) – BBIBP-CorV
DEU	Germany	Pfizer BioNTech – Comirnaty	Pfizer BioNTech – Comirnaty
GHA	Ghana	Janssen – Ad26.COV 2.S	Janssen – Ad26.COV 2.S
GRC	Greece	Pfizer BioNTech – Comirnaty	Pfizer BioNTech – Comirnaty
GRD	Grenada	Pfizer BioNTech – Comirnaty	Pfizer BioNTech – Comirnaty
GTM	Guatemala	Moderna – Spikevax	Moderna – Spikevax
GIN	Guinea	Sinovac – CoronaVac	Sinovac – CoronaVac
GNB	Guinea-Bissau	Janssen – Ad26.COV 2.S	Janssen – Ad26.COV 2.S
GUY	Guyana	Gamaleya - Sputnik V	Gamaleya - Sputnik V
HTI	Haiti	Moderna – Spikevax	Moderna – Spikevax
HND	Honduras	Moderna – Spikevax	Moderna – Spikevax
HKG	Hong Kong SAR China	Pfizer BioNTech – Comirnaty	Pfizer BioNTech – Comirnaty
HUN	Hungary	Pfizer BioNTech – Comirnaty	Pfizer BioNTech – Comirnaty
ISL	Iceland	Pfizer BioNTech – Comirnaty	Pfizer BioNTech – Comirnaty
IND	India	SII – Covishield	SII – Covishield
IDN	Indonesia	Sinovac – CoronaVac	Sinovac – CoronaVac
NA	Iran	Sinovac – CoronaVac	Sinovac – CoronaVac
IRQ	Iraq	Pfizer BioNTech – Comirnaty	Pfizer BioNTech – Comirnaty
IRL	Ireland	Pfizer BioNTech – Comirnaty	Pfizer BioNTech – Comirnaty
ISR	Israel	Unknown	Pfizer BioNTech – Comirnaty
ITA	Italy	Pfizer BioNTech – Comirnaty	Pfizer BioNTech – Comirnaty
JAM	Jamaica	AstraZeneca – Vaxzevria	AstraZeneca – Vaxzevria
JPN	Japan	Pfizer BioNTech – Comirnaty	Pfizer BioNTech – Comirnaty
JOR	Jordan	Unknown	Pfizer BioNTech – Comirnaty
KAZ	Kazakhstan	Unknown	Sinopharm (Beijing) – BBIBP-CorV
KEN	Kenya	AstraZeneca – Vaxzevria	AstraZeneca – Vaxzevria

KWT	Kuwait	Unknown	Covishield
KGZ	Kyrgyzstan	Sinopharm (Beijing) – BBIBP-CorV	Sinopharm (Beijing) – BBIBP-CorV
LAO	Laos	Sinopharm (Beijing) – BBIBP-CorV	Sinopharm (Beijing) – BBIBP-CorV
LVA	Latvia	Pfizer BioNTech – Comirnaty	Pfizer BioNTech – Comirnaty
LBN	Lebanon	Unknown	Pfizer BioNTech – Comirnaty
LSO	Lesotho	Janssen – Ad26.COV 2.S	Janssen – Ad26.COV 2.S
LBR	Liberia	Janssen – Ad26.COV 2.S	Janssen – Ad26.COV 2.S
LBY	Libya	Sinopharm (Beijing) – BBIBP-CorV	Sinopharm (Beijing) – BBIBP-CorV
LTU	Lithuania	Pfizer BioNTech – Comirnaty	Pfizer BioNTech – Comirnaty
LUX	Luxembourg	Pfizer BioNTech – Comirnaty	Pfizer BioNTech – Comirnaty
MAC	Macao SAR China	Pfizer BioNTech – Comirnaty	Pfizer BioNTech – Comirnaty
MDG	Madagascar	Janssen – Ad26.COV 2.S	Janssen – Ad26.COV 2.S
MWI	Malawi	AstraZeneca – Vaxzevria	AstraZeneca – Vaxzevria
MYS	Malaysia	Unknown	Sinovac – CoronaVac
MDV	Maldives	Pfizer BioNTech – Comirnaty	Pfizer BioNTech – Comirnaty
MLI	Mali	Sinovac – CoronaVac	Sinovac – CoronaVac
MLT	Malta	Unknown	Pfizer BioNTech – Comirnaty
MRT	Mauritania	Janssen – Ad26.COV 2.S	Janssen – Ad26.COV 2.S
MUS	Mauritius	Unknown	Janssen – Ad26.COV 2.S
MEX	Mexico	AstraZeneca – Vaxzevria	AstraZeneca – Vaxzevria
MDA	Moldova	Pfizer BioNTech – Comirnaty	Pfizer BioNTech – Comirnaty
MNG	Mongolia	Sinopharm (Beijing) – BBIBP-CorV	Sinopharm (Beijing) – BBIBP-CorV
MNE	Montenegro	Sinopharm (Beijing) – BBIBP-CorV	Sinopharm (Beijing) – BBIBP-CorV
MAR	Morocco	Unknown	Sinopharm (Beijing) – BBIBP-CorV
MOZ	Mozambique	Sinopharm (Beijing) – BBIBP-CorV	Sinopharm (Beijing) – BBIBP-CorV
MMR	Myanmar (Burma)	Sinopharm (Beijing) – BBIBP-CorV	Sinopharm (Beijing) – BBIBP-CorV
NAM	Namibia	Janssen – Ad26.COV 2.S	Janssen – Ad26.COV 2.S
NPL	Nepal	Unknown	Sinopharm (Beijing) – BBIBP-CorV
NLD	Netherlands	Pfizer BioNTech – Comirnaty	Pfizer BioNTech – Comirnaty
NZL	New Zealand	Unknown	Pfizer BioNTech – Comirnaty
NIC	Nicaragua	Unknown	AstraZeneca – Vaxzevria
NER	Niger	Janssen – Ad26.COV 2.S	Janssen – Ad26.COV 2.S
NGA	Nigeria	AstraZeneca – Vaxzevria	AstraZeneca – Vaxzevria
PRK	North Korea	NA	NA
MKD	North Macedonia	Unknown	Pfizer BioNTech – Comirnaty
NOR	Norway	Pfizer BioNTech – Comirnaty	Pfizer BioNTech – Comirnaty
OMN	Oman	Unknown	Pfizer BioNTech – Comirnaty
PAK	Pakistan	Sinovac – CoronaVac	Sinovac – CoronaVac
PSE	Palestinian Territories	Moderna – Spikevax	Moderna – Spikevax
PAN	Panama	Pfizer BioNTech – Comirnaty	Pfizer BioNTech – Comirnaty
PNG	Papua New Guinea	Janssen – Ad26.COV 2.S	Janssen – Ad26.COV 2.S
PRY	Paraguay	Pfizer BioNTech – Comirnaty	Pfizer BioNTech – Comirnaty
PER	Peru	Pfizer BioNTech – Comirnaty	Pfizer BioNTech – Comirnaty
PHL	Philippines	Pfizer BioNTech – Comirnaty	Pfizer BioNTech – Comirnaty
POL	Poland	Pfizer BioNTech – Comirnaty	Pfizer BioNTech – Comirnaty
PRT	Portugal	Pfizer BioNTech – Comirnaty	Pfizer BioNTech – Comirnaty
QAT	Qatar	AstraZeneca – Vaxzevria	AstraZeneca – Vaxzevria
ROU	Romania	Pfizer BioNTech – Comirnaty	Pfizer BioNTech – Comirnaty
RUS	Russia	Unknown	Gamaleya - Sputnik V
RWA	Rwanda	AstraZeneca – Vaxzevria	AstraZeneca – Vaxzevria
STP	Sao Tome and Principe	AstraZeneca – Vaxzevria	AstraZeneca – Vaxzevria
SAU	Saudi Arabia	Unknown	SII – Covishield
SEN	Senegal	Janssen – Ad26.COV 2.S	Janssen – Ad26.COV 2.S

SRB	Serbia	Sinopharm (Beijing) – BBIBP-CorV	Sinopharm (Beijing) – BBIBP-CorV
SYC	Seychelles	Sinopharm (Beijing) – BBIBP-CorV	Sinopharm (Beijing) – BBIBP-CorV
SLE	Sierra Leone	Janssen – Ad26.COV 2.S	Janssen – Ad26.COV 2.S
SGP	Singapore	Unknown	Pfizer BioNTech – Comirnaty
SVK	Slovakia	Pfizer BioNTech – Comirnaty	Pfizer BioNTech – Comirnaty
SVN	Slovenia	Pfizer BioNTech – Comirnaty	Pfizer BioNTech – Comirnaty
SLB	Solomon Islands	AstraZeneca – Vaxzevria	AstraZeneca – Vaxzevria
SOM	Somalia	Janssen – Ad26.COV 2.S	Janssen – Ad26.COV 2.S
ZAF	South Africa	Pfizer BioNTech – Comirnaty	Pfizer BioNTech – Comirnaty
KOR	South Korea	Pfizer BioNTech – Comirnaty	Pfizer BioNTech – Comirnaty
SSD	South Sudan	Janssen – Ad26.COV 2.S	Janssen – Ad26.COV 2.S
ESP	Spain	Pfizer BioNTech – Comirnaty	Pfizer BioNTech – Comirnaty
LKA	Sri Lanka	Sinopharm (Beijing) – BBIBP-CorV	Sinopharm (Beijing) – BBIBP-CorV
LCA	St. Lucia	AstraZeneca – Vaxzevria	AstraZeneca – Vaxzevria
VCT	St. Vincent & Grenadines	AstraZeneca – Vaxzevria	AstraZeneca – Vaxzevria
SDN	Sudan	Janssen – Ad26.COV 2.S	Janssen – Ad26.COV 2.S
SUR	Suriname	AstraZeneca – Vaxzevria	AstraZeneca – Vaxzevria
SWE	Sweden	Pfizer BioNTech – Comirnaty	Pfizer BioNTech – Comirnaty
CHE	Switzerland	Unknown	Moderna – Spikevax
SYR	Syria	Janssen – Ad26.COV 2.S	Janssen – Ad26.COV 2.S
TWN	Taiwan	AstraZeneca – Vaxzevria	AstraZeneca – Vaxzevria
TJK	Tajikistan	Unknown	Sinovac – CoronaVac
TZA	Tanzania	Pfizer BioNTech – Comirnaty	Pfizer BioNTech – Comirnaty
THA	Thailand	Unknown	AstraZeneca – Vaxzevria
TLS	Timor-Leste	Unknown	AstraZeneca – Vaxzevria
TGO	Togo	Janssen – Ad26.COV 2.S	Janssen – Ad26.COV 2.S
TTO	Trinidad & Tobago	Sinopharm (Beijing) – BBIBP-CorV	Sinopharm (Beijing) – BBIBP-CorV
TUN	Tunisia	Unknown	AstraZeneca – Vaxzevria
TUR	Turkey	Unknown	Sinovac – CoronaVac
TKM	Turkmenistan	Unknown	Gamaleya - Sputnik V
UGA	Uganda	Janssen – Ad26.COV 2.S	Janssen – Ad26.COV 2.S
UKR	Ukraine	Unknown	Pfizer BioNTech – Comirnaty
ARE	United Arab Emirates	Unknown	Sinopharm (Beijing) – BBIBP-CorV
GBR	United Kingdom	Pfizer BioNTech – Comirnaty	Pfizer BioNTech – Comirnaty
USA	United States	Pfizer BioNTech – Comirnaty	Pfizer BioNTech – Comirnaty
URY	Uruguay	Unknown	Sinovac – CoronaVac
UZB	Uzbekistan	Anhui ZL - Recombinant SARS-CoV-2 vaccine	Anhui ZL - Recombinant SARS-CoV-2 vaccine
VUT	Vanuatu	Sinopharm (Beijing) – BBIBP-CorV	Sinopharm (Beijing) – BBIBP-CorV
VEN	Venezuela	Sinopharm (Beijing) – BBIBP-CorV	Sinopharm (Beijing) – BBIBP-CorV
VNM	Vietnam	Unknown	AstraZeneca – Vaxzevria
YEM	Yemen	Janssen – Ad26.COV 2.S	Janssen – Ad26.COV 2.S
ZMB	Zambia	Janssen – Ad26.COV 2.S	Janssen – Ad26.COV 2.S
ZWE	Zimbabwe	Sinopharm (Beijing) – BBIBP-CorV	Sinopharm (Beijing) – BBIBP-CorV

3. Supplementary Figures

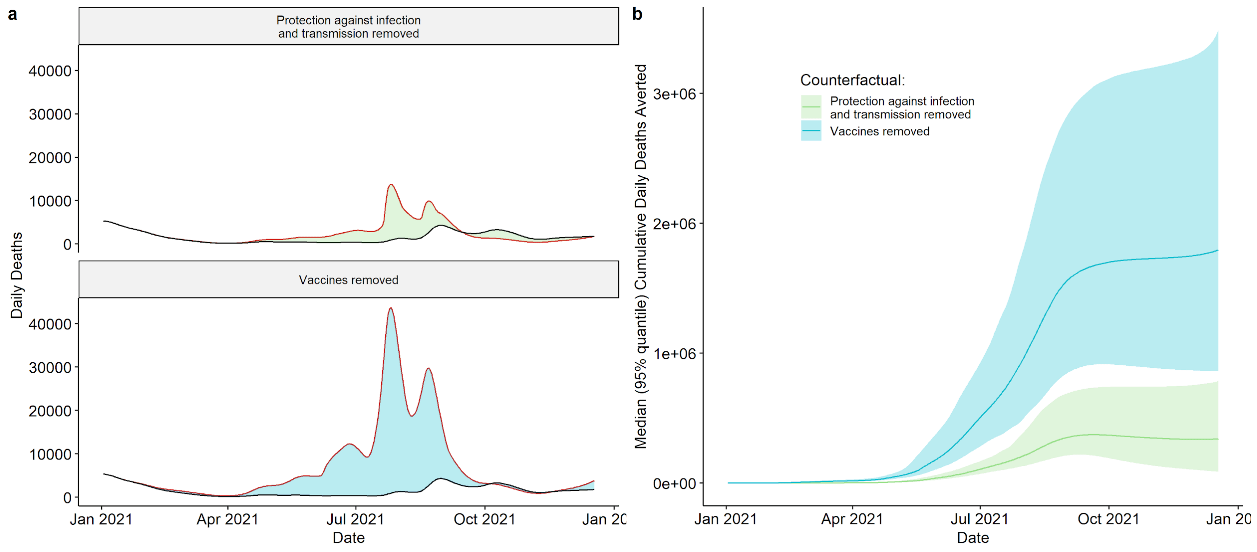

Supplementary Figure 1: Illustration of the process for estimating deaths averted by COVID-19 vaccinations: United States. a) The simulated COVID-19 deaths for a single draw from the estimated distribution of R_t. The black curves represent the simulation in the presence of vaccinations (the baseline simulation), i.e. the model fit to excess mortality, the red curves represent the counterfactual death curves with either no vaccinations (bottom panel) or vaccinations that only provide direct protection against severe disease (top panel). Shaded regions represent the deaths averted as calculated by the difference between the simulated number of deaths. **b)** The estimated cumulative number of deaths averted. The blue curve is the total number of deaths averted calculated from the baseline simulation and the counterfactual with no vaccines. The green curve is the number of deaths averted by the indirect effects of the vaccinations, calculated from the baseline simulation and the counterfactual with only direct vaccine effects. The median and quantiles are calculated from the deaths averted of 100 draws from the distribution of R_t.

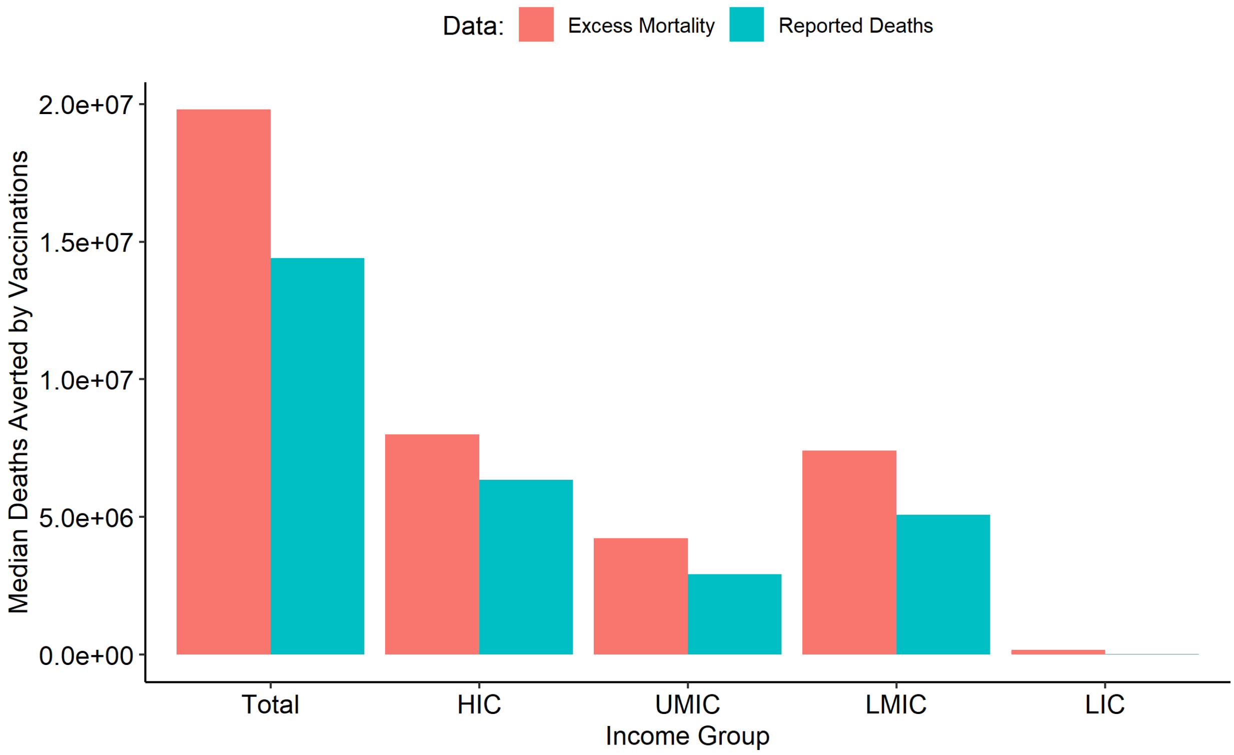

Supplementary Figure 2: Comparison of median global deaths averted by vaccinations by income group, across data type. Analyses were generated with countries fitted to either the excess mortality (orange bars) and reported deaths (blue bars). World Bank Income Group definitions: HIC High Income Countries, UMIC Upper-Middle Income Countries, LMIC Lower-Middle Income Countries, LIC Low Income Countries.

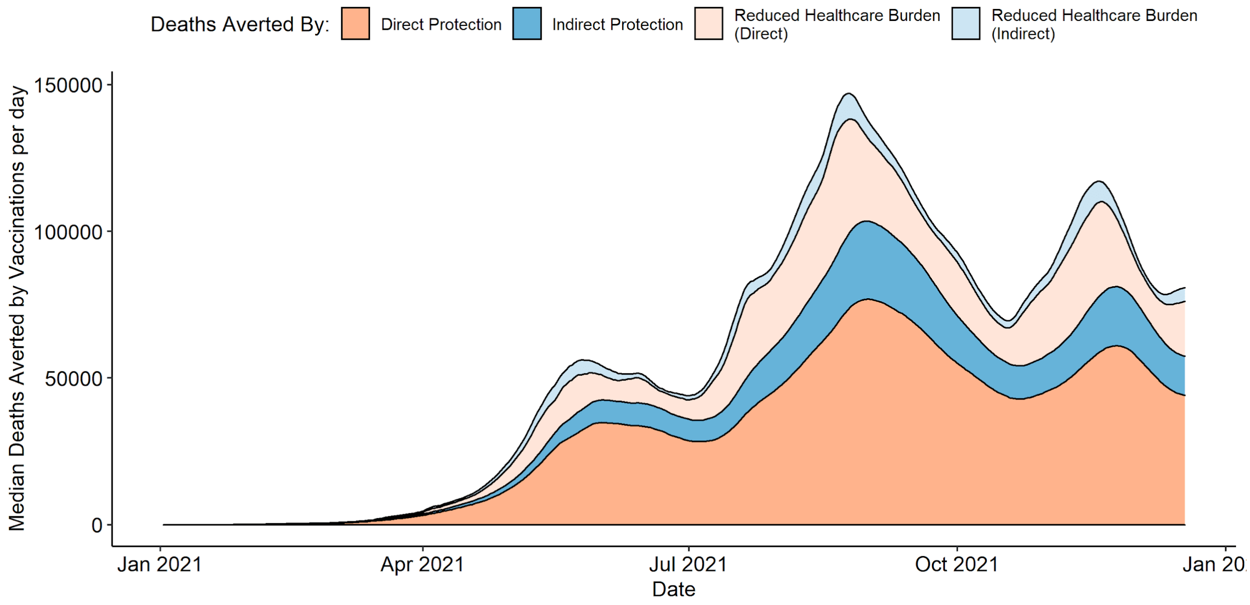

Supplementary Figure 3: Median global deaths averted by direct and indirect effects account for reductions in healthcare burden. By simulating counterfactuals without the effects of healthcare burden for the baseline simulation and counterfactuals without vaccination or protection and transmission we can further split deaths averted into those caused by the mechanisms:

- Direct Protection: reduced probability of being hospitalised once infected,
- Indirect Protection: reduced levels of COVID-19 from protection against infection and transmission,
- Reduced Healthcare Burden (Direct): increased access to healthcare due to a reduction in hospitalisation due to the direct protection of vaccination,
- Reduced Healthcare Burden (Indirect): increased access to healthcare due to a reduction in hospitalisation from the indirect protection of vaccination.

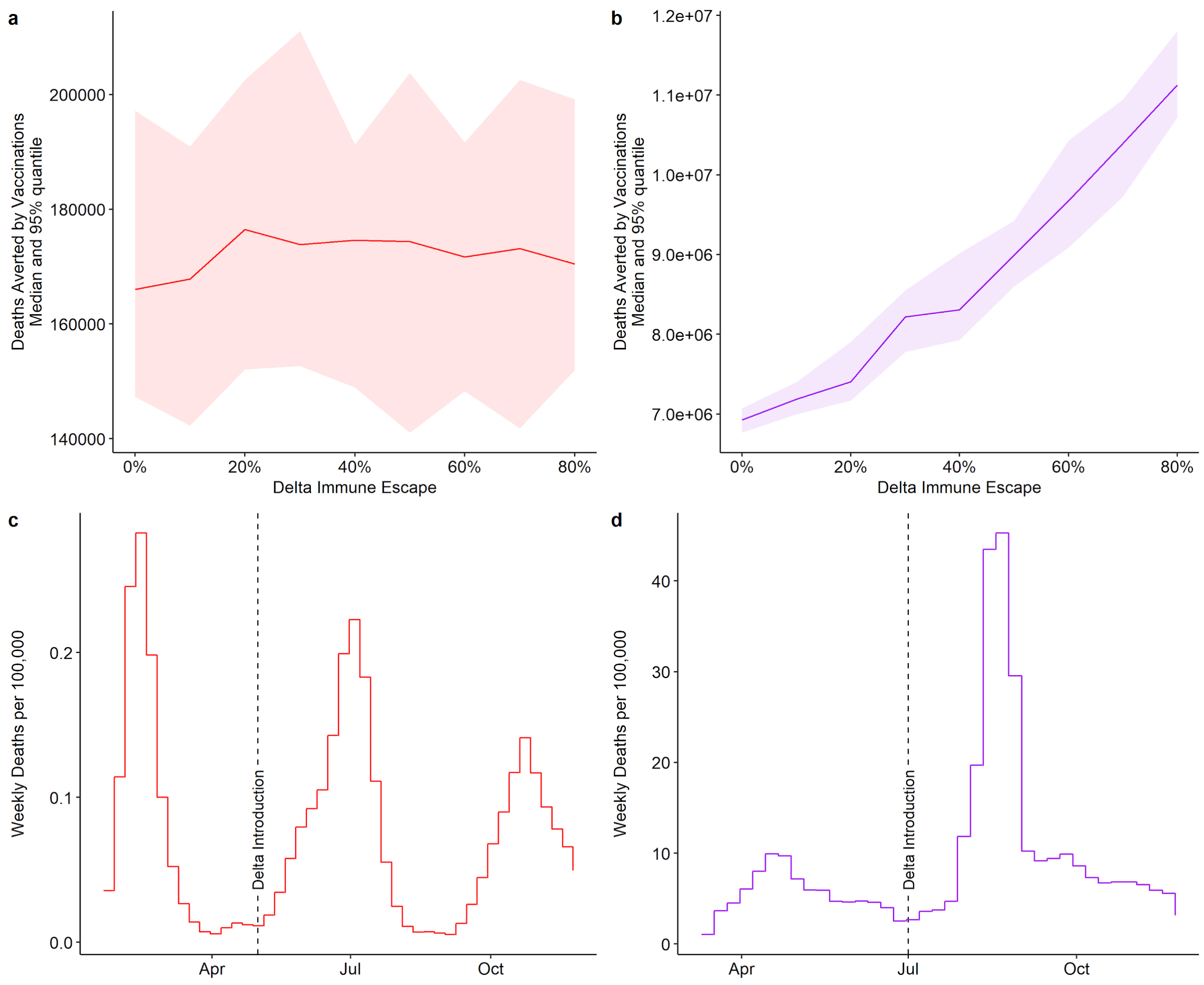

Supplementary Figure 4: Immune escape impact on deaths averted sensitivity analysis. a) Median (with 95% quantiles) deaths averted for the low deaths scenario (depicted in **c)**) with varying levels of immune escape for the Delta wave. **b)** Median (with 95% quantiles) deaths averted for the high deaths scenario (depicted in **d)**) with varying levels of immune escape for the Delta wave. Both plots generated by re-fitting the model to the same death curves with different levels of immune escape.

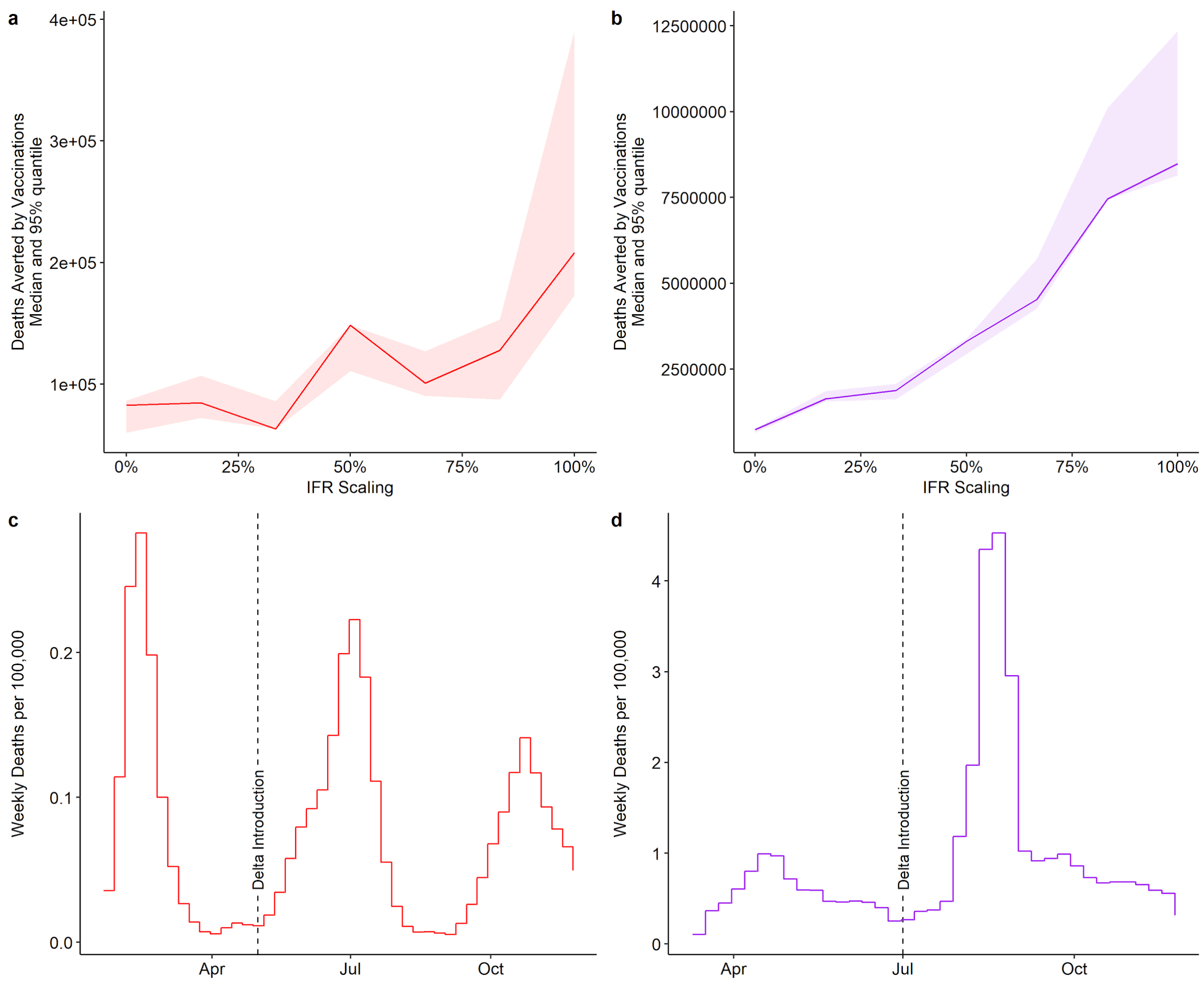

Supplementary Figure 5: Infection Fatality Ratio impact on deaths averted sensitivity analysis. a) Median (with 95% quantiles) deaths averted for the low deaths scenario (depicted in **c)**) with IFR scaled between 95% confidence levels as reported in Brazeau et al.[21] **b)** Median (with 95% quantiles) deaths averted for the high deaths scenario (depicted in **d)**) with IFR scaled between 95% confidence levels as reported in Brazeau et al.[21] Both plots generated by re-fitting the model to the same death curves with different probabilities of hospitalisation.

Supplementary Figure 6: Country level model fits to excess mortality

Model fits to the estimated excess mortality are presented for each country. Daily deaths are shown with estimated and reported weekly excess mortality shown as black lines. Red lines represent model estimates of daily deaths, dashed black lines represent the 95% quantiles of the estimated daily deaths. Plots of the cumulative estimated deaths are also shown, with the median and 95% quantiles of model estimated deaths in red and predicted and reported excess mortality as dashed black lines.

Plots are included at the end of the supplementary appendix for ease.

4. Supplementary References

1 Economist T, Solstad SU. The pandemic's true death toll. *Economist* 5 2021.

2 The Economist. Tracking covid-19 excess deaths across countries. 2020; published online July 15. https://www.economist.com/graphic-detail/2020/07/15/tracking-covid-19-excess-deaths-across-countries (accessed Aug 19, 2020).

3 Karlinsky A, Kobak D. Tracking excess mortality across countries during the COVID-19 pandemic with the World Mortality Dataset. *Elife* 2021; **10**. DOI:10.7554/eLife.69336.

4 Lunde BÅS, Kleppe TS. agtboost: Adaptive and Automatic Gradient Tree Boosting Computations. arXiv [stat.ML]. 2020; published online Aug 28. http://arxiv.org/abs/2008.12625.

5 Arel-Bundock V. World Development Indicators and Other World Bank Data [R package WDI version 2.7.4]. 2021; published online April 6. https://CRAN.R-project.org/package=WDI (accessed Jan 25, 2022).

6 Coppedge M, Gerring J, Knutsen CH, *et al.* V-Dem Dataset v11.1. 2021; published online April 22. DOI:10.2139/ssrn.3831905.

7 Boix C, Miller M, Rosato S. Boix-miller-Rosato dichotomous coding of democracy, 1800-2015. 2018. DOI:10.7910/DVN/FJLMKT.

8 House F. Freedom in the World 2020. 2020. https://apo.org.au/node/277656.

9 World Health Organization, Geneva. The 2018 update, Global Health Workforce Statistics. https://www.who.int/data/gho/data/themes/topics/health-workforce (accessed Feb 19, 2022).

10 Dong E, Du H, Gardner L. An interactive web-based dashboard to track COVID-19 in real time. *Lancet Infect Dis* 2020; **20**: 533–4.

11 Hasell J, Mathieu E, Beltekian D, *et al.* A cross-country database of COVID-19 testing. *Sci Data* 2020; **7**: 345.

12 Mathieu E, Ritchie H, Ortiz-Ospina E, *et al.* A global database of COVID-19 vaccinations. *Nat Hum Behav* 2021; **5**: 947–53.

13 Google. COVID-19 Community Mobility Reports. https://www.google.com/covid19/mobility/ (accessed Aug 19, 2020).

14 Hale T, Angrist N, Goldszmidt R, *et al.* A global panel database of pandemic policies (Oxford COVID-19 Government Response Tracker). *Nat Hum Behav* 2021; **5**: 529–38.

15 Arora RK, Joseph A, Van Wyk J, *et al.* SeroTracker: a global SARS-CoV-2 seroprevalence

dashboard. *Lancet Infect Dis* 2021; **21**: e75–6.

16 Number of deaths due to HIV/AIDS. https://www.who.int/data/gho/data/indicators/indicator-details/GHO/number-of-deaths-due-to-hiv-aids (accessed Feb 19, 2022).

17 Organization WH, Others. World malaria report 2020: 20 years of global progress and challenges. 2020. https://apps.who.int/iris/bitstream/handle/10665/337660/9789240015791-eng.pdf.

18 Organization WH, Others. Global tuberculosis report 2020: executive summary. 2020. https://apps.who.int/iris/bitstream/handle/10665/337538/9789240016941-ara.pdf.

19 Vardell E. Global Health Observatory Data Repository. *Med Ref Serv Q* 2020; **39**: 67–74.

20 World Population Prospects - Population Division - United Nations. https://population.un.org/wpp/ (accessed Aug 22, 2020).

21 Brazeau N, Verity R, Jenks S, *et al.* Report 34: COVID-19 infection fatality ratio: estimates from seroprevalence. DOI:10.25561/83545.

22 Richard A. Becker, Allan R. Wilks, Ray Brownrigg. maps: Draw Geographical Maps. 2021 https://cran.r-project.org/web/packages/maps/index.html.

23 Gallup JL, Mellinger AD, Sachs JD. Geography Datasets. 2010. DOI:10.7910/DVN/SPHS5E.

24 Mayer T, Zignago S. Notes on CEPII's Distances Measures: The GeoDist Database. 2011; published online Dec 1. DOI:10.2139/ssrn.1994531.

25 Geneva: World Health Organization. WHO COVID-19 Dashboard. https://covid19.who.int/explorer (accessed Feb 19, 2022).

26 Walker PGT, Whittaker C, Watson OJ, *et al.* The impact of COVID-19 and strategies for mitigation and suppression in low- and middle-income countries. *Science* 2020; **369**: 413–22.

27 Hogan AB, Winskill P, Watson OJ, *et al.* Within-country age-based prioritisation, global allocation, and public health impact of a vaccine against SARS-CoV-2: A mathematical modelling analysis. *Vaccine* 2021; **39**: 2995–3006.

28 Watson OJ, Alhaffar M, Mehchy Z, *et al.* Leveraging community mortality indicators to infer COVID-19 mortality and transmission dynamics in Damascus, Syria. *Nat Commun* 2021; **12**: 2394.

29 Eyre DW, Taylor D, Purver M, *et al.* Effect of Covid-19 Vaccination on Transmission of Alpha and Delta Variants. *N Engl J Med* 2022; published online Jan 5. DOI:10.1056/NEJMoa2116597.

30 Unicef COVID-19 Vaccine Market Dashboard. https://www.unicef.org/supply/covid-19-vaccine-market-dashboard (accessed March 28,

2022).

31 Imai N, Hogan AB, Williams L, *et al.* Interpreting estimates of coronavirus disease 2019 (COVID-19) vaccine efficacy and effectiveness to inform simulation studies of vaccine impact: a systematic review. *Wellcome Open Res* 2021; **6**: 185.

32 Khare S, Gurry C, Freitas L, *et al.* GISAID's Role in Pandemic Response. *China CDC Wkly* 2021; **3**: 1049–51.

33 Hodcroft. EB. CoVariants: SARS-CoV-2 Mutations and Variants of Interest. 2021. https://covariants.org/ (accessed Feb 19, 2022).

34 Twohig KA, Nyberg T, Zaidi A, *et al.* Hospital admission and emergency care attendance risk for SARS-CoV-2 delta (B.1.617.2) compared with alpha (B.1.1.7) variants of concern: a cohort study. *Lancet Infect Dis* 2022; **22**: 35–42.

35 Salje H, Tran Kiem C, Lefrancq N, *et al.* Estimating the burden of SARS-CoV-2 in France. *Science* 2020; **369**: 208–11.

36 Seppälä E, Veneti L, Starrfelt J, *et al.* Vaccine effectiveness against infection with the Delta (B.1.617.2) variant, Norway, April to August 2021. Eurosurveillance. 2021; **26**. DOI:10.2807/1560-7917.es.2021.26.35.2100793.

37 Chadeau-Hyam M, Wang H, Eales O, *et al.* REACT-1 study round 14: High and increasing prevalence of SARS-CoV-2 infection among school-aged children during September 2021 and vaccine effectiveness against infection in England. bioRxiv. 2021; published online Oct 22. DOI:10.1101/2021.10.14.21264965.

38 Stowe J, Andrews N, Gower C, *et al.* Effectiveness of COVID-19 vaccines against hospital admission with the Delta (B.1.617.2) variant. *Public Health England Publishing* 2021. https://khub.net/web/phe-national/public-library/-/document_library/v2WsRK3ZlEig/view/479607266 (accessed Feb 19, 2022).

39 Dhar MS, Marwal R, Vs R, *et al.* Genomic characterization and epidemiology of an emerging SARS-CoV-2 variant in Delhi, India. *Science* 2021; **374**: 995–9.

40 Imperial College COVID-19 LMIC Reports. Version 5. MRC Centre for Global Infectious Disease Analysis, Imperial College London. https://mrc-ide.github.io/global-lmic-reports/ (accessed Aug 19, 2020).

41 Verity R, Winskill P. drjacoby: Flexible Markov Chain Monte Carlo via Reparameterization. https://mrc-ide.github.io/drjacoby/index.html (accessed April 26, 2022).

42 Worldometers. Worldometers--real time world statistics. 2015.

43 Toledo-Romani ME, Garcia-Carmenate M, Silva CV, *et al.* Efficacy and safety of SOBERANA 02, a COVID-19 conjugate vaccine in heterologous three-dose combination. bioRxiv. 2021; published online Nov 1. DOI:10.1101/2021.10.31.21265703.

44 Novavax Confirms High Levels of Efficacy Against Original and Variant COVID-19 Strains in United Kingdom and South Africa Trials. Novavax Investor Relations. 2021; published

online March 11. https://ir.novavax.com/2021-03-11-Novavax-Confirms-High-Levels-of-Efficacy-Against-Original-and-Variant-COVID-19-Strains-in-United-Kingdom-and-South-Africa-Trials (accessed April 26, 2022).

45 Kang M, Yi Y, Li Y, *et al.* Effectiveness of Inactivated COVID-19 Vaccines Against Illness Caused by the B.1.617.2 (Delta) Variant During an Outbreak in Guangdong, China : A Cohort Study. *Ann Intern Med* 2022; **175**: 533–40.

46 Martínez-Baz I, Trobajo-Sanmartín C, Miqueleiz A, *et al.* Product-specific COVID-19 vaccine effectiveness against secondary infection in close contacts, Navarre, Spain, April to August 2021. *Euro Surveill* 2021; **26**. DOI:10.2807/1560-7917.ES.2021.26.39.2100894.

47 Lopez Bernal J, Andrews N, Gower C, *et al.* Effectiveness of Covid-19 Vaccines against the B.1.617.2 (Delta) Variant. *N Engl J Med* 2021; **385**: 585–94.

48 Barnsley G, Watson OJ. mrc-ide/covid-vaccine-impact-orderly. v1.0.1. 2022 DOI:10.5281/zenodo.6539545.

Afghanistan

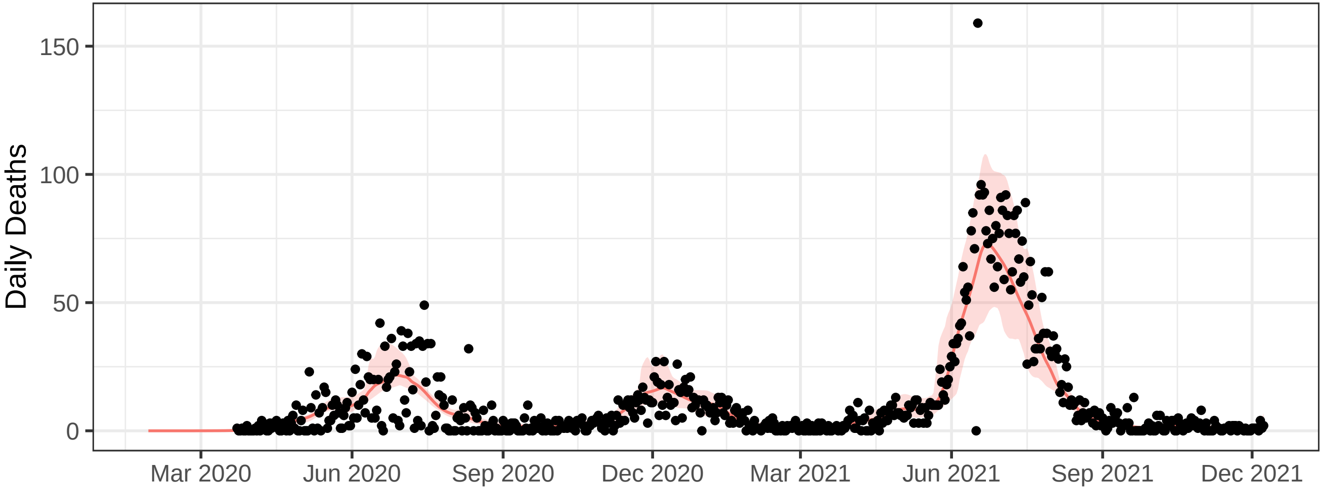

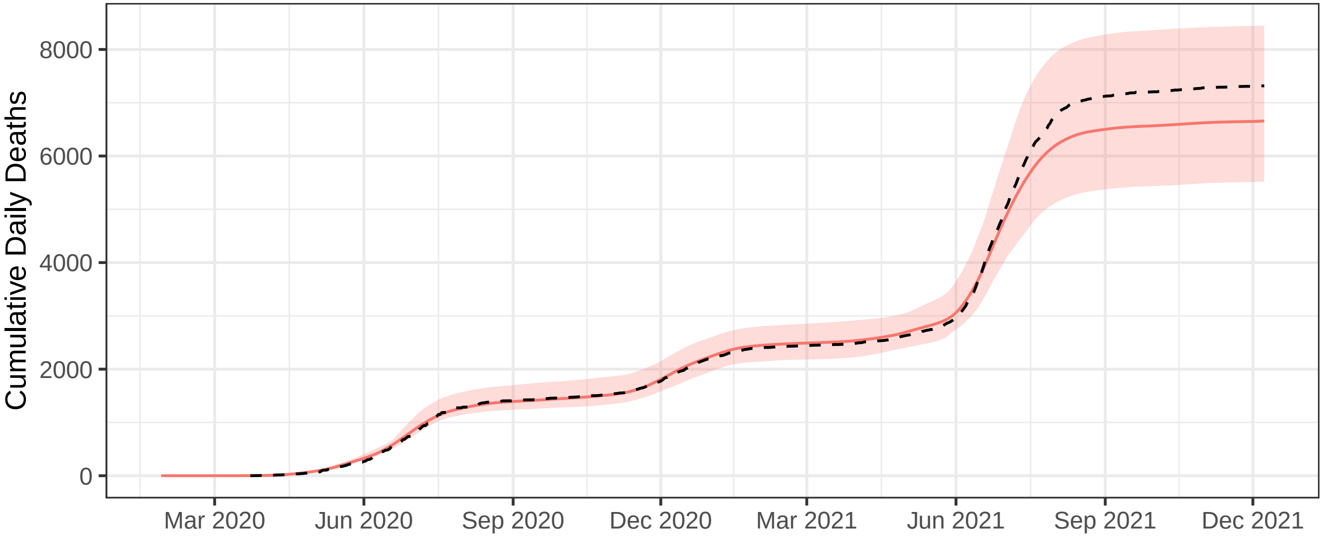

Daily deaths are included with reported COVID−19 deaths shown as points. The red line represents median model estimates of daily deaths, and the shaded region represents the 95% quantiles of the model estimated deaths. Plots of the cumulative estimated deaths are also shown, with the cumulative number of reported deaths represented by a dashed black line.

Angola

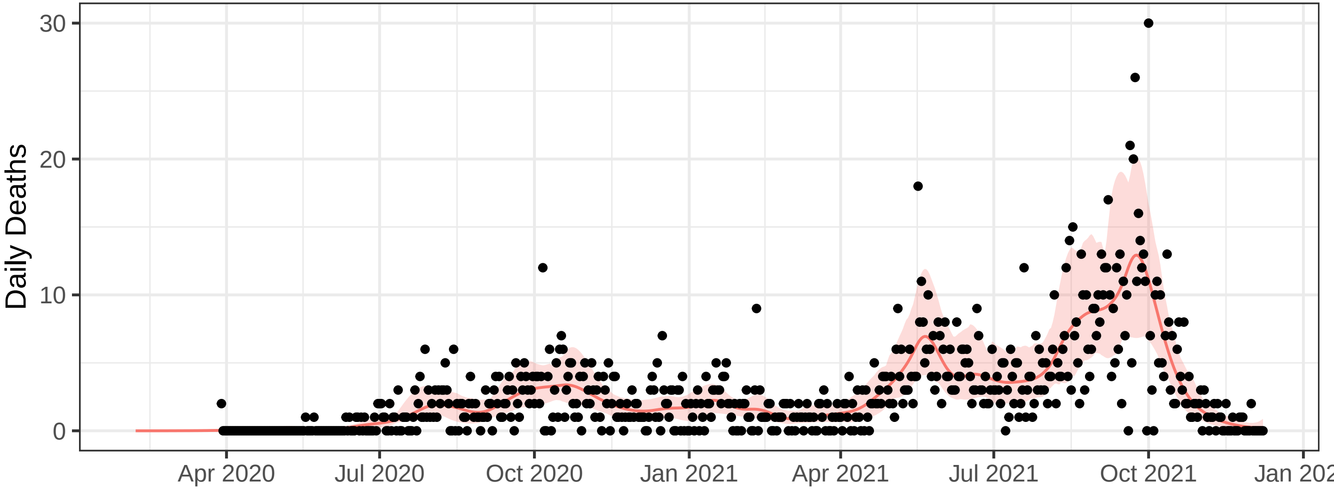

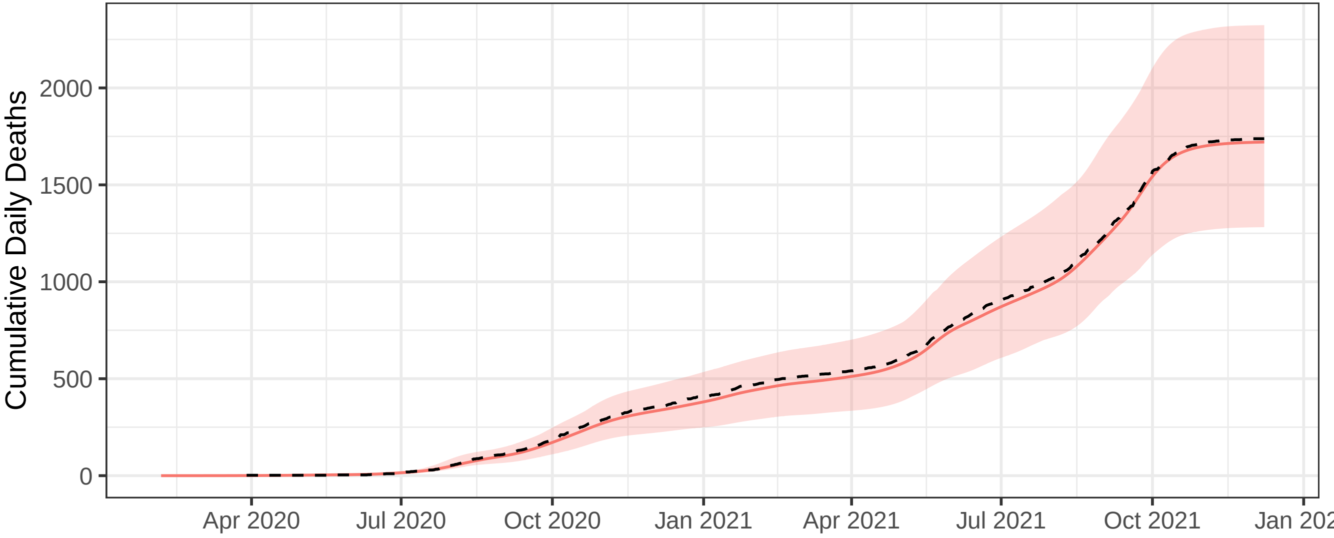

Daily deaths are included with reported COVID−19 deaths shown as points. The red line represents median model estimates of daily deaths, and the shaded region represents the 95% quantiles of the model estimated deaths. Plots of the cumulative estimated deaths are also shown, with the cumulative number of reported deaths represented by a dashed black line.

Albania

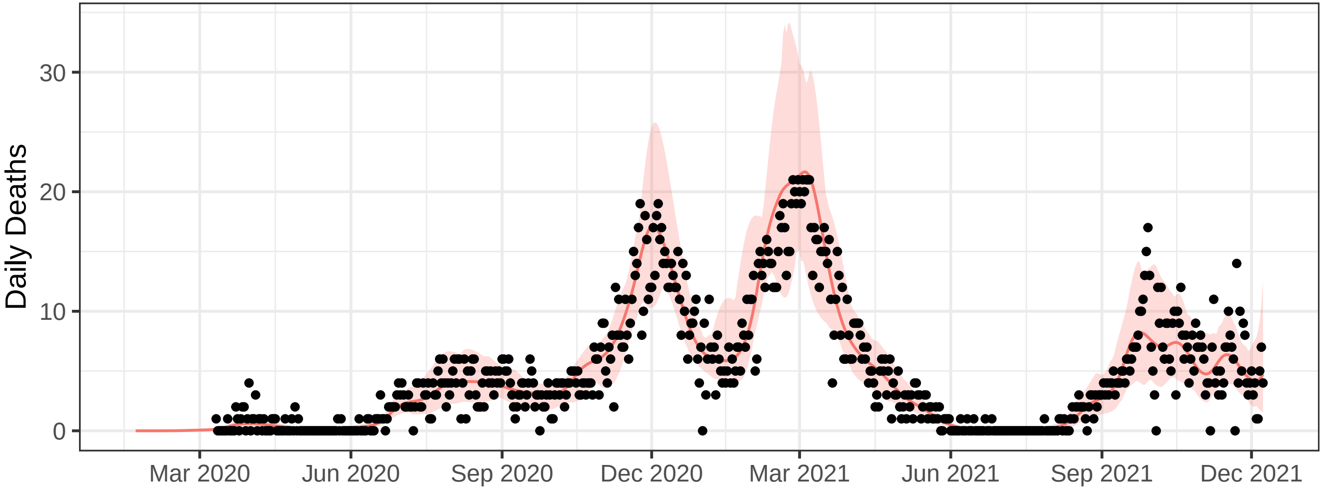

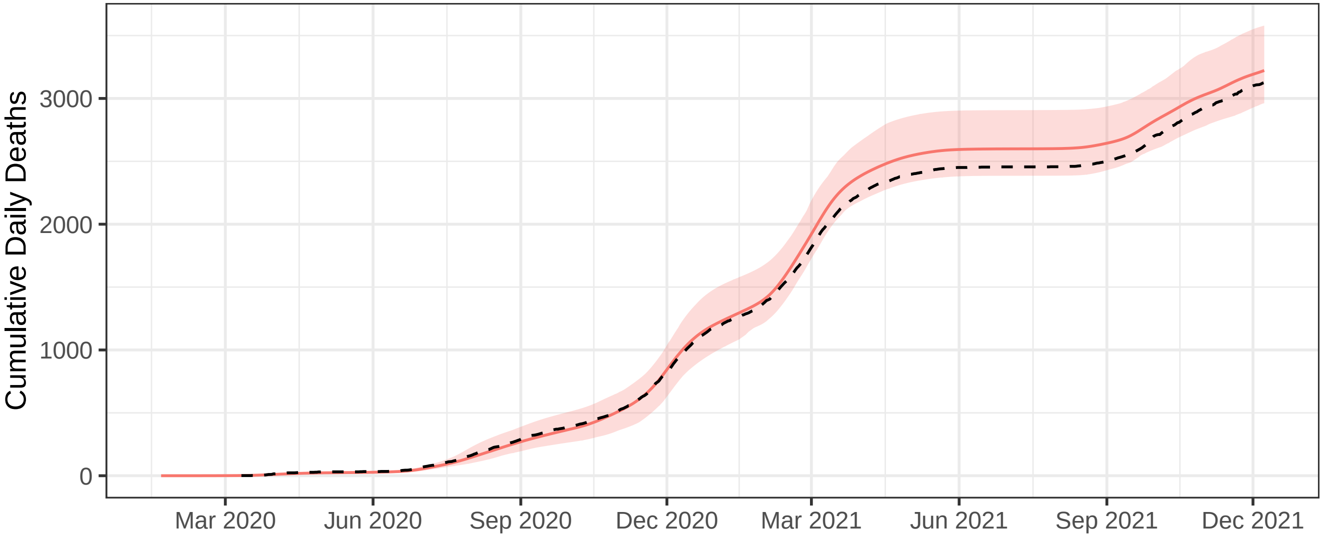

Daily deaths are included with reported COVID−19 deaths shown as points. The red line represents median model estimates of daily deaths, and the shaded region represents the 95% quantiles of the model estimated deaths. Plots of the cumulative estimated deaths are also shown, with the cumulative number of reported deaths represented by a dashed black line.

United Arab Emirates

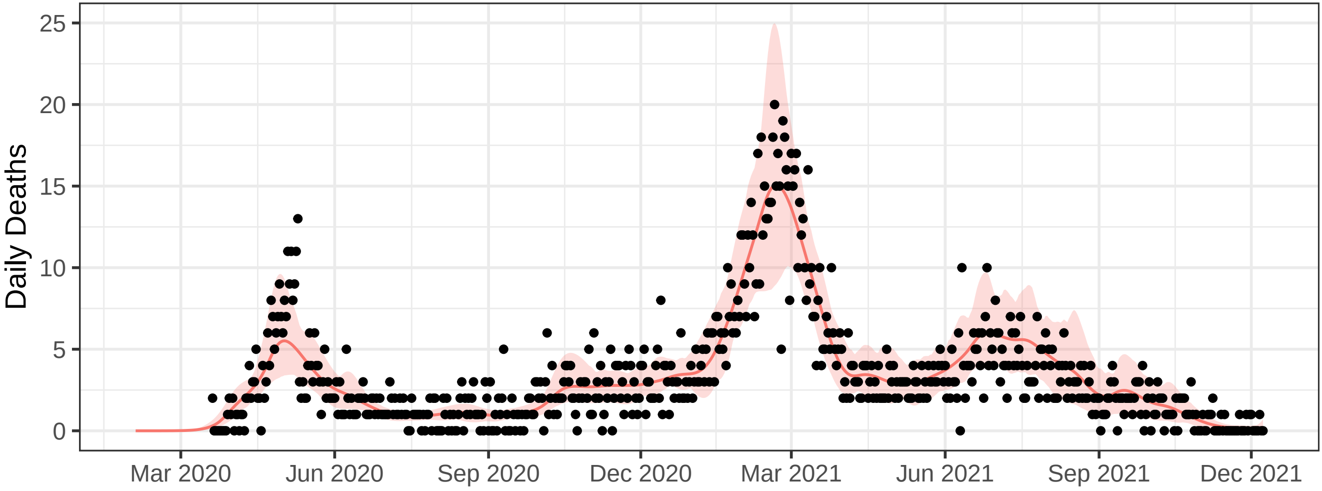

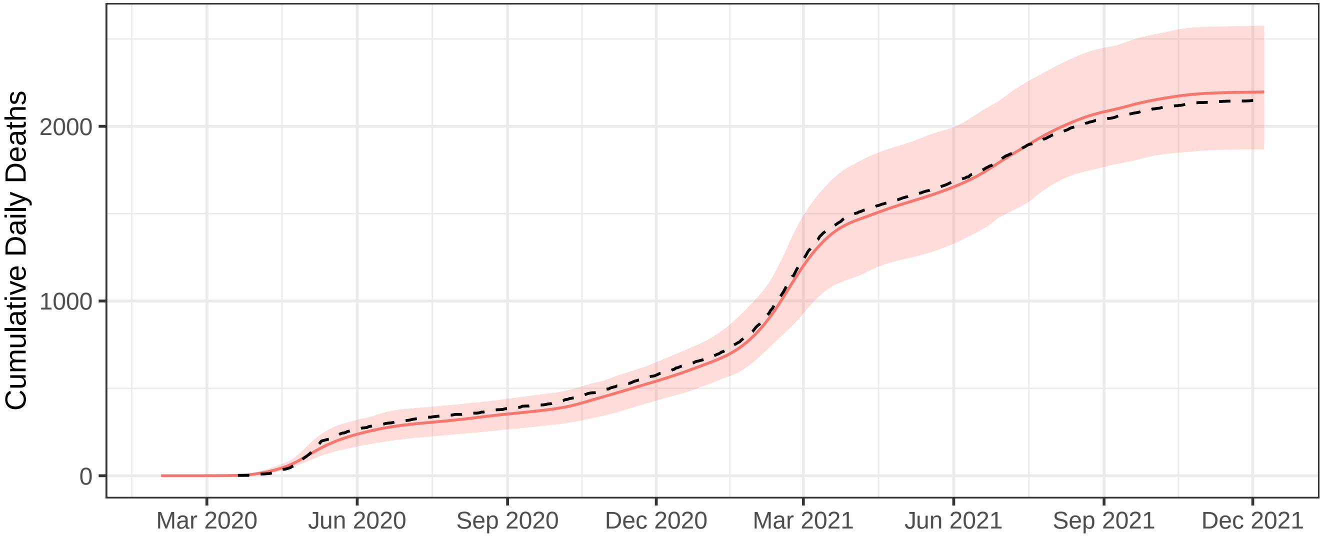

Daily deaths are included with reported COVID−19 deaths shown as points. The red line represents median model estimates of daily deaths, and the shaded region represents the 95% quantiles of the model estimated deaths. Plots of the cumulative estimated deaths are also shown, with the cumulative number of reported deaths represented by a dashed black line.

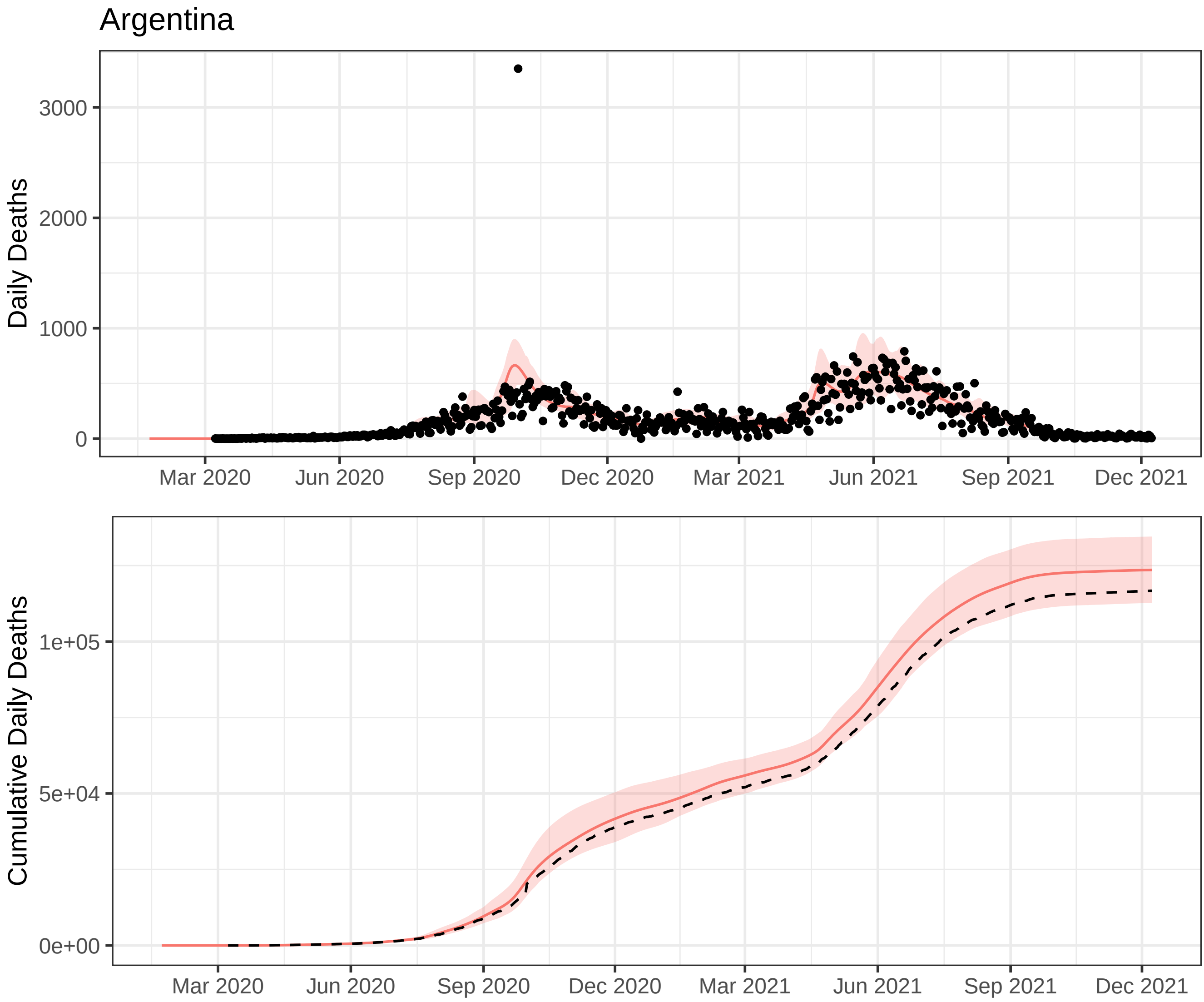

Daily deaths are included with reported COVID−19 deaths shown as points. The red line represents median model estimates of daily deaths, and the shaded region represents the 95% quantiles of the model estimated deaths. Plots of the cumulative estimated deaths are also shown, with the cumulative number of reported deaths represented by a dashed black line.

Armenia

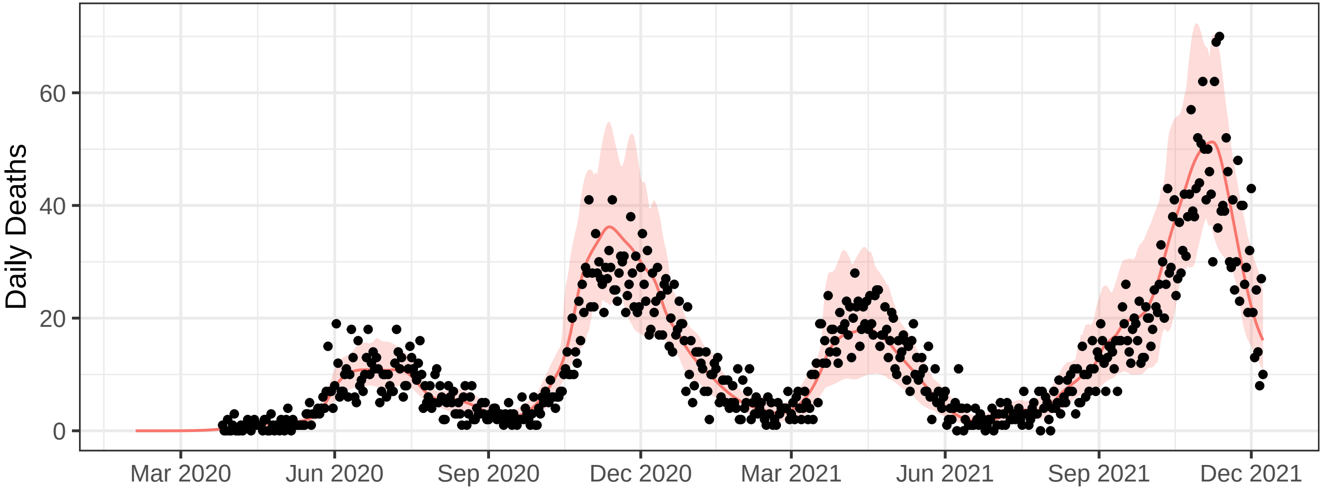

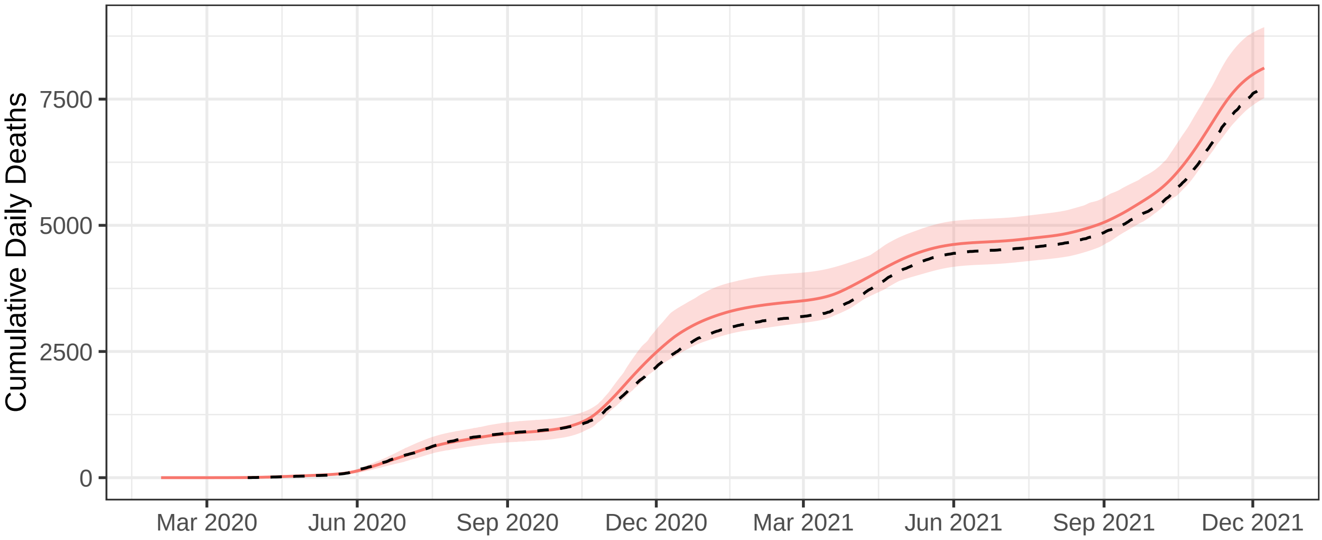

Daily deaths are included with reported COVID−19 deaths shown as points. The red line represents median model estimates of daily deaths, and the shaded region represents the 95% quantiles of the model estimated deaths. Plots of the cumulative estimated deaths are also shown, with the cumulative number of reported deaths represented by a dashed black line.

Antigua and Barbuda

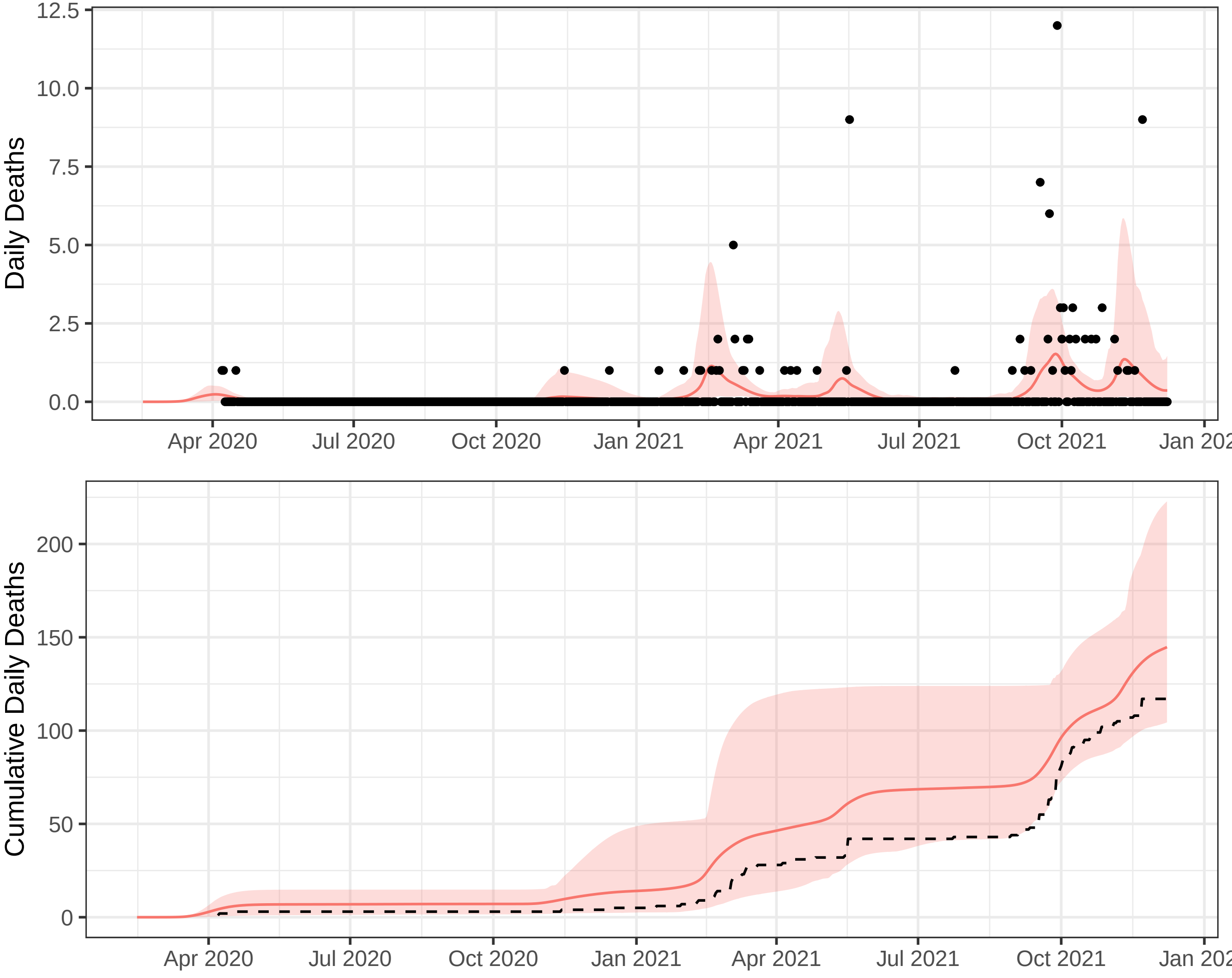

Daily deaths are included with reported COVID−19 deaths shown as points. The red line represents median model estimates of daily deaths, and the shaded region represents the 95% quantiles of the model estimated deaths. Plots of the cumulative estimated deaths are also shown, with the cumulative number of reported deaths represented by a dashed black line.

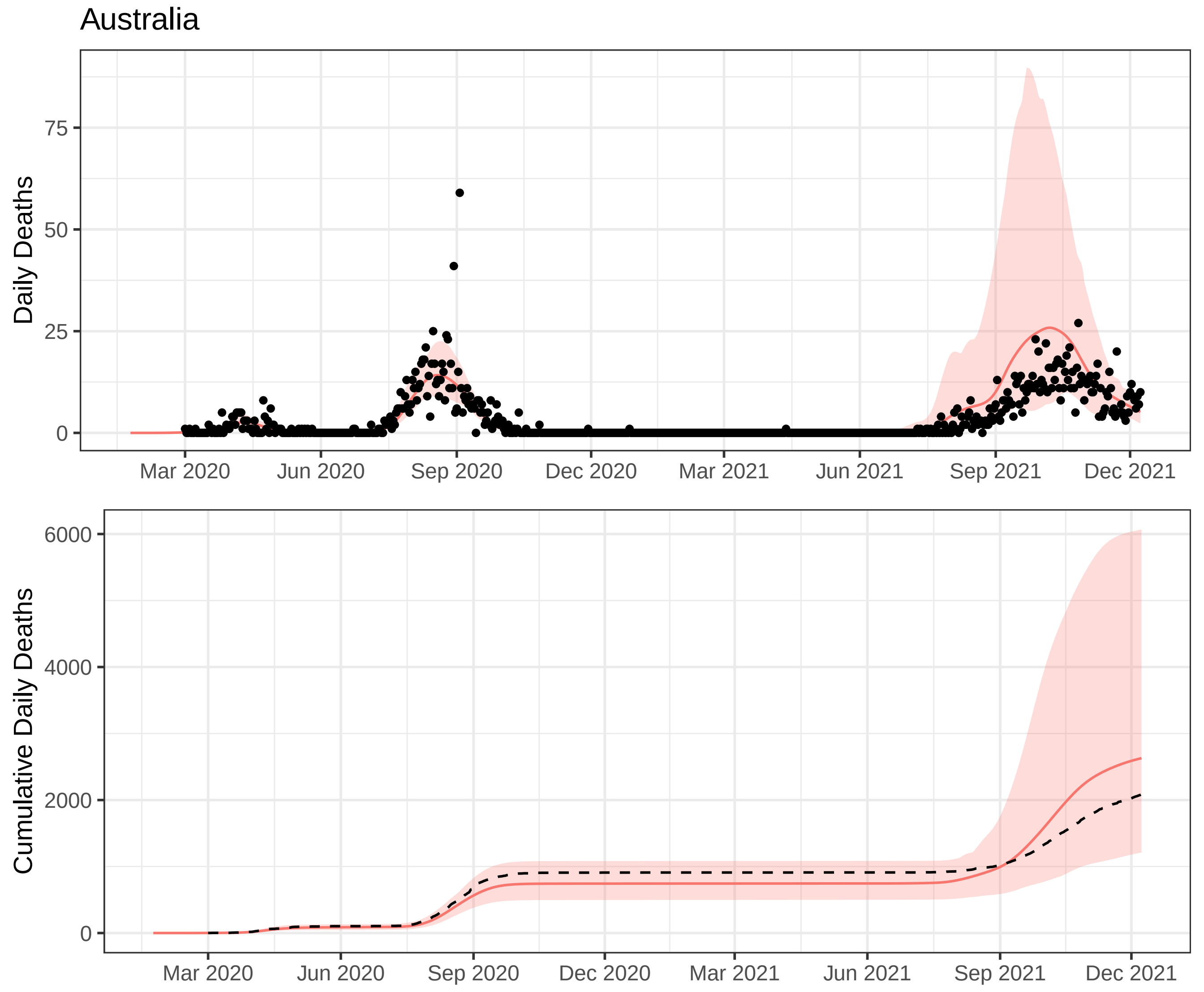

Daily deaths are included with reported COVID−19 deaths shown as points. The red line represents median model estimates of daily deaths, and the shaded region represents the 95% quantiles of the model estimated deaths. Plots of the cumulative estimated deaths are also shown, with the cumulative number of reported deaths represented by a dashed black line.

Austria

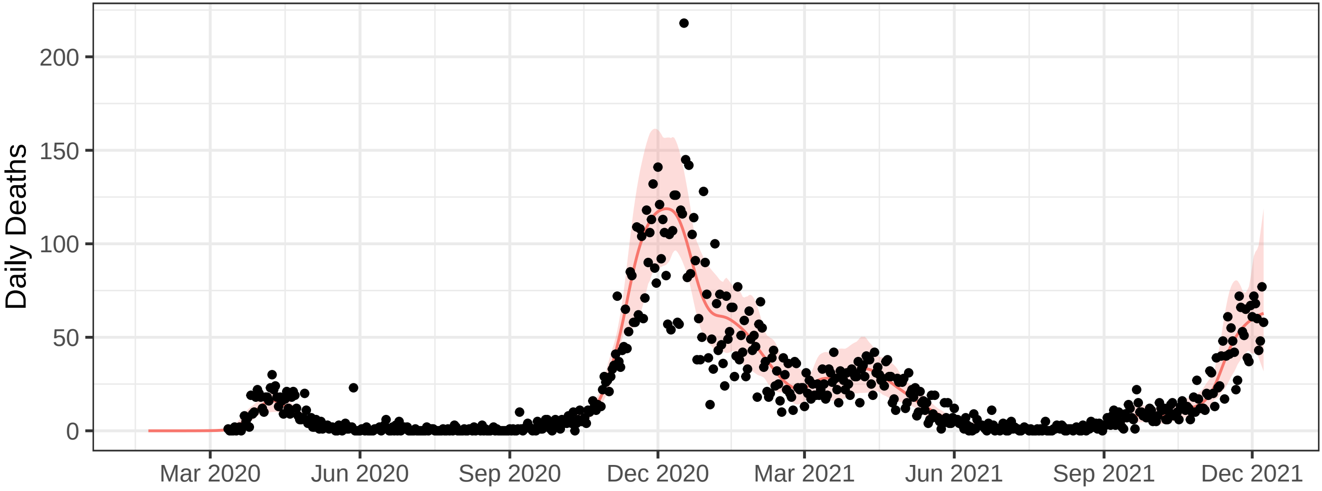

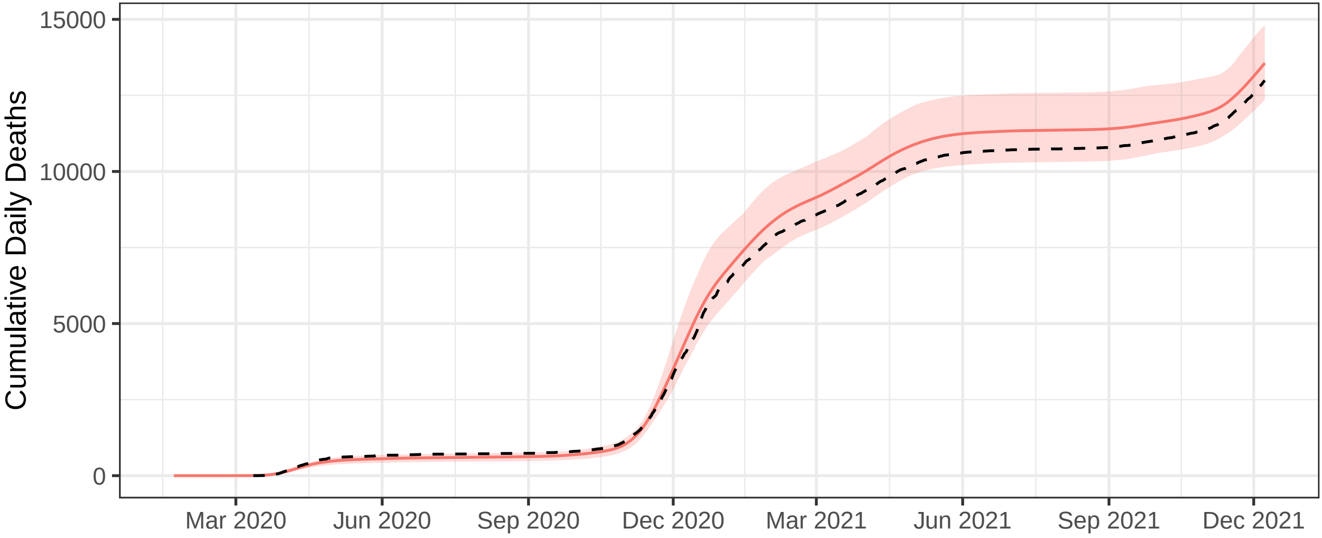

Daily deaths are included with reported COVID−19 deaths shown as points. The red line represents median model estimates of daily deaths, and the shaded region represents the 95% quantiles of the model estimated deaths. Plots of the cumulative estimated deaths are also shown, with the cumulative number of reported deaths represented by a dashed black line.

Azerbaijan

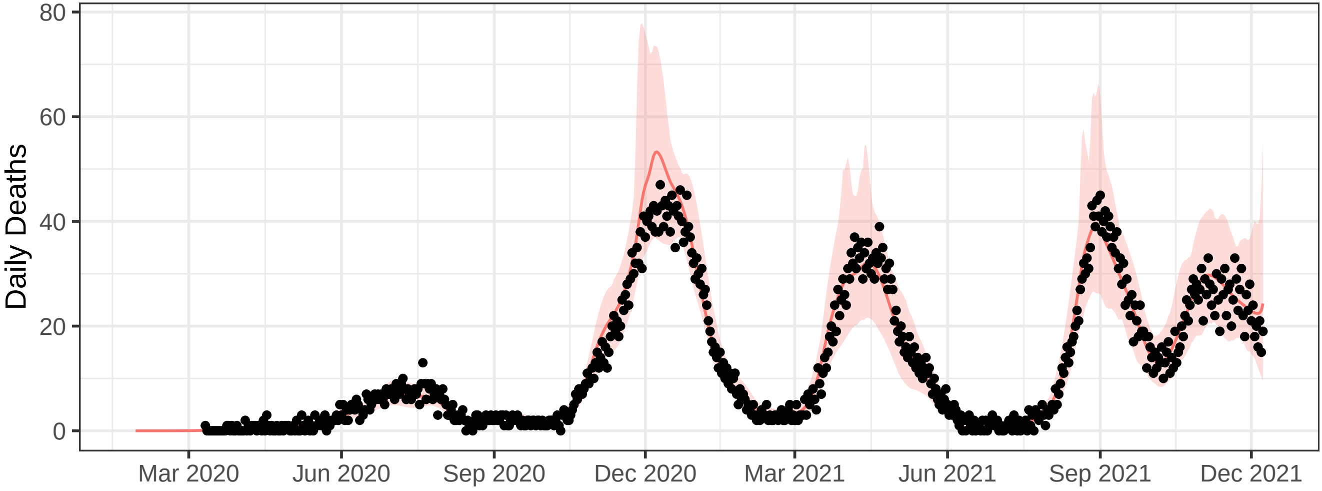

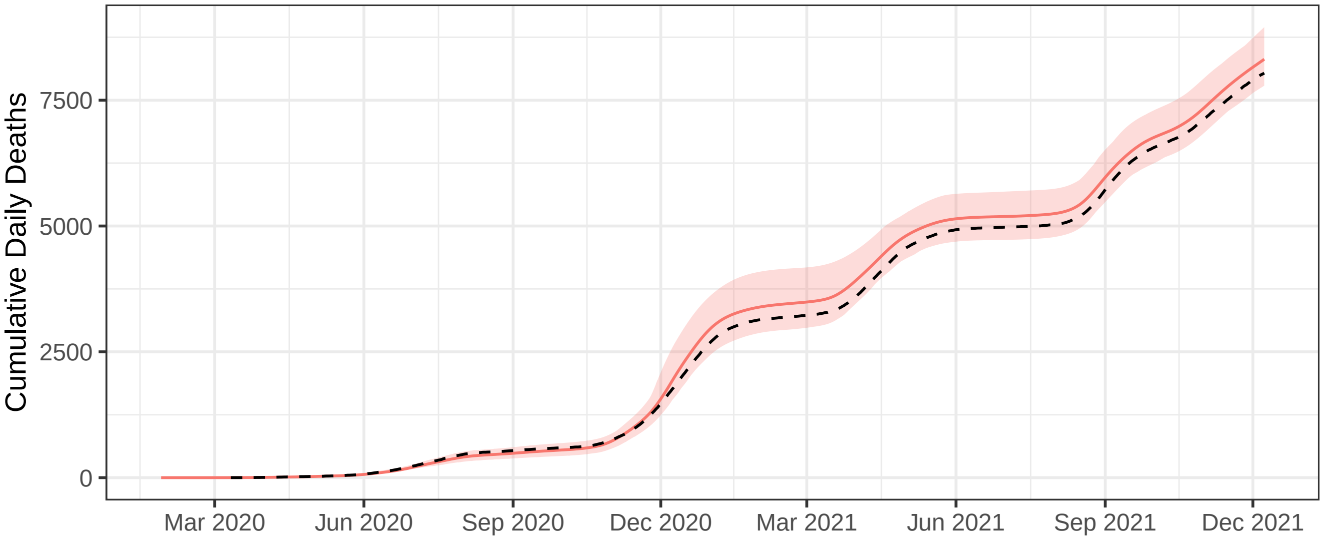

Daily deaths are included with reported COVID−19 deaths shown as points. The red line represents median model estimates of daily deaths, and the shaded region represents the 95% quantiles of the model estimated deaths. Plots of the cumulative estimated deaths are also shown, with the cumulative number of reported deaths represented by a dashed black line.

Burundi

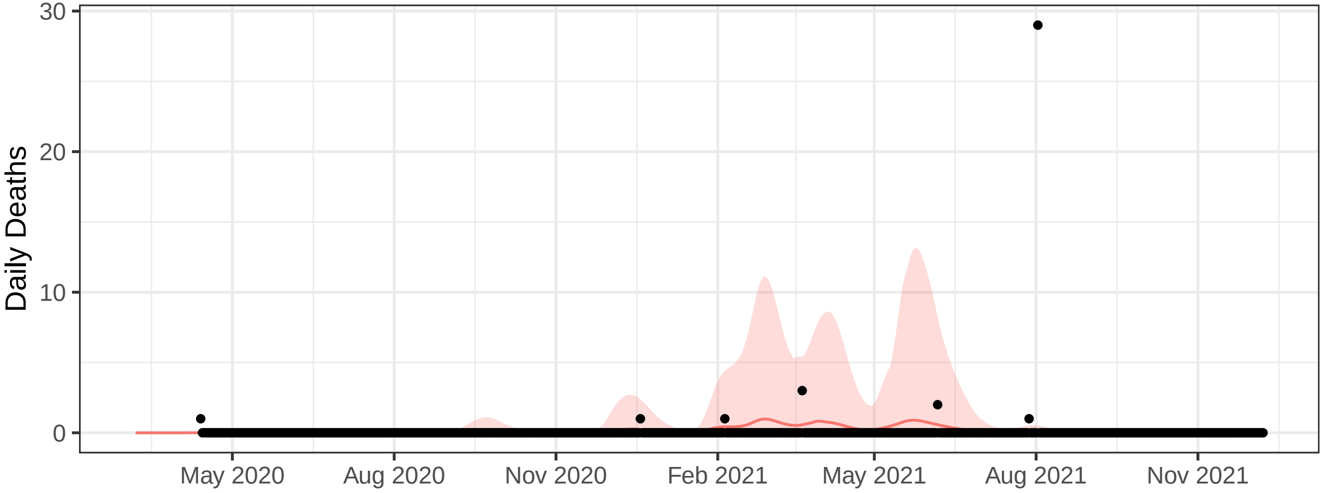

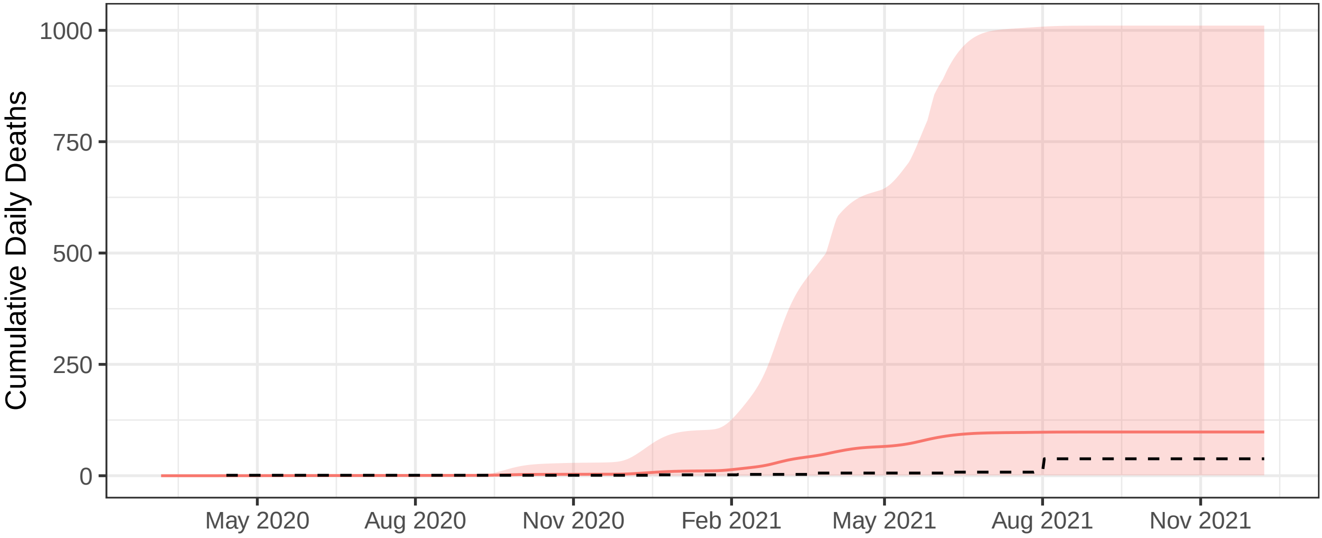

Daily deaths are included with reported COVID−19 deaths shown as points. The red line represents median model estimates of daily deaths, and the shaded region represents the 95% quantiles of the model estimated deaths. Plots of the cumulative estimated deaths are also shown, with the cumulative number of reported deaths represented by a dashed black line.

Belgium

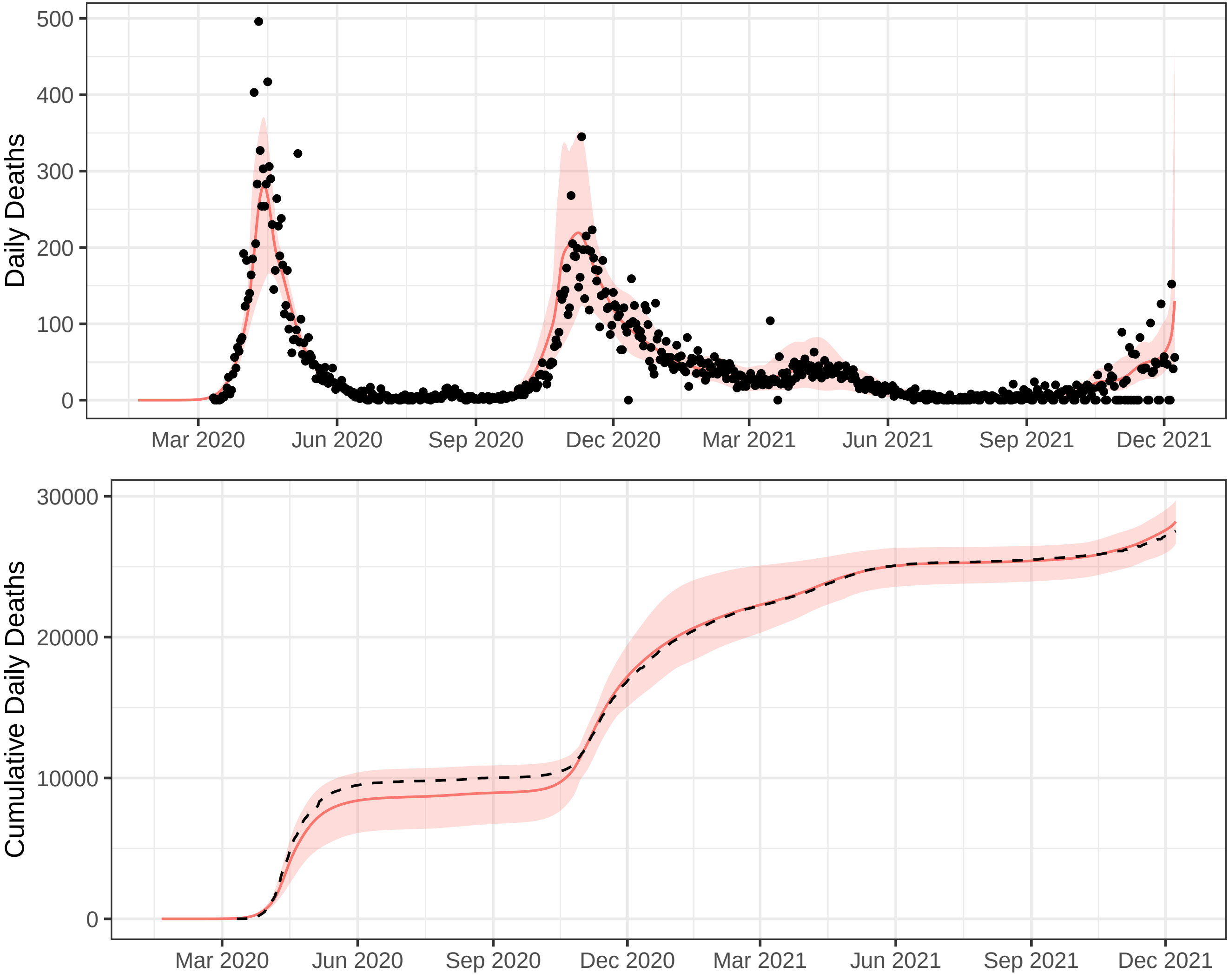

Daily deaths are included with reported COVID−19 deaths shown as points. The red line represents median model estimates of daily deaths, and the shaded region represents the 95% quantiles of the model estimated deaths. Plots of the cumulative estimated deaths are also shown, with the cumulative number of reported deaths represented by a dashed black line.

Benin

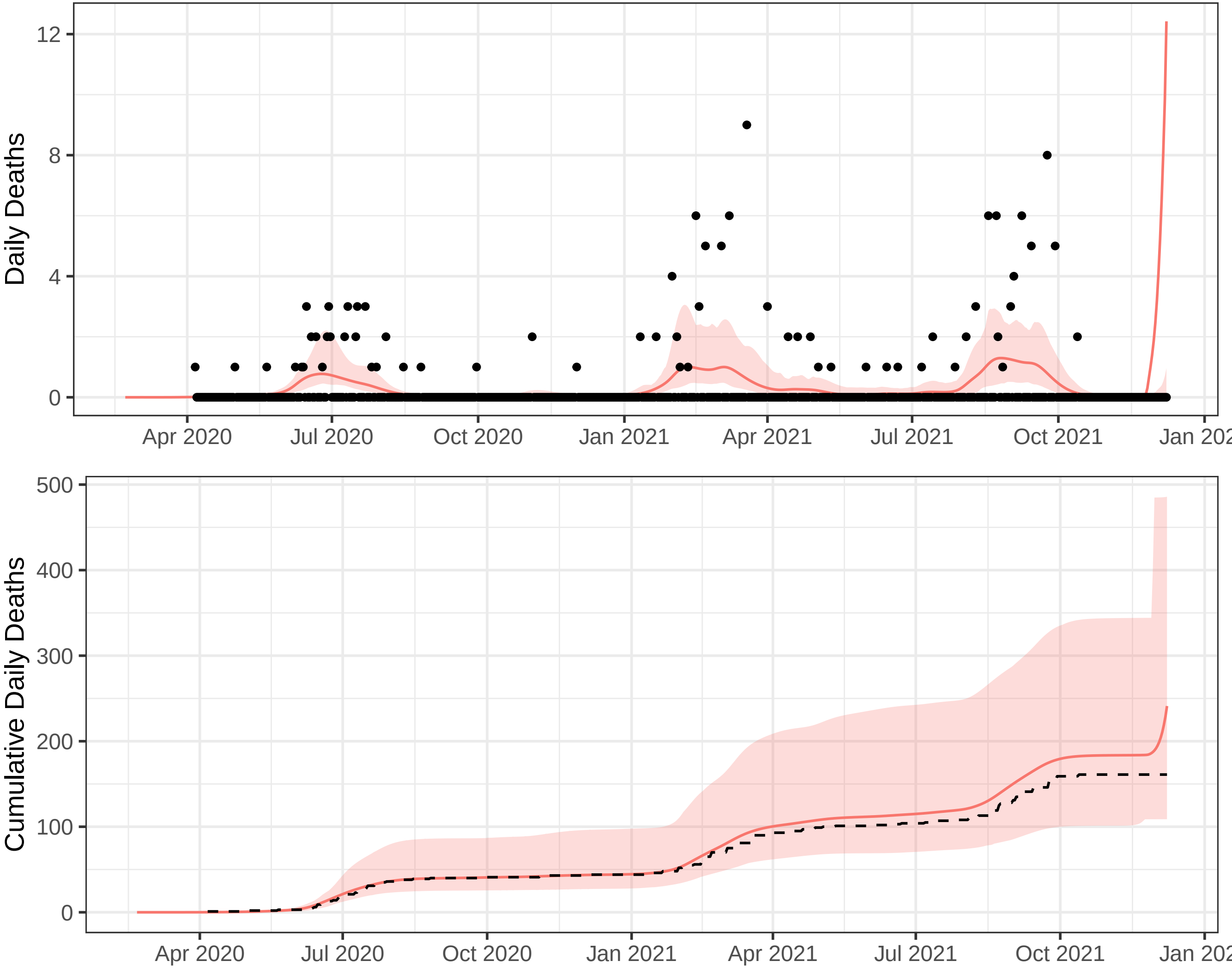

Daily deaths are included with reported COVID−19 deaths shown as points. The red line represents median model estimates of daily deaths, and the shaded region represents the 95% quantiles of the model estimated deaths. Plots of the cumulative estimated deaths are also shown, with the cumulative number of reported deaths represented by a dashed black line.

Burkina Faso

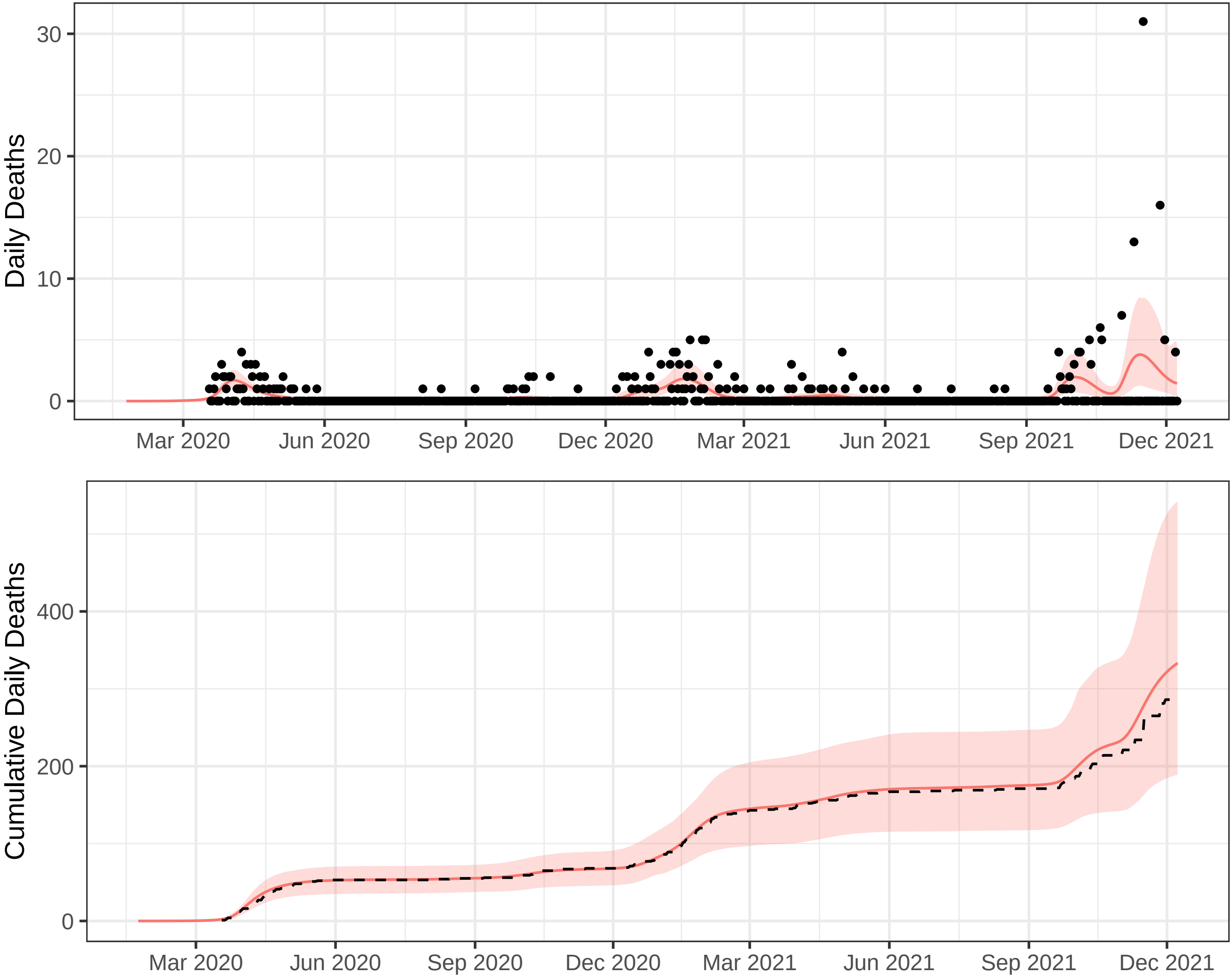

Daily deaths are included with reported COVID−19 deaths shown as points. The red line represents median model estimates of daily deaths, and the shaded region represents the 95% quantiles of the model estimated deaths. Plots of the cumulative estimated deaths are also shown, with the cumulative number of reported deaths represented by a dashed black line.

Bangladesh

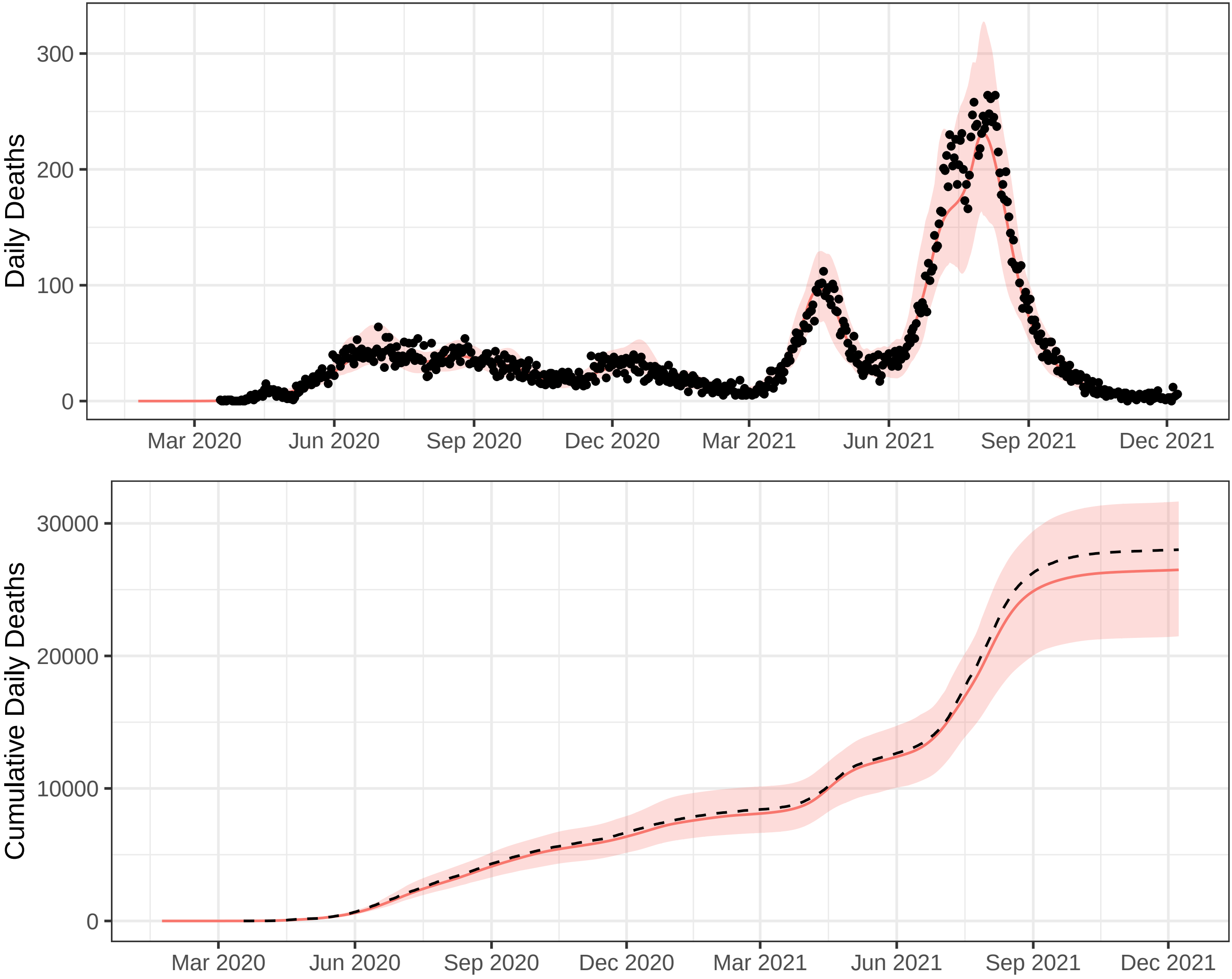

Daily deaths are included with reported COVID−19 deaths shown as points. The red line represents median model estimates of daily deaths, and the shaded region represents the 95% quantiles of the model estimated deaths. Plots of the cumulative estimated deaths are also shown, with the cumulative number of reported deaths represented by a dashed black line.

Bulgaria

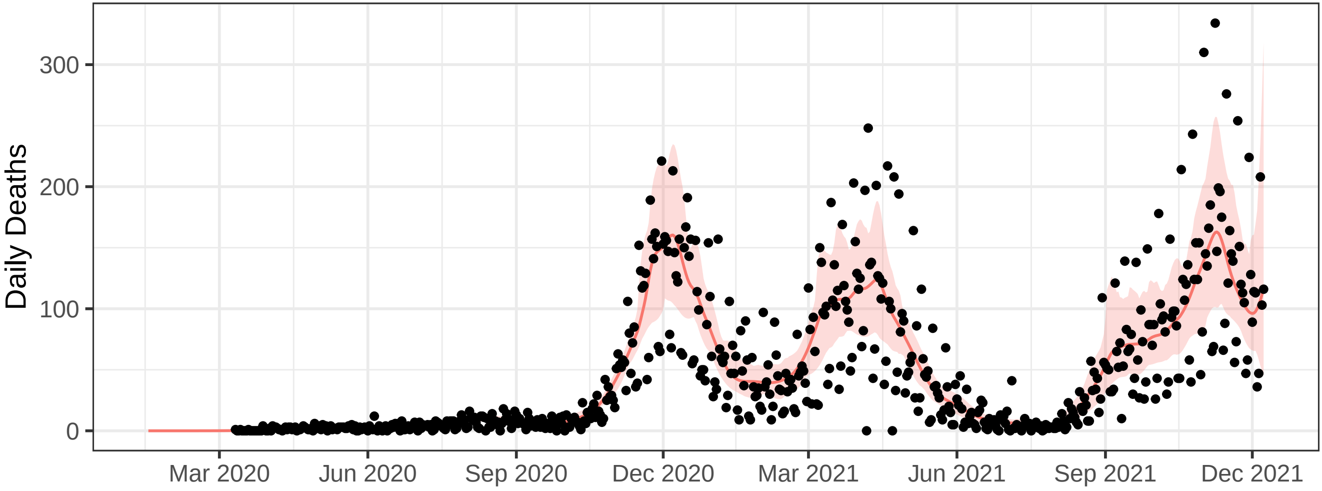

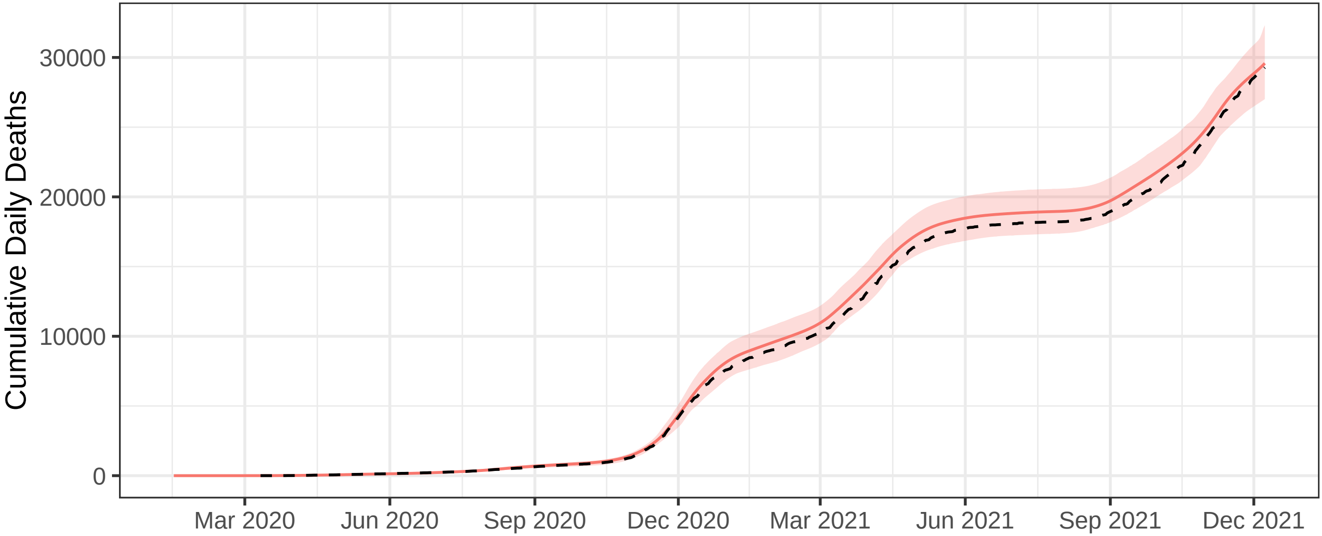

Daily deaths are included with reported COVID−19 deaths shown as points. The red line represents median model estimates of daily deaths, and the shaded region represents the 95% quantiles of the model estimated deaths. Plots of the cumulative estimated deaths are also shown, with the cumulative number of reported deaths represented by a dashed black line.

Bahrain

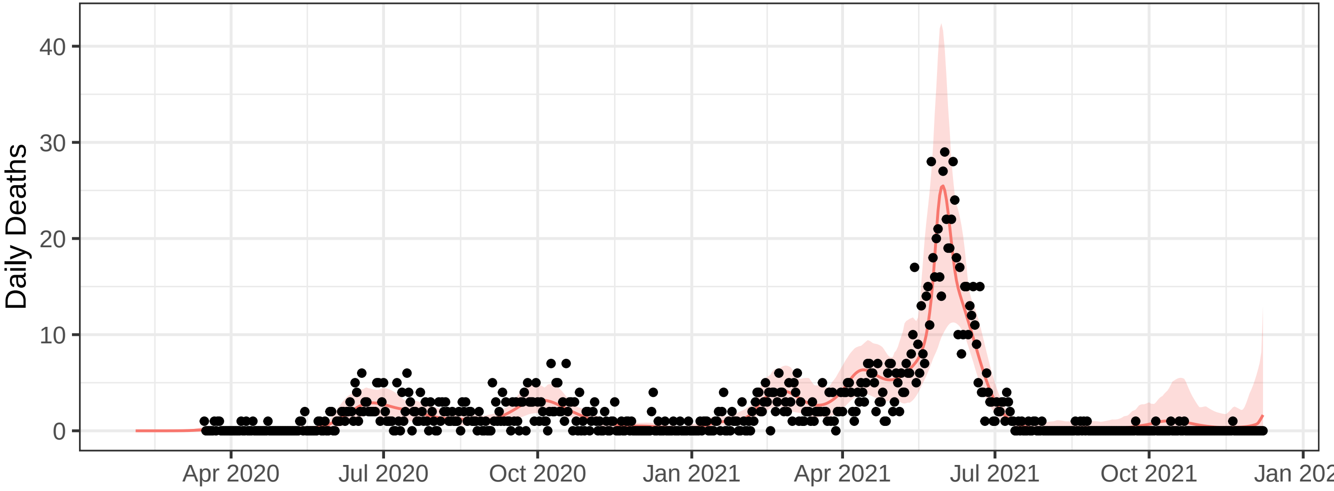

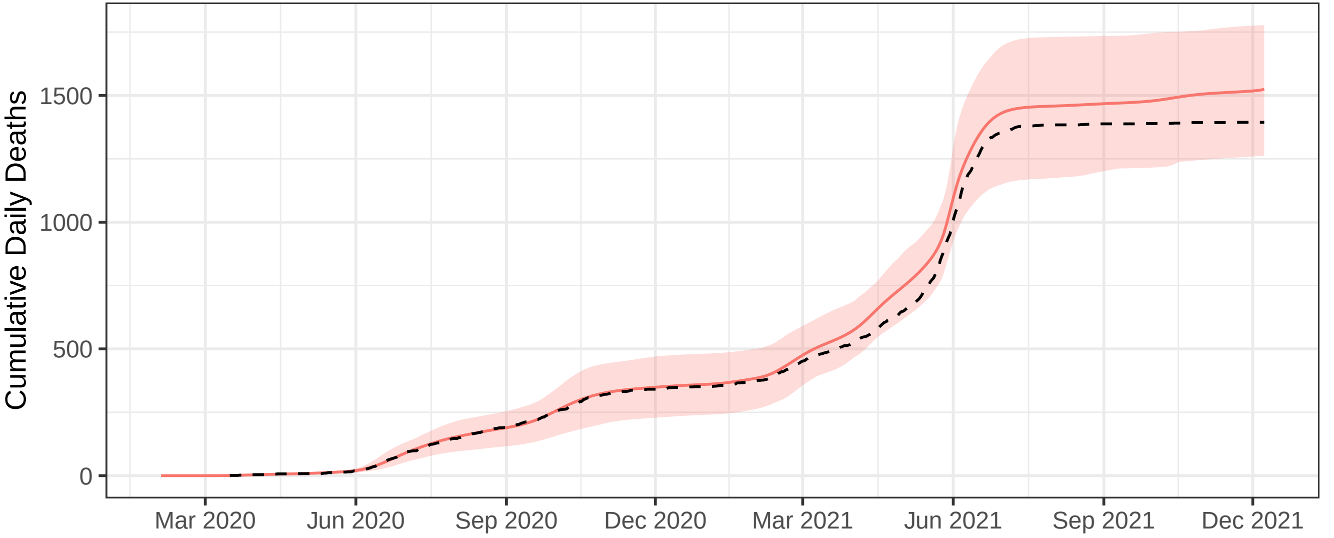

Daily deaths are included with reported COVID−19 deaths shown as points. The red line represents median model estimates of daily deaths, and the shaded region represents the 95% quantiles of the model estimated deaths. Plots of the cumulative estimated deaths are also shown, with the cumulative number of reported deaths represented by a dashed black line.

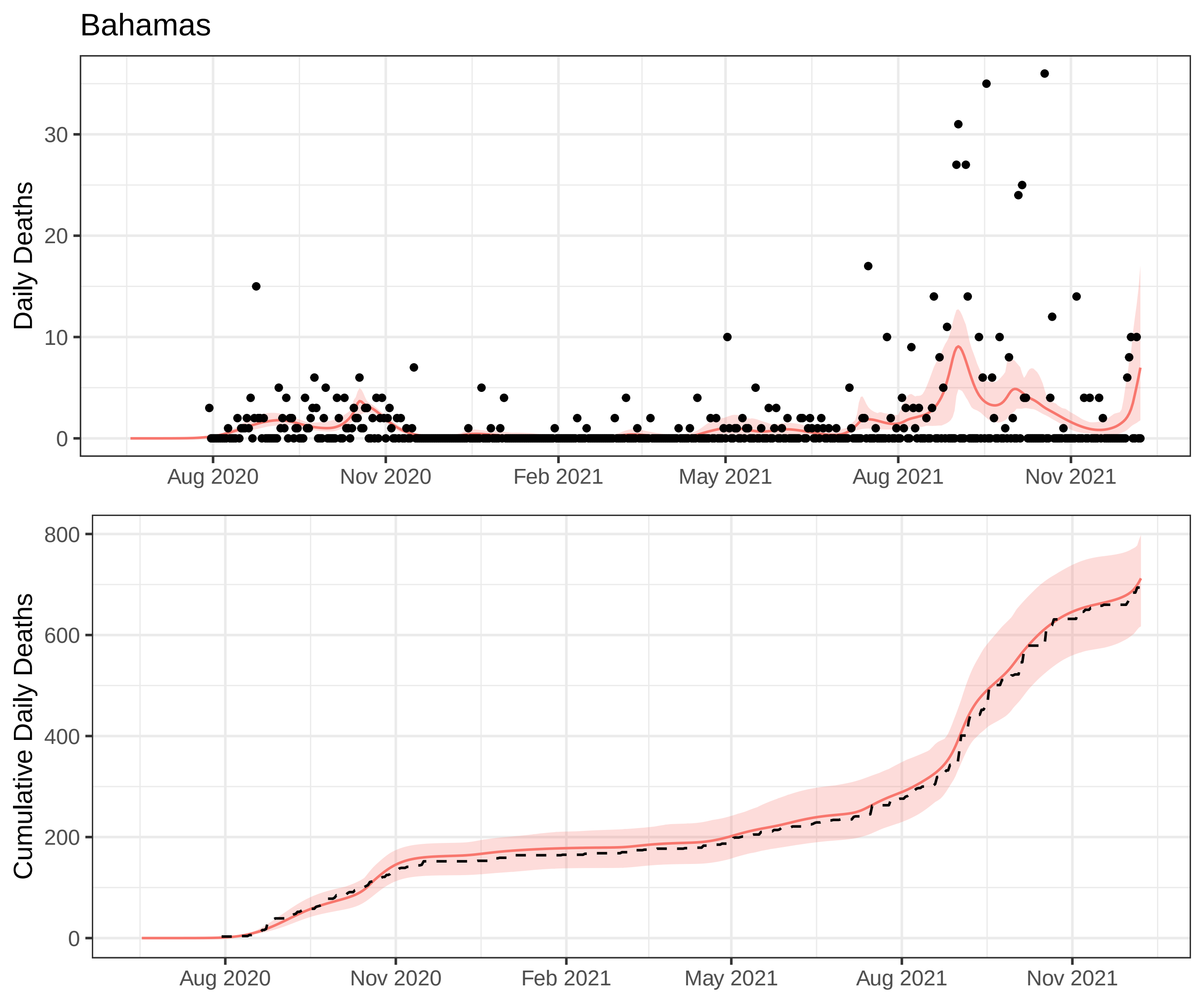

Daily deaths are included with reported COVID−19 deaths shown as points. The red line represents median model estimates of daily deaths, and the shaded region represents the 95% quantiles of the model estimated deaths. Plots of the cumulative estimated deaths are also shown, with the cumulative number of reported deaths represented by a dashed black line.

Bosnia and Herzegovina

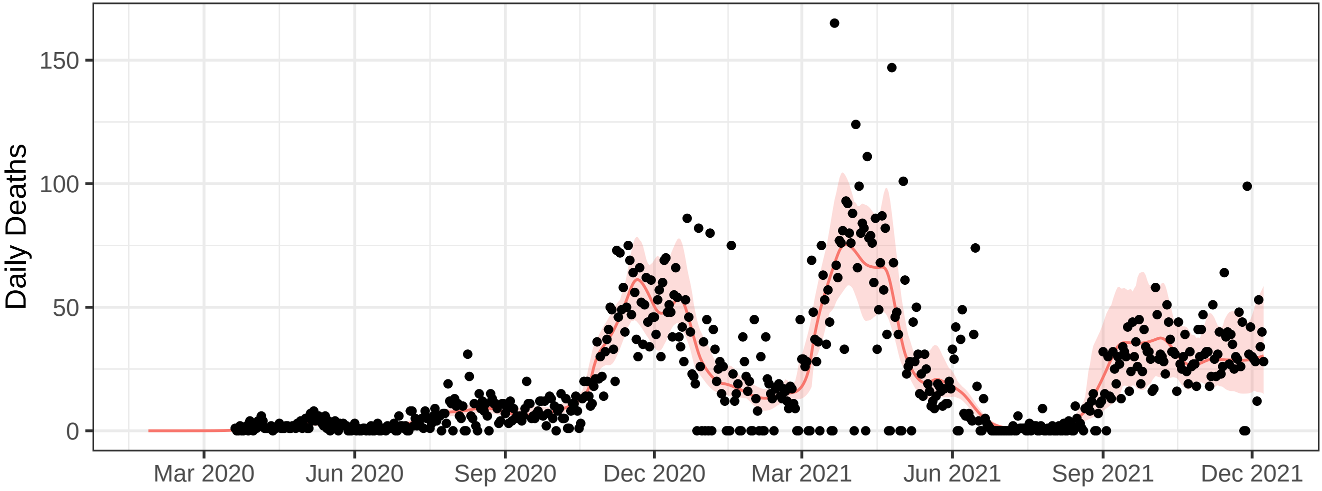

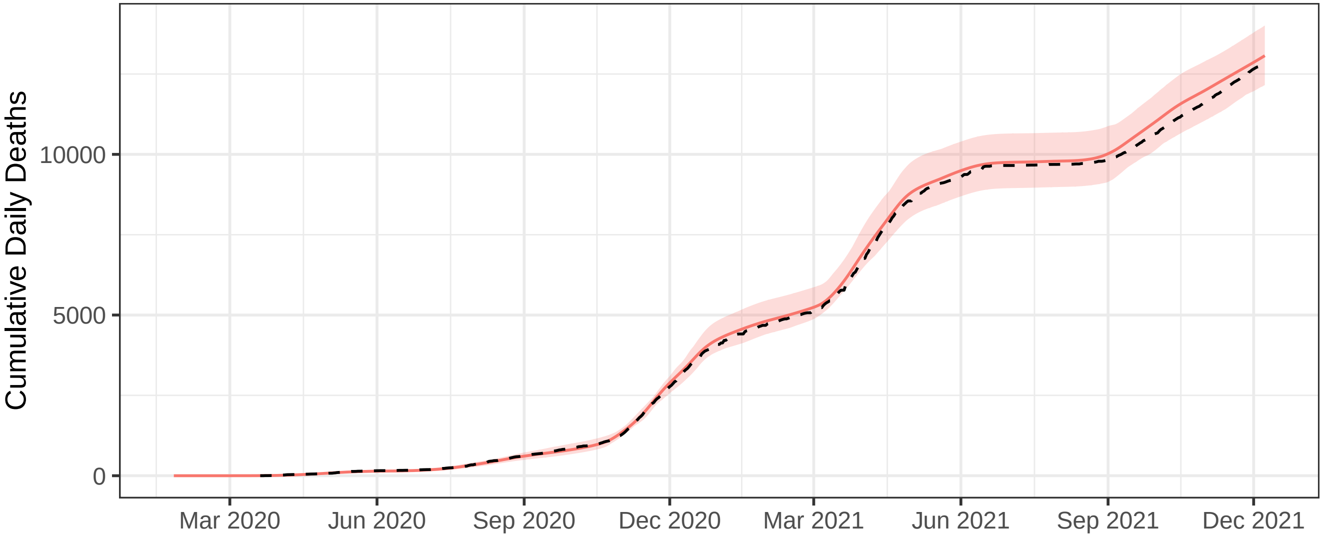

Daily deaths are included with reported COVID−19 deaths shown as points. The red line represents median model estimates of daily deaths, and the shaded region represents the 95% quantiles of the model estimated deaths. Plots of the cumulative estimated deaths are also shown, with the cumulative number of reported deaths represented by a dashed black line.

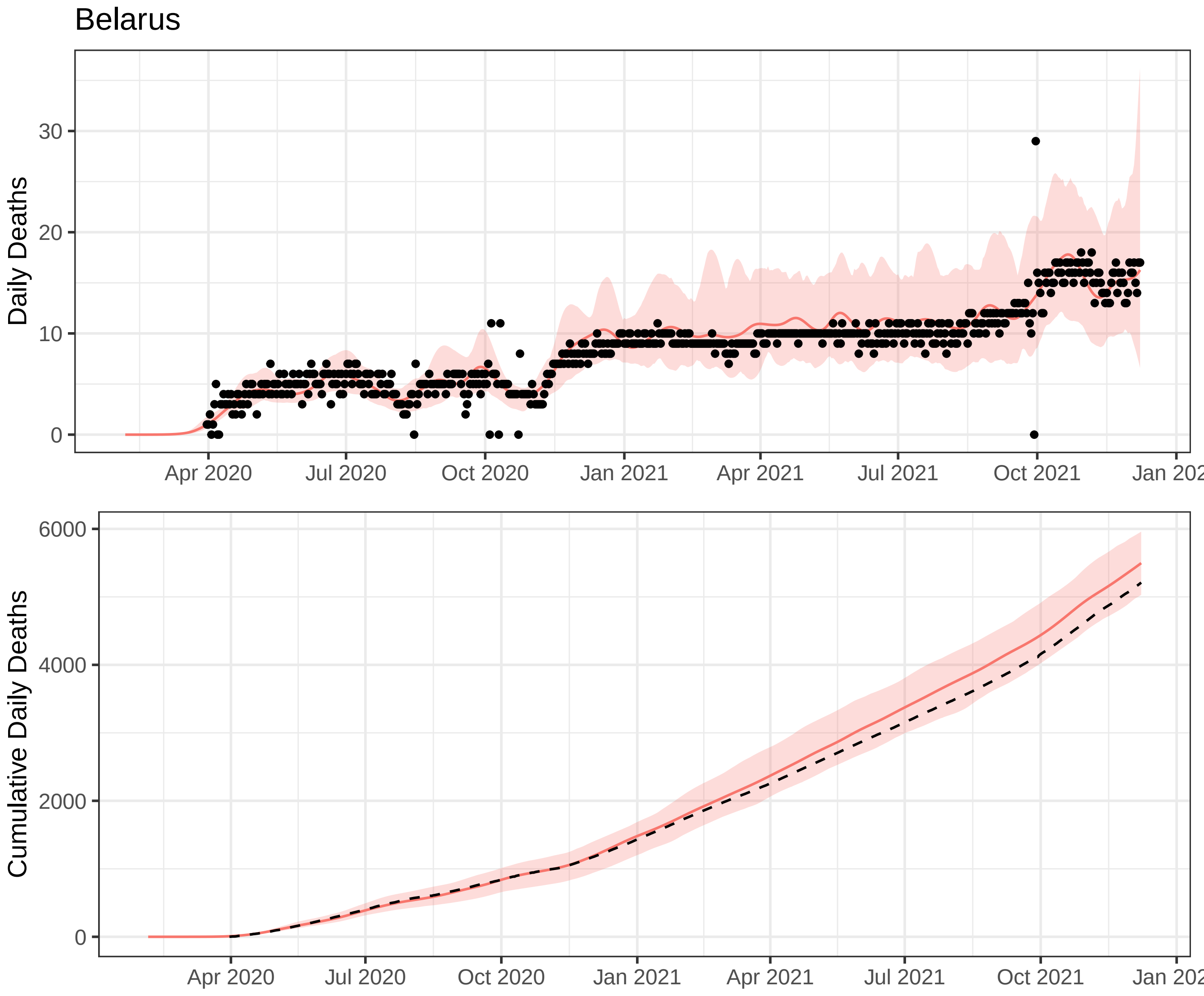

Daily deaths are included with reported COVID−19 deaths shown as points. The red line represents median model estimates of daily deaths, and the shaded region represents the 95% quantiles of the model estimated deaths. Plots of the cumulative estimated deaths are also shown, with the cumulative number of reported deaths represented by a dashed black line.

Belize

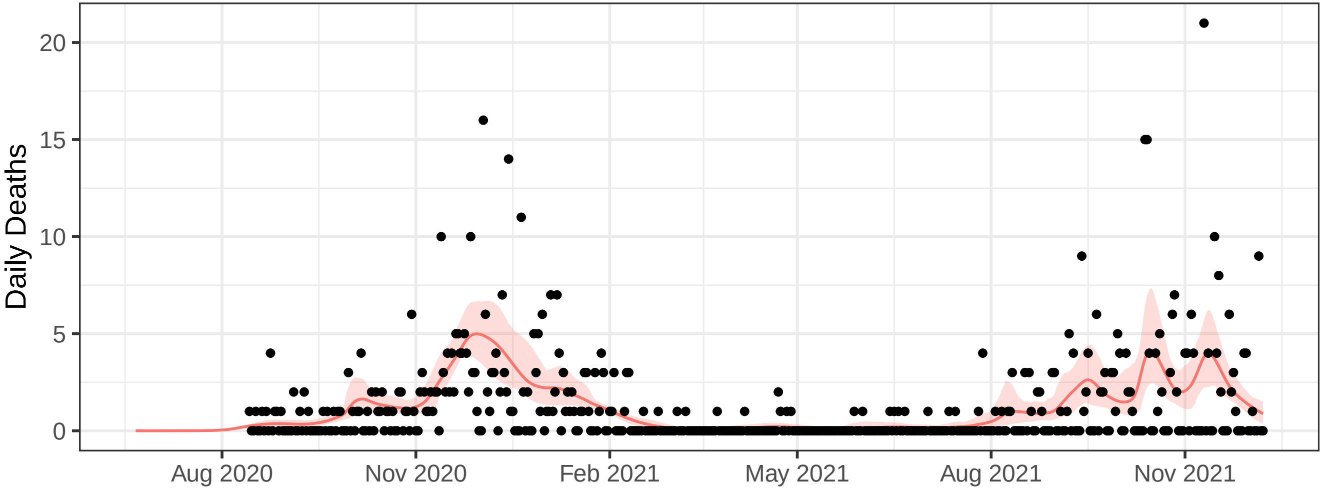

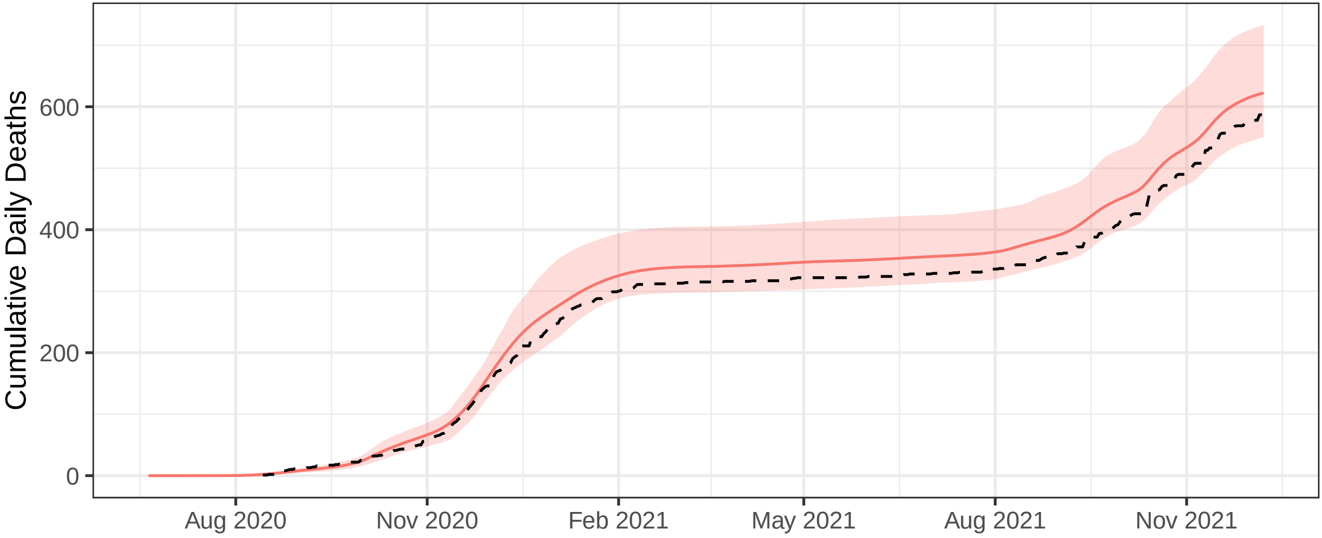

Daily deaths are included with reported COVID−19 deaths shown as points. The red line represents median model estimates of daily deaths, and the shaded region represents the 95% quantiles of the model estimated deaths. Plots of the cumulative estimated deaths are also shown, with the cumulative number of reported deaths represented by a dashed black line.

Bolivia

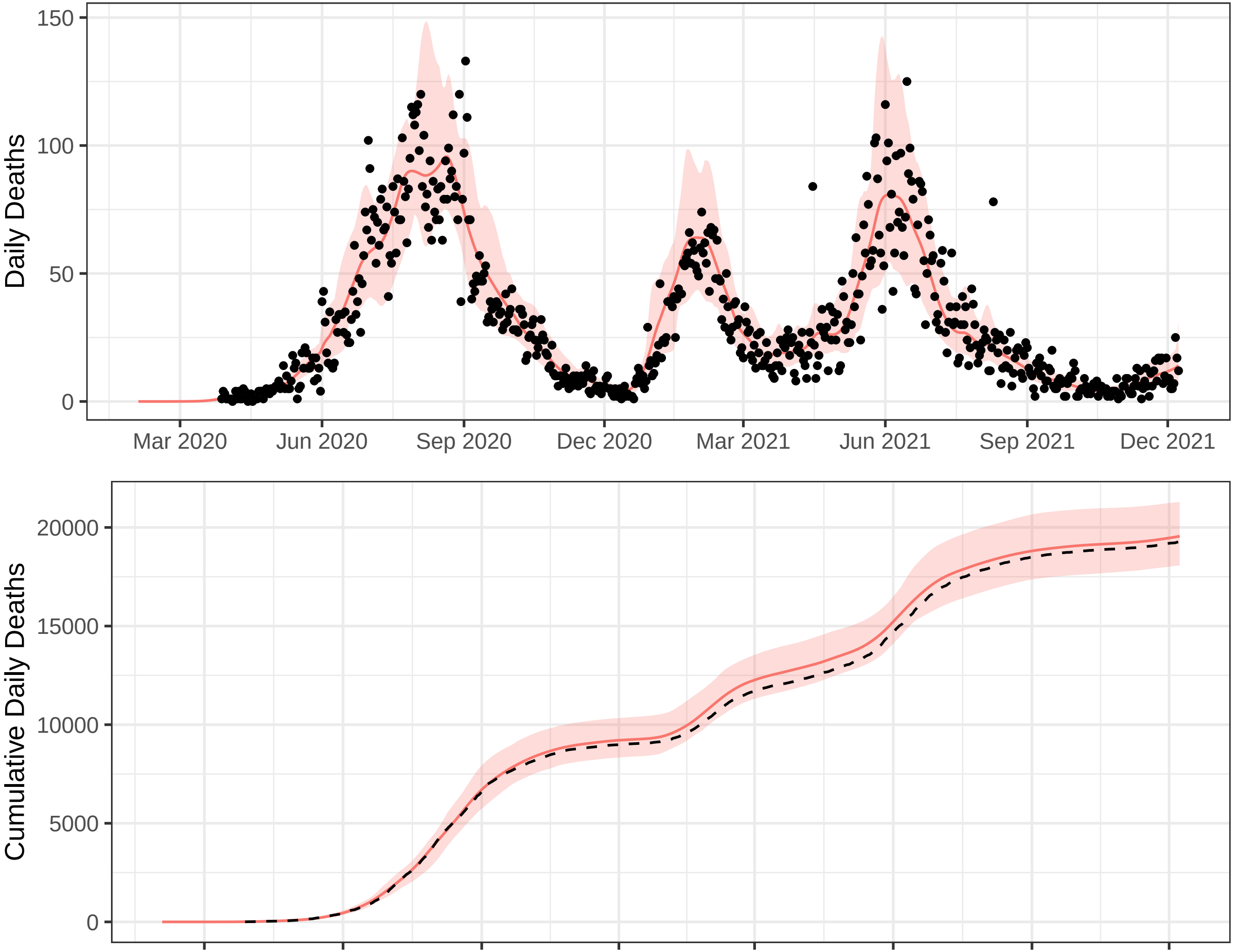

Daily deaths are included with reported COVID−19 deaths shown as points. The red line represents median model estimates of daily deaths, and the shaded region represents the 95% quantiles of the model estimated deaths. Plots of the cumulative estimated deaths are also shown, with the cumulative number of reported deaths represented by a dashed black line.

Brazil

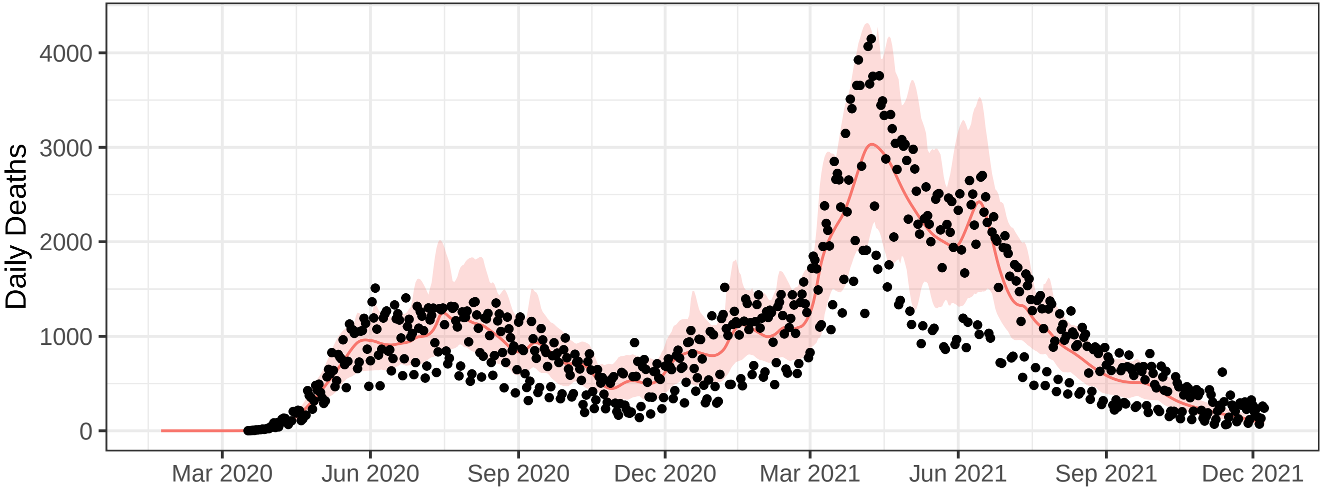

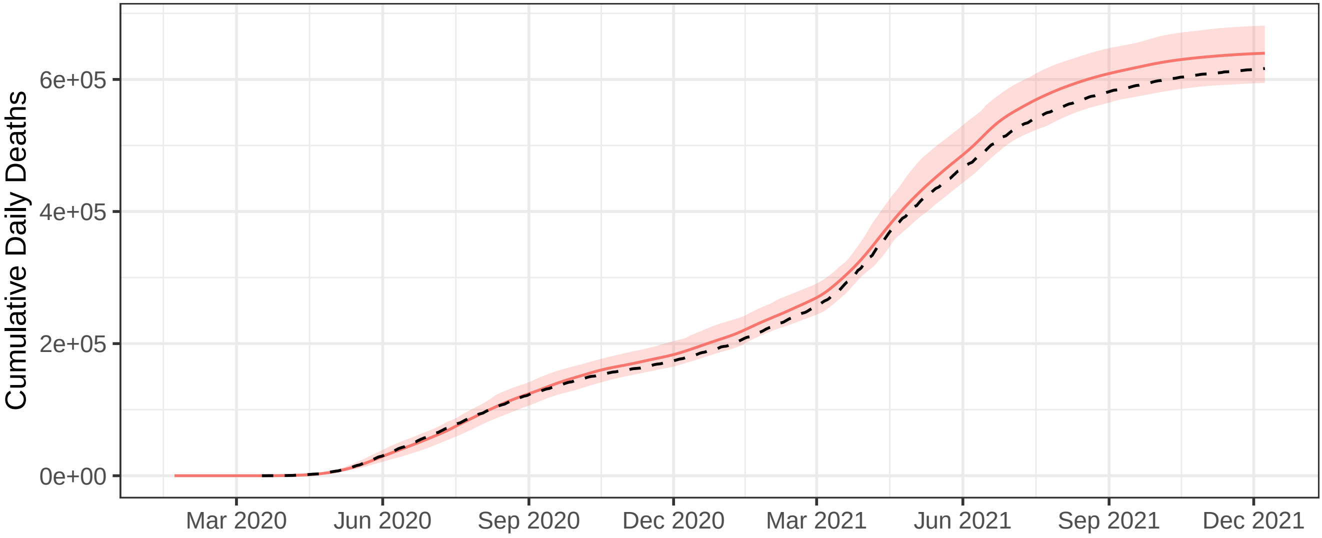

Daily deaths are included with reported COVID−19 deaths shown as points. The red line represents median model estimates of daily deaths, and the shaded region represents the 95% quantiles of the model estimated deaths. Plots of the cumulative estimated deaths are also shown, with the cumulative number of reported deaths represented by a dashed black line.

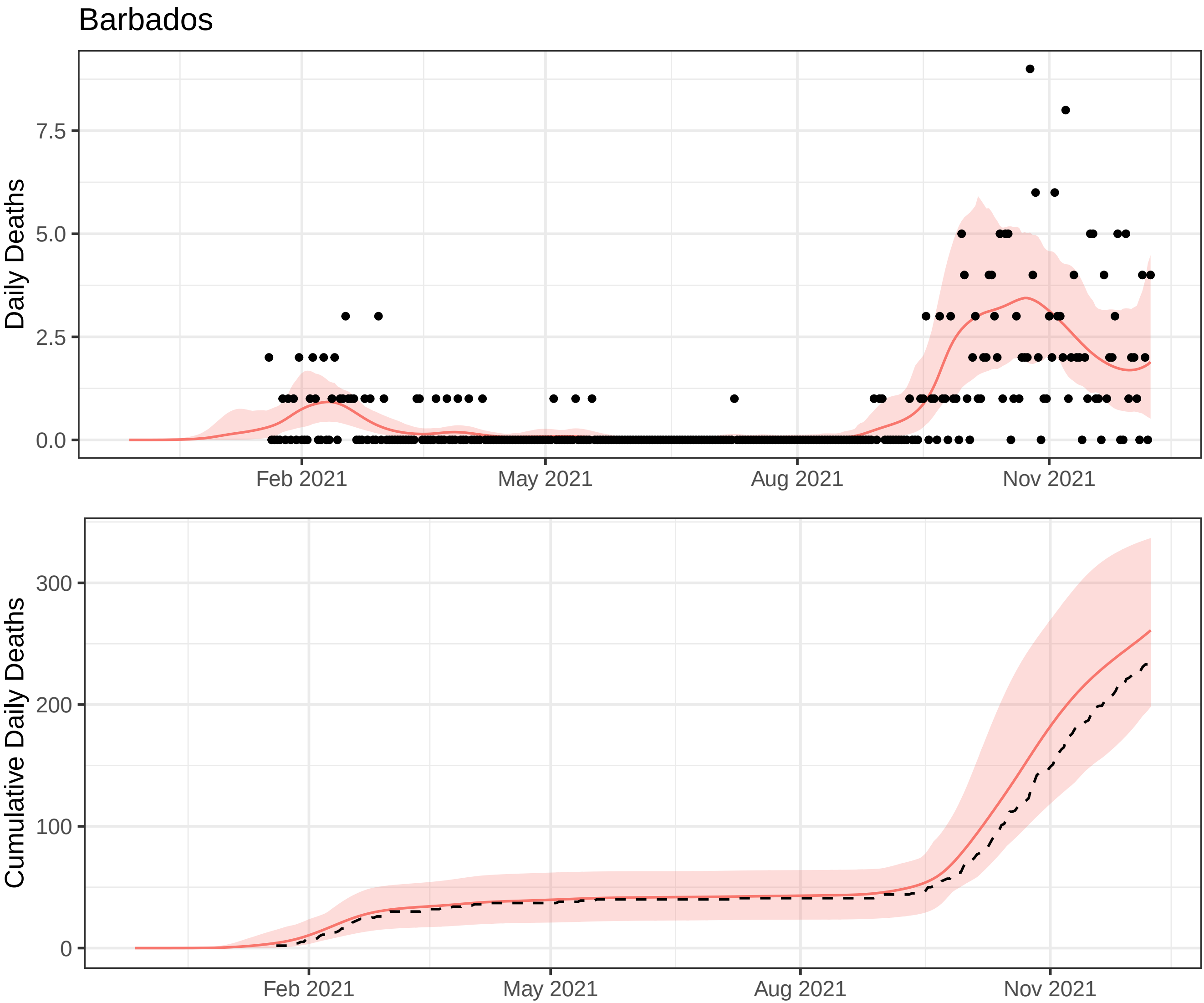

Daily deaths are included with reported COVID−19 deaths shown as points. The red line represents median model estimates of daily deaths, and the shaded region represents the 95% quantiles of the model estimated deaths. Plots of the cumulative estimated deaths are also shown, with the cumulative number of reported deaths represented by a dashed black line.

Brunei Darussalam

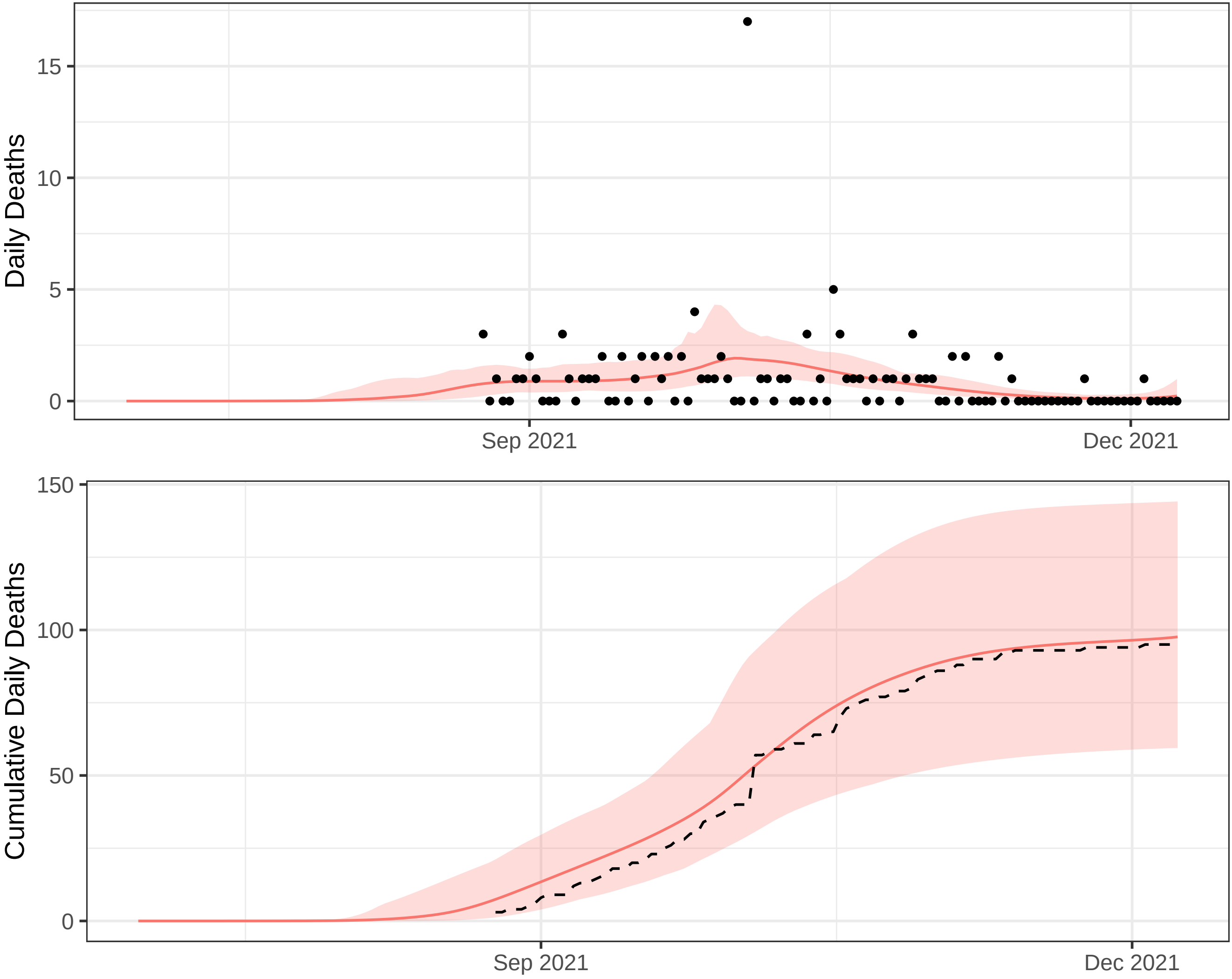

Daily deaths are included with reported COVID−19 deaths shown as points. The red line represents median model estimates of daily deaths, and the shaded region represents the 95% quantiles of the model estimated deaths. Plots of the cumulative estimated deaths are also shown, with the cumulative number of reported deaths represented by a dashed black line.

Bhutan

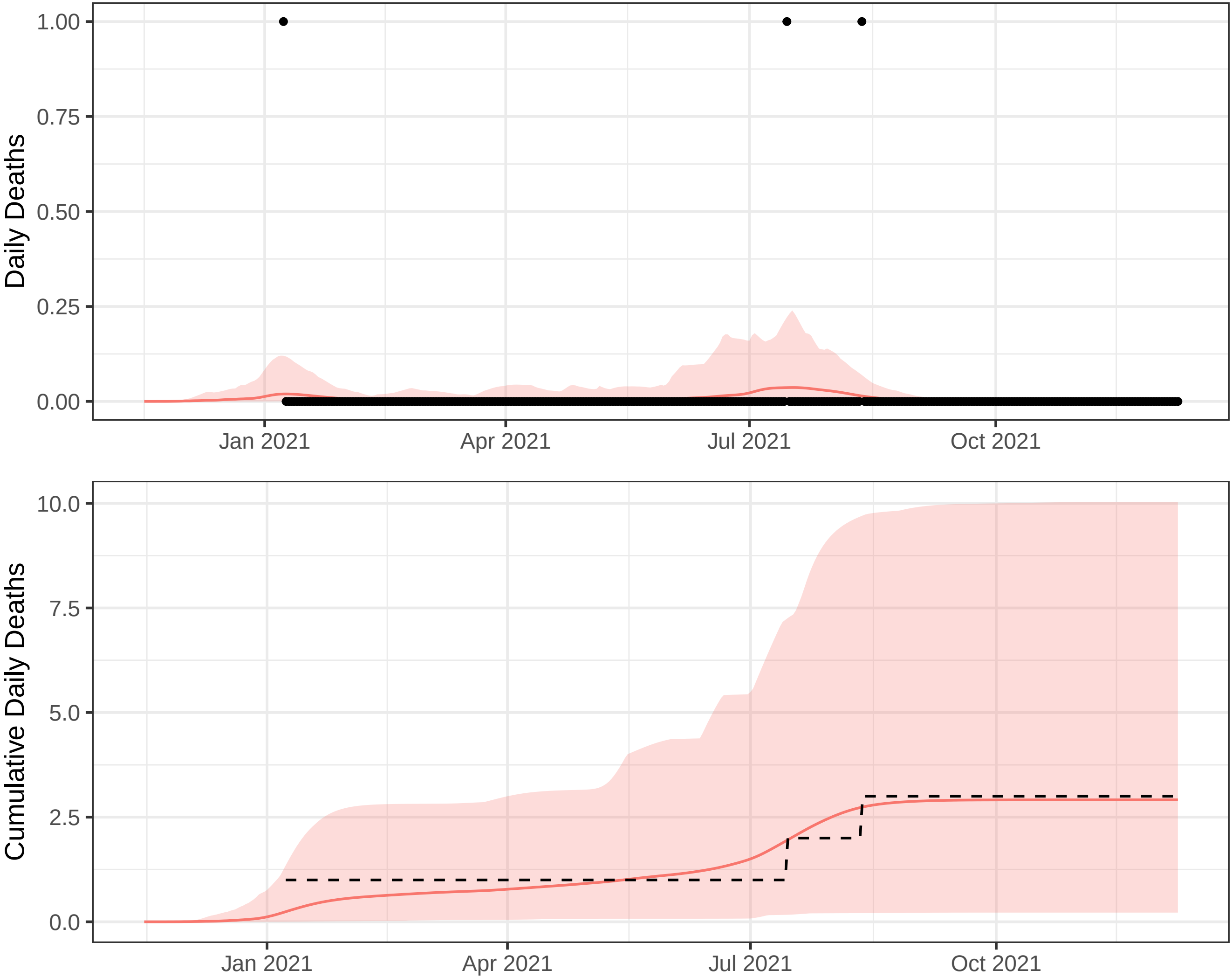

Daily deaths are included with reported COVID−19 deaths shown as points. The red line represents median model estimates of daily deaths, and the shaded region represents the 95% quantiles of the model estimated deaths. Plots of the cumulative estimated deaths are also shown, with the cumulative number of reported deaths represented by a dashed black line.

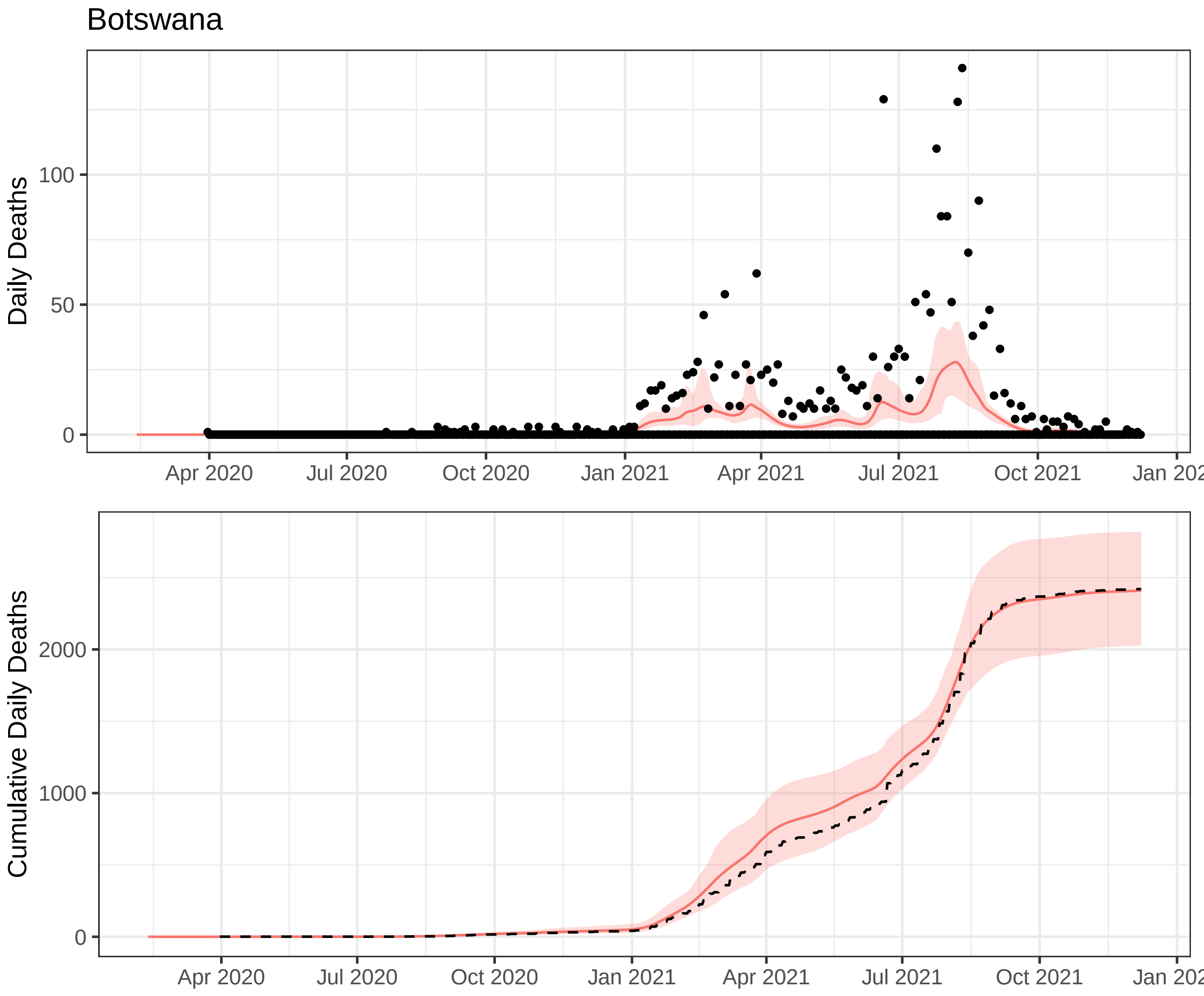

Daily deaths are included with reported COVID−19 deaths shown as points. The red line represents median model estimates of daily deaths, and the shaded region represents the 95% quantiles of the model estimated deaths. Plots of the cumulative estimated deaths are also shown, with the cumulative number of reported deaths represented by a dashed black line.

Central African Republic

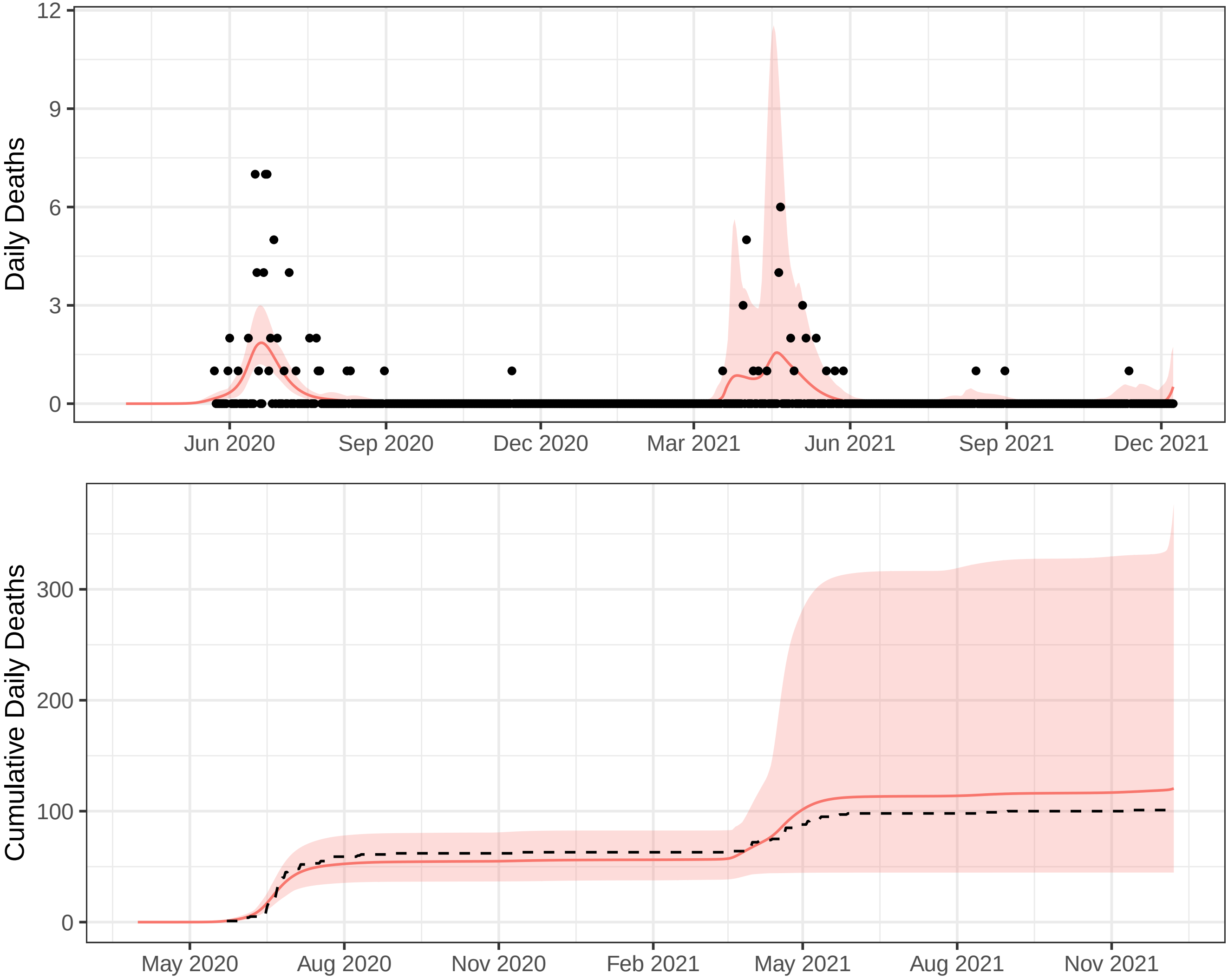

Daily deaths are included with reported COVID−19 deaths shown as points. The red line represents median model estimates of daily deaths, and the shaded region represents the 95% quantiles of the model estimated deaths. Plots of the cumulative estimated deaths are also shown, with the cumulative number of reported deaths represented by a dashed black line.

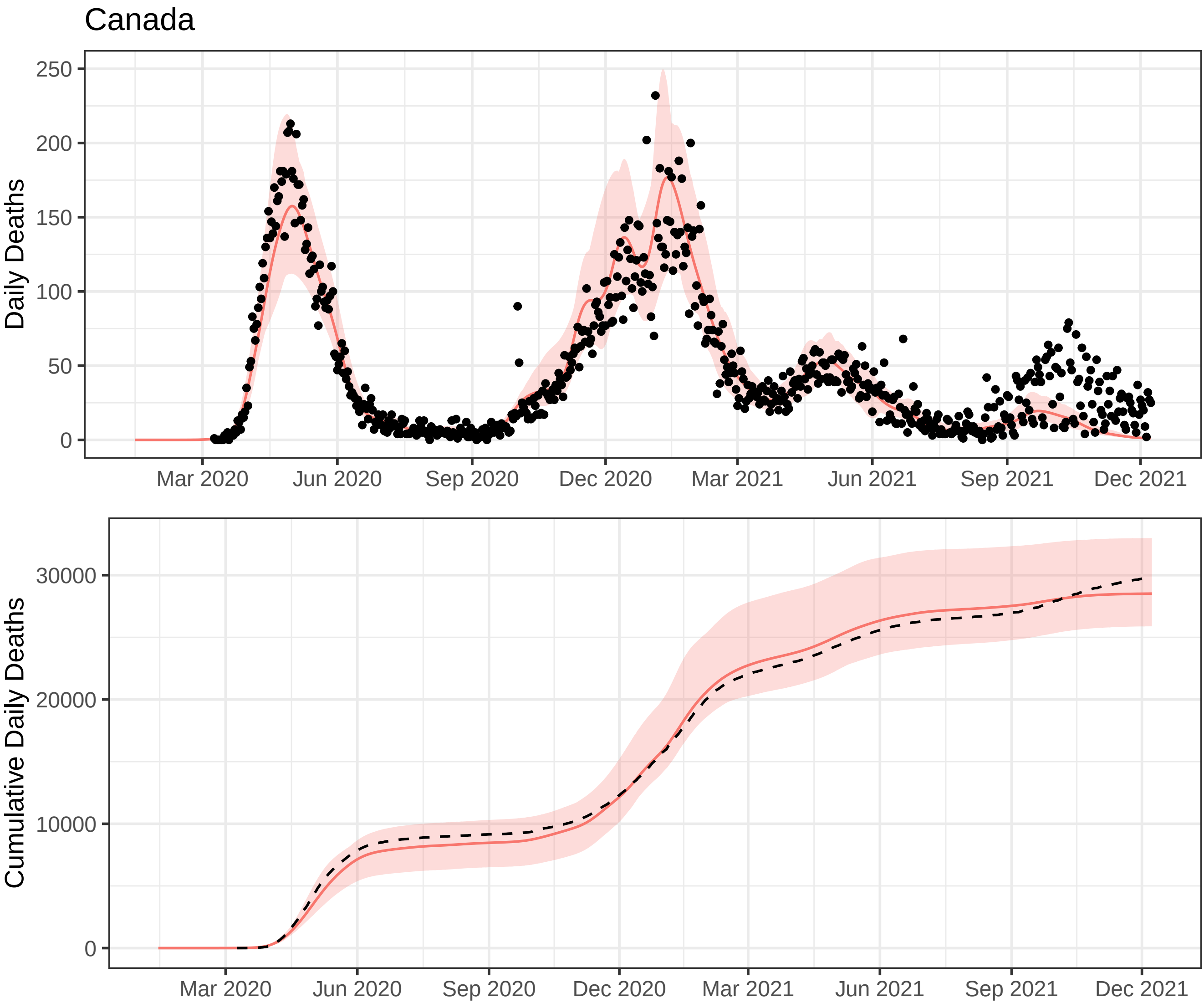

Daily deaths are included with reported COVID−19 deaths shown as points. The red line represents median model estimates of daily deaths, and the shaded region represents the 95% quantiles of the model estimated deaths. Plots of the cumulative estimated deaths are also shown, with the cumulative number of reported deaths represented by a dashed black line.

Switzerland

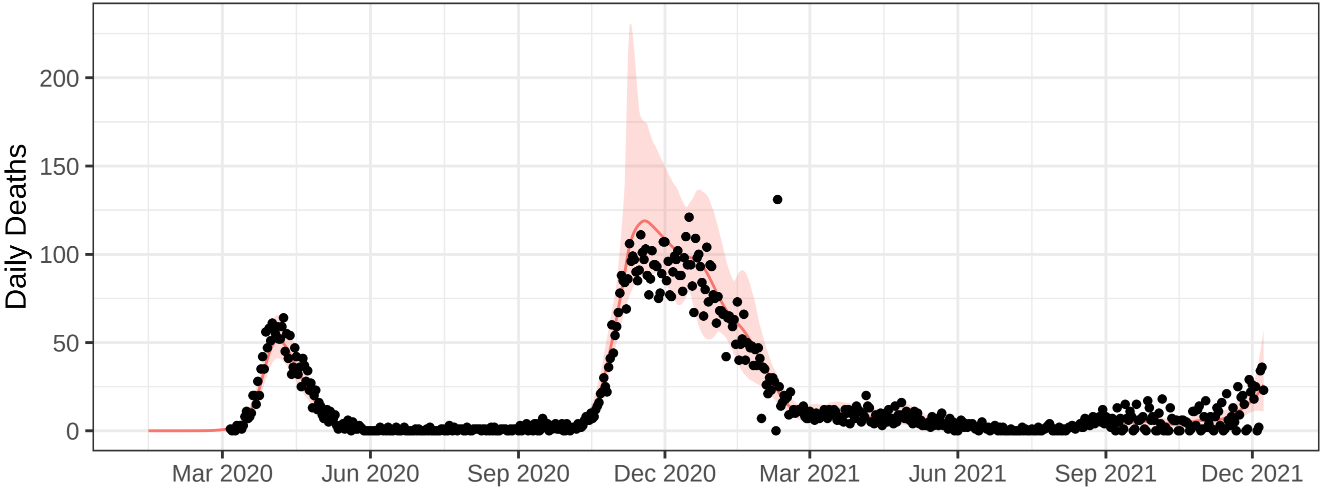

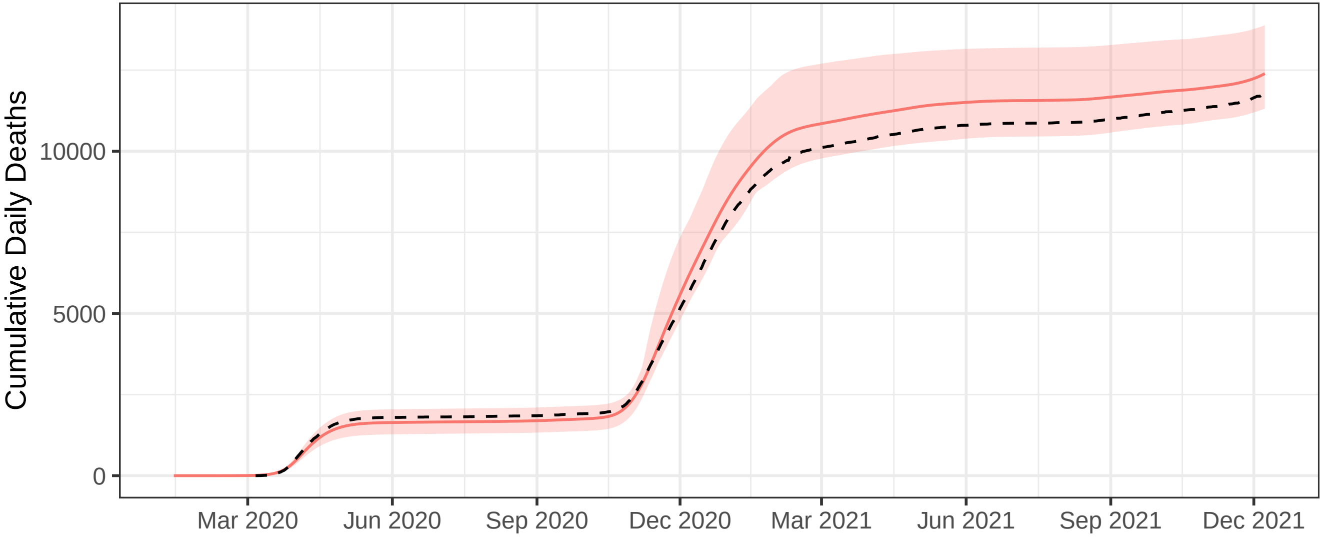

Daily deaths are included with reported COVID−19 deaths shown as points. The red line represents median model estimates of daily deaths, and the shaded region represents the 95% quantiles of the model estimated deaths. Plots of the cumulative estimated deaths are also shown, with the cumulative number of reported deaths represented by a dashed black line.

Chile

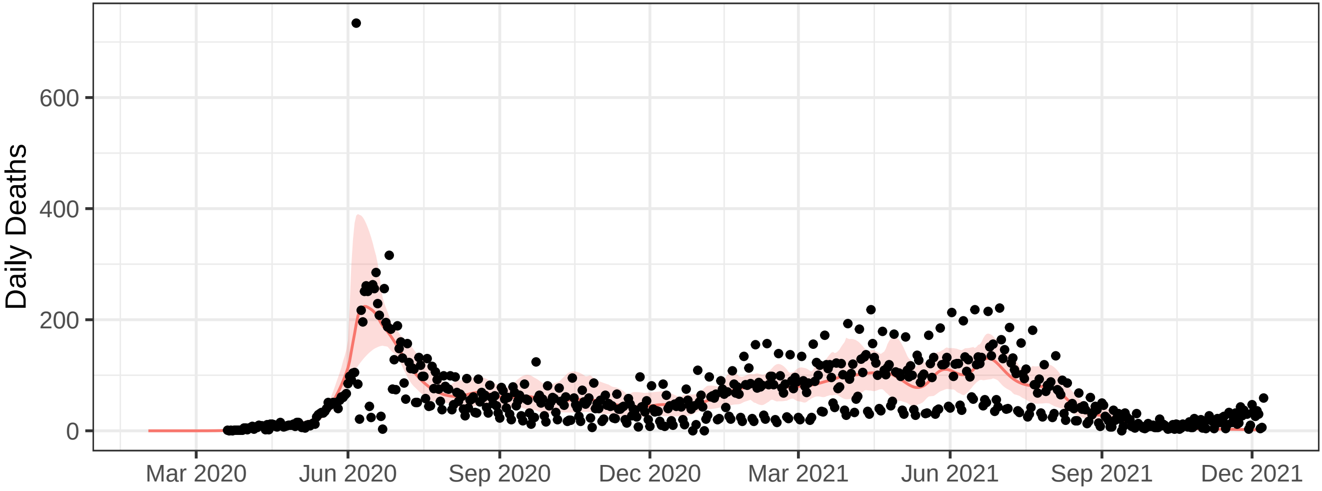

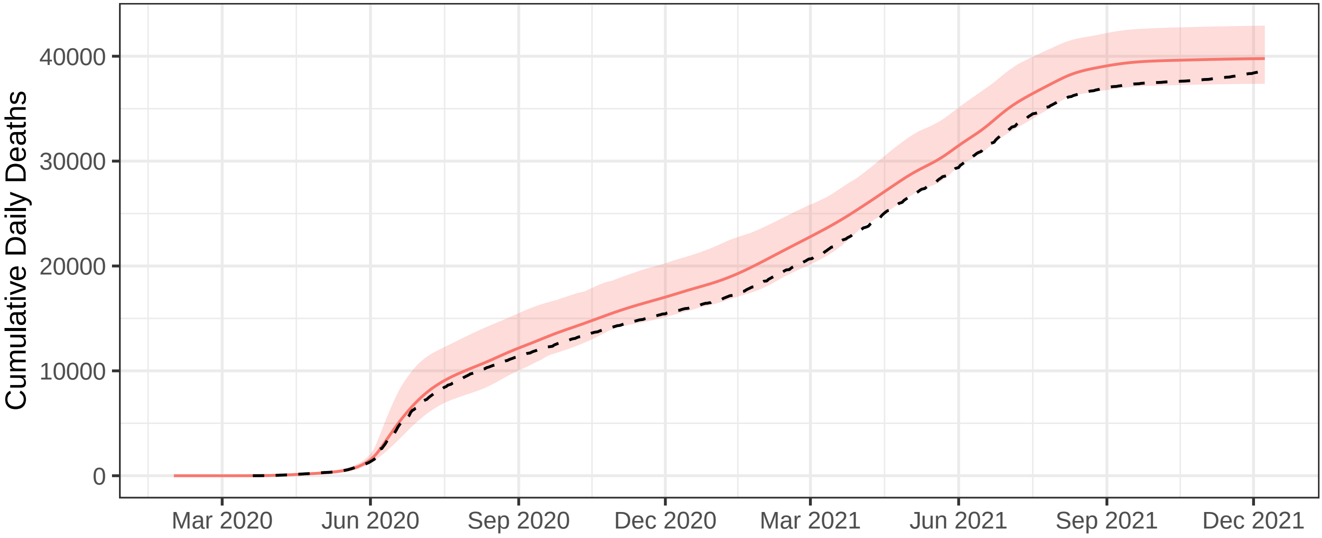

Daily deaths are included with reported COVID−19 deaths shown as points. The red line represents median model estimates of daily deaths, and the shaded region represents the 95% quantiles of the model estimated deaths. Plots of the cumulative estimated deaths are also shown, with the cumulative number of reported deaths represented by a dashed black line.

Cote d'Ivoire

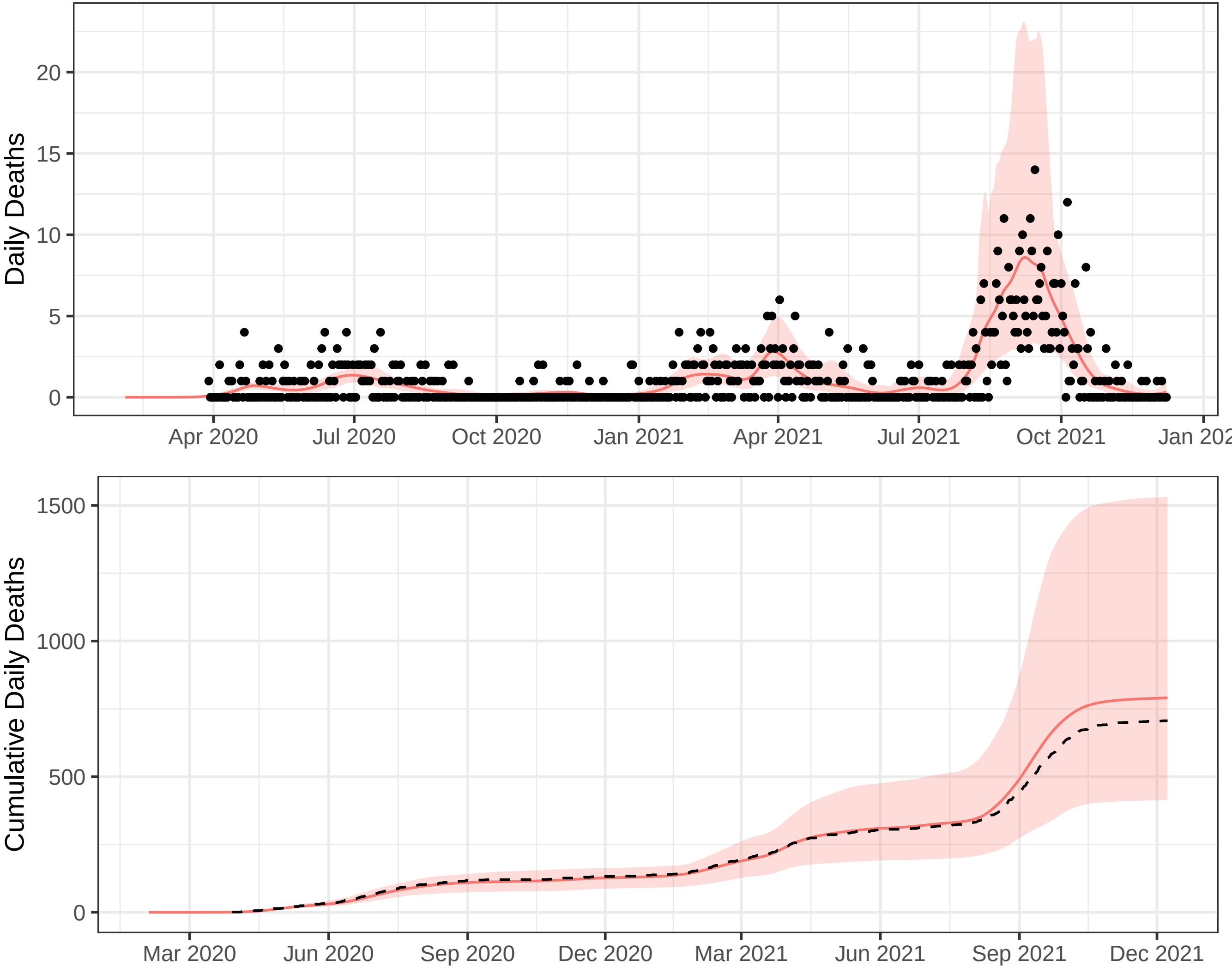

Daily deaths are included with reported COVID−19 deaths shown as points. The red line represents median model estimates of daily deaths, and the shaded region represents the 95% quantiles of the model estimated deaths. Plots of the cumulative estimated deaths are also shown, with the cumulative number of reported deaths represented by a dashed black line.

Cameroon

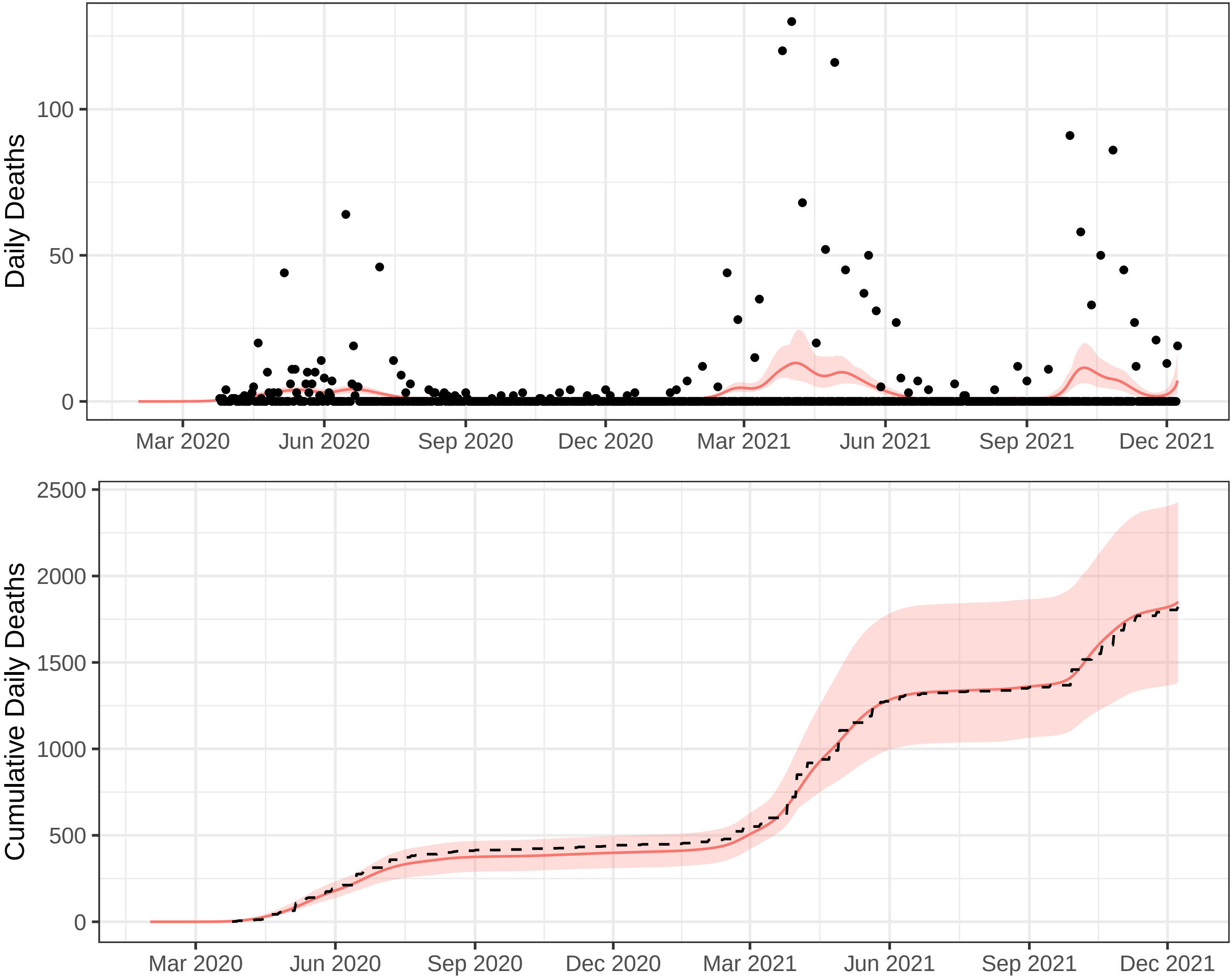

Daily deaths are included with reported COVID−19 deaths shown as points. The red line represents median model estimates of daily deaths, and the shaded region represents the 95% quantiles of the model estimated deaths. Plots of the cumulative estimated deaths are also shown, with the cumulative number of reported deaths represented by a dashed black line.

Democratic Republic of Congo

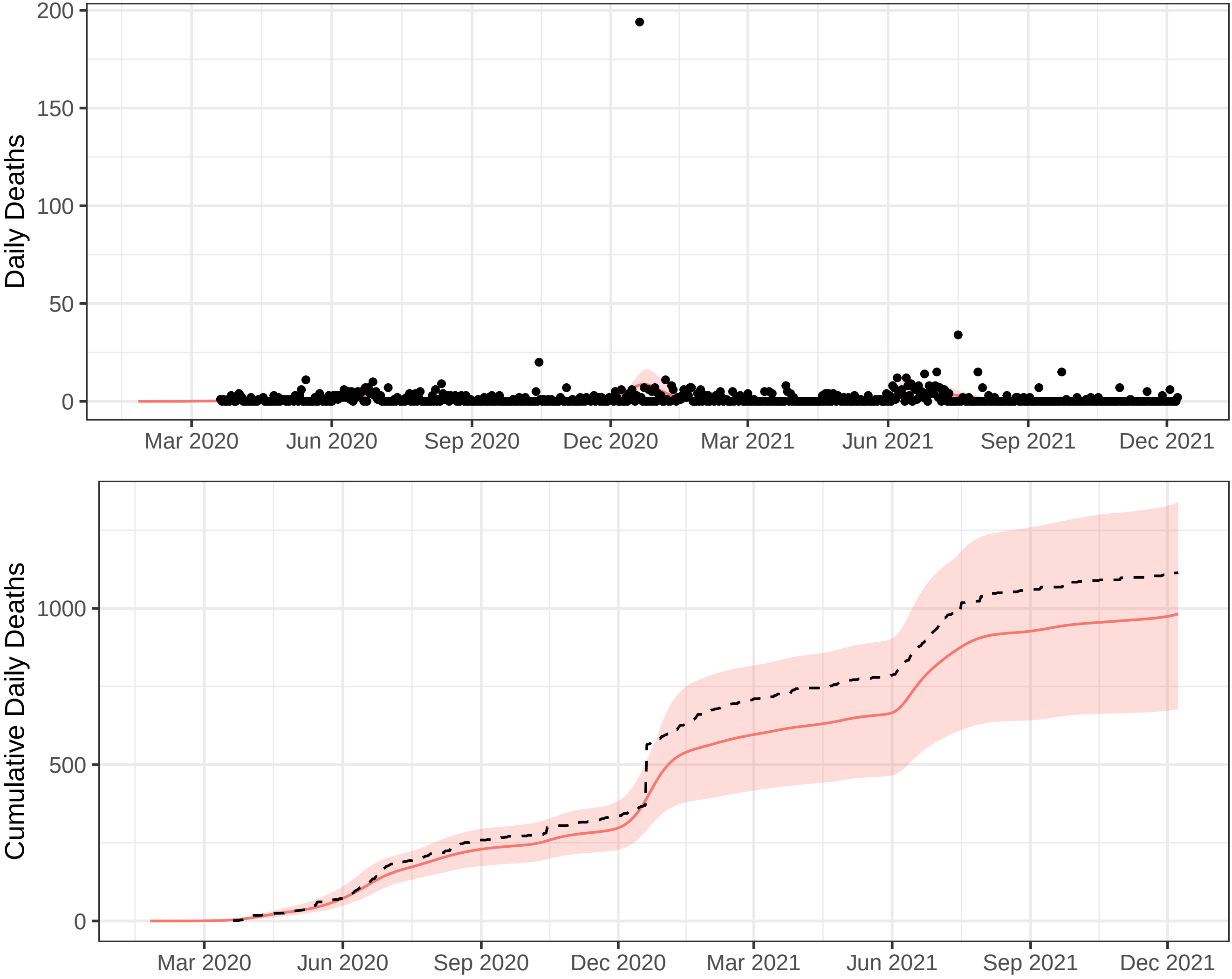

Daily deaths are included with reported COVID−19 deaths shown as points. The red line represents median model estimates of daily deaths, and the shaded region represents the 95% quantiles of the model estimated deaths. Plots of the cumulative estimated deaths are also shown, with the cumulative number of reported deaths represented by a dashed black line.

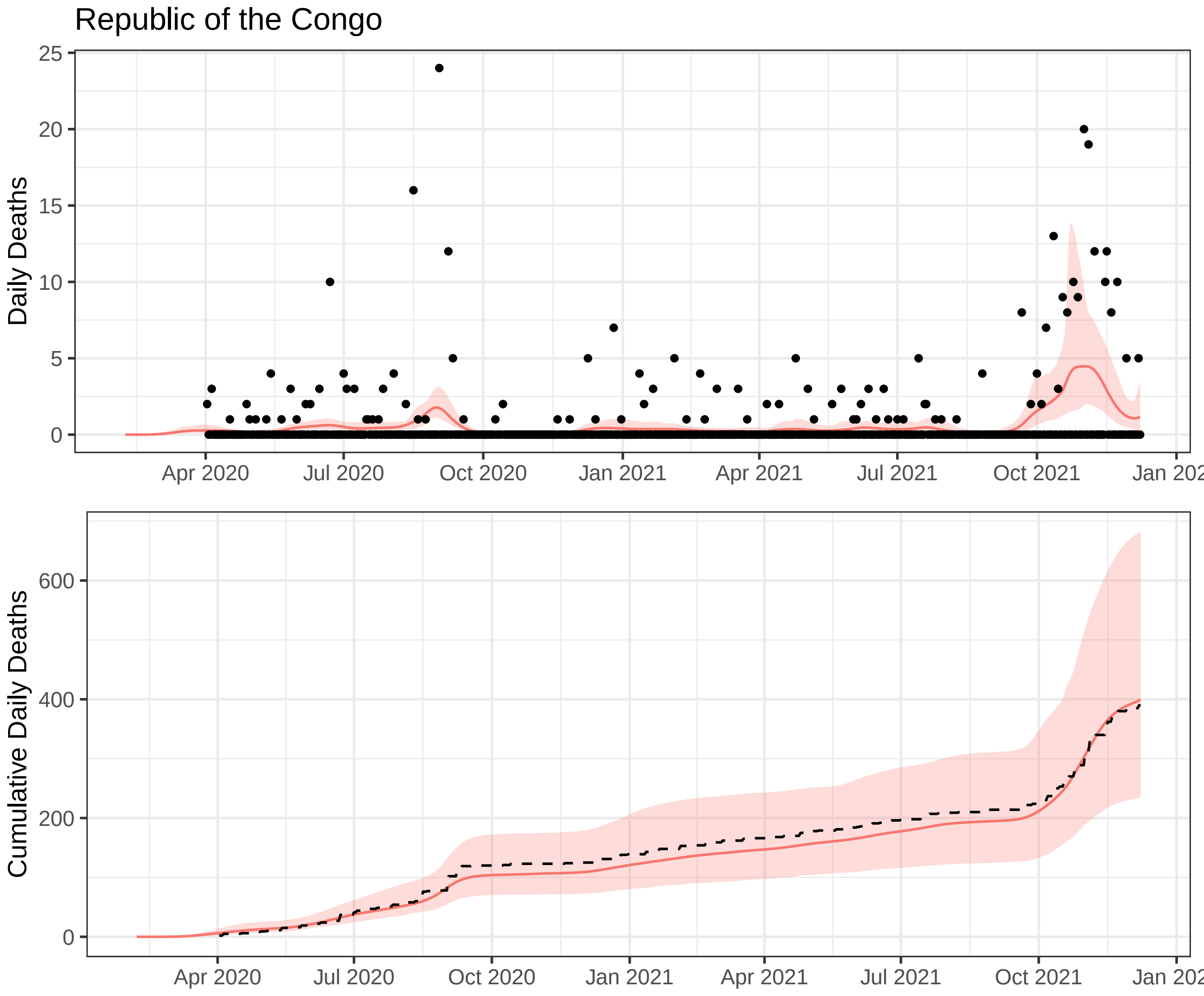

Daily deaths are included with reported COVID−19 deaths shown as points. The red line represents median model estimates of daily deaths, and the shaded region represents the 95% quantiles of the model estimated deaths. Plots of the cumulative estimated deaths are also shown, with the cumulative number of reported deaths represented by a dashed black line.

Colombia

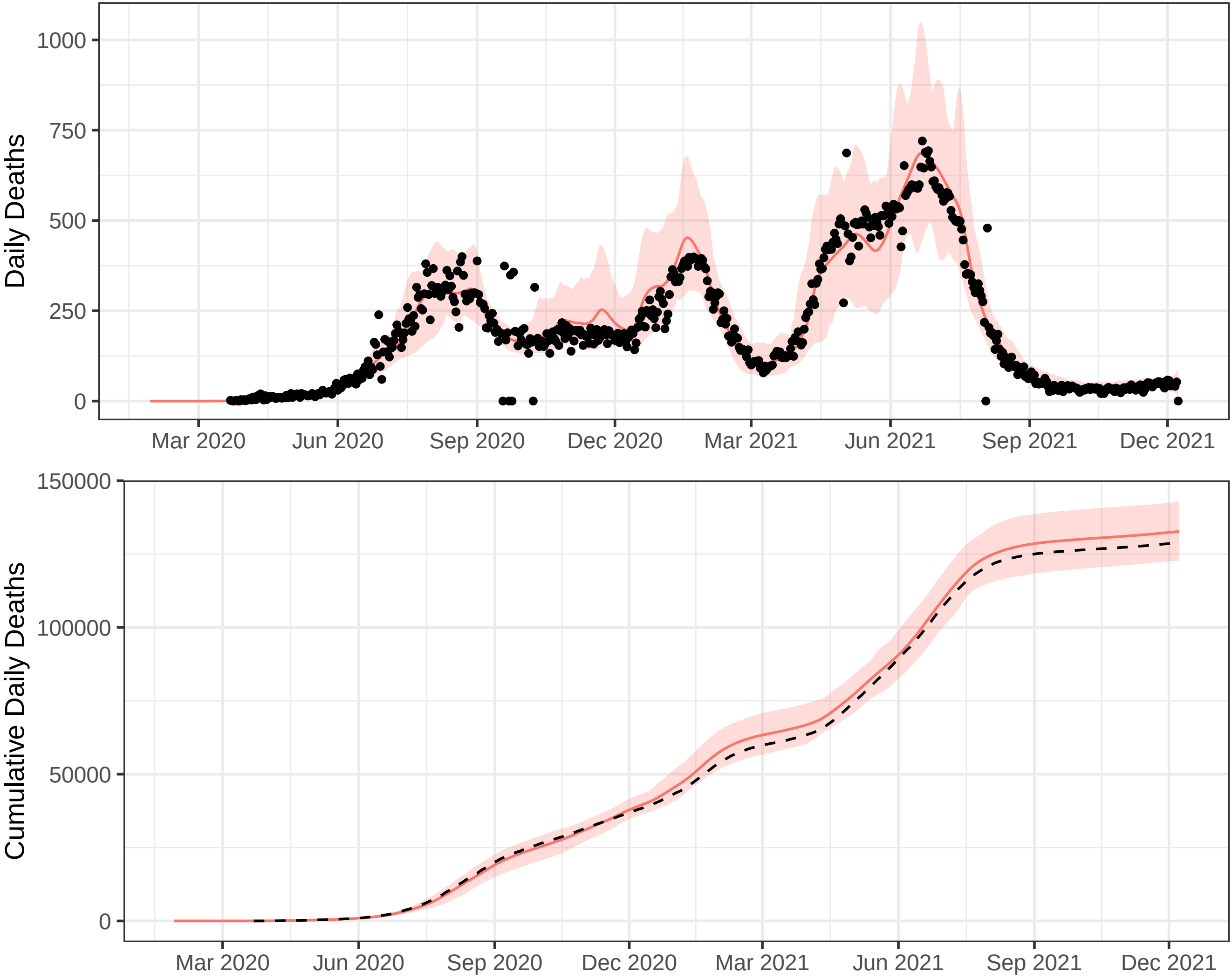

Daily deaths are included with reported COVID−19 deaths shown as points. The red line represents median model estimates of daily deaths, and the shaded region represents the 95% quantiles of the model estimated deaths. Plots of the cumulative estimated deaths are also shown, with the cumulative number of reported deaths represented by a dashed black line.

Comoros

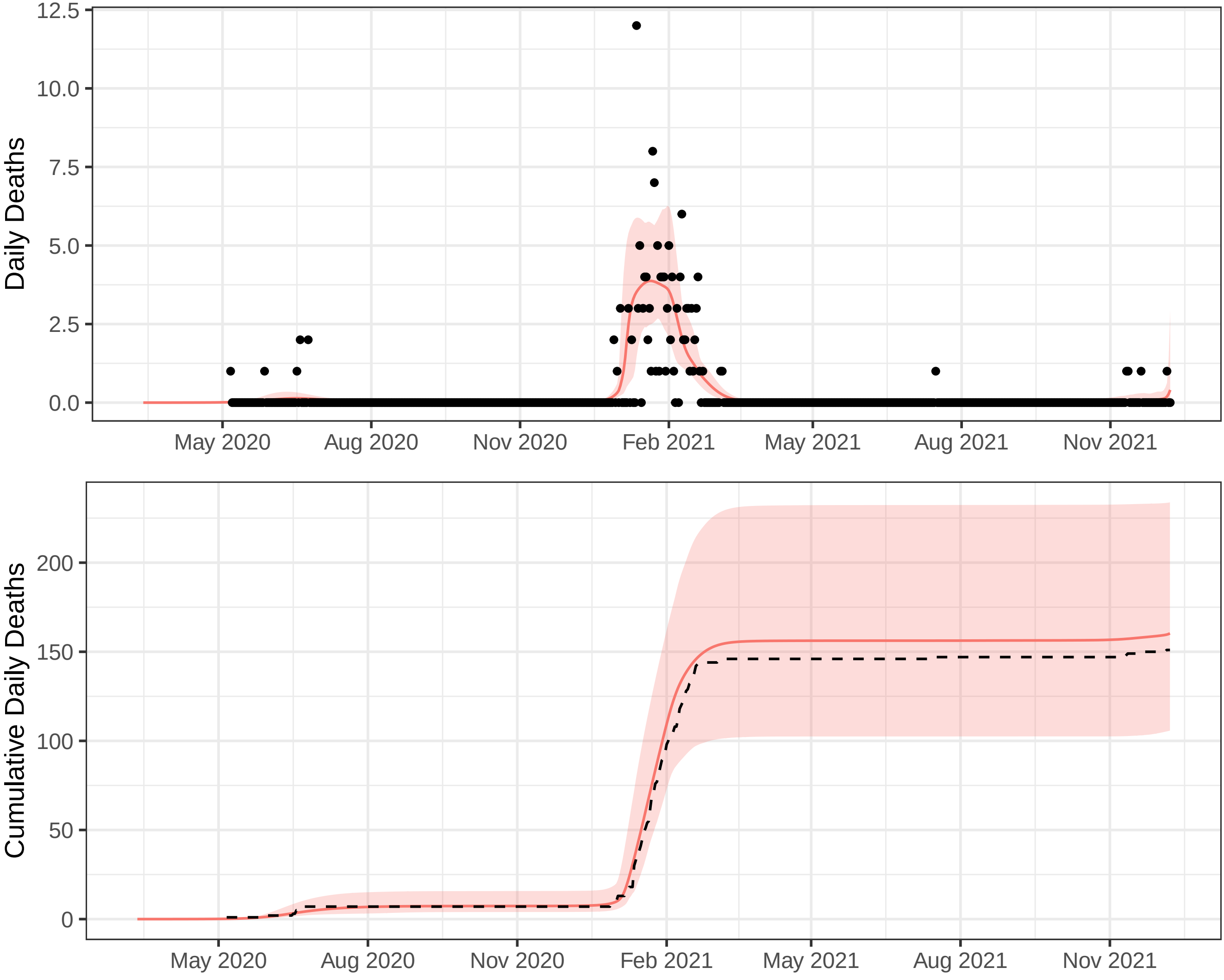

Daily deaths are included with reported COVID−19 deaths shown as points. The red line represents median model estimates of daily deaths, and the shaded region represents the 95% quantiles of the model estimated deaths. Plots of the cumulative estimated deaths are also shown, with the cumulative number of reported deaths represented by a dashed black line.

Cabo Verde

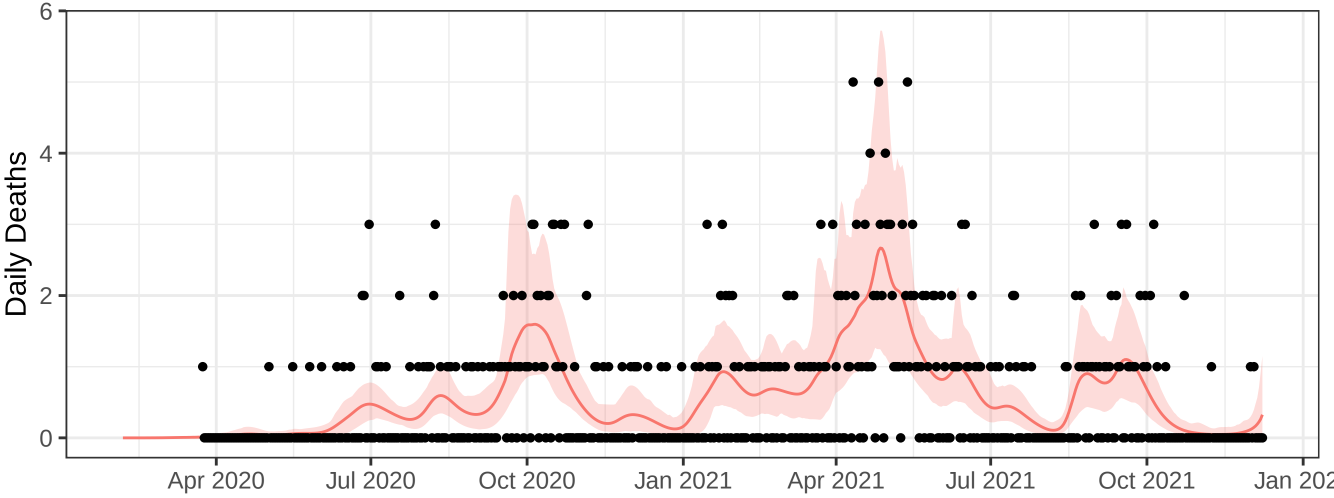

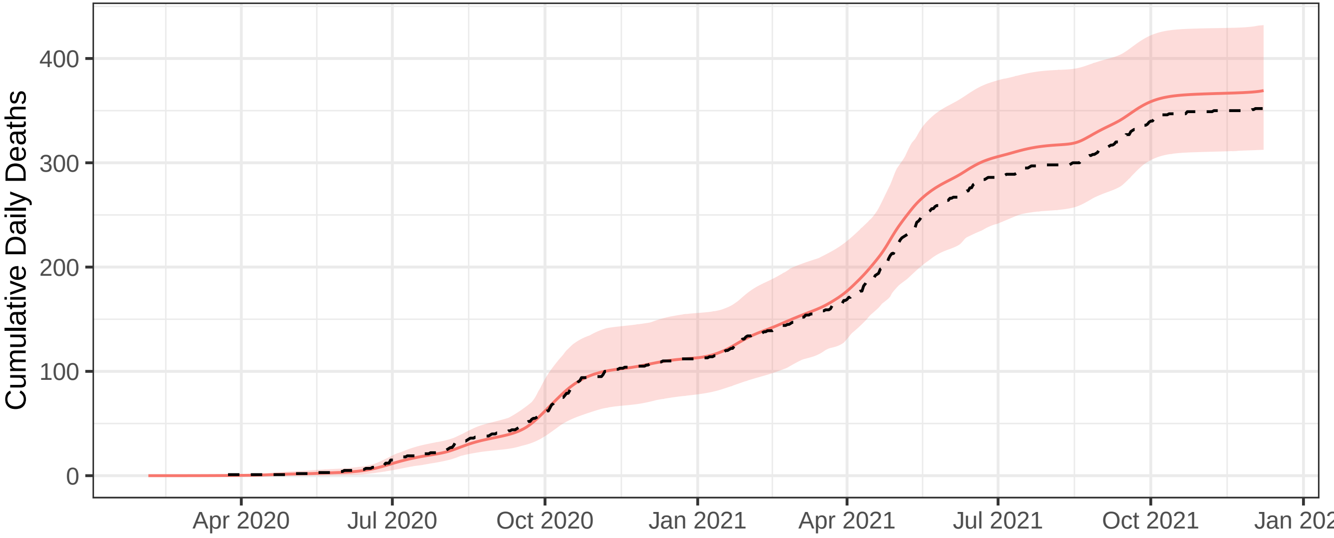

Daily deaths are included with reported COVID−19 deaths shown as points. The red line represents median model estimates of daily deaths, and the shaded region represents the 95% quantiles of the model estimated deaths. Plots of the cumulative estimated deaths are also shown, with the cumulative number of reported deaths represented by a dashed black line.

Costa Rica

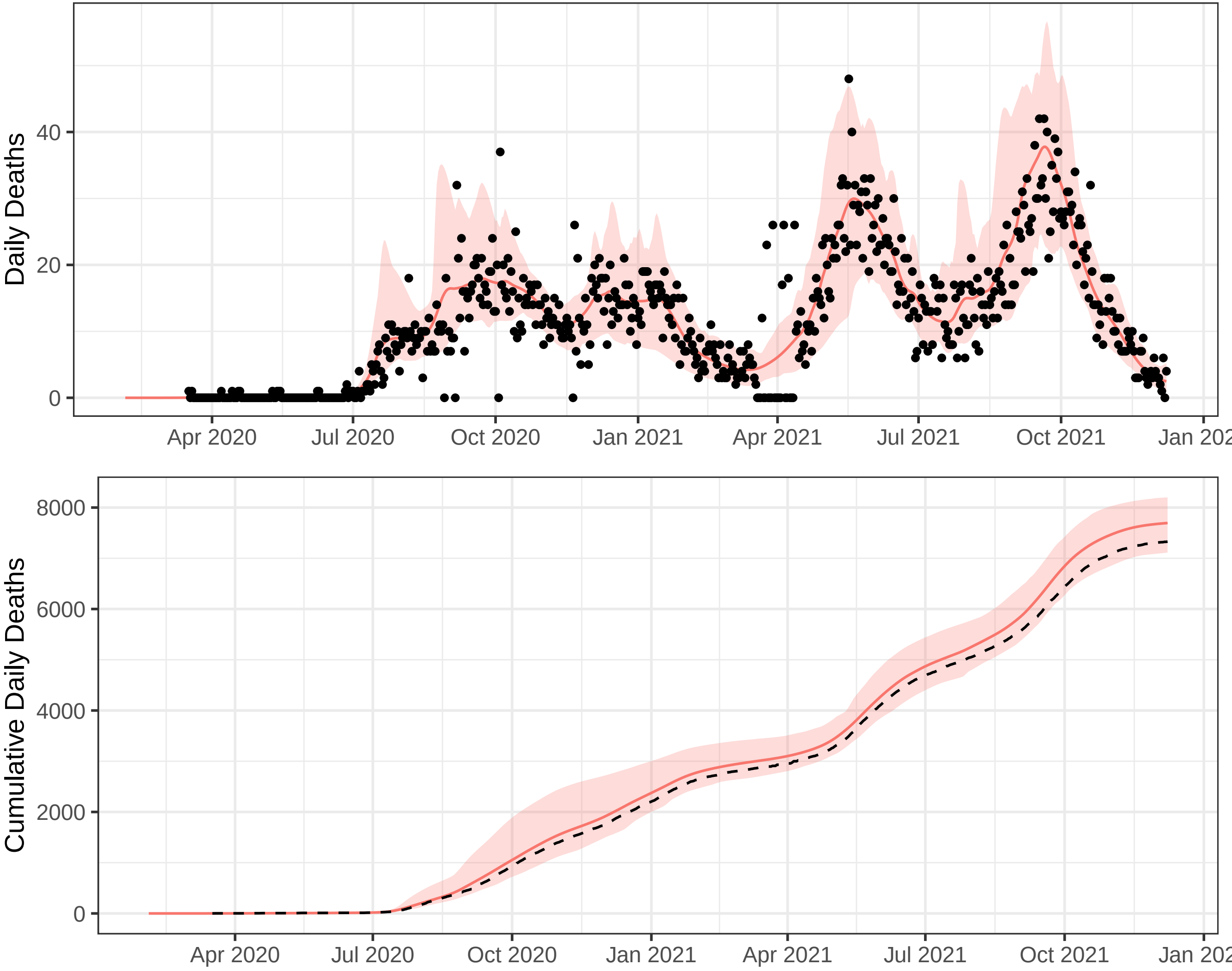

Daily deaths are included with reported COVID−19 deaths shown as points. The red line represents median model estimates of daily deaths, and the shaded region represents the 95% quantiles of the model estimated deaths. Plots of the cumulative estimated deaths are also shown, with the cumulative number of reported deaths represented by a dashed black line.

Cuba

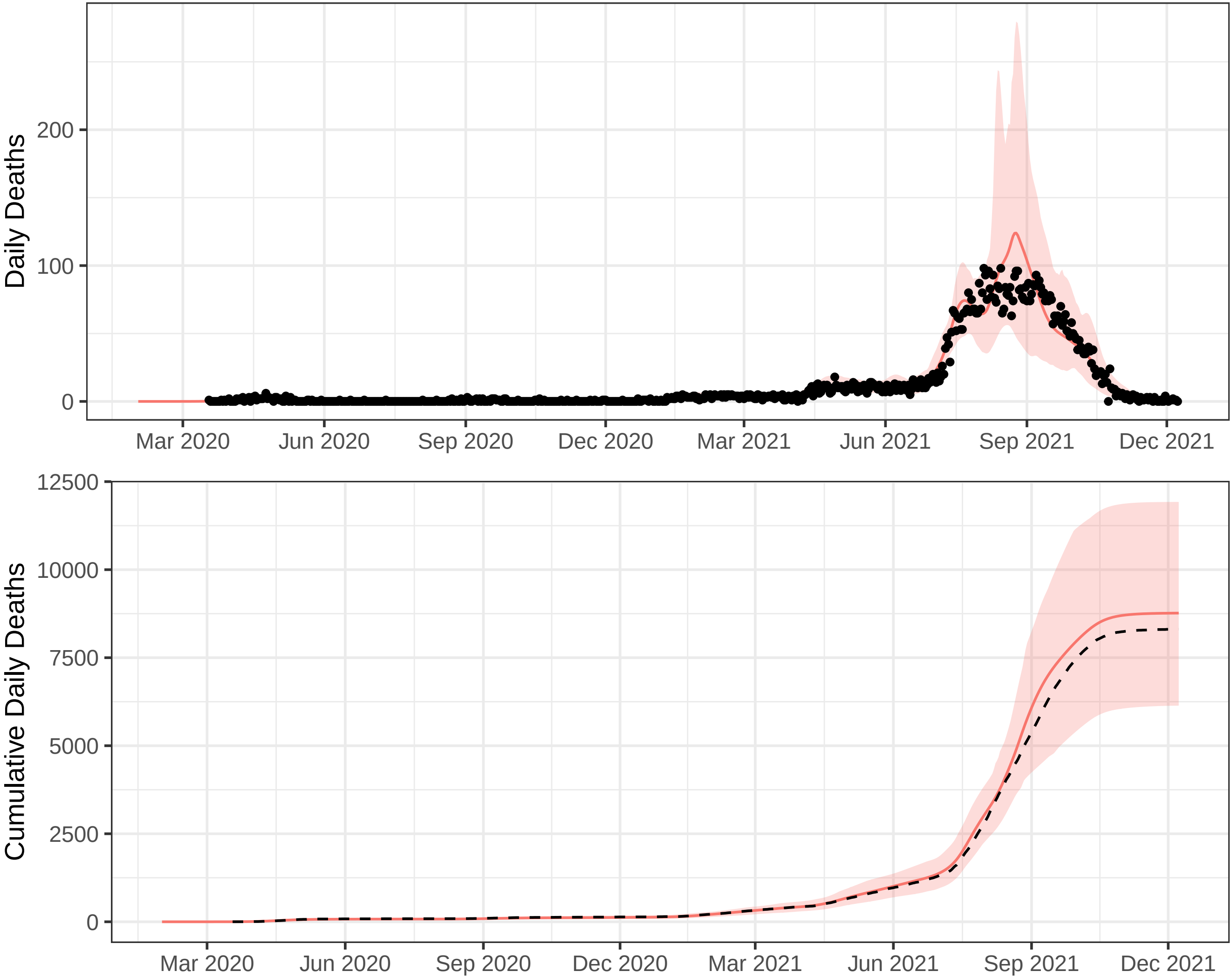

Daily deaths are included with reported COVID−19 deaths shown as points. The red line represents median model estimates of daily deaths, and the shaded region represents the 95% quantiles of the model estimated deaths. Plots of the cumulative estimated deaths are also shown, with the cumulative number of reported deaths represented by a dashed black line.

Cyprus

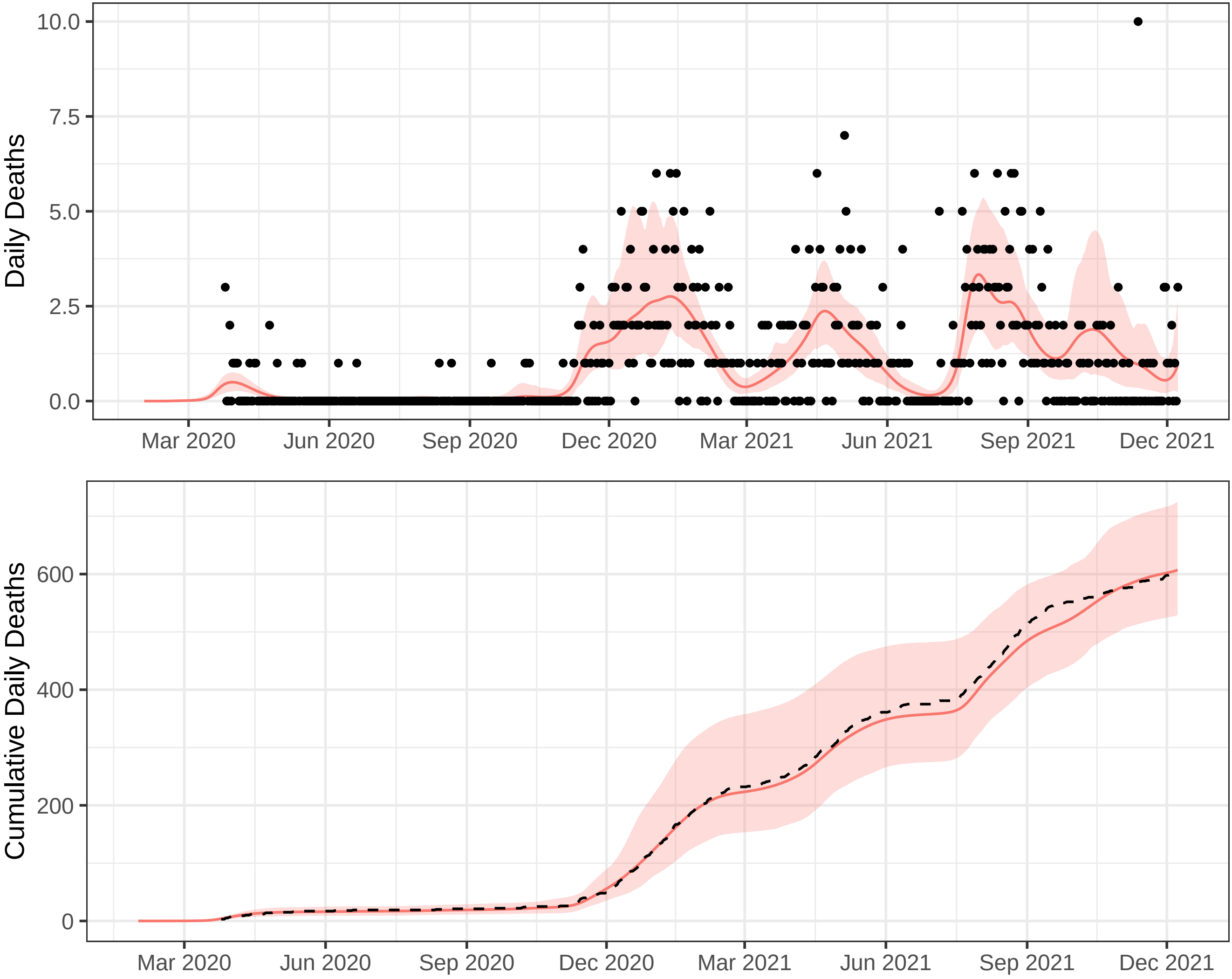

Daily deaths are included with reported COVID−19 deaths shown as points. The red line represents median model estimates of daily deaths, and the shaded region represents the 95% quantiles of the model estimated deaths. Plots of the cumulative estimated deaths are also shown, with the cumulative number of reported deaths represented by a dashed black line.

Czechia

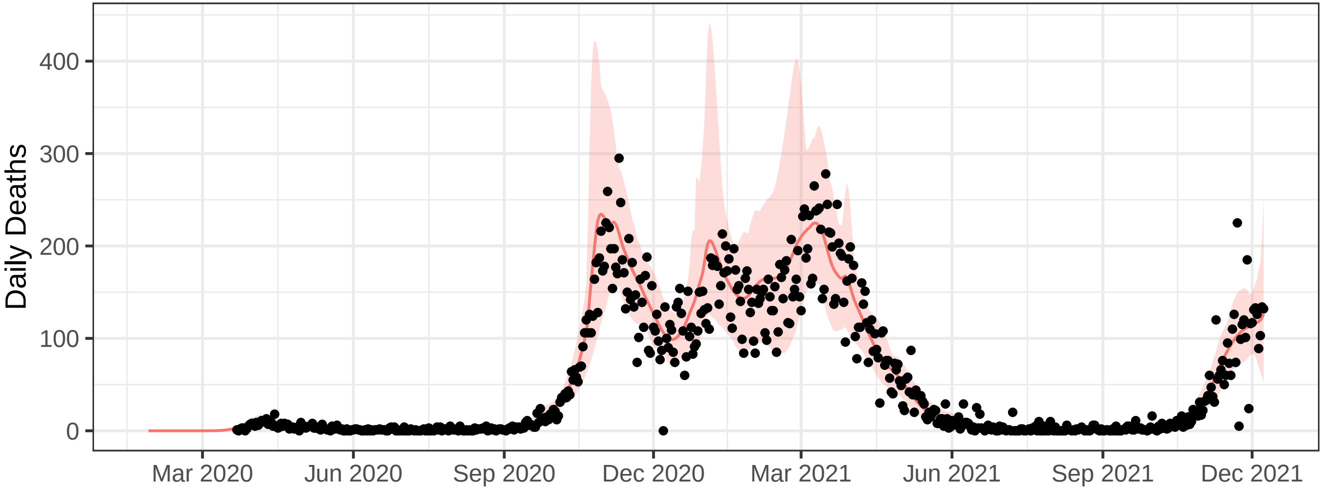

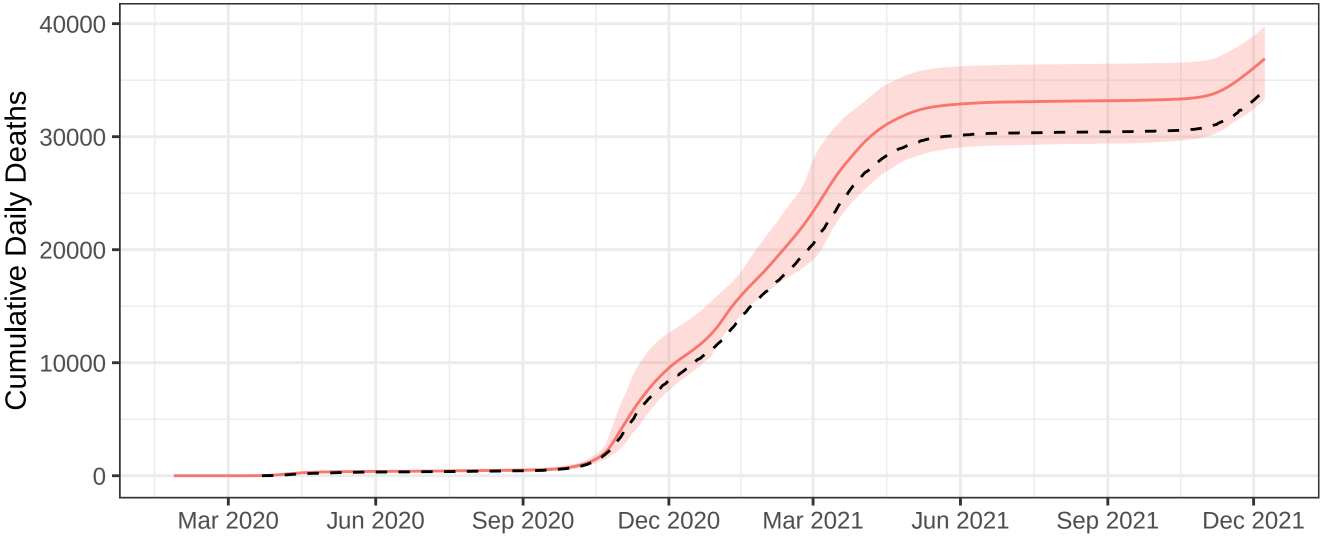

Daily deaths are included with reported COVID−19 deaths shown as points. The red line represents median model estimates of daily deaths, and the shaded region represents the 95% quantiles of the model estimated deaths. Plots of the cumulative estimated deaths are also shown, with the cumulative number of reported deaths represented by a dashed black line.

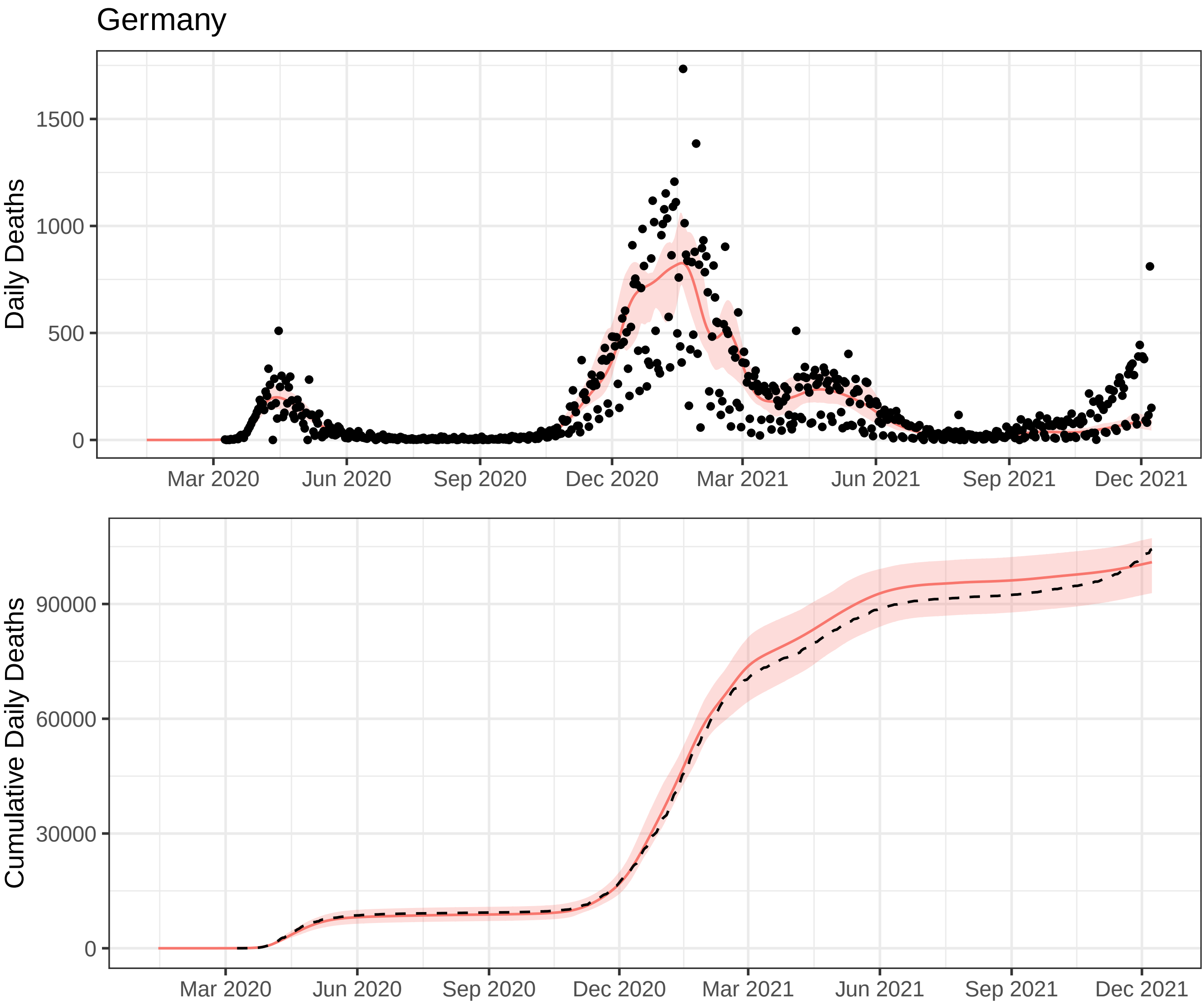

Daily deaths are included with reported COVID−19 deaths shown as points. The red line represents median model estimates of daily deaths, and the shaded region represents the 95% quantiles of the model estimated deaths. Plots of the cumulative estimated deaths are also shown, with the cumulative number of reported deaths represented by a dashed black line.

Djibouti

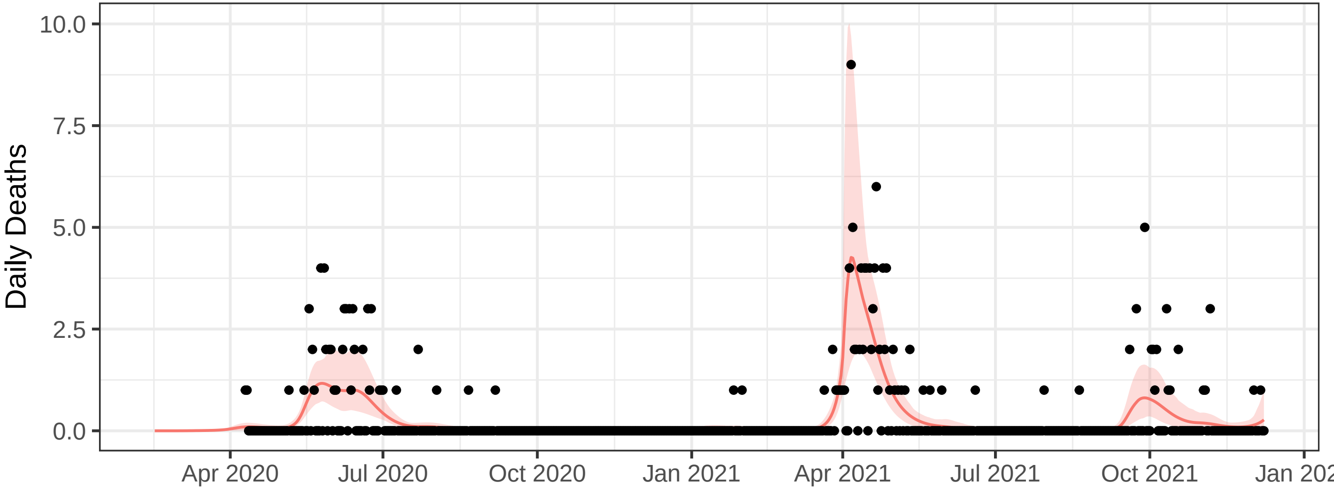

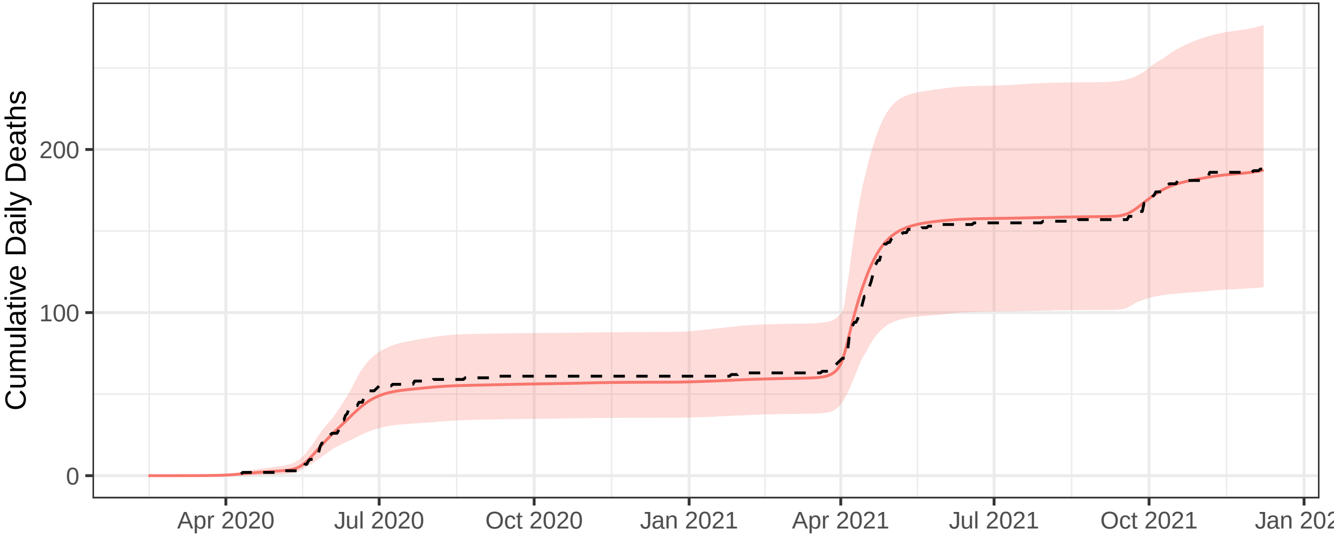

Daily deaths are included with reported COVID−19 deaths shown as points. The red line represents median model estimates of daily deaths, and the shaded region represents the 95% quantiles of the model estimated deaths. Plots of the cumulative estimated deaths are also shown, with the cumulative number of reported deaths represented by a dashed black line.

Denmark

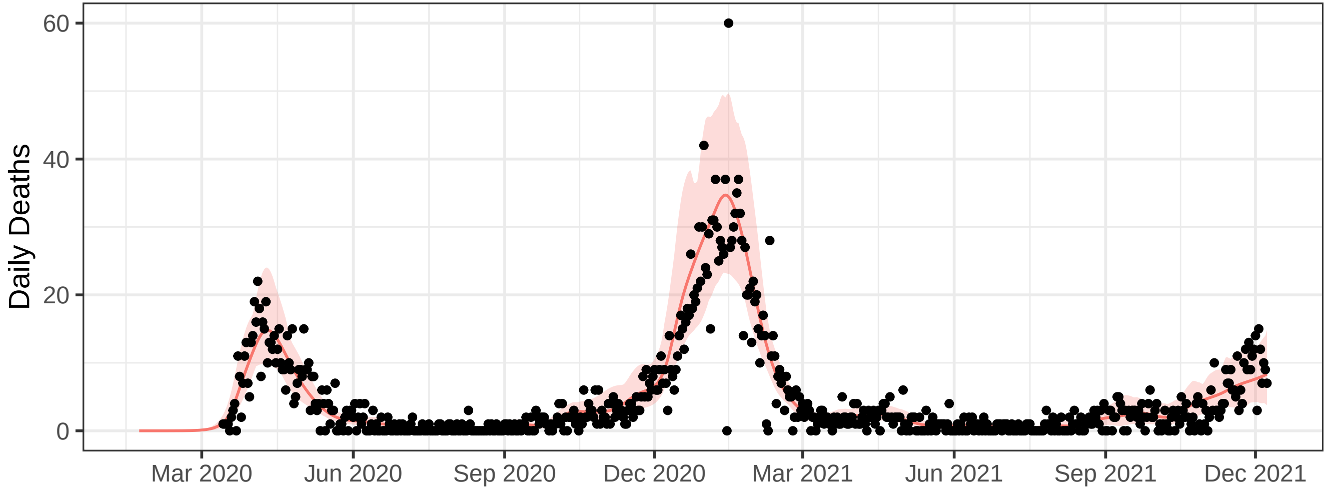

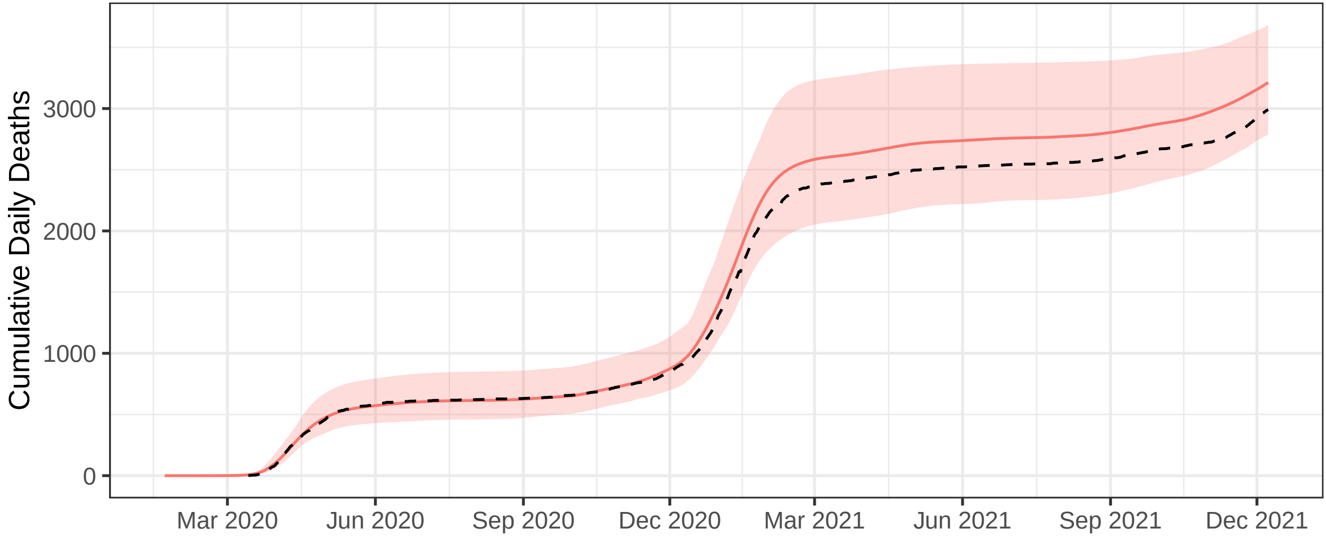

Daily deaths are included with reported COVID−19 deaths shown as points. The red line represents median model estimates of daily deaths, and the shaded region represents the 95% quantiles of the model estimated deaths. Plots of the cumulative estimated deaths are also shown, with the cumulative number of reported deaths represented by a dashed black line.

Dominican Republic

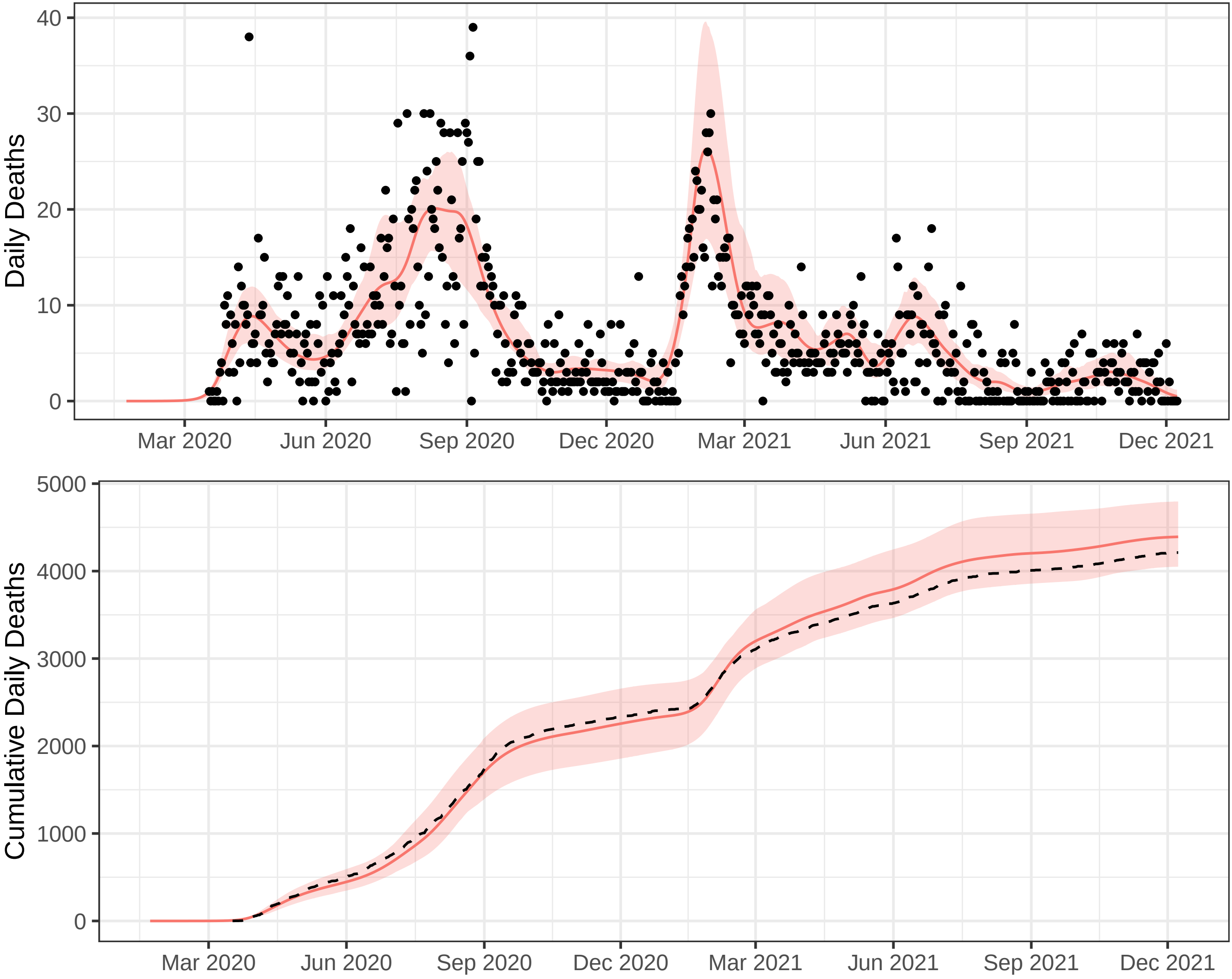

Daily deaths are included with reported COVID−19 deaths shown as points. The red line represents median model estimates of daily deaths, and the shaded region represents the 95% quantiles of the model estimated deaths. Plots of the cumulative estimated deaths are also shown, with the cumulative number of reported deaths represented by a dashed black line.

Algeria

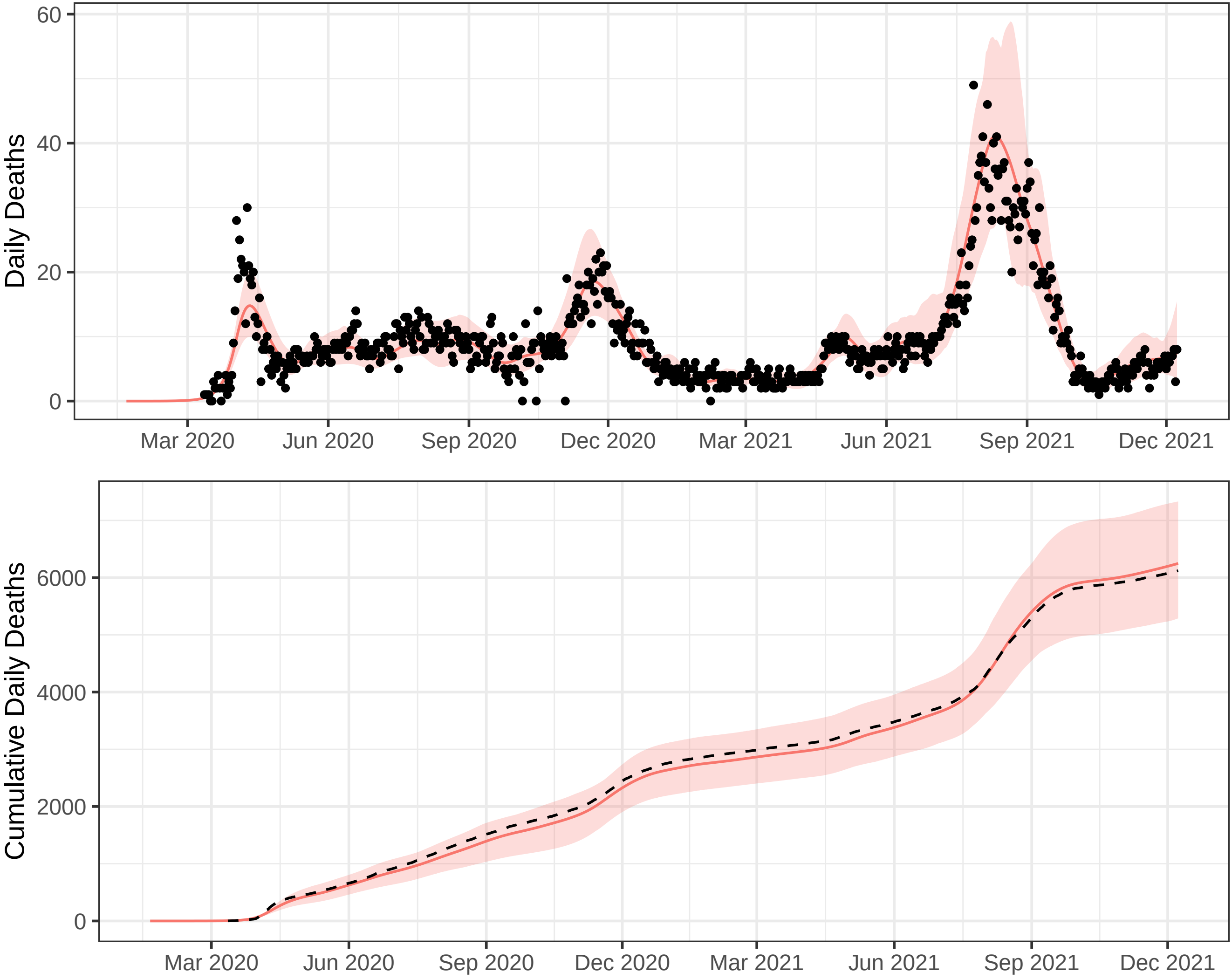

Daily deaths are included with reported COVID−19 deaths shown as points. The red line represents median model estimates of daily deaths, and the shaded region represents the 95% quantiles of the model estimated deaths. Plots of the cumulative estimated deaths are also shown, with the cumulative number of reported deaths represented by a dashed black line.

Ecuador

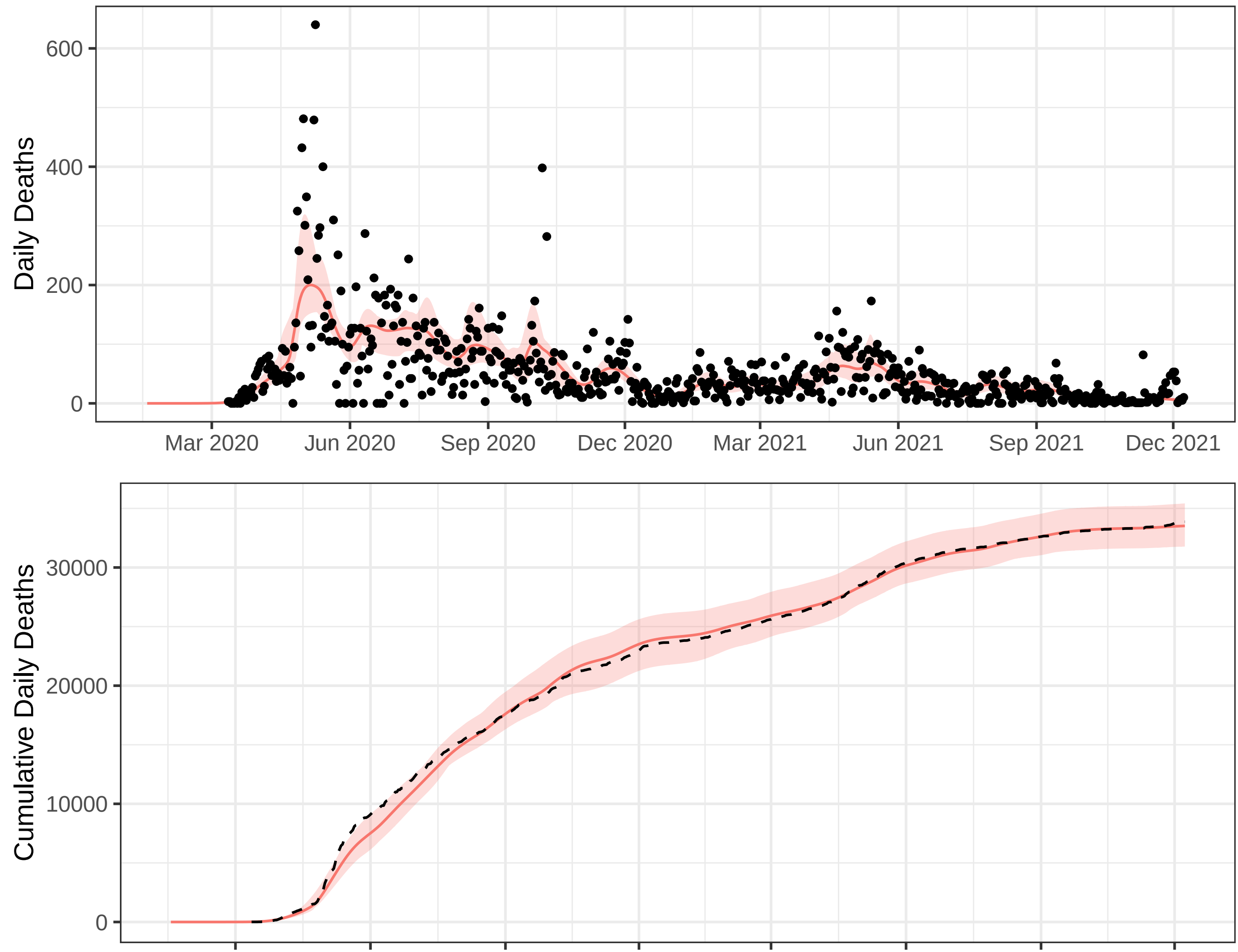

Daily deaths are included with reported COVID−19 deaths shown as points. The red line represents median model estimates of daily deaths, and the shaded region represents the 95% quantiles of the model estimated deaths. Plots of the cumulative estimated deaths are also shown, with the cumulative number of reported deaths represented by a dashed black line.

Egypt

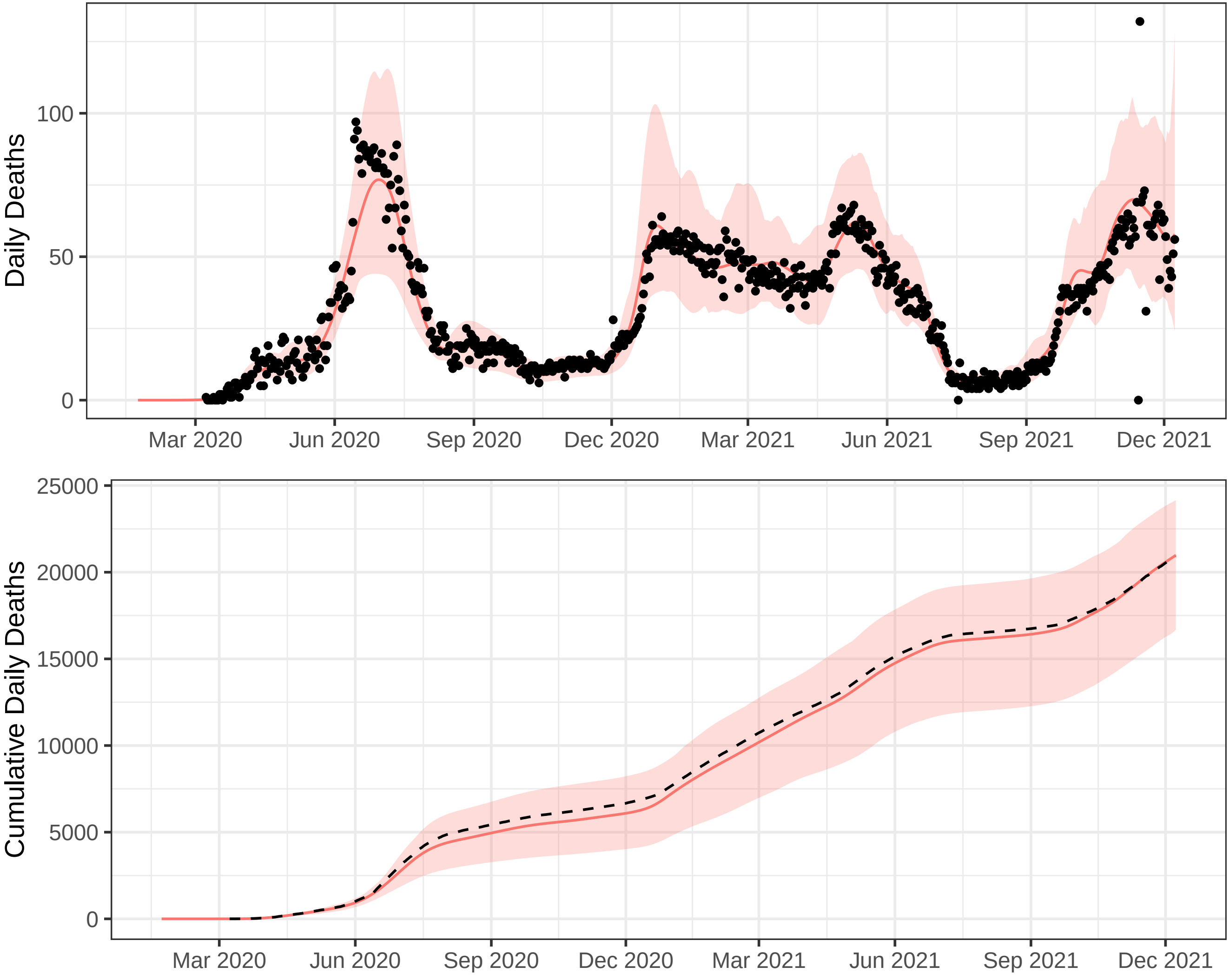

Daily deaths are included with reported COVID−19 deaths shown as points. The red line represents median model estimates of daily deaths, and the shaded region represents the 95% quantiles of the model estimated deaths. Plots of the cumulative estimated deaths are also shown, with the cumulative number of reported deaths represented by a dashed black line.

Eritrea

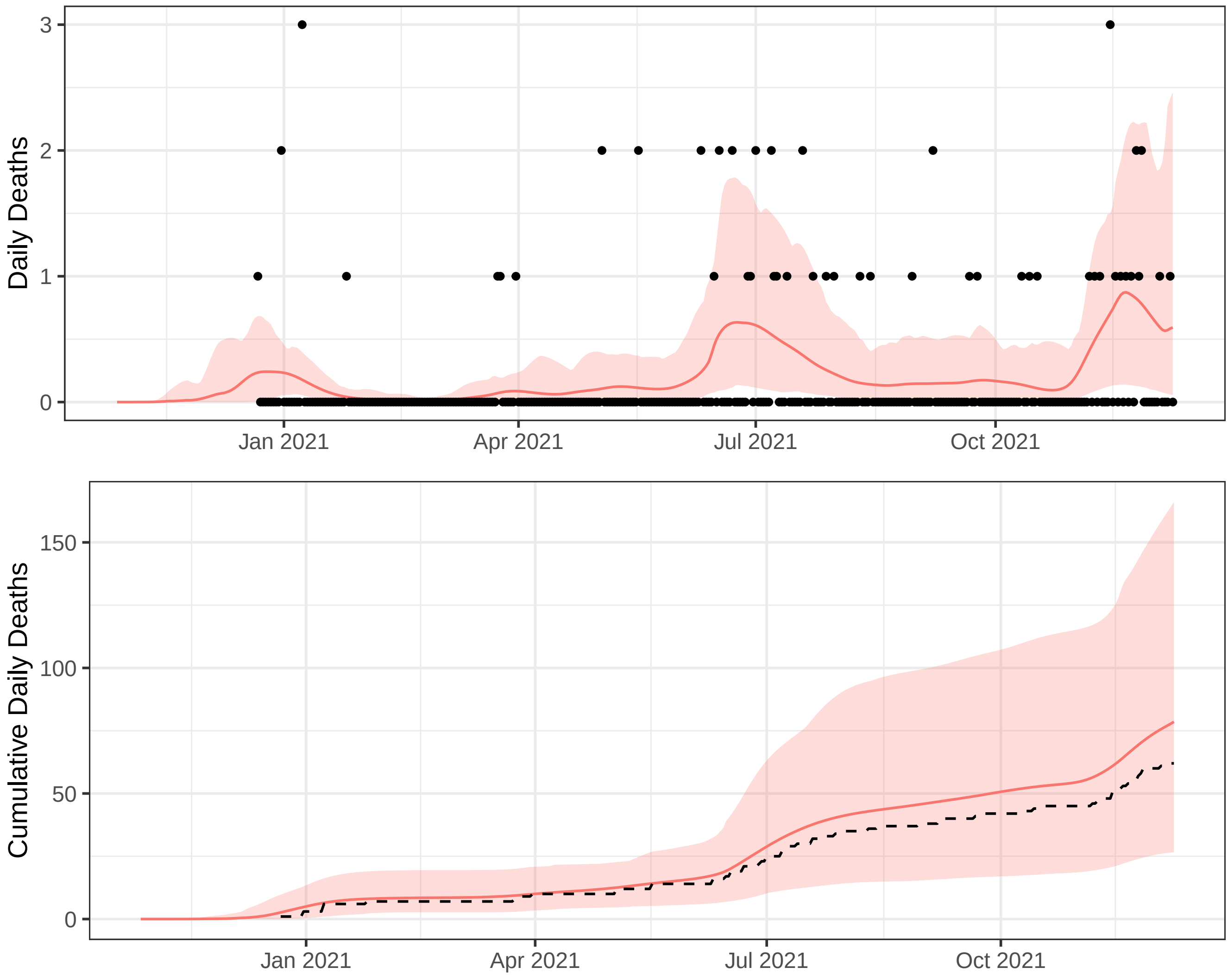

Daily deaths are included with reported COVID−19 deaths shown as points. The red line represents median model estimates of daily deaths, and the shaded region represents the 95% quantiles of the model estimated deaths. Plots of the cumulative estimated deaths are also shown, with the cumulative number of reported deaths represented by a dashed black line.

Spain

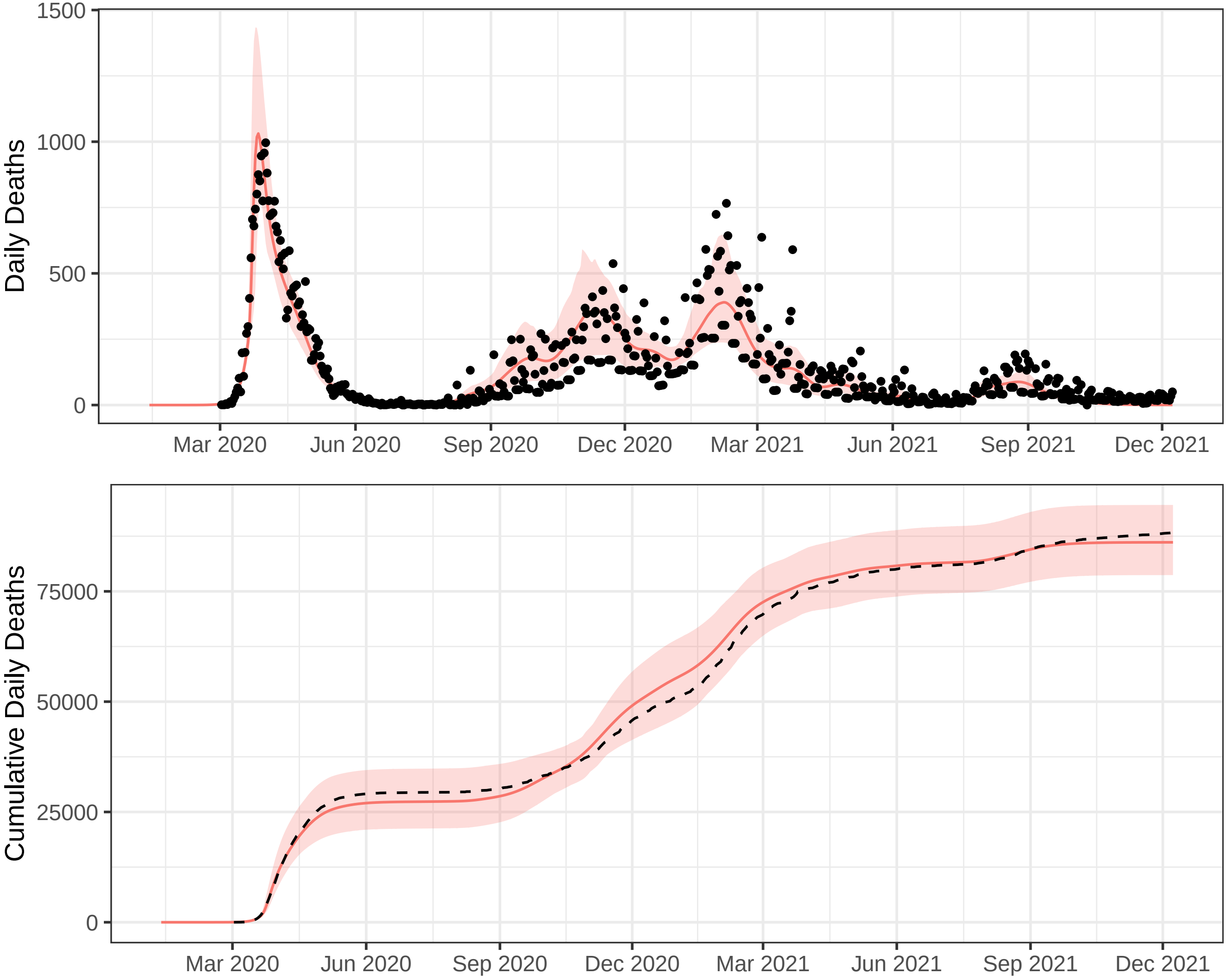

Daily deaths are included with reported COVID−19 deaths shown as points. The red line represents median model estimates of daily deaths, and the shaded region represents the 95% quantiles of the model estimated deaths. Plots of the cumulative estimated deaths are also shown, with the cumulative number of reported deaths represented by a dashed black line.

Estonia

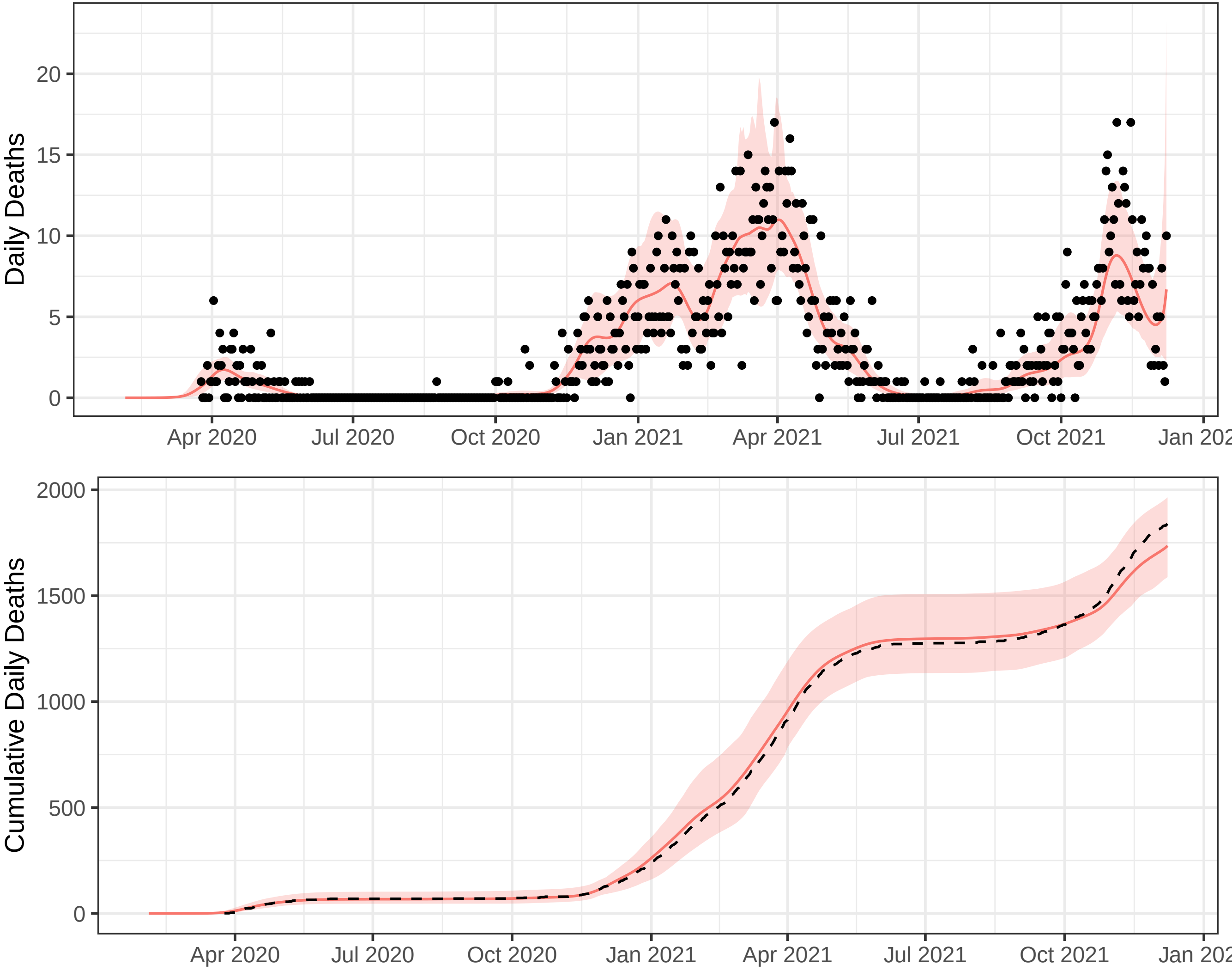

Daily deaths are included with reported COVID−19 deaths shown as points. The red line represents median model estimates of daily deaths, and the shaded region represents the 95% quantiles of the model estimated deaths. Plots of the cumulative estimated deaths are also shown, with the cumulative number of reported deaths represented by a dashed black line.

Ethiopia

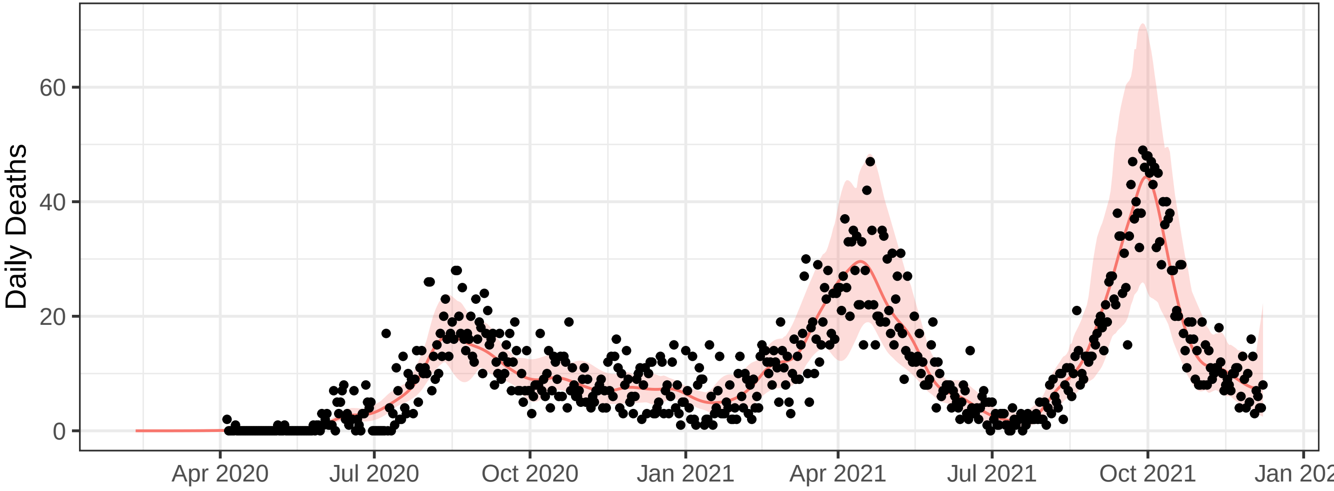

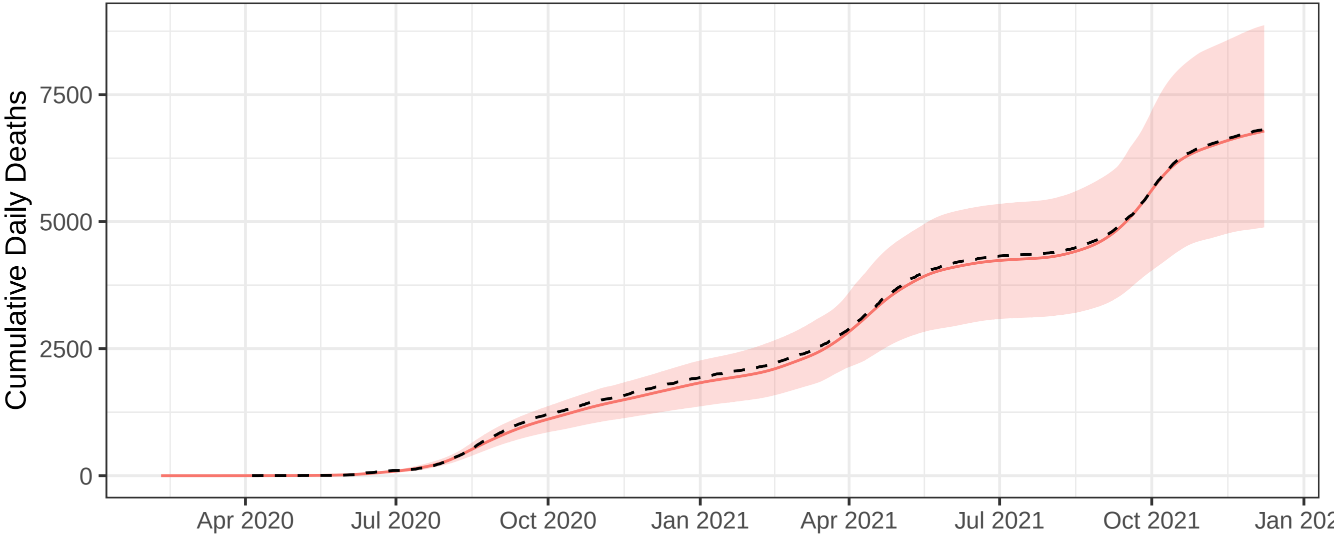

Daily deaths are included with reported COVID−19 deaths shown as points. The red line represents median model estimates of daily deaths, and the shaded region represents the 95% quantiles of the model estimated deaths. Plots of the cumulative estimated deaths are also shown, with the cumulative number of reported deaths represented by a dashed black line.

Finland

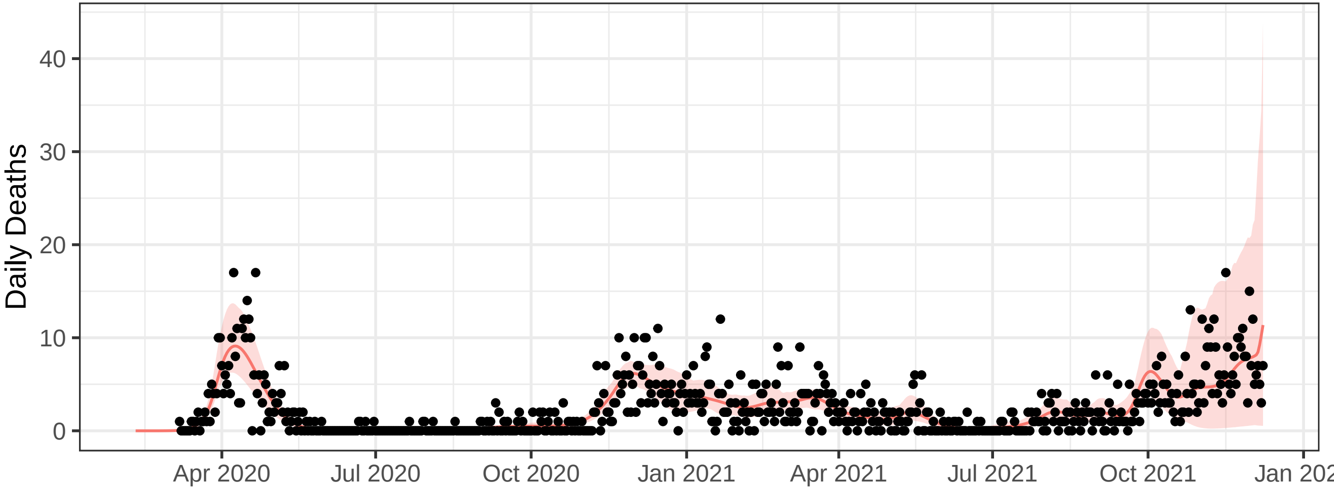

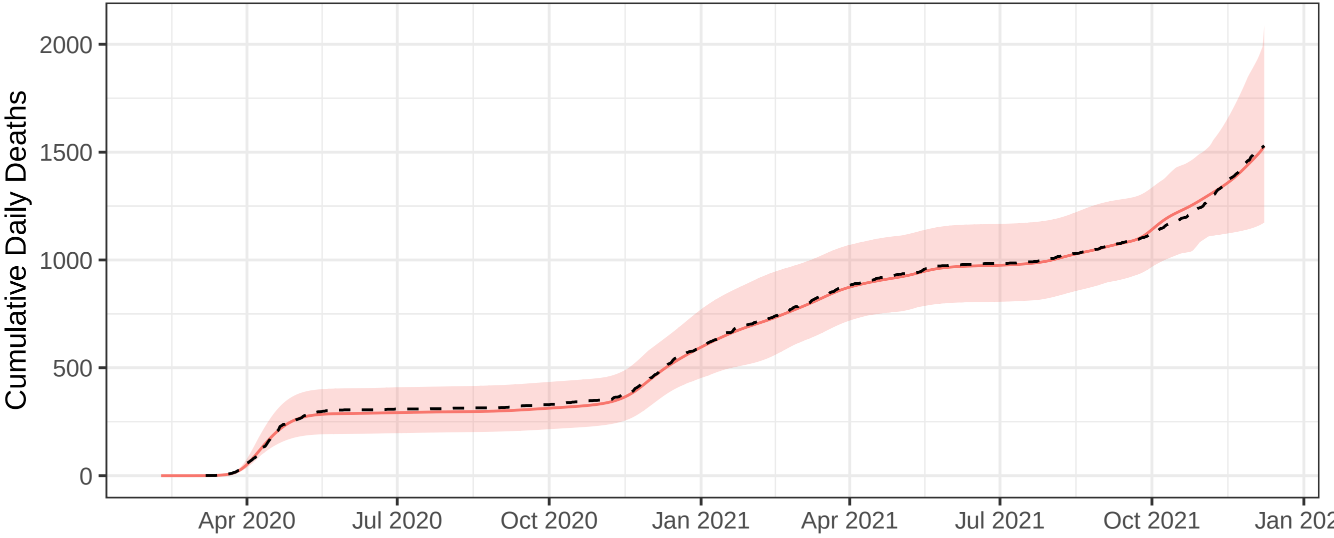

Daily deaths are included with reported COVID−19 deaths shown as points. The red line represents median model estimates of daily deaths, and the shaded region represents the 95% quantiles of the model estimated deaths. Plots of the cumulative estimated deaths are also shown, with the cumulative number of reported deaths represented by a dashed black line.

Fiji

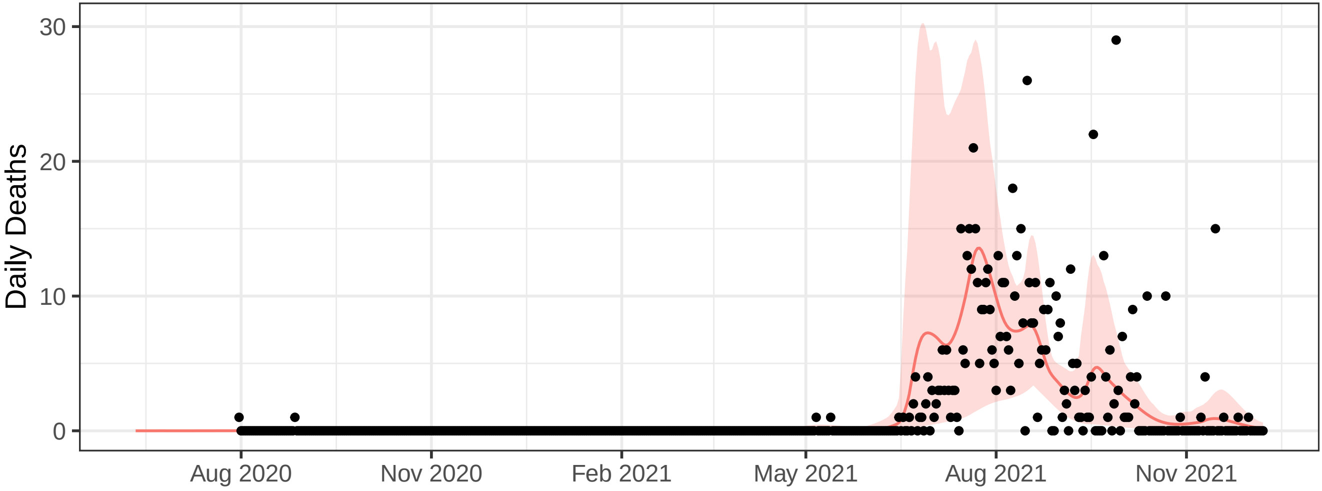

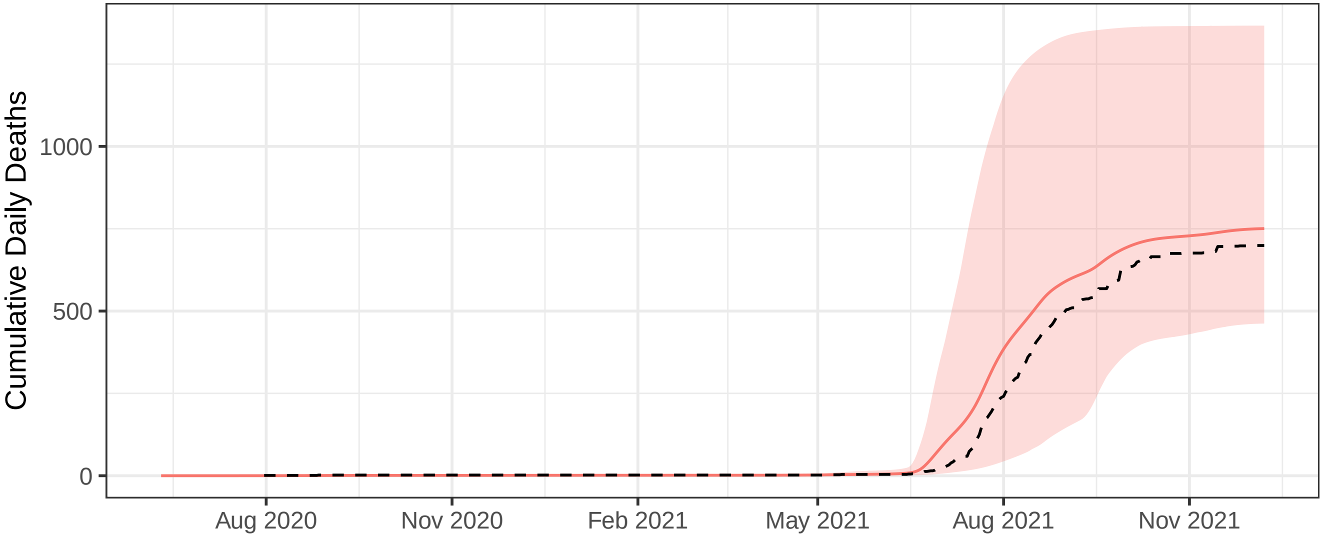

Daily deaths are included with reported COVID−19 deaths shown as points. The red line represents median model estimates of daily deaths, and the shaded region represents the 95% quantiles of the model estimated deaths. Plots of the cumulative estimated deaths are also shown, with the cumulative number of reported deaths represented by a dashed black line.

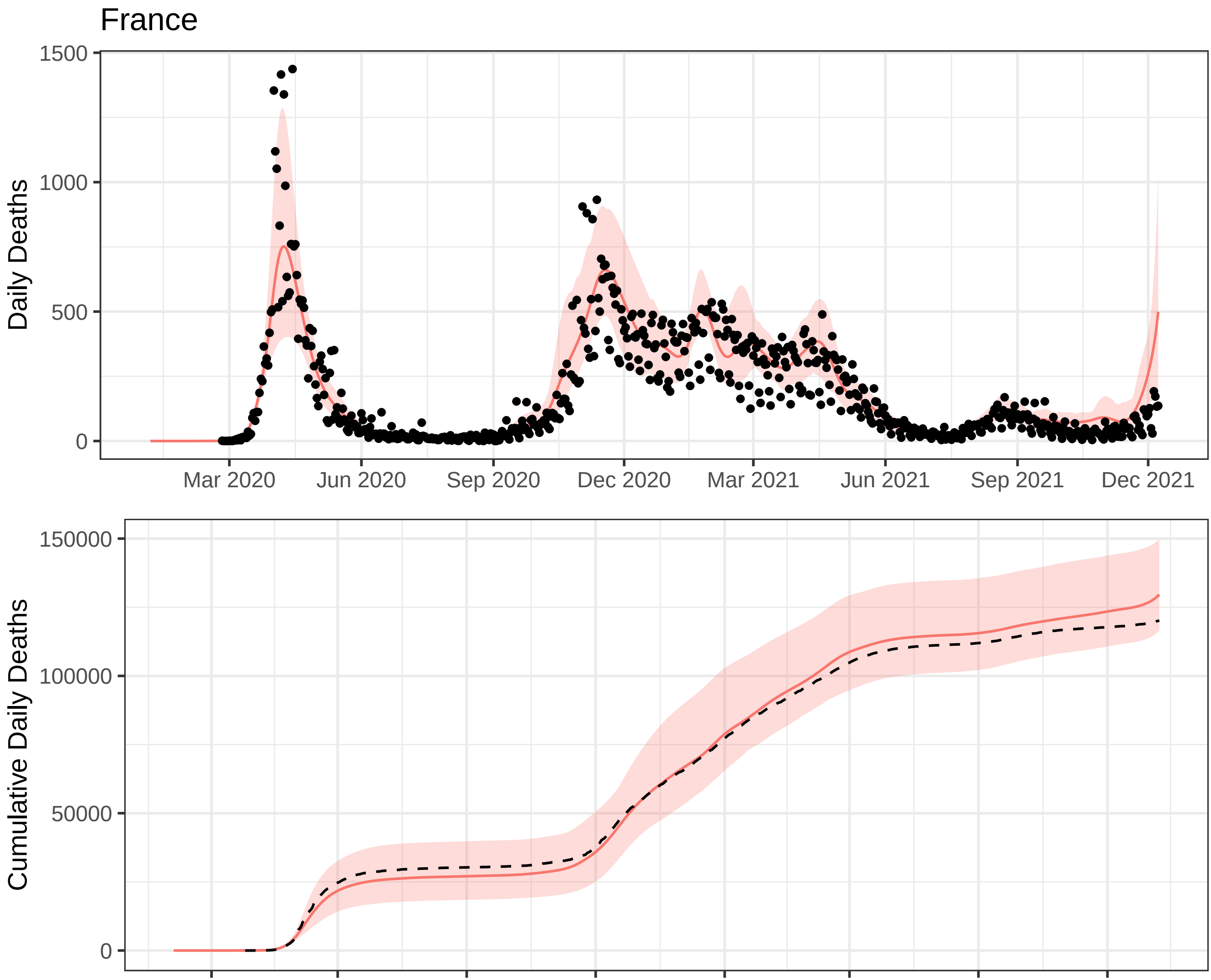

Daily deaths are included with reported COVID−19 deaths shown as points. The red line represents median model estimates of daily deaths, and the shaded region represents the 95% quantiles of the model estimated deaths. Plots of the cumulative estimated deaths are also shown, with the cumulative number of reported deaths represented by a dashed black line.

Gabon

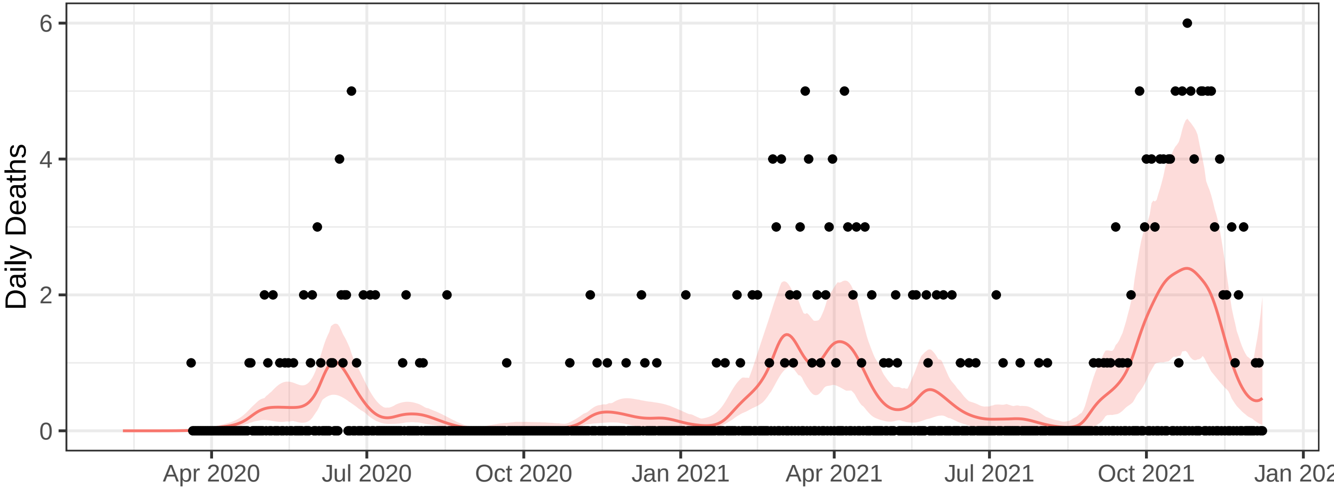

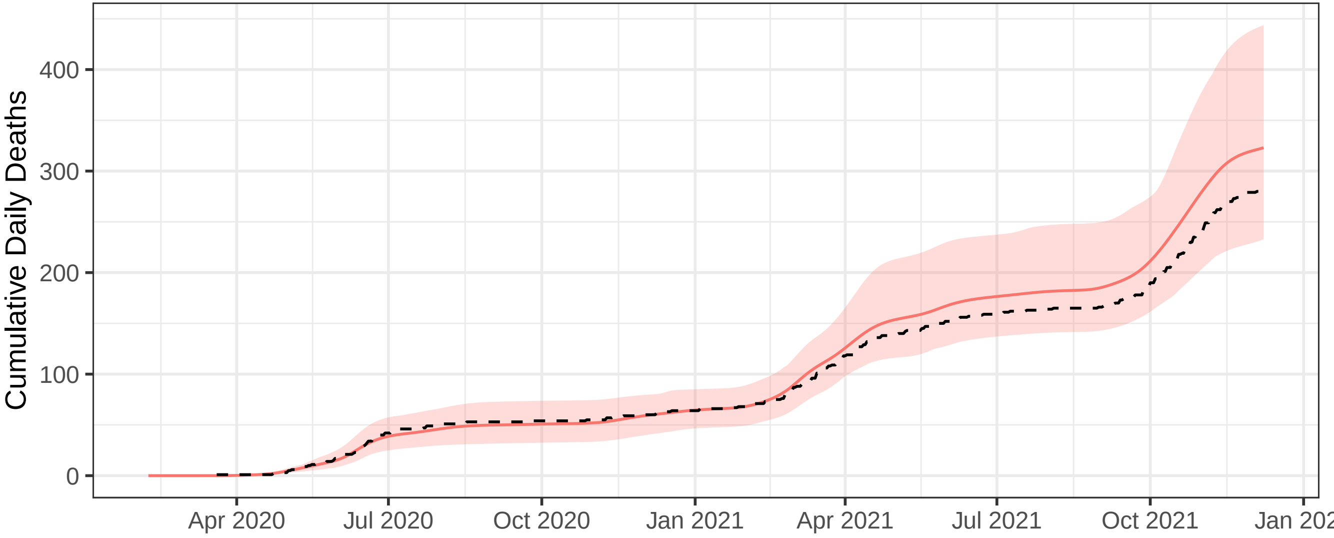

Daily deaths are included with reported COVID−19 deaths shown as points. The red line represents median model estimates of daily deaths, and the shaded region represents the 95% quantiles of the model estimated deaths. Plots of the cumulative estimated deaths are also shown, with the cumulative number of reported deaths represented by a dashed black line.

United Kingdom

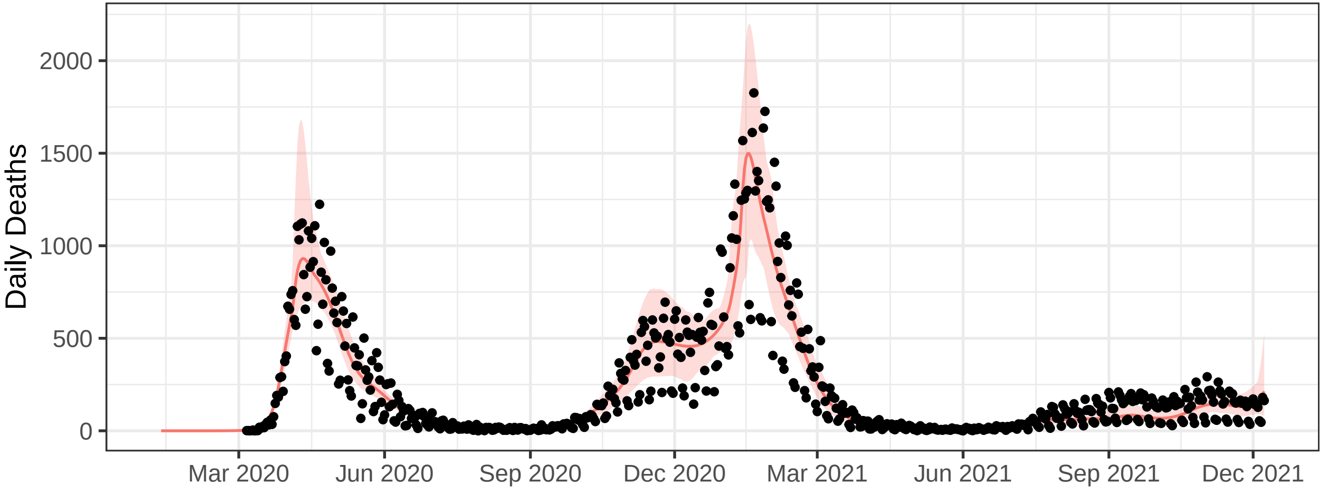

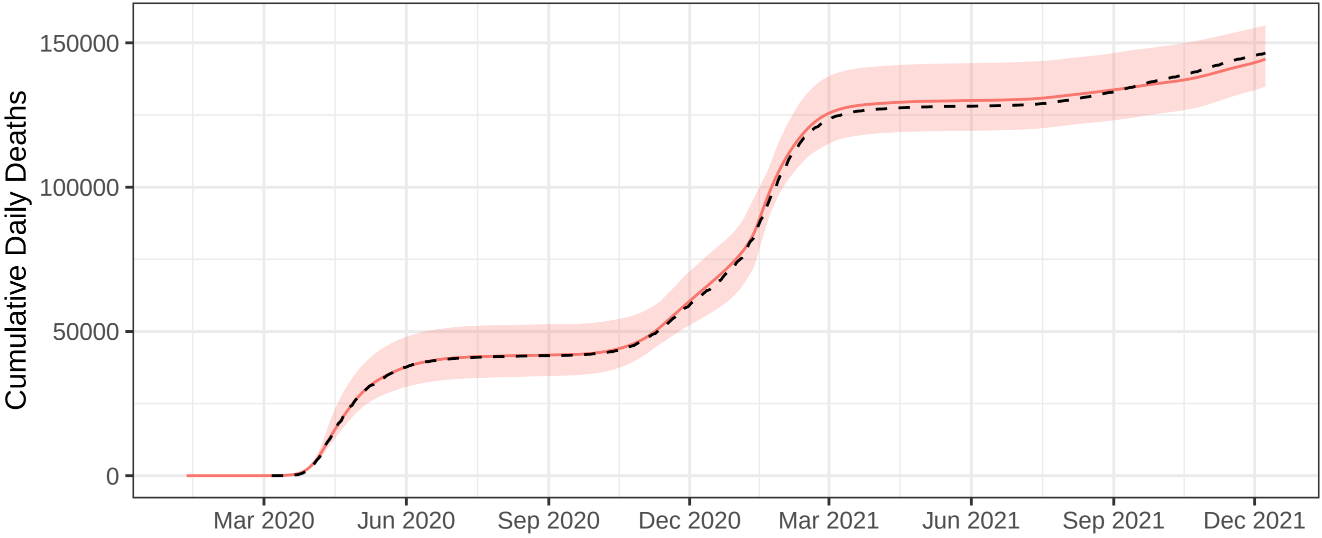

Daily deaths are included with reported COVID−19 deaths shown as points. The red line represents median model estimates of daily deaths, and the shaded region represents the 95% quantiles of the model estimated deaths. Plots of the cumulative estimated deaths are also shown, with the cumulative number of reported deaths represented by a dashed black line.

Georgia

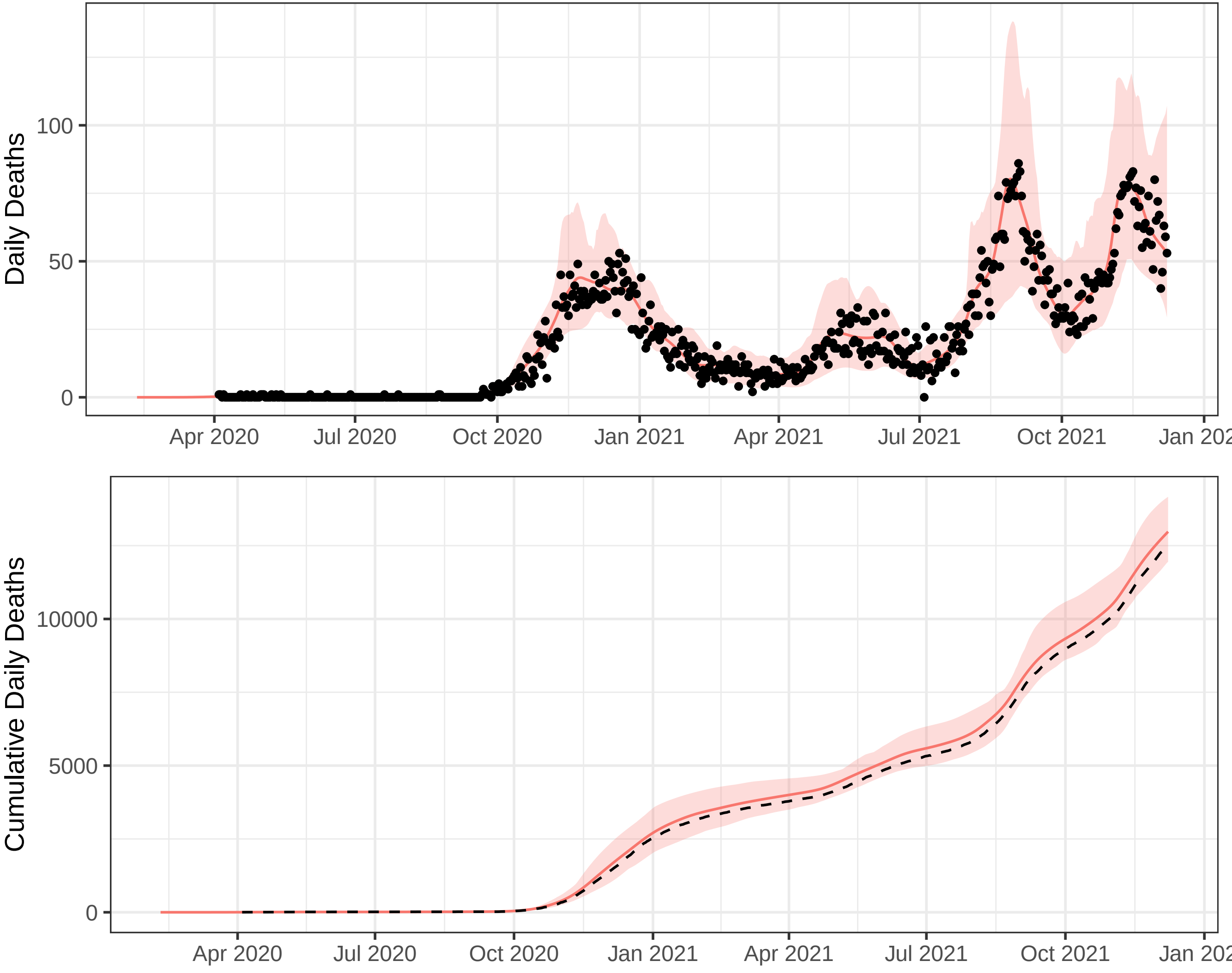

Daily deaths are included with reported COVID−19 deaths shown as points. The red line represents median model estimates of daily deaths, and the shaded region represents the 95% quantiles of the model estimated deaths. Plots of the cumulative estimated deaths are also shown, with the cumulative number of reported deaths represented by a dashed black line.

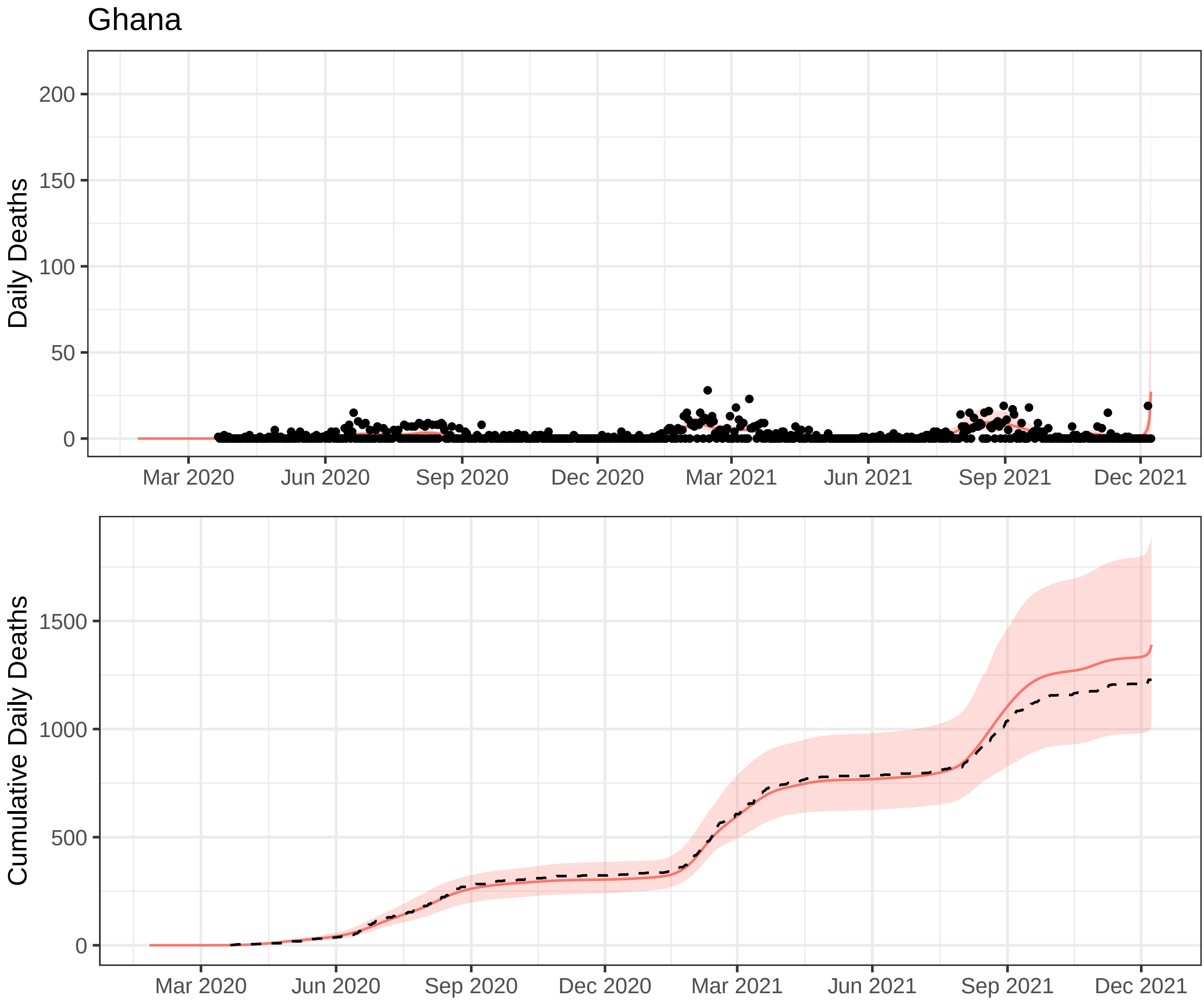

Daily deaths are included with reported COVID−19 deaths shown as points. The red line represents median model estimates of daily deaths, and the shaded region represents the 95% quantiles of the model estimated deaths. Plots of the cumulative estimated deaths are also shown, with the cumulative number of reported deaths represented by a dashed black line.

Guinea

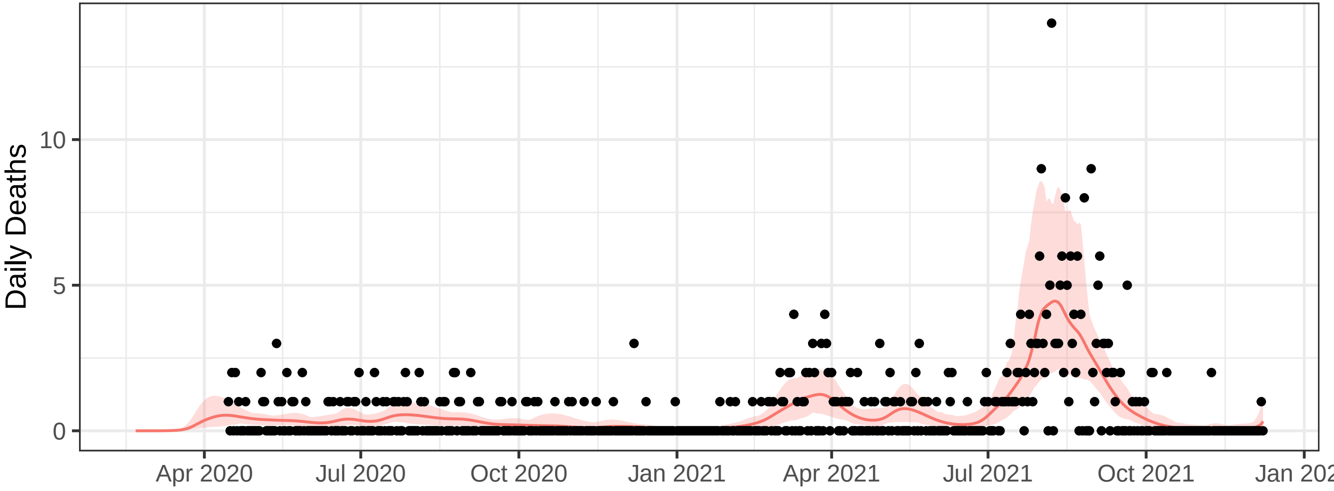

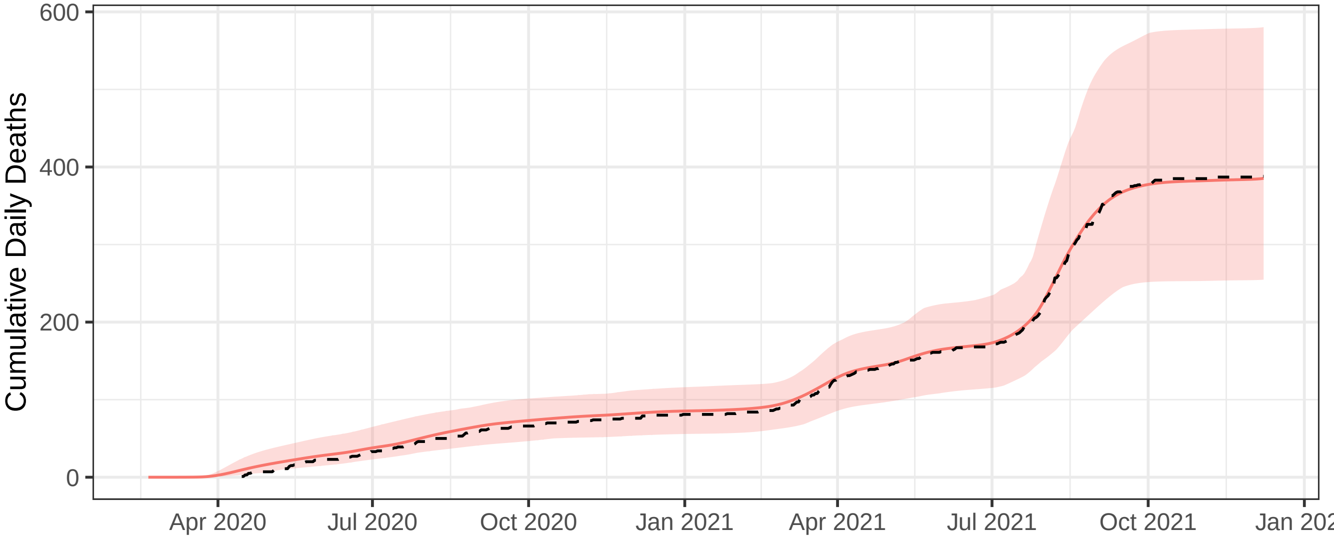

Daily deaths are included with reported COVID−19 deaths shown as points. The red line represents median model estimates of daily deaths, and the shaded region represents the 95% quantiles of the model estimated deaths. Plots of the cumulative estimated deaths are also shown, with the cumulative number of reported deaths represented by a dashed black line.

Gambia

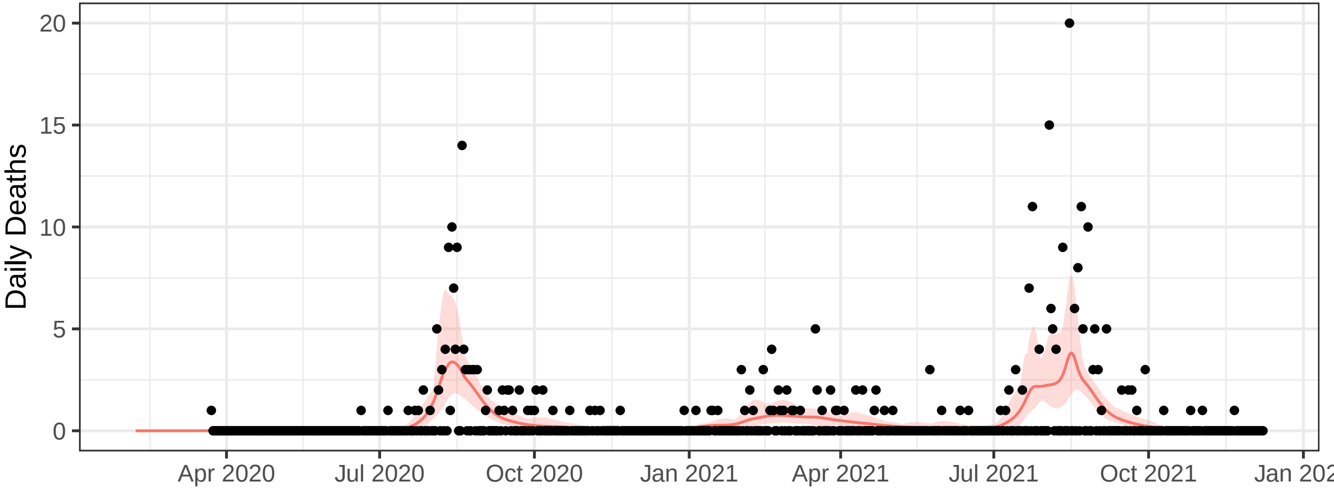

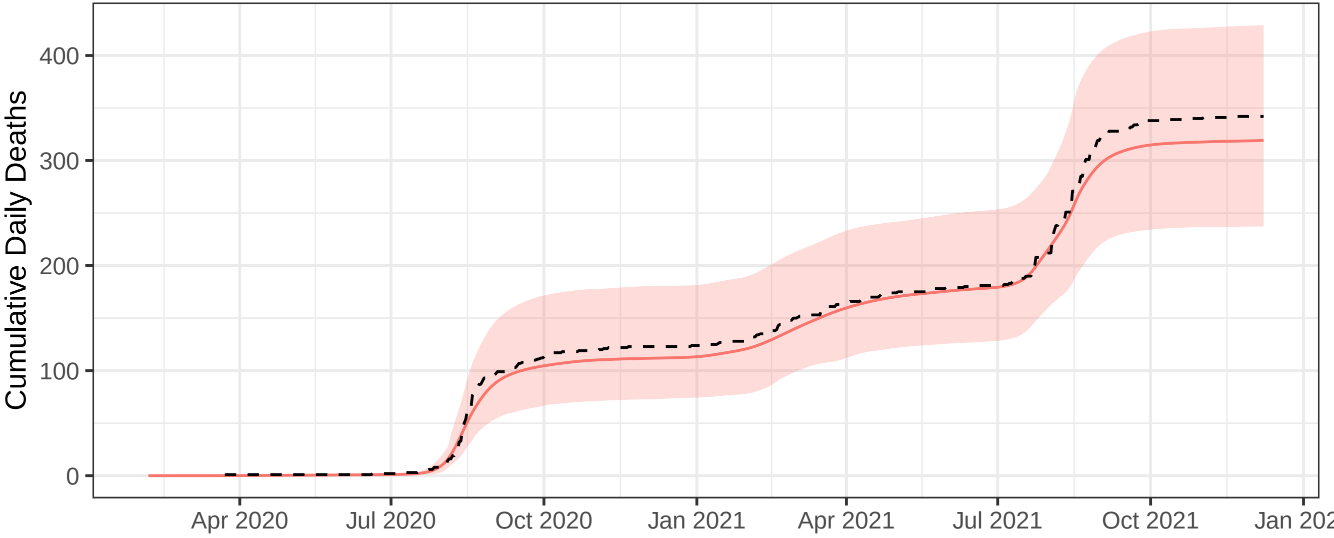

Daily deaths are included with reported COVID−19 deaths shown as points. The red line represents median model estimates of daily deaths, and the shaded region represents the 95% quantiles of the model estimated deaths. Plots of the cumulative estimated deaths are also shown, with the cumulative number of reported deaths represented by a dashed black line.

Guinea−Bissau

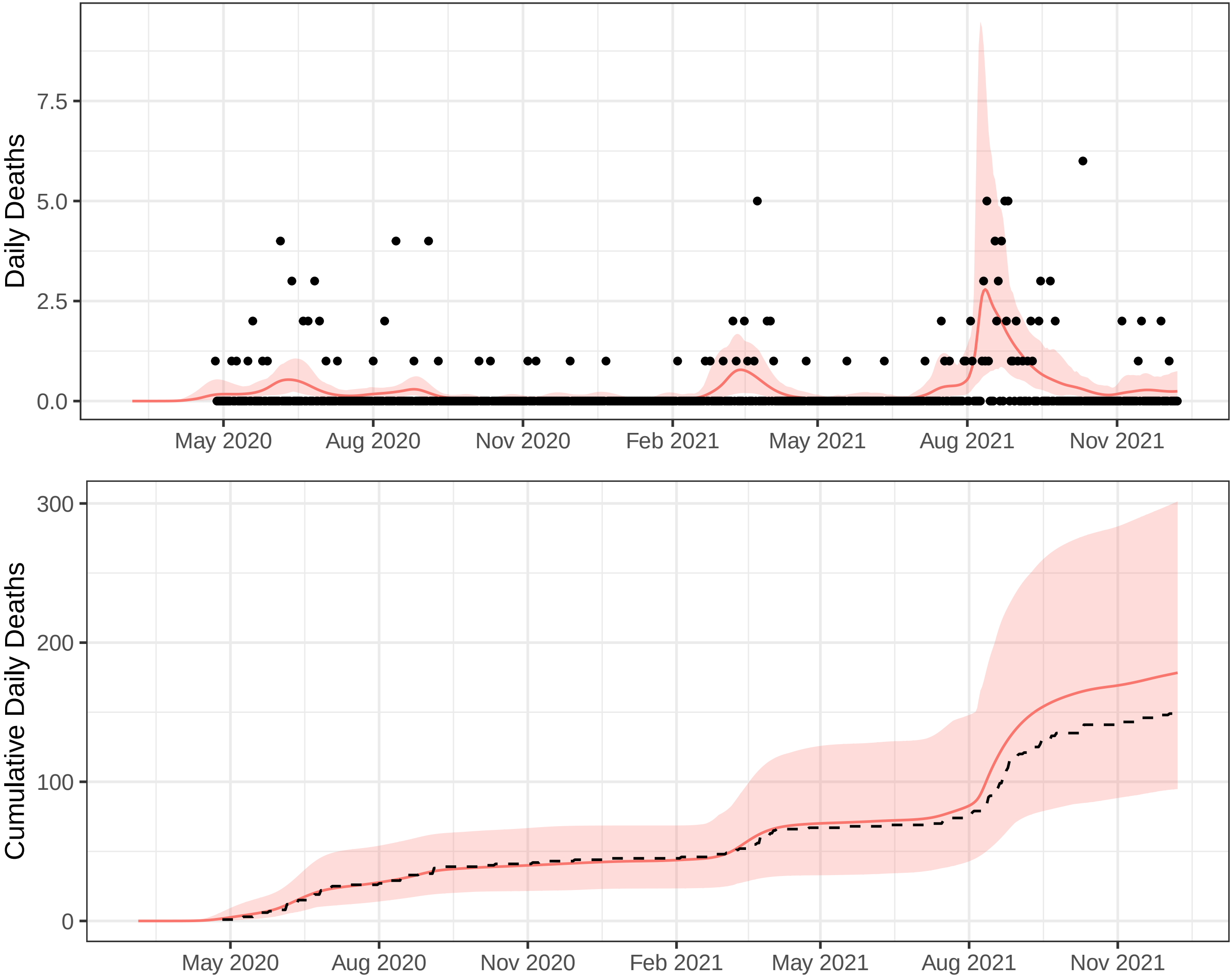

Daily deaths are included with reported COVID−19 deaths shown as points. The red line represents median model estimates of daily deaths, and the shaded region represents the 95% quantiles of the model estimated deaths. Plots of the cumulative estimated deaths are also shown, with the cumulative number of reported deaths represented by a dashed black line.

Equatorial Guinea

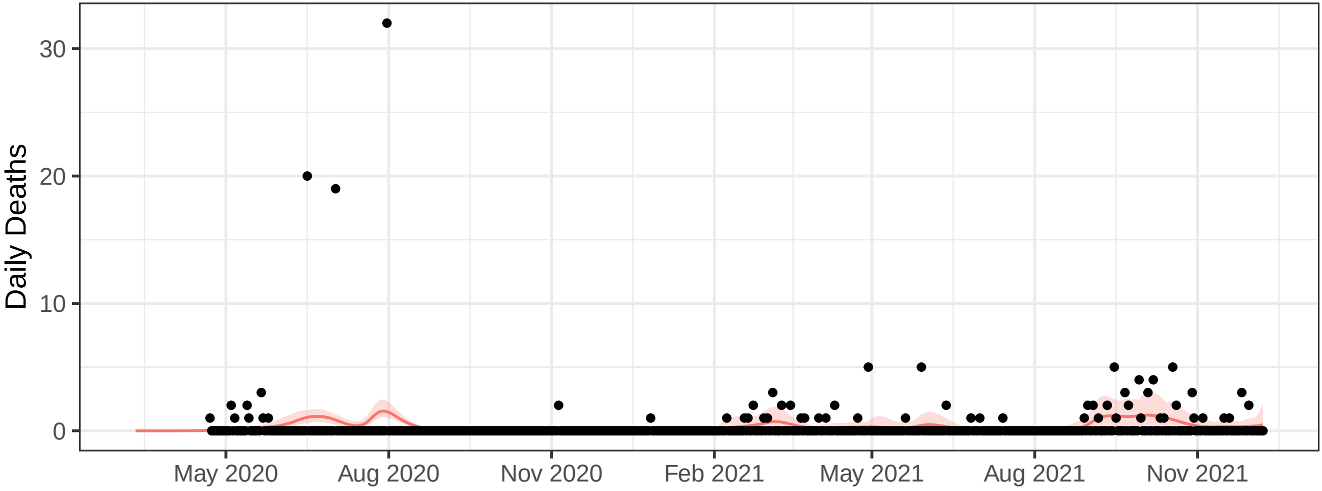

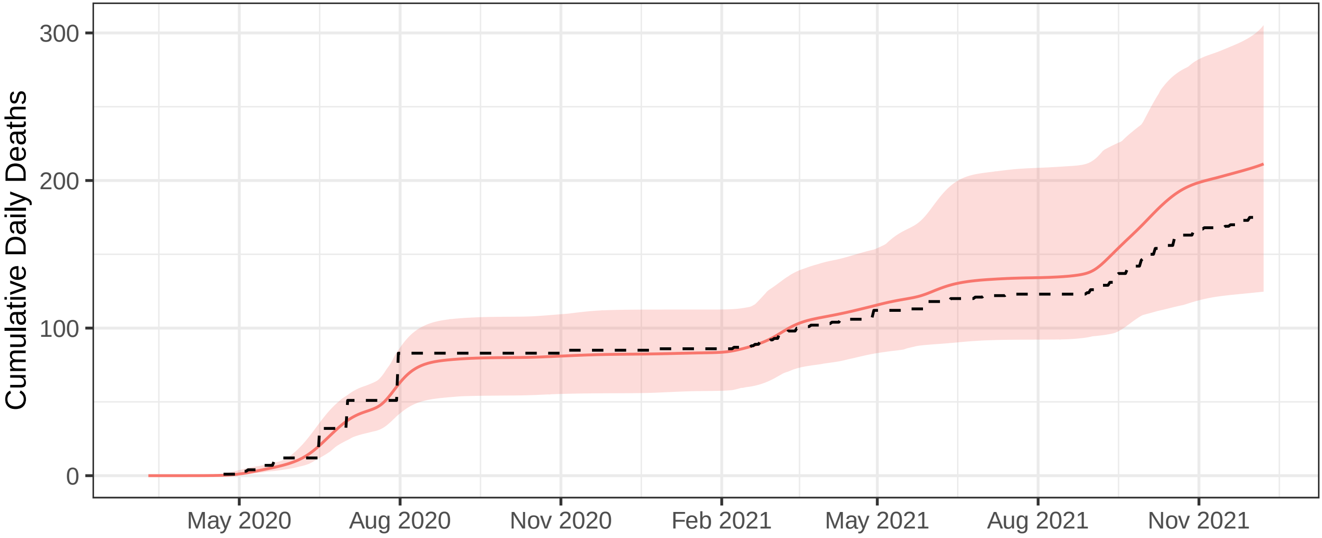

Daily deaths are included with reported COVID−19 deaths shown as points. The red line represents median model estimates of daily deaths, and the shaded region represents the 95% quantiles of the model estimated deaths. Plots of the cumulative estimated deaths are also shown, with the cumulative number of reported deaths represented by a dashed black line.

Greece

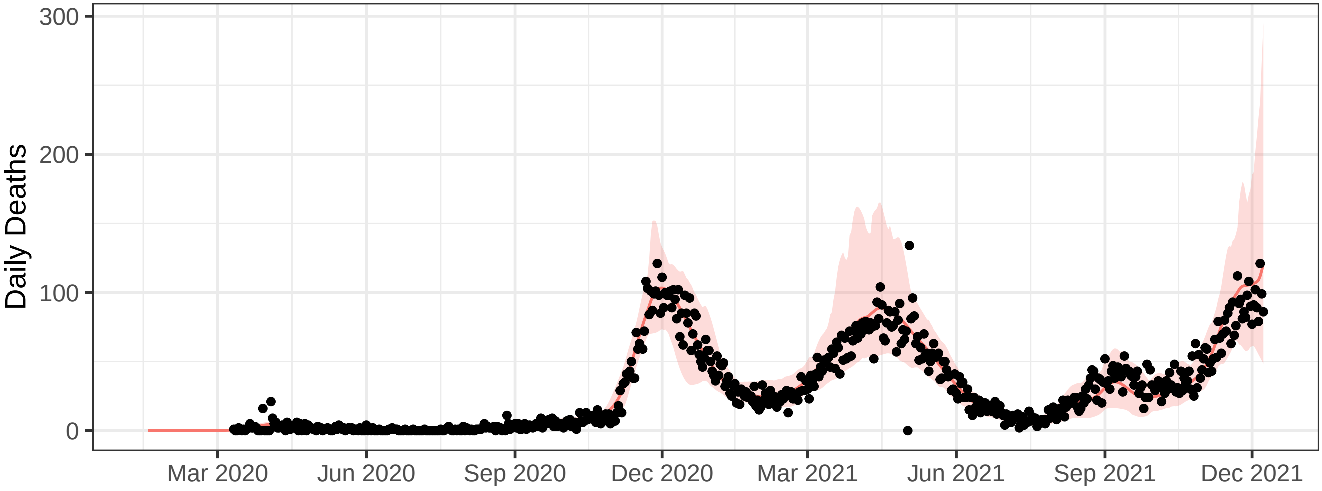

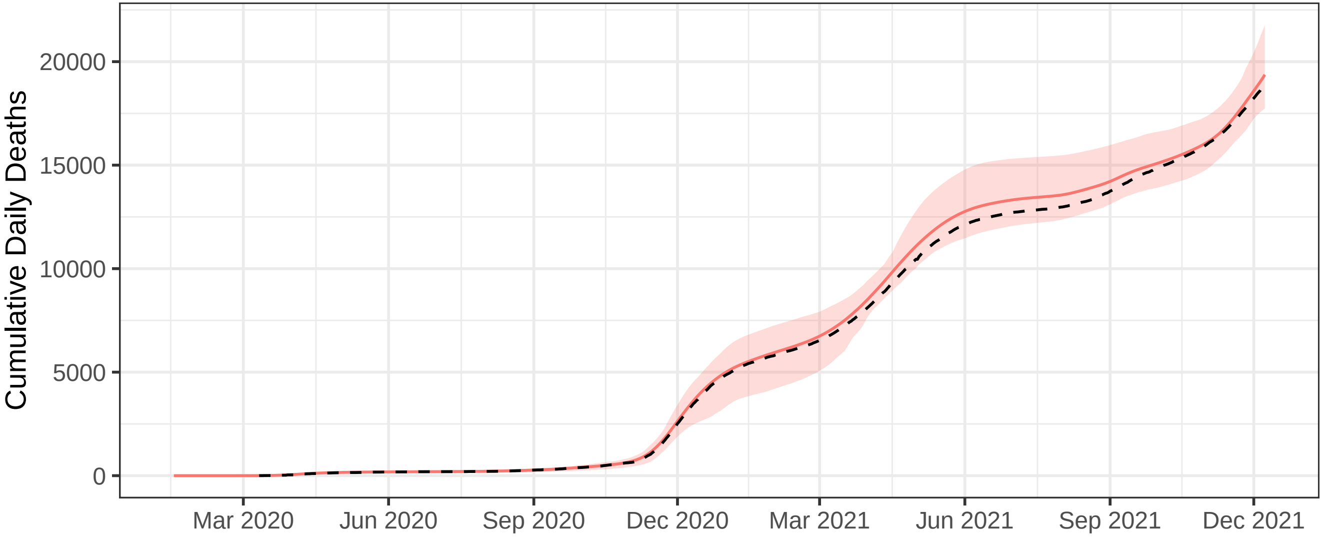

Daily deaths are included with reported COVID−19 deaths shown as points. The red line represents median model estimates of daily deaths, and the shaded region represents the 95% quantiles of the model estimated deaths. Plots of the cumulative estimated deaths are also shown, with the cumulative number of reported deaths represented by a dashed black line.

Grenada

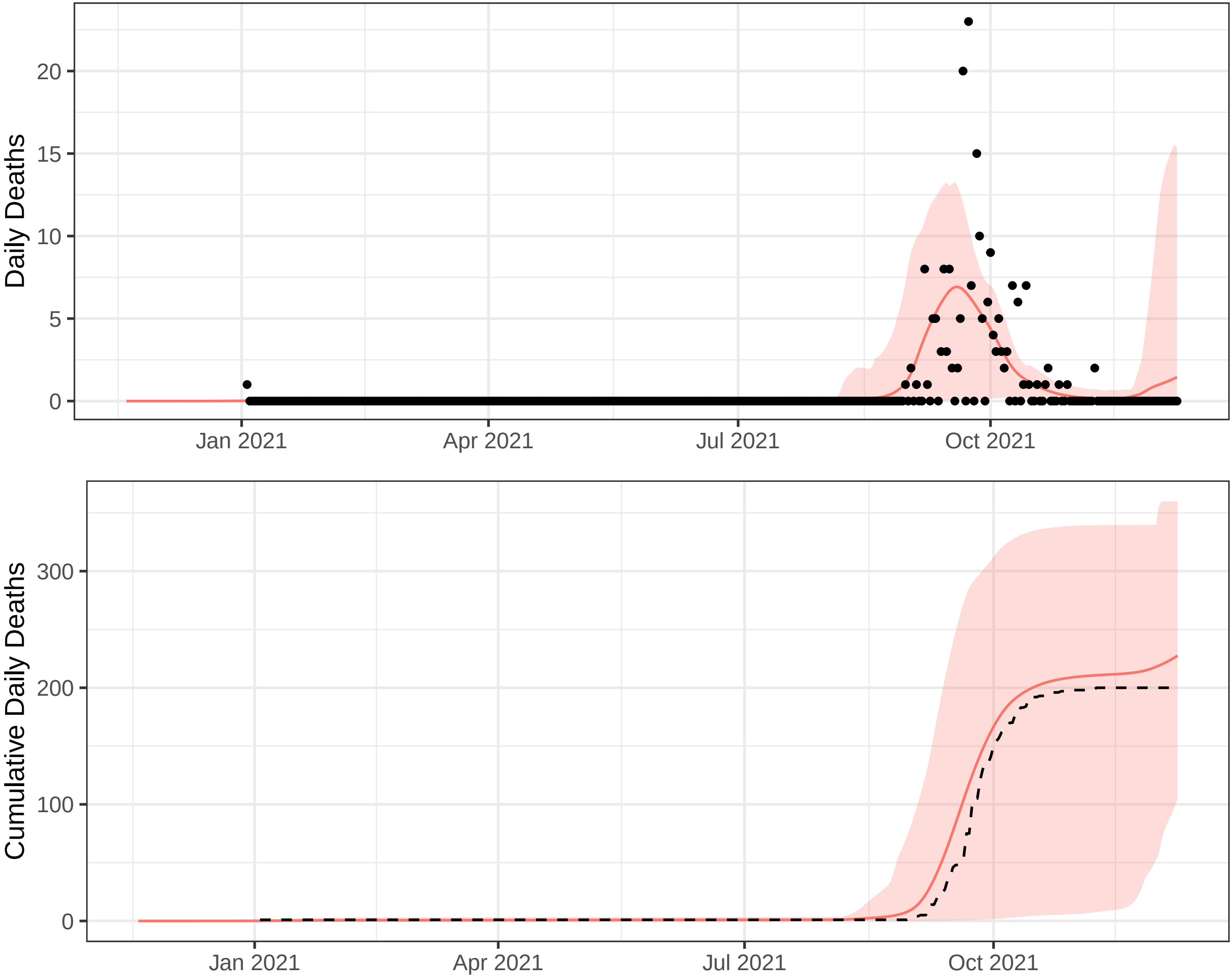

Daily deaths are included with reported COVID−19 deaths shown as points. The red line represents median model estimates of daily deaths, and the shaded region represents the 95% quantiles of the model estimated deaths. Plots of the cumulative estimated deaths are also shown, with the cumulative number of reported deaths represented by a dashed black line.

Guatemala

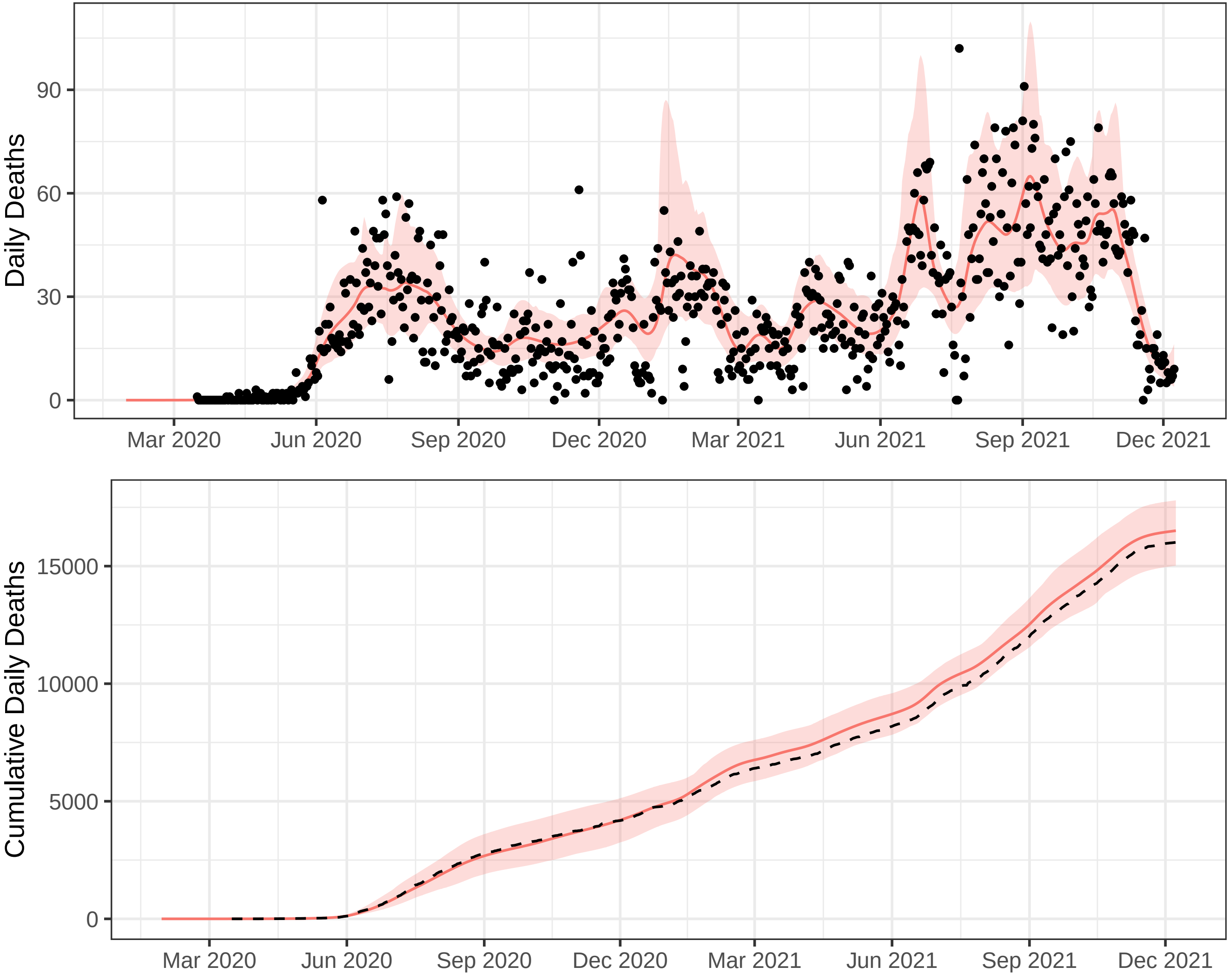

Daily deaths are included with reported COVID−19 deaths shown as points. The red line represents median model estimates of daily deaths, and the shaded region represents the 95% quantiles of the model estimated deaths. Plots of the cumulative estimated deaths are also shown, with the cumulative number of reported deaths represented by a dashed black line.

Guyana

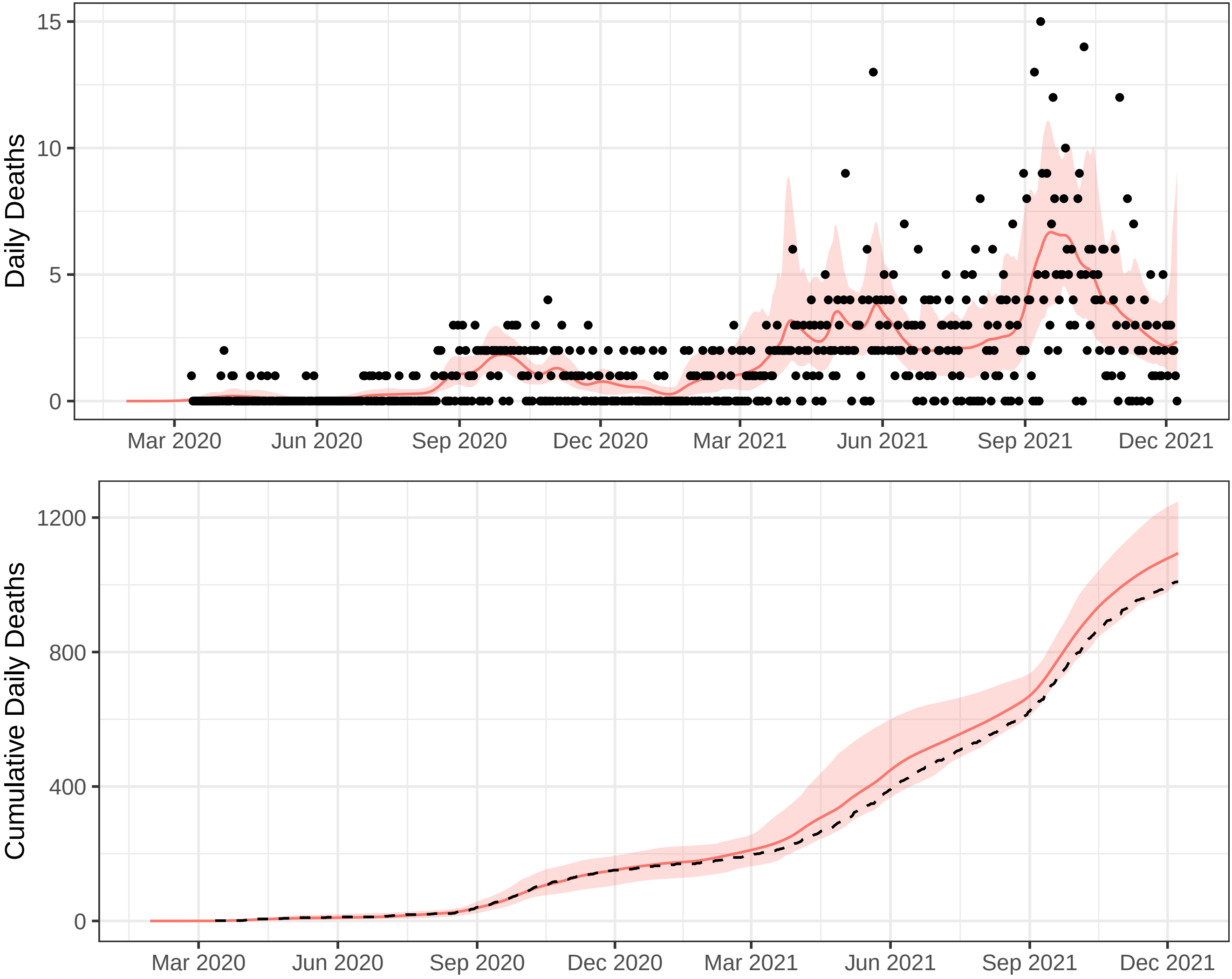

Daily deaths are included with reported COVID−19 deaths shown as points. The red line represents median model estimates of daily deaths, and the shaded region represents the 95% quantiles of the model estimated deaths. Plots of the cumulative estimated deaths are also shown, with the cumulative number of reported deaths represented by a dashed black line.

Hong Kong SAR, China

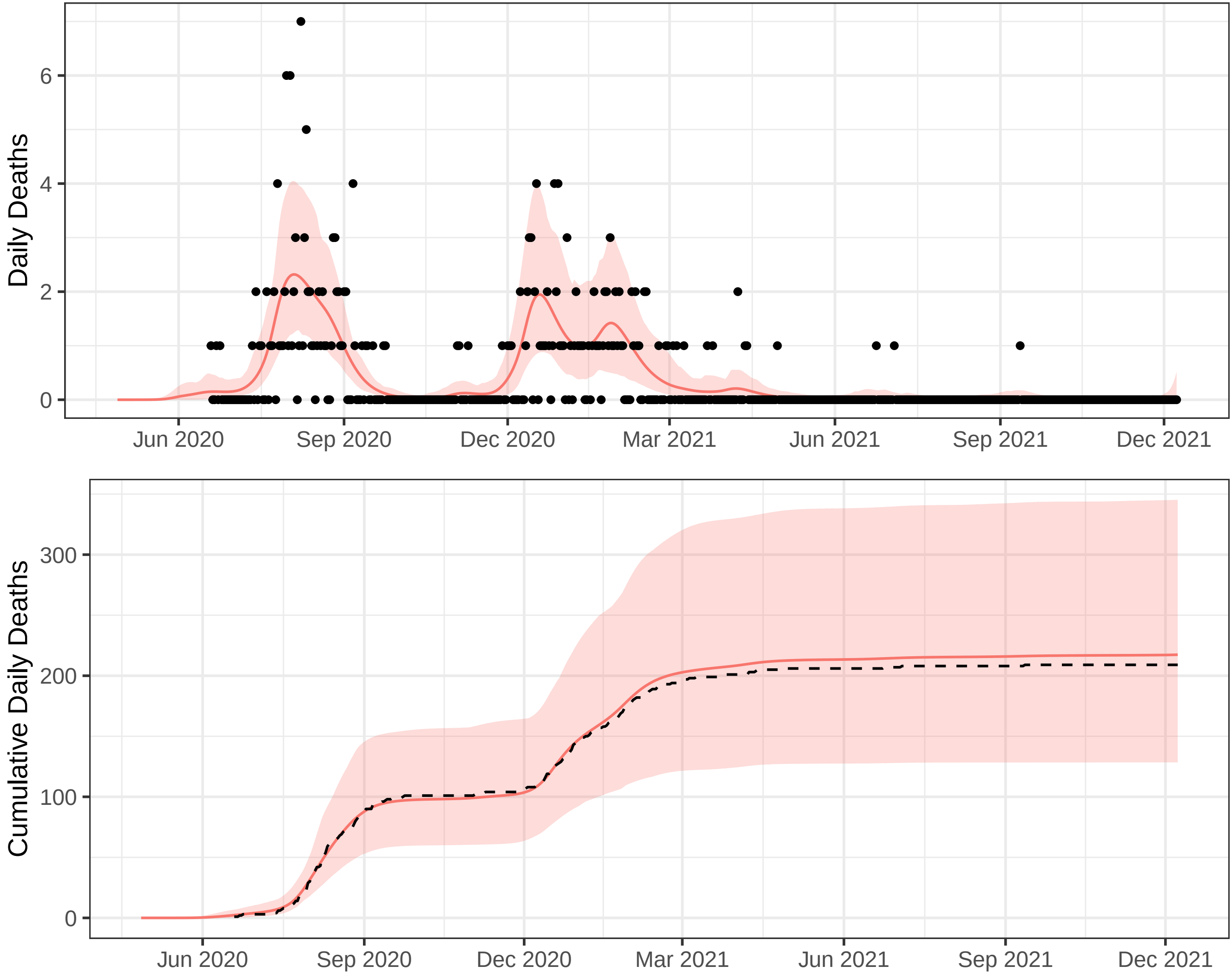

Daily deaths are included with reported COVID−19 deaths shown as points. The red line represents median model estimates of daily deaths, and the shaded region represents the 95% quantiles of the model estimated deaths. Plots of the cumulative estimated deaths are also shown, with the cumulative number of reported deaths represented by a dashed black line.

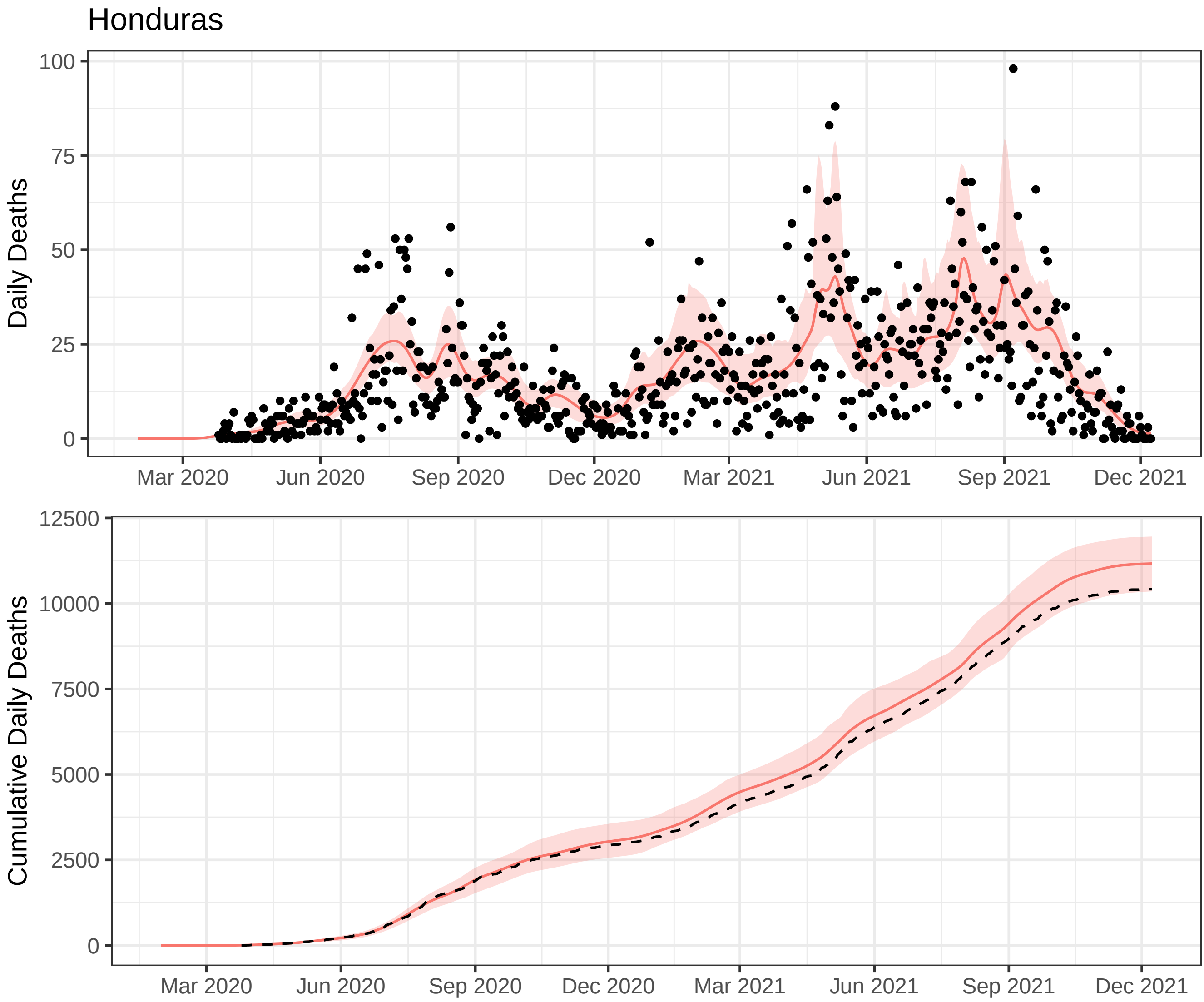

Daily deaths are included with reported COVID−19 deaths shown as points. The red line represents median model estimates of daily deaths, and the shaded region represents the 95% quantiles of the model estimated deaths. Plots of the cumulative estimated deaths are also shown, with the cumulative number of reported deaths represented by a dashed black line.

Croatia

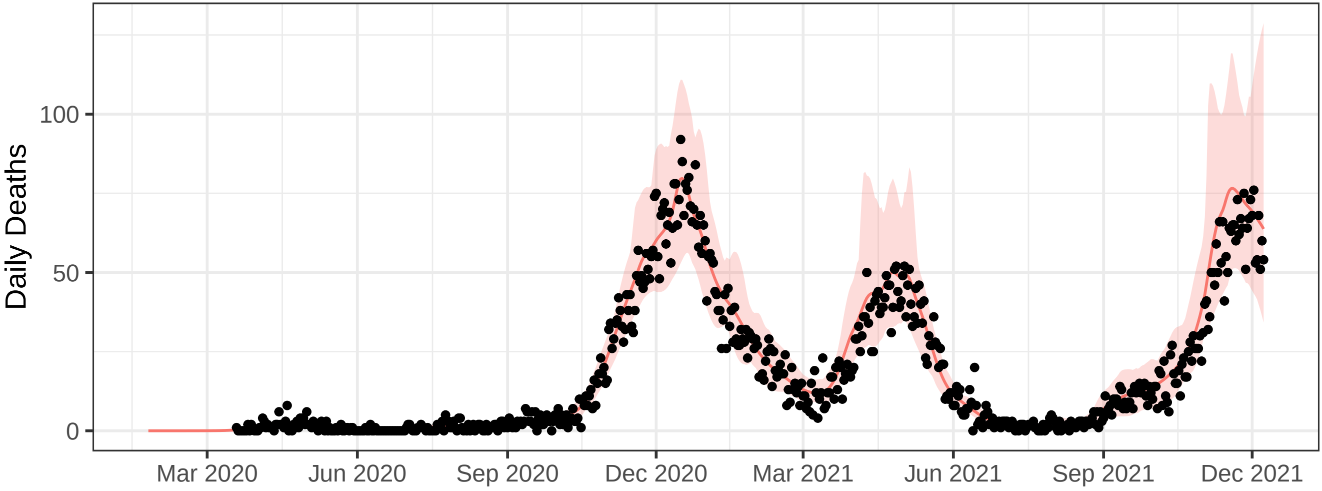

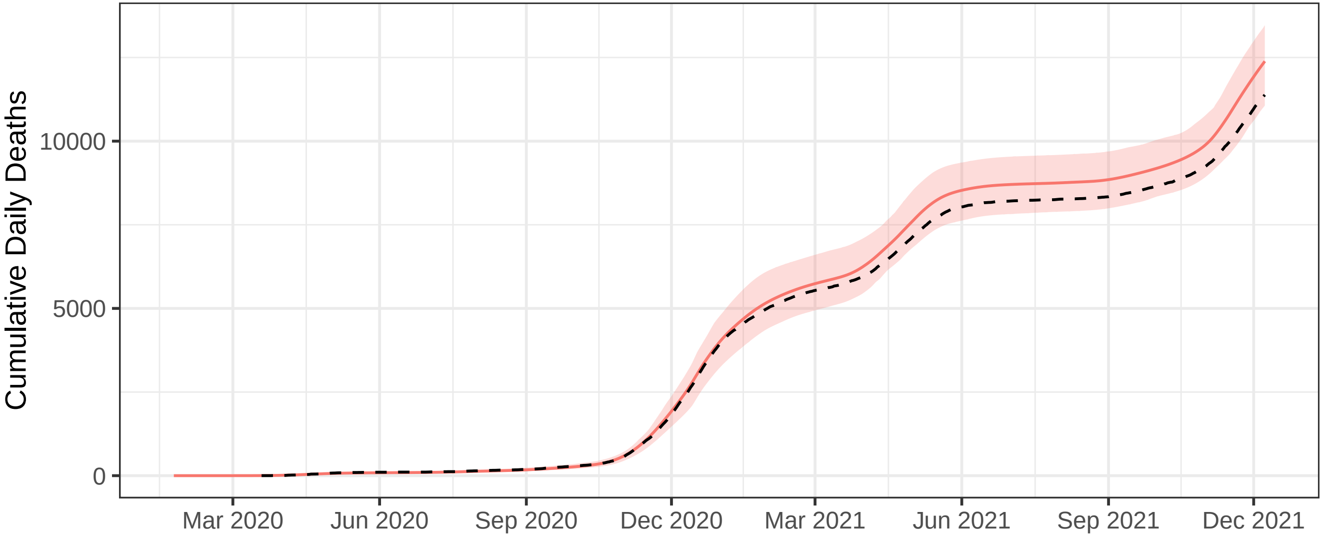

Daily deaths are included with reported COVID−19 deaths shown as points. The red line represents median model estimates of daily deaths, and the shaded region represents the 95% quantiles of the model estimated deaths. Plots of the cumulative estimated deaths are also shown, with the cumulative number of reported deaths represented by a dashed black line.

Haiti

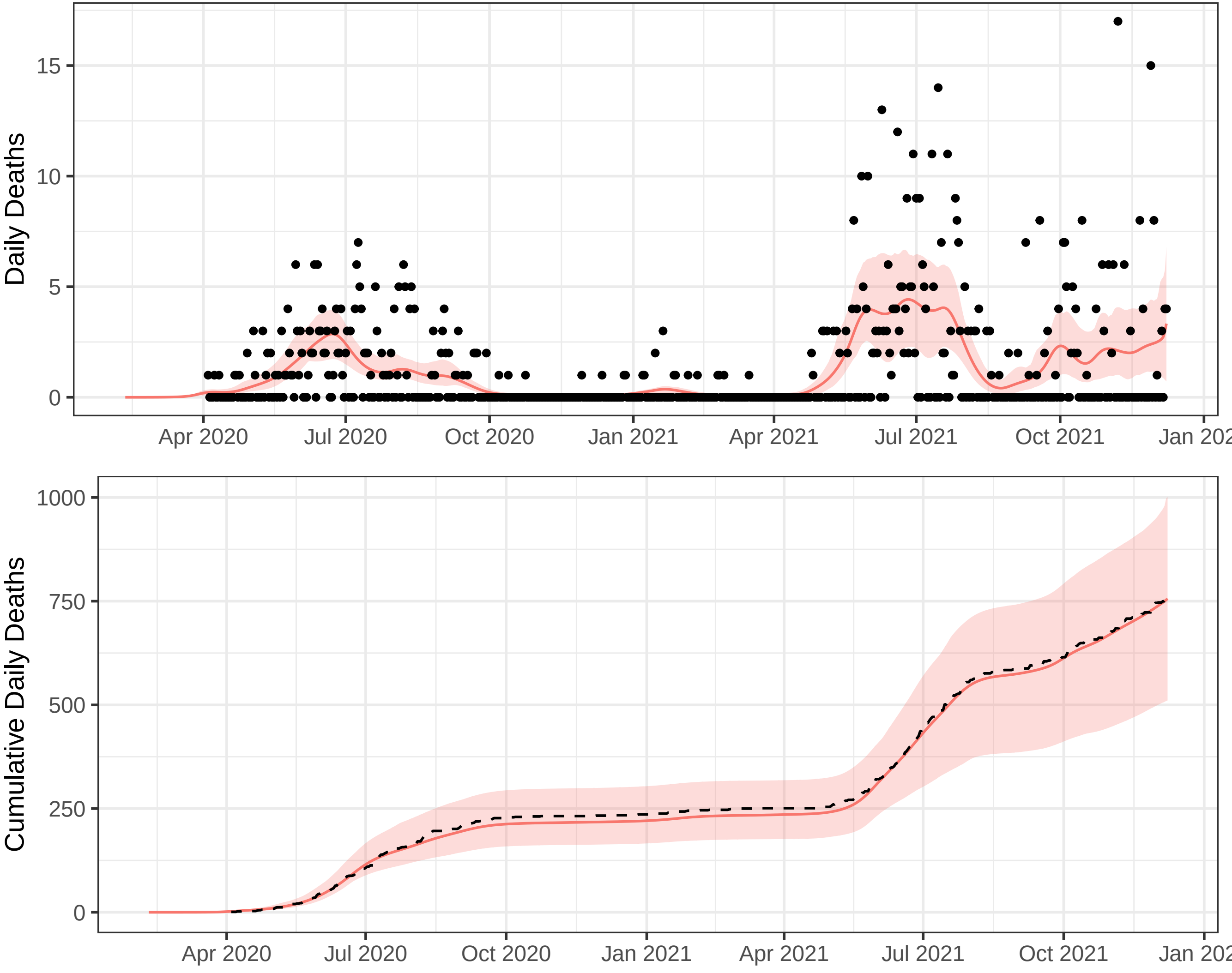

Daily deaths are included with reported COVID−19 deaths shown as points. The red line represents median model estimates of daily deaths, and the shaded region represents the 95% quantiles of the model estimated deaths. Plots of the cumulative estimated deaths are also shown, with the cumulative number of reported deaths represented by a dashed black line.

Hungary

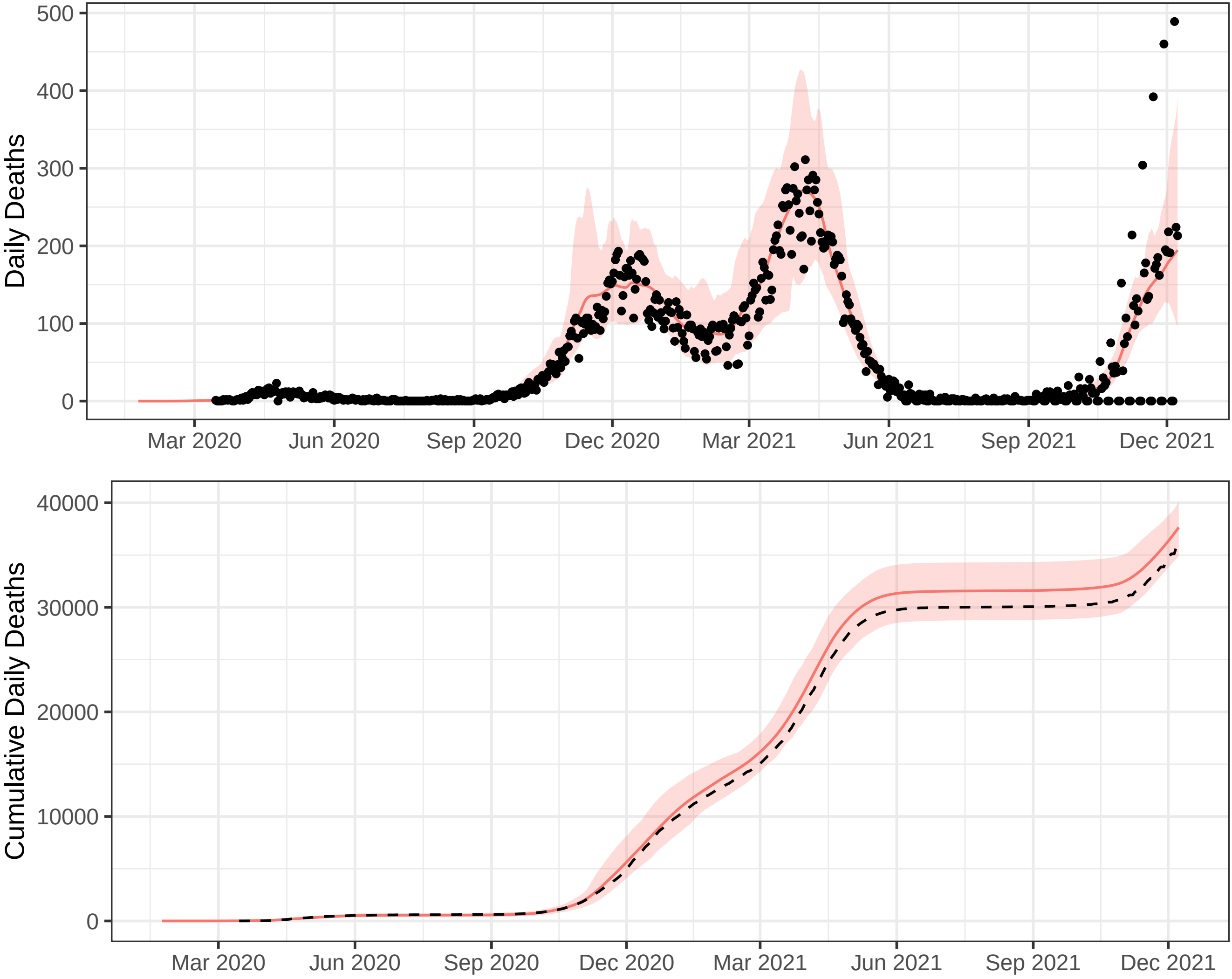

Daily deaths are included with reported COVID−19 deaths shown as points. The red line represents median model estimates of daily deaths, and the shaded region represents the 95% quantiles of the model estimated deaths. Plots of the cumulative estimated deaths are also shown, with the cumulative number of reported deaths represented by a dashed black line.

Indonesia

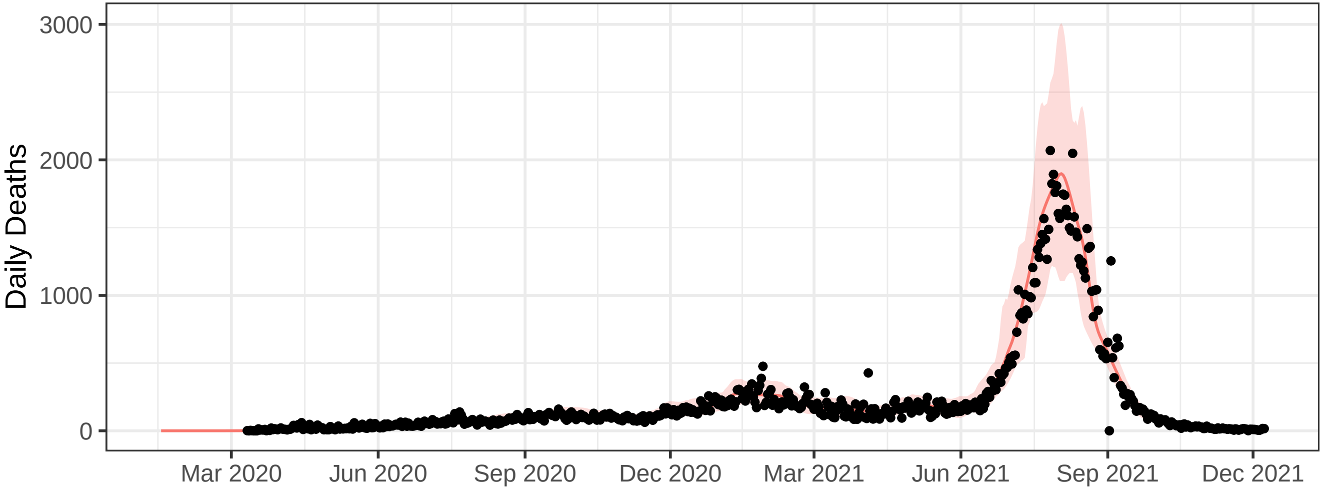

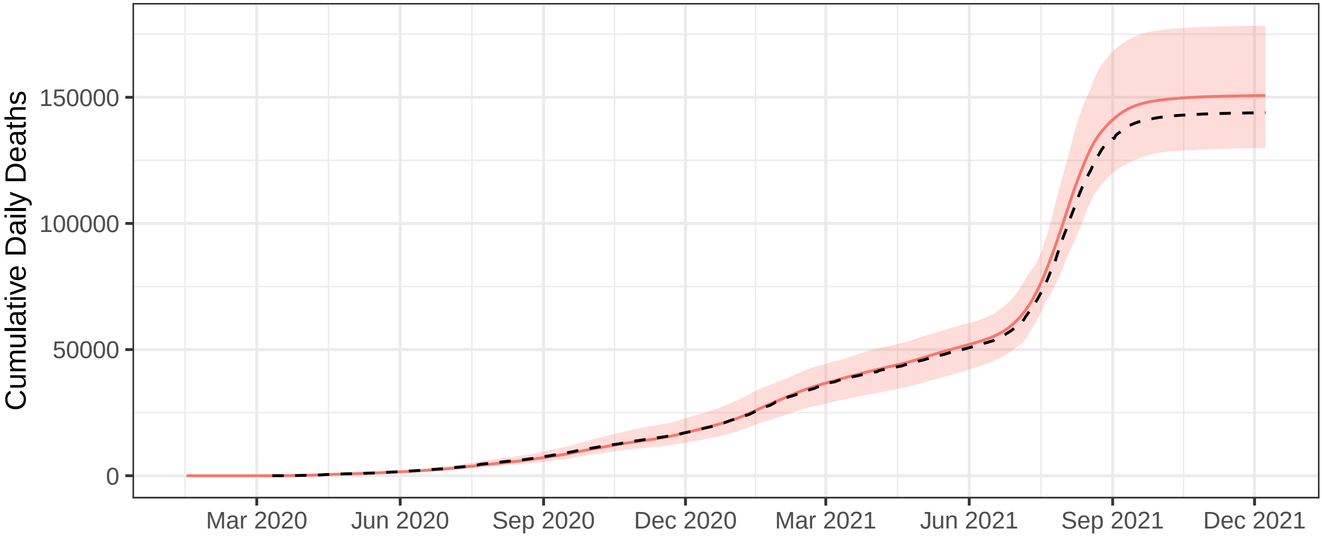

Daily deaths are included with reported COVID−19 deaths shown as points. The red line represents median model estimates of daily deaths, and the shaded region represents the 95% quantiles of the model estimated deaths. Plots of the cumulative estimated deaths are also shown, with the cumulative number of reported deaths represented by a dashed black line.

India

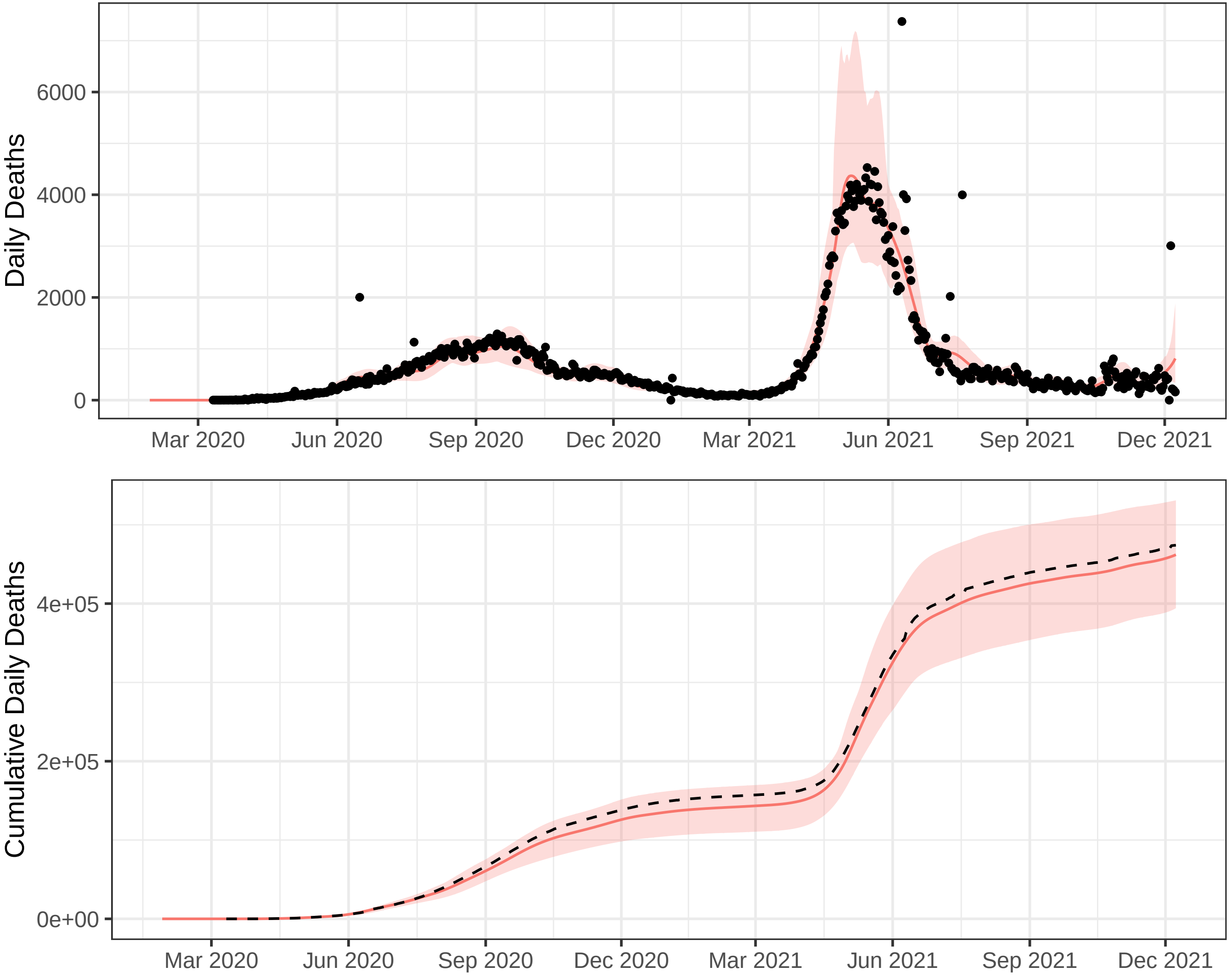

Daily deaths are included with reported COVID−19 deaths shown as points. The red line represents median model estimates of daily deaths, and the shaded region represents the 95% quantiles of the model estimated deaths. Plots of the cumulative estimated deaths are also shown, with the cumulative number of reported deaths represented by a dashed black line.

Ireland

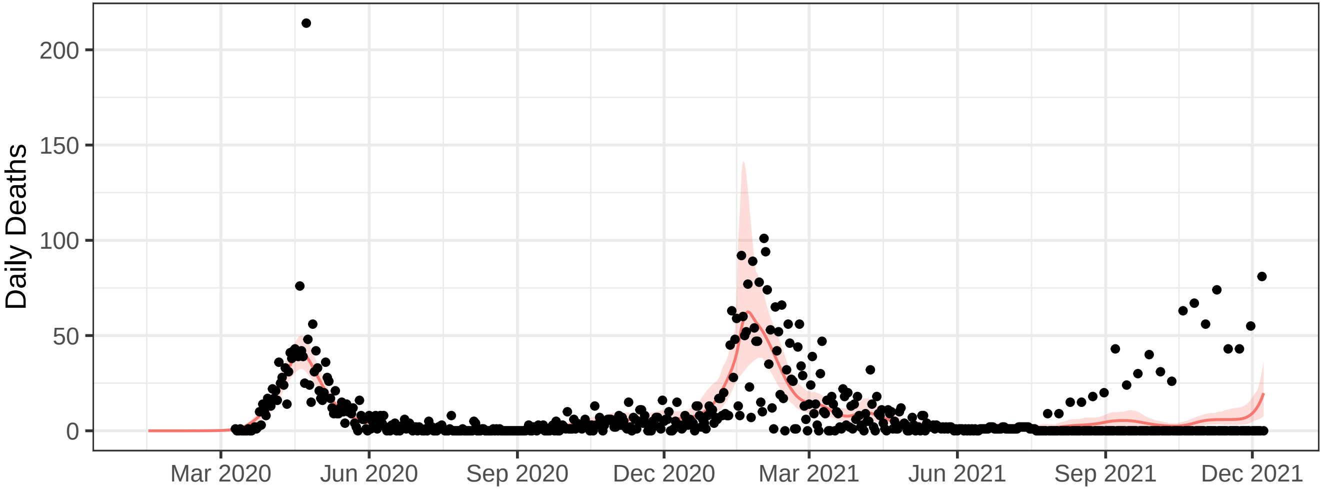

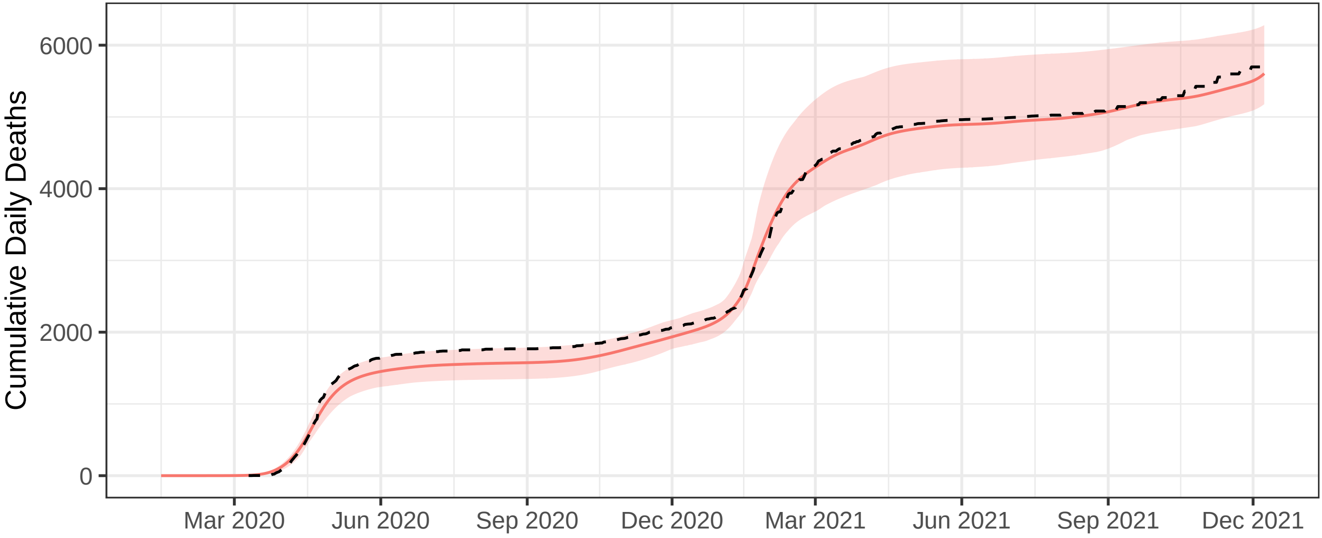

Daily deaths are included with reported COVID−19 deaths shown as points. The red line represents median model estimates of daily deaths, and the shaded region represents the 95% quantiles of the model estimated deaths. Plots of the cumulative estimated deaths are also shown, with the cumulative number of reported deaths represented by a dashed black line.

Iran

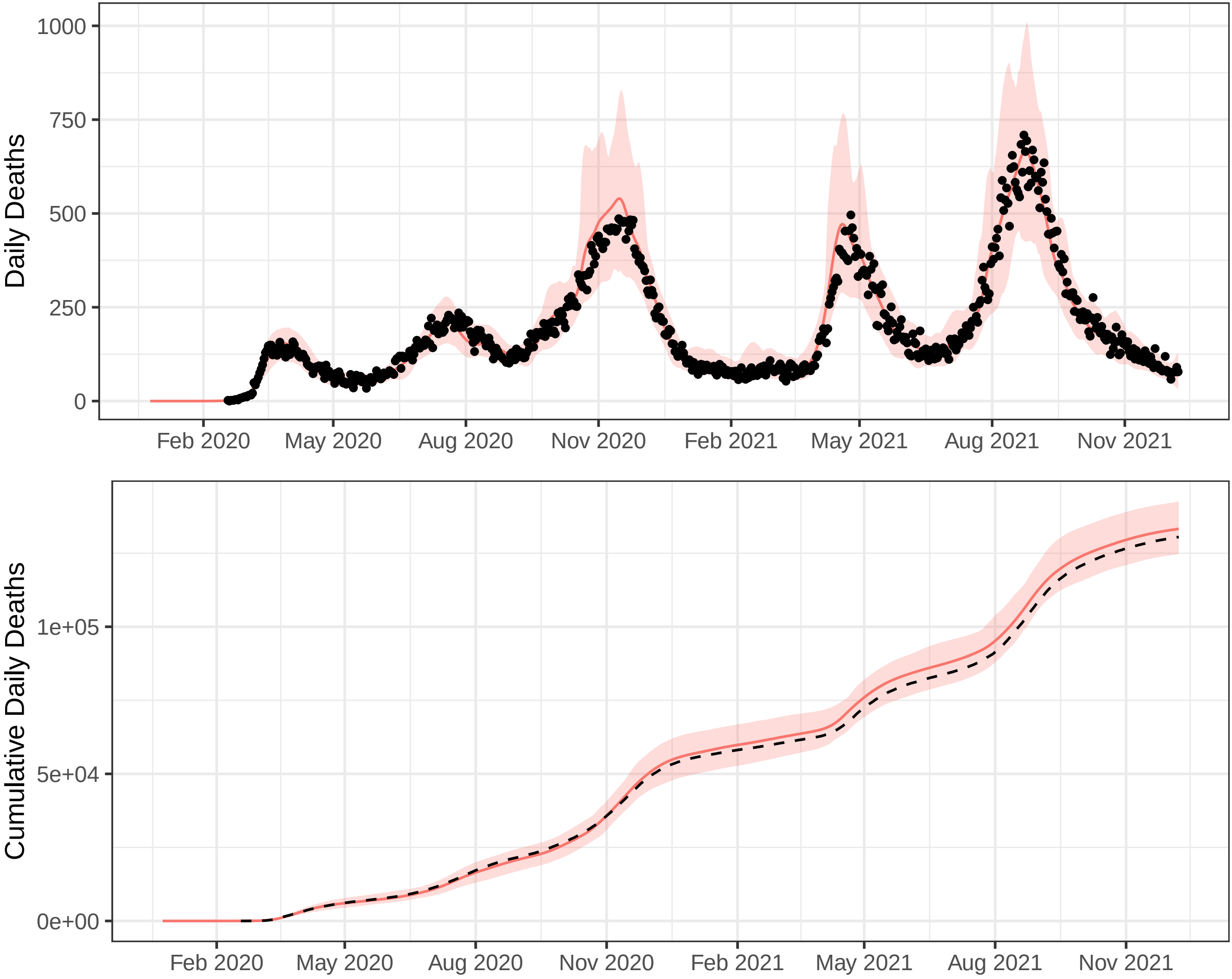

Daily deaths are included with reported COVID−19 deaths shown as points. The red line represents median model estimates of daily deaths, and the shaded region represents the 95% quantiles of the model estimated deaths. Plots of the cumulative estimated deaths are also shown, with the cumulative number of reported deaths represented by a dashed black line.

Iraq

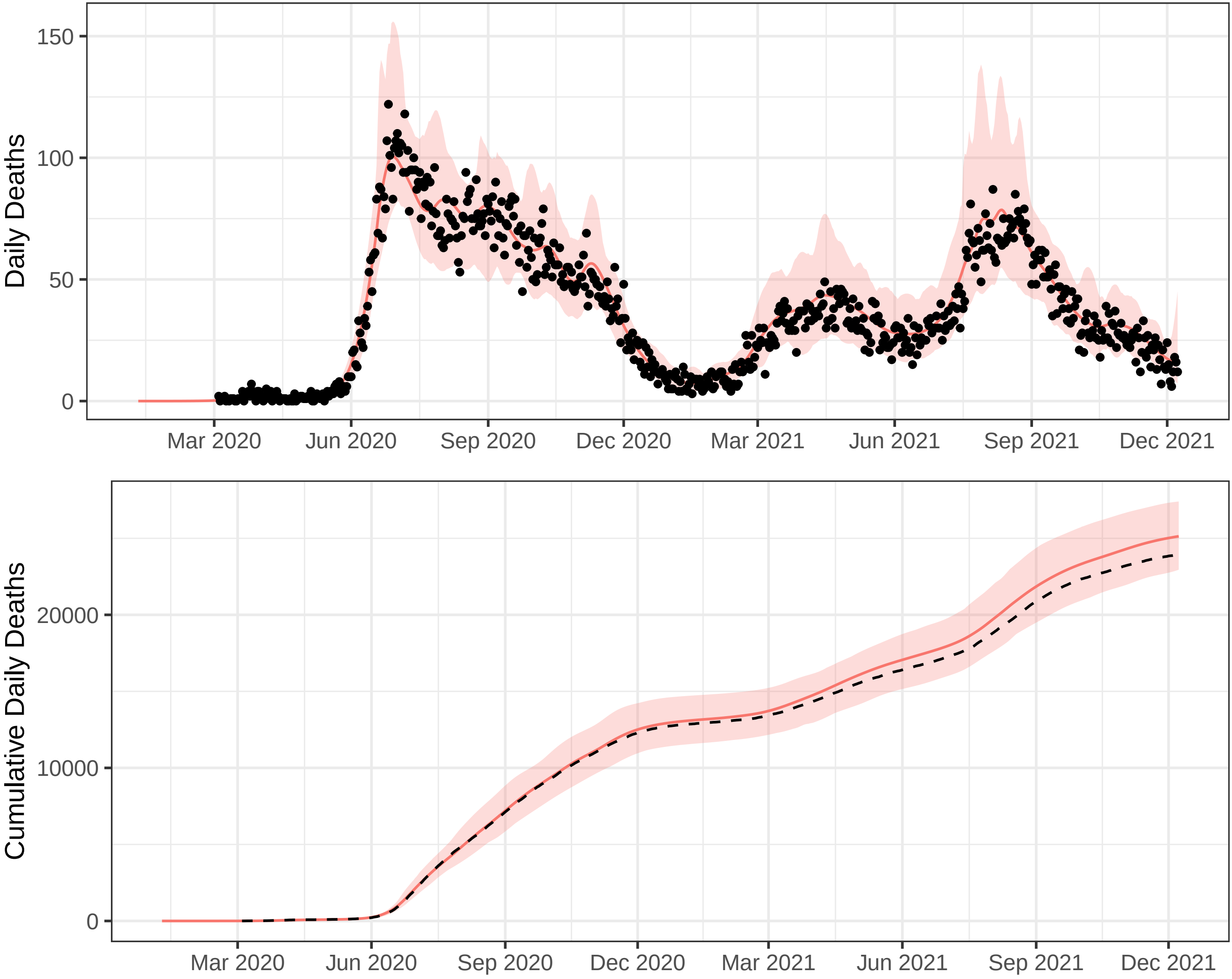

Daily deaths are included with reported COVID−19 deaths shown as points. The red line represents median model estimates of daily deaths, and the shaded region represents the 95% quantiles of the model estimated deaths. Plots of the cumulative estimated deaths are also shown, with the cumulative number of reported deaths represented by a dashed black line.

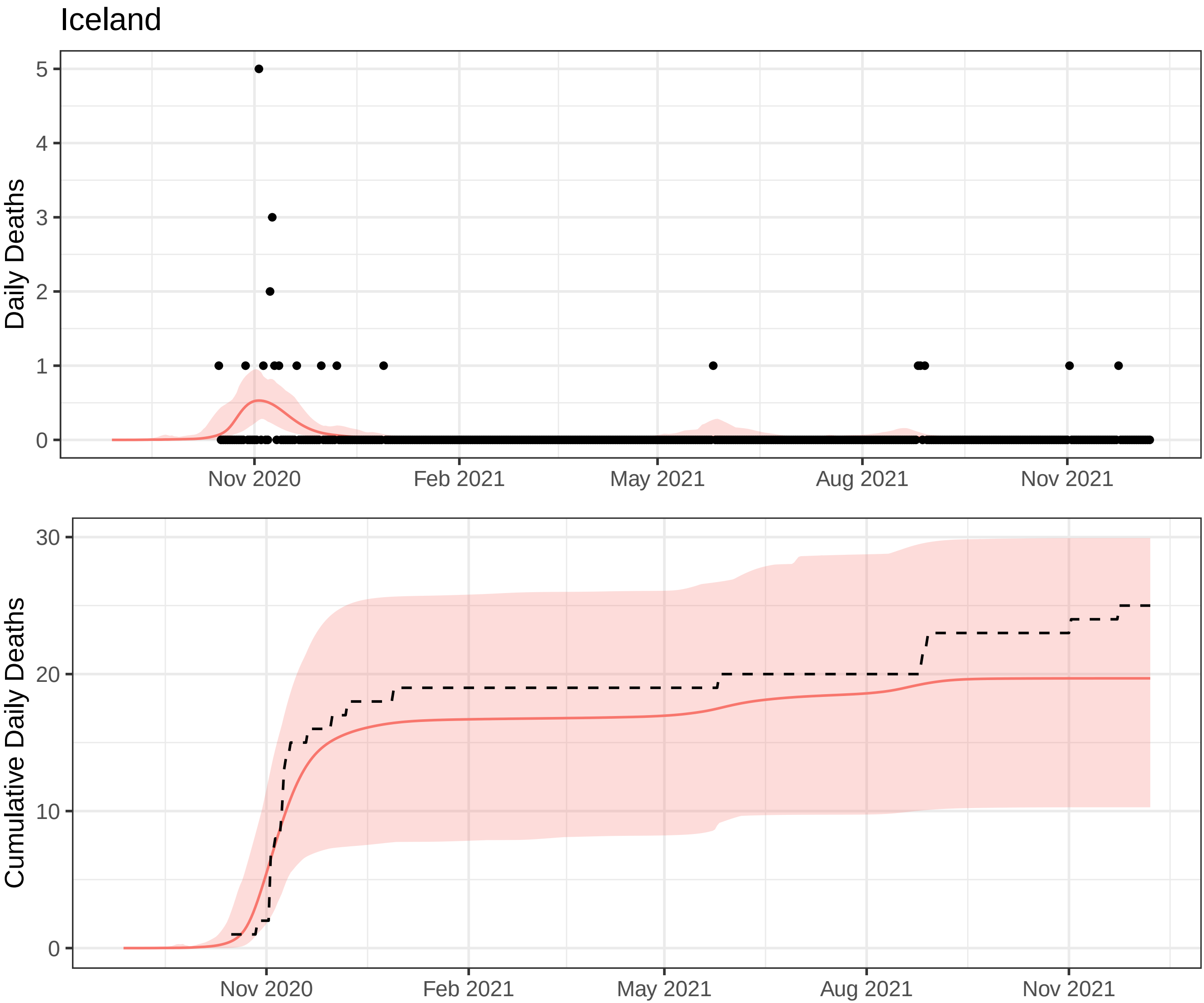

Daily deaths are included with reported COVID−19 deaths shown as points. The red line represents median model estimates of daily deaths, and the shaded region represents the 95% quantiles of the model estimated deaths. Plots of the cumulative estimated deaths are also shown, with the cumulative number of reported deaths represented by a dashed black line.

Israel

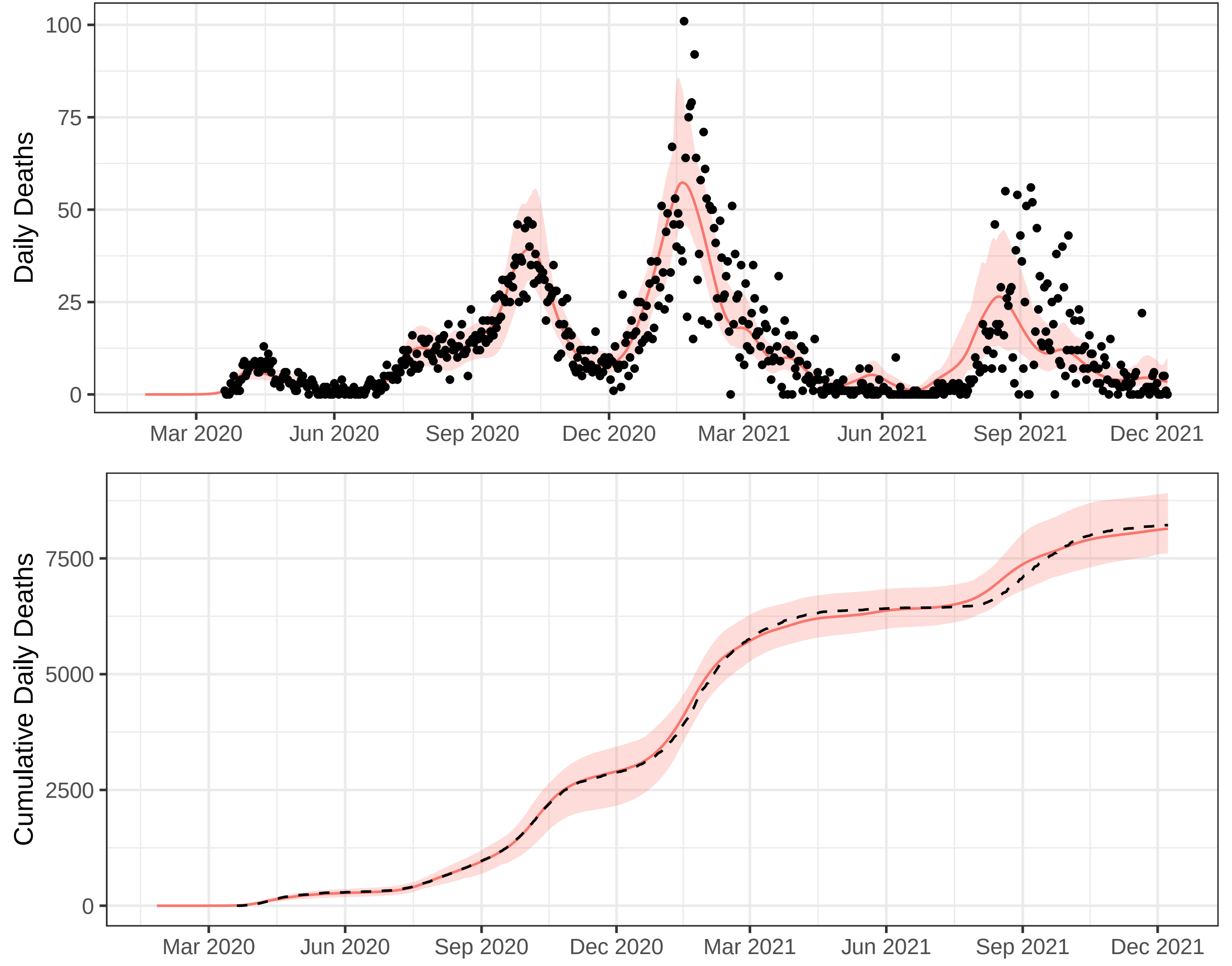

Daily deaths are included with reported COVID−19 deaths shown as points. The red line represents median model estimates of daily deaths, and the shaded region represents the 95% quantiles of the model estimated deaths. Plots of the cumulative estimated deaths are also shown, with the cumulative number of reported deaths represented by a dashed black line.

Italy

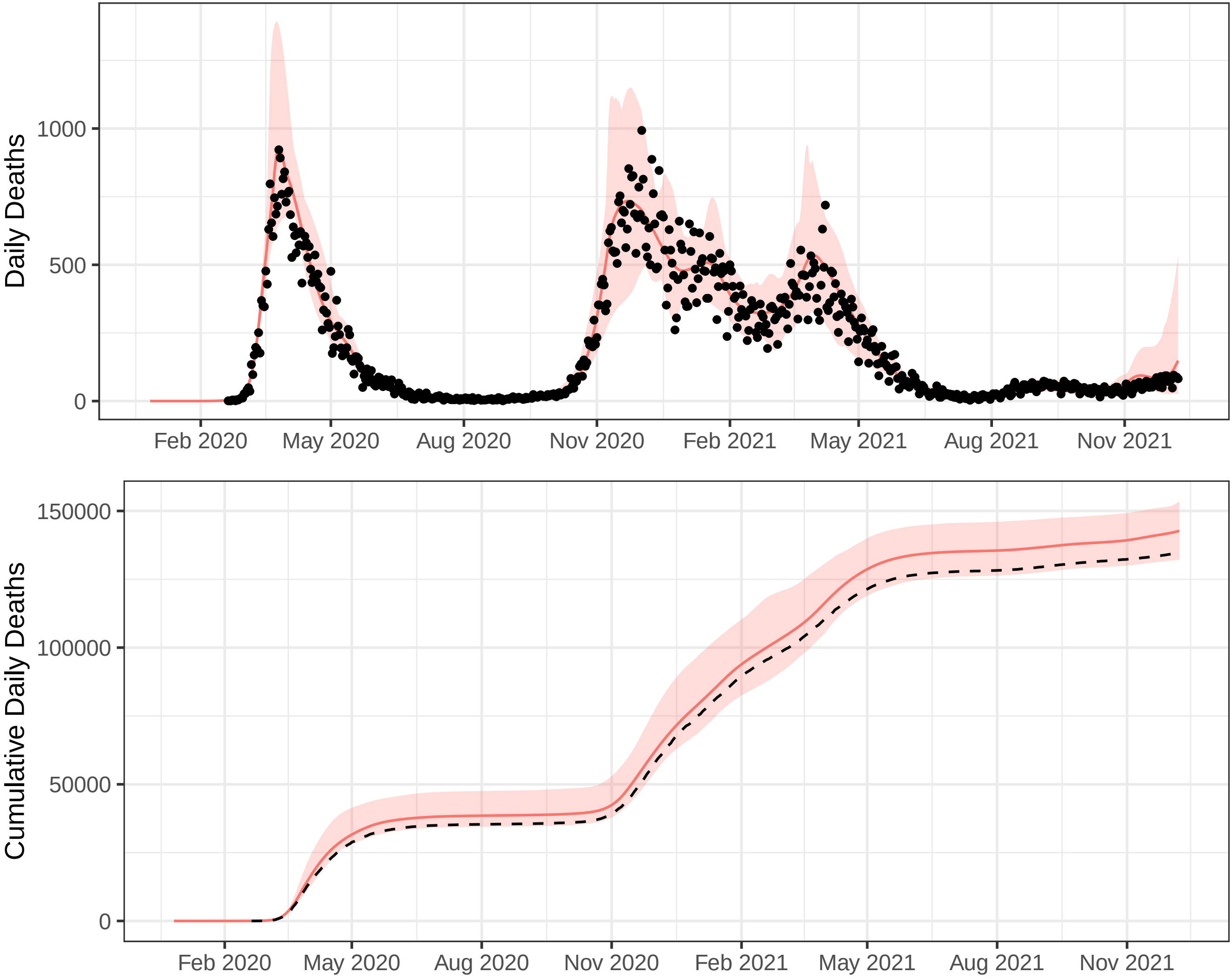

Daily deaths are included with reported COVID−19 deaths shown as points. The red line represents median model estimates of daily deaths, and the shaded region represents the 95% quantiles of the model estimated deaths. Plots of the cumulative estimated deaths are also shown, with the cumulative number of reported deaths represented by a dashed black line.

Jamaica

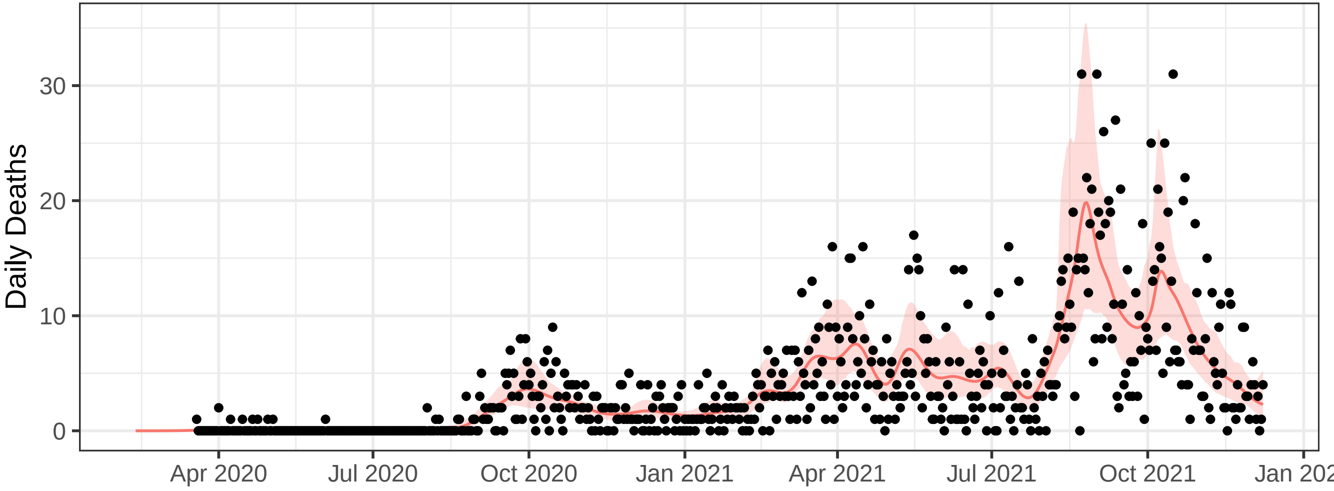

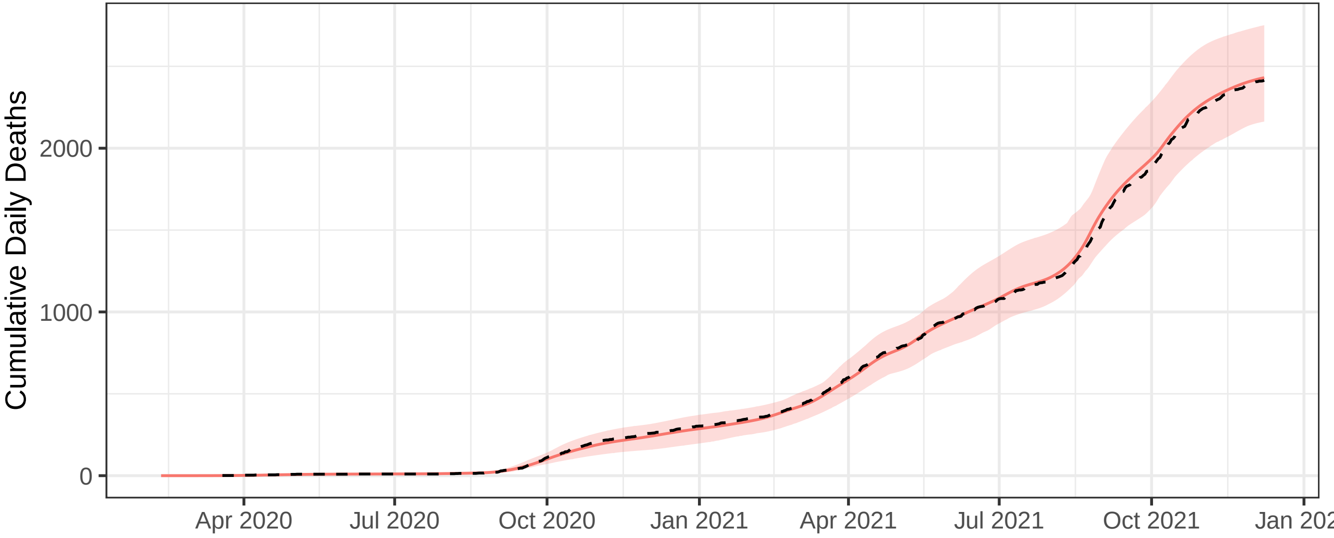

Daily deaths are included with reported COVID−19 deaths shown as points. The red line represents median model estimates of daily deaths, and the shaded region represents the 95% quantiles of the model estimated deaths. Plots of the cumulative estimated deaths are also shown, with the cumulative number of reported deaths represented by a dashed black line.

Jordan

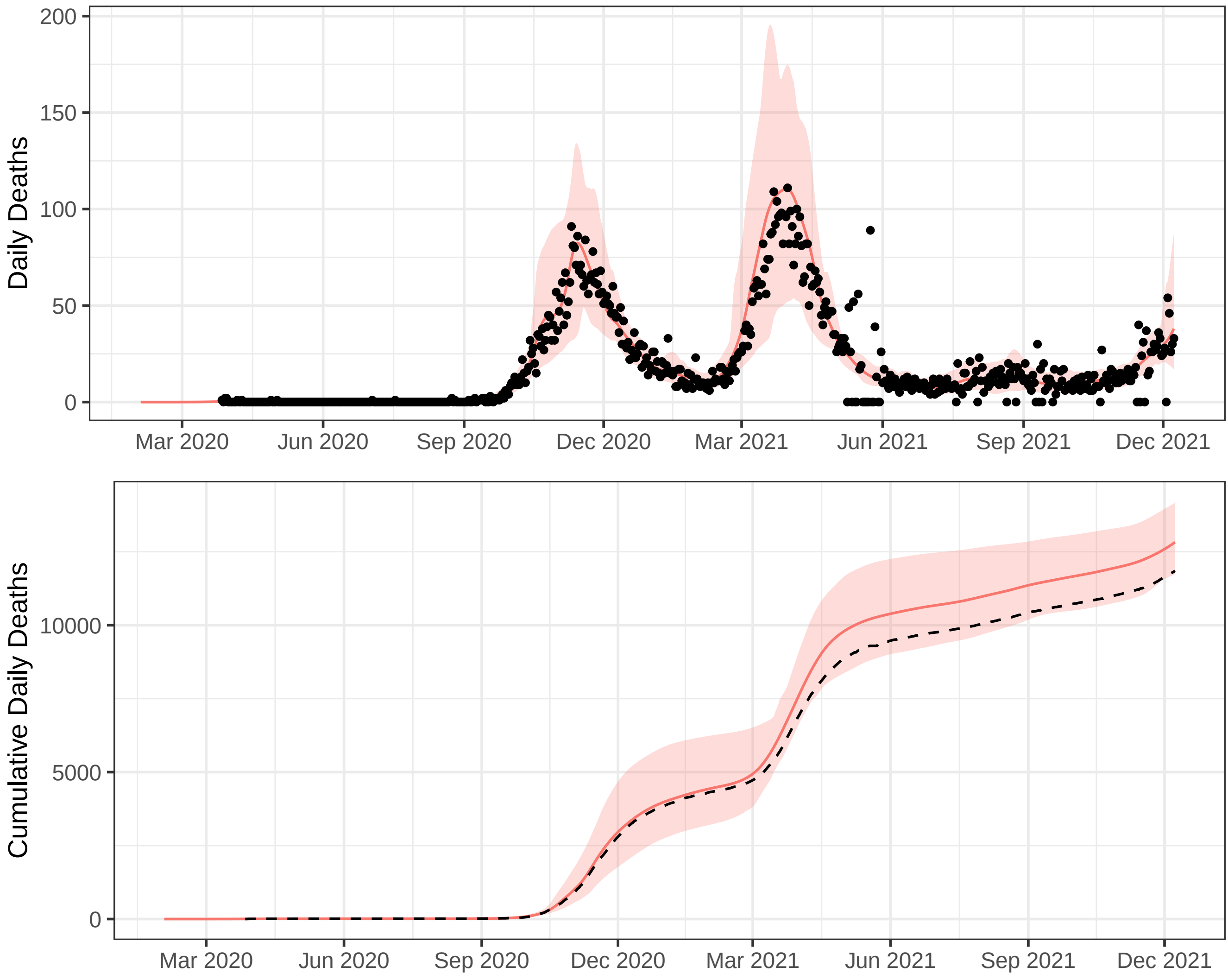

Daily deaths are included with reported COVID−19 deaths shown as points. The red line represents median model estimates of daily deaths, and the shaded region represents the 95% quantiles of the model estimated deaths. Plots of the cumulative estimated deaths are also shown, with the cumulative number of reported deaths represented by a dashed black line.

Japan

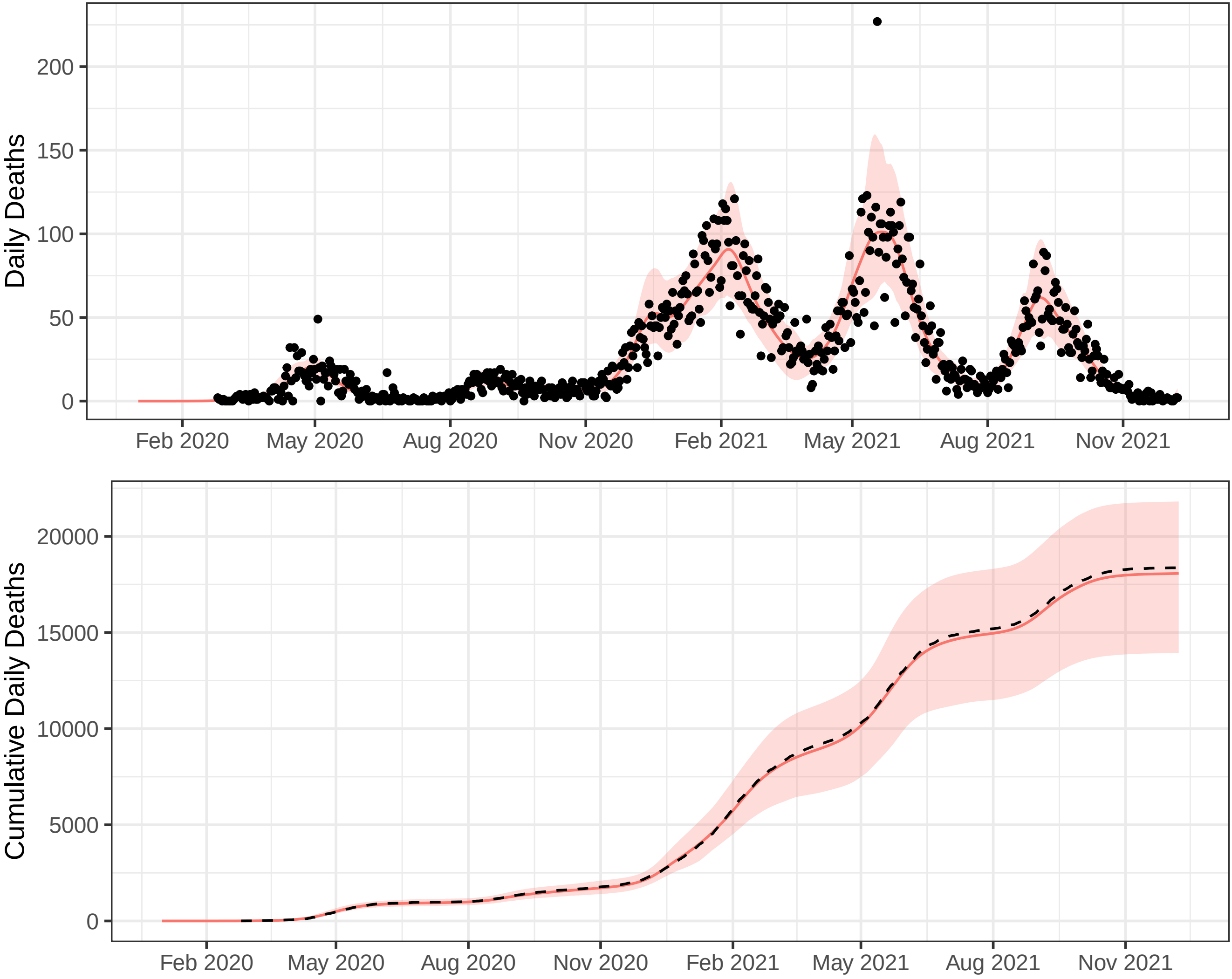

Daily deaths are included with reported COVID−19 deaths shown as points. The red line represents median model estimates of daily deaths, and the shaded region represents the 95% quantiles of the model estimated deaths. Plots of the cumulative estimated deaths are also shown, with the cumulative number of reported deaths represented by a dashed black line.

Kazakhstan

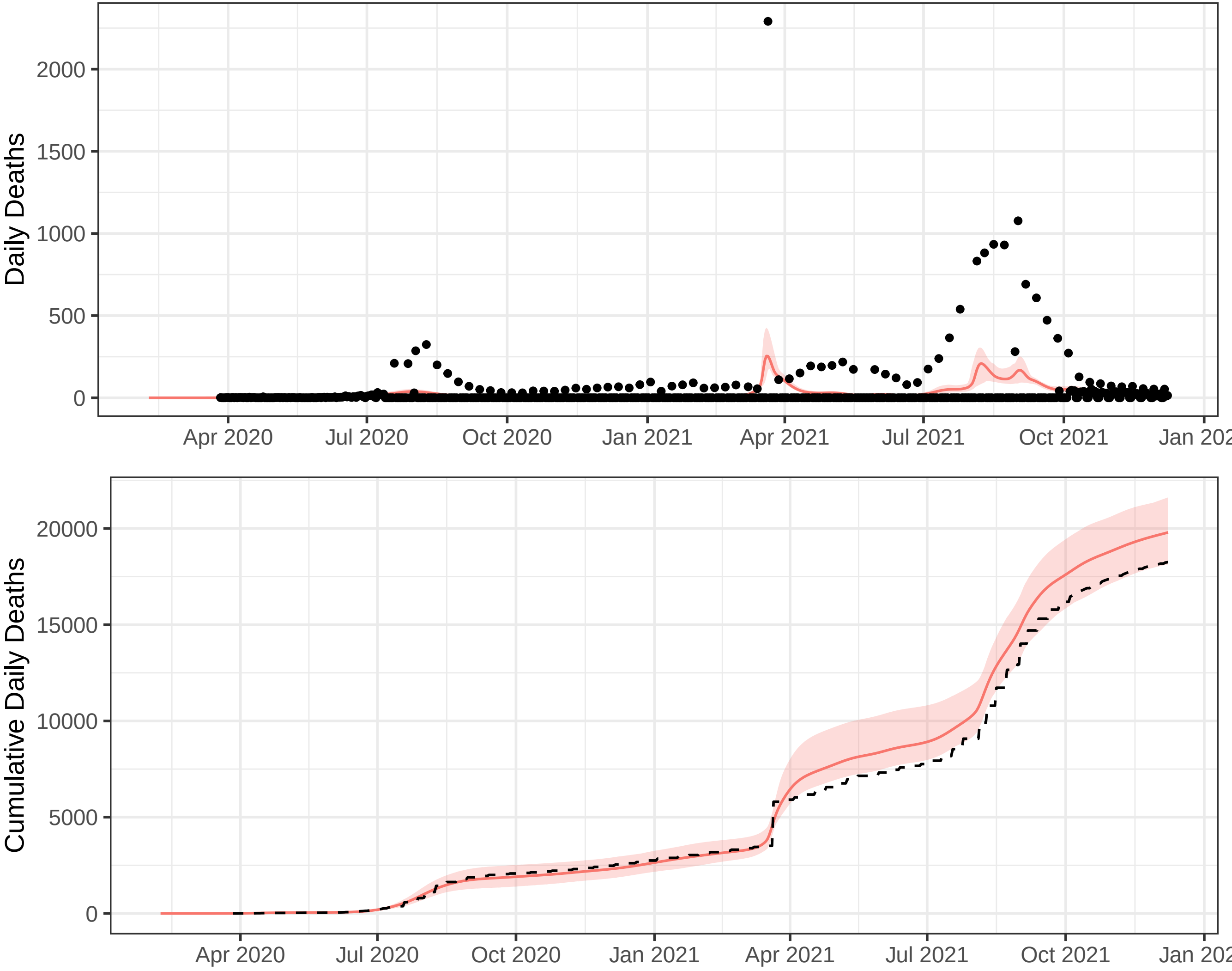

Daily deaths are included with reported COVID−19 deaths shown as points. The red line represents median model estimates of daily deaths, and the shaded region represents the 95% quantiles of the model estimated deaths. Plots of the cumulative estimated deaths are also shown, with the cumulative number of reported deaths represented by a dashed black line.

Kenya

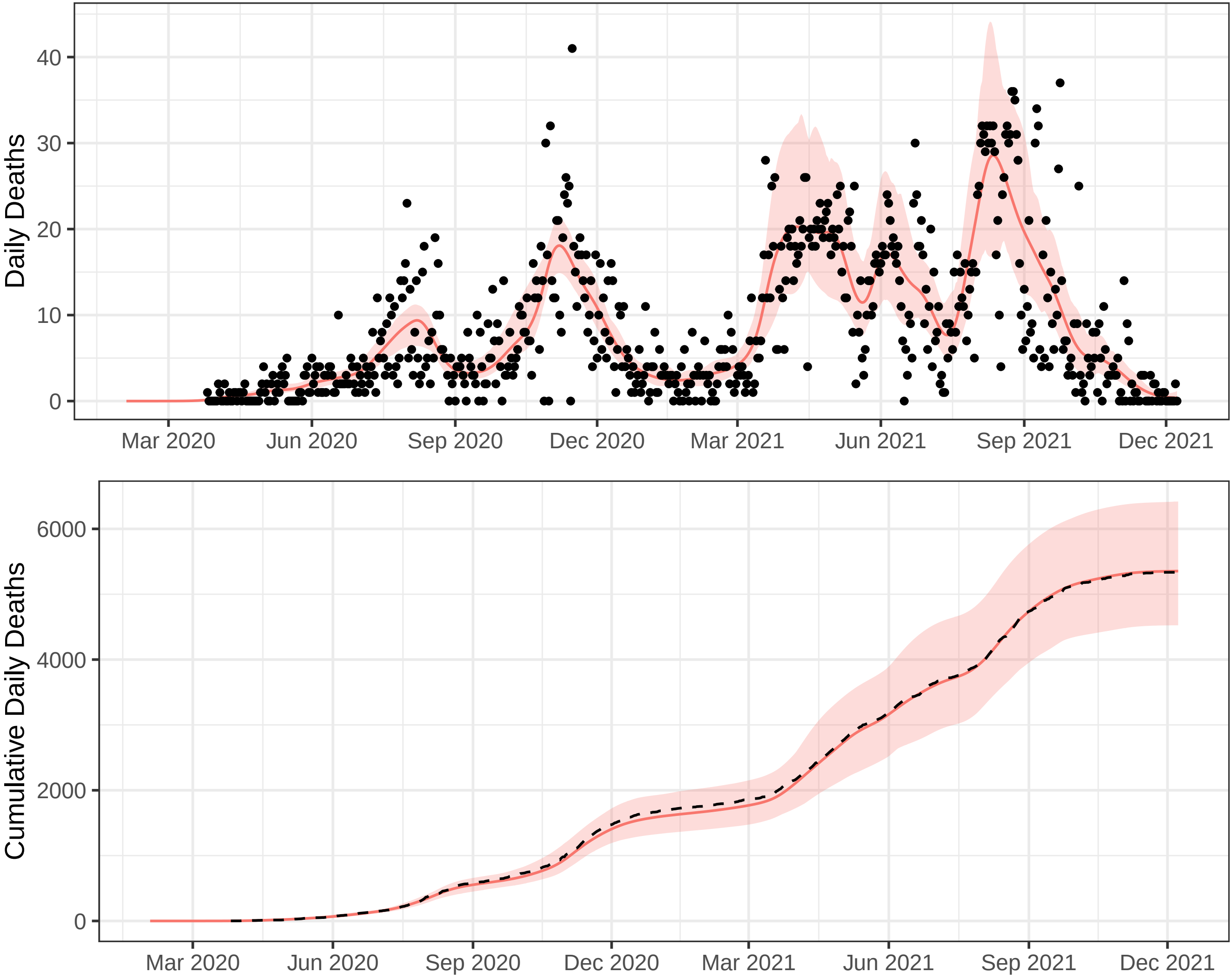

Daily deaths are included with reported COVID−19 deaths shown as points. The red line represents median model estimates of daily deaths, and the shaded region represents the 95% quantiles of the model estimated deaths. Plots of the cumulative estimated deaths are also shown, with the cumulative number of reported deaths represented by a dashed black line.

Kyrgyz Republic

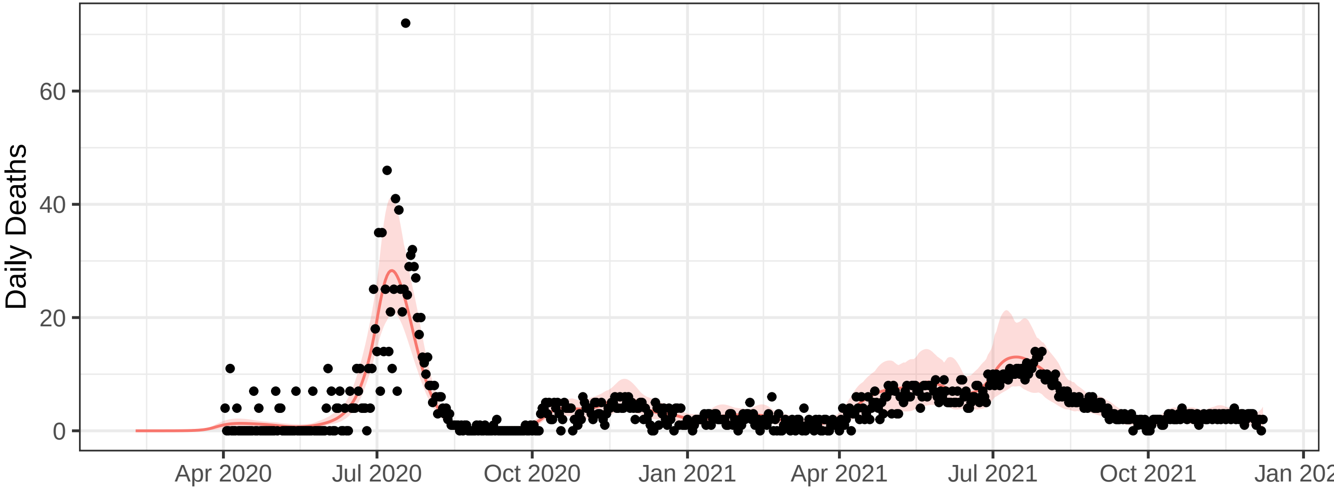

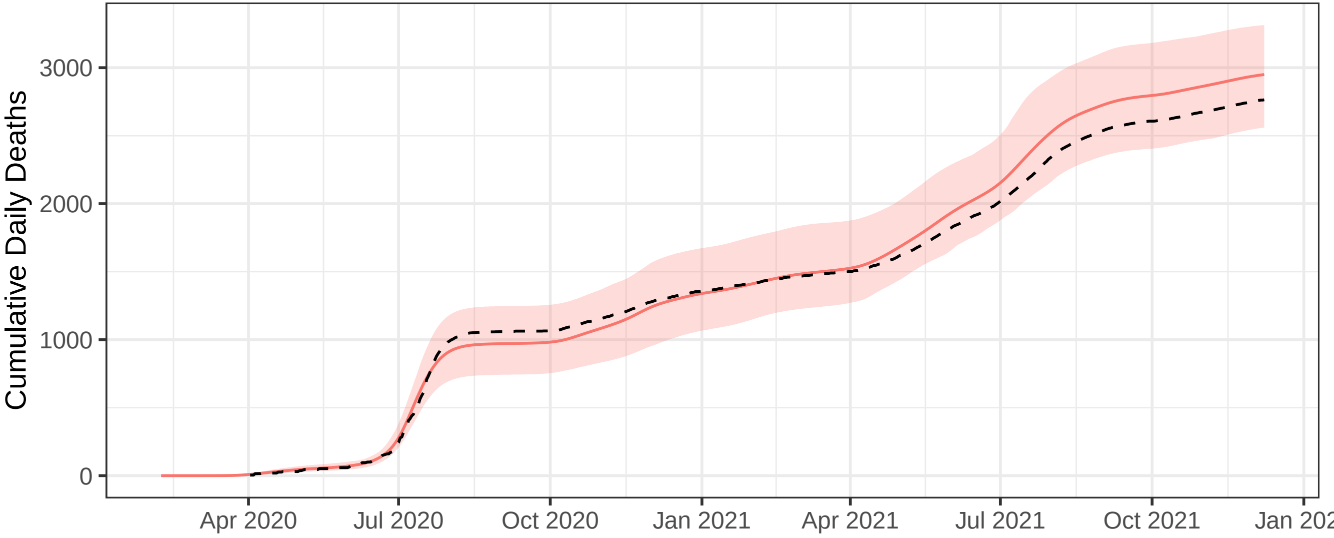

Daily deaths are included with reported COVID−19 deaths shown as points. The red line represents median model estimates of daily deaths, and the shaded region represents the 95% quantiles of the model estimated deaths. Plots of the cumulative estimated deaths are also shown, with the cumulative number of reported deaths represented by a dashed black line.

Cambodia

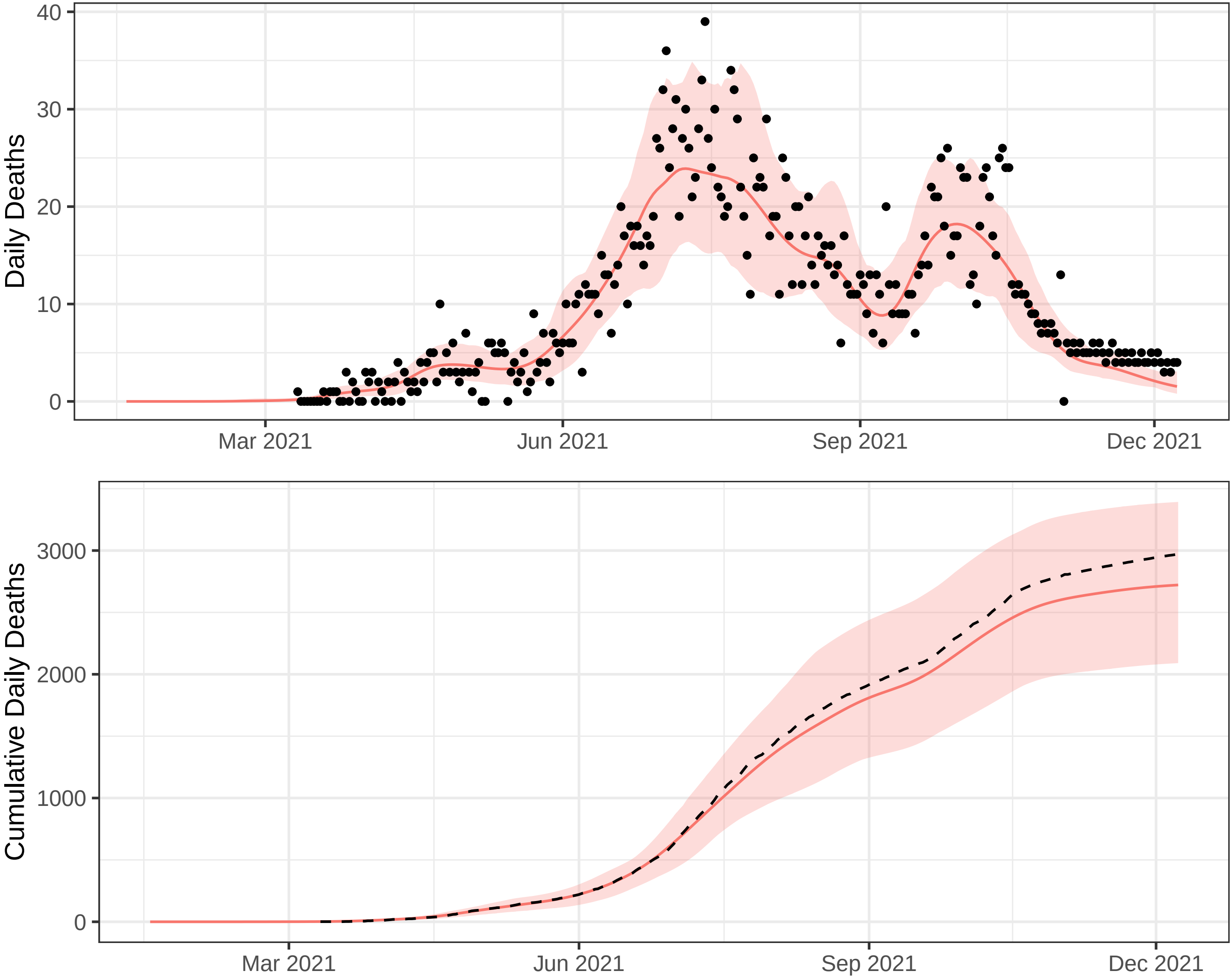

Daily deaths are included with reported COVID−19 deaths shown as points. The red line represents median model estimates of daily deaths, and the shaded region represents the 95% quantiles of the model estimated deaths. Plots of the cumulative estimated deaths are also shown, with the cumulative number of reported deaths represented by a dashed black line.

South Korea

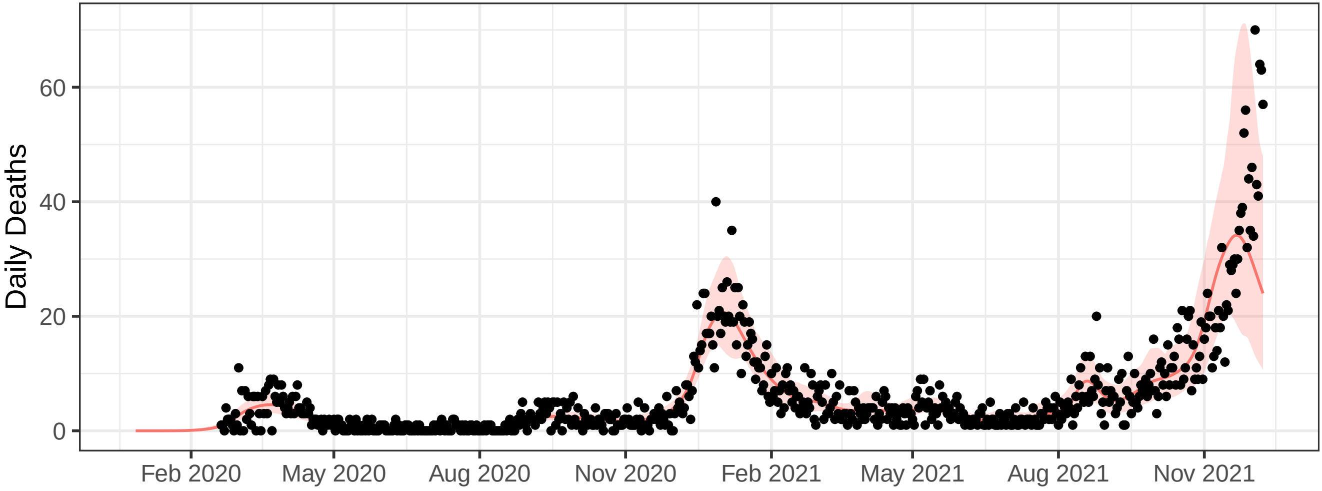

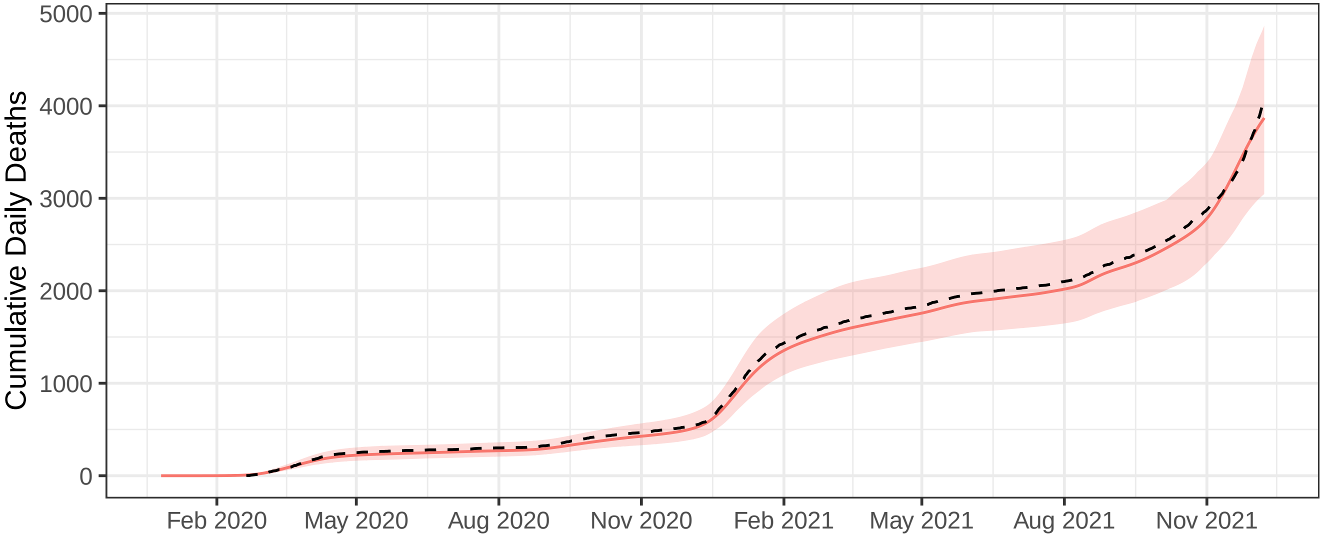

Daily deaths are included with reported COVID−19 deaths shown as points. The red line represents median model estimates of daily deaths, and the shaded region represents the 95% quantiles of the model estimated deaths. Plots of the cumulative estimated deaths are also shown, with the cumulative number of reported deaths represented by a dashed black line.

Kuwait

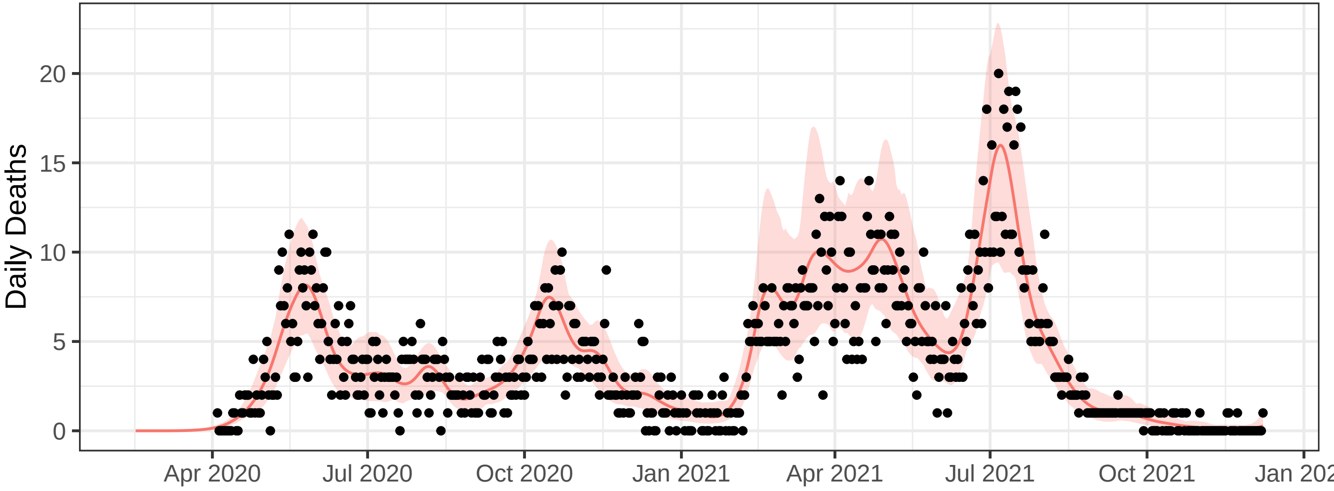

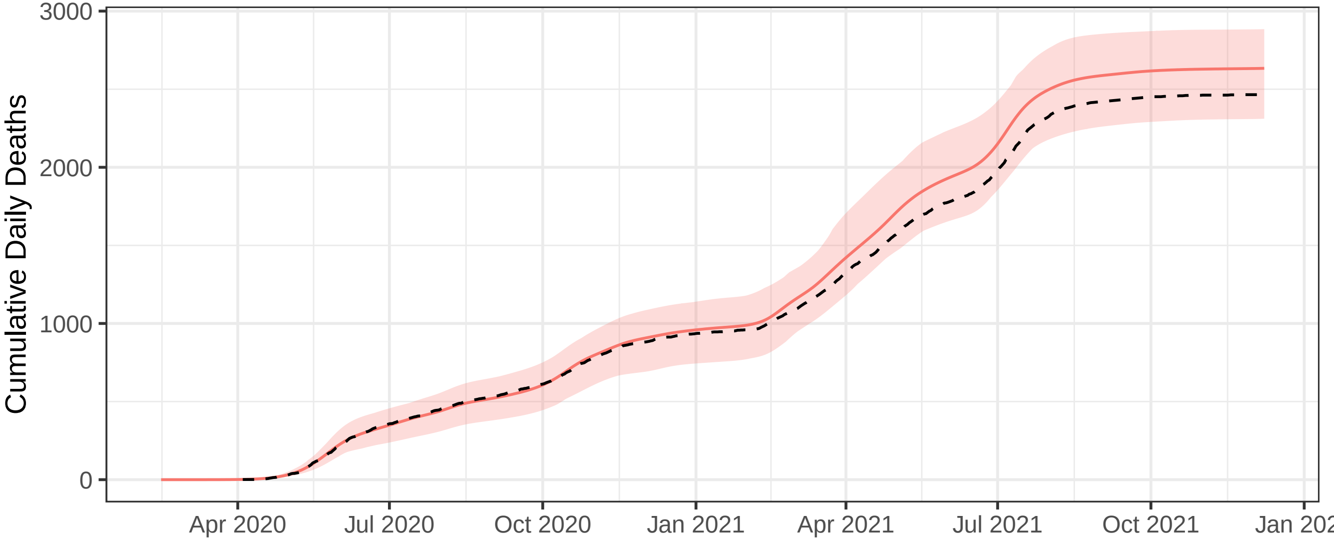

Daily deaths are included with reported COVID−19 deaths shown as points. The red line represents median model estimates of daily deaths, and the shaded region represents the 95% quantiles of the model estimated deaths. Plots of the cumulative estimated deaths are also shown, with the cumulative number of reported deaths represented by a dashed black line.

Lao PDR

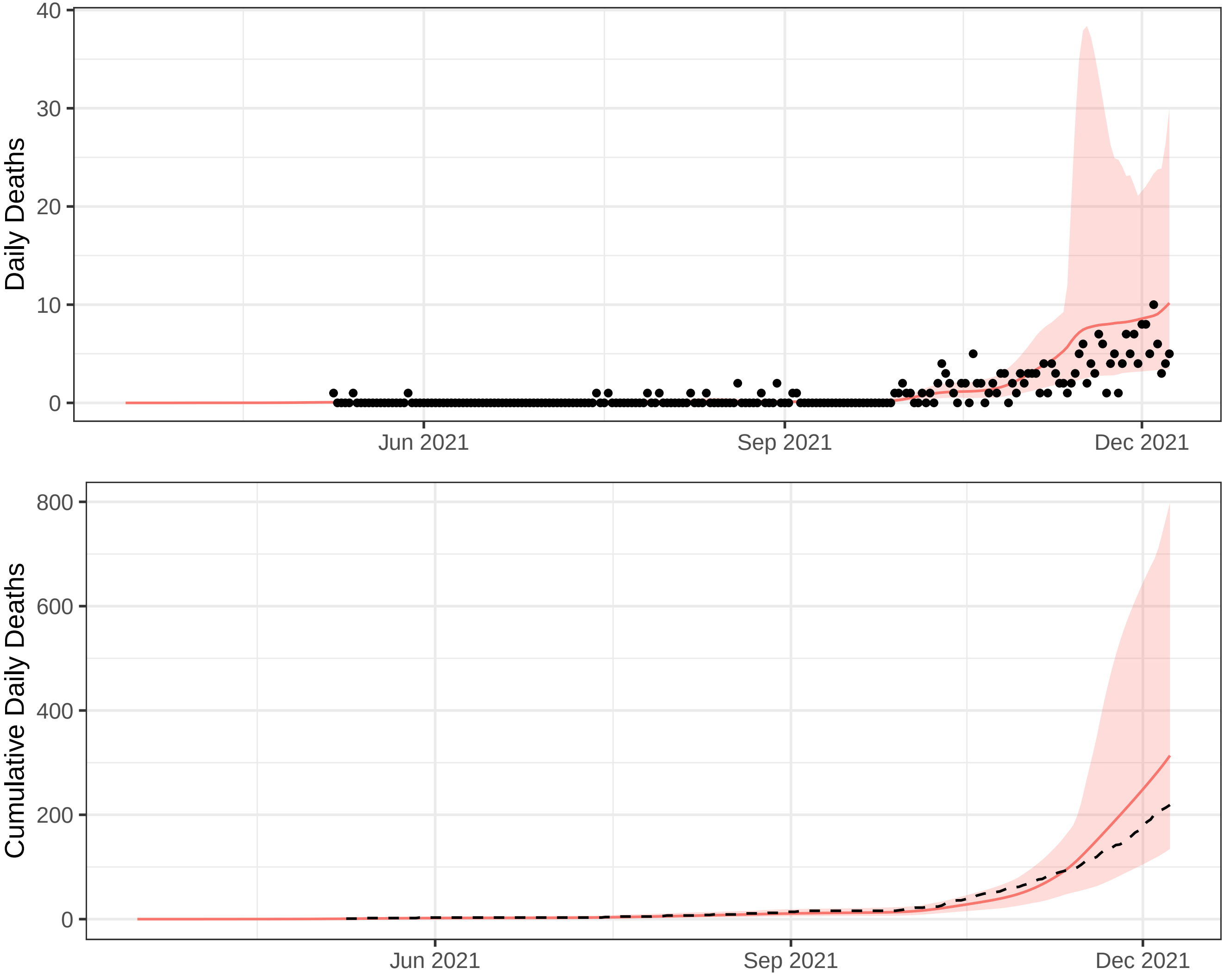

Daily deaths are included with reported COVID−19 deaths shown as points. The red line represents median model estimates of daily deaths, and the shaded region represents the 95% quantiles of the model estimated deaths. Plots of the cumulative estimated deaths are also shown, with the cumulative number of reported deaths represented by a dashed black line.

Lebanon

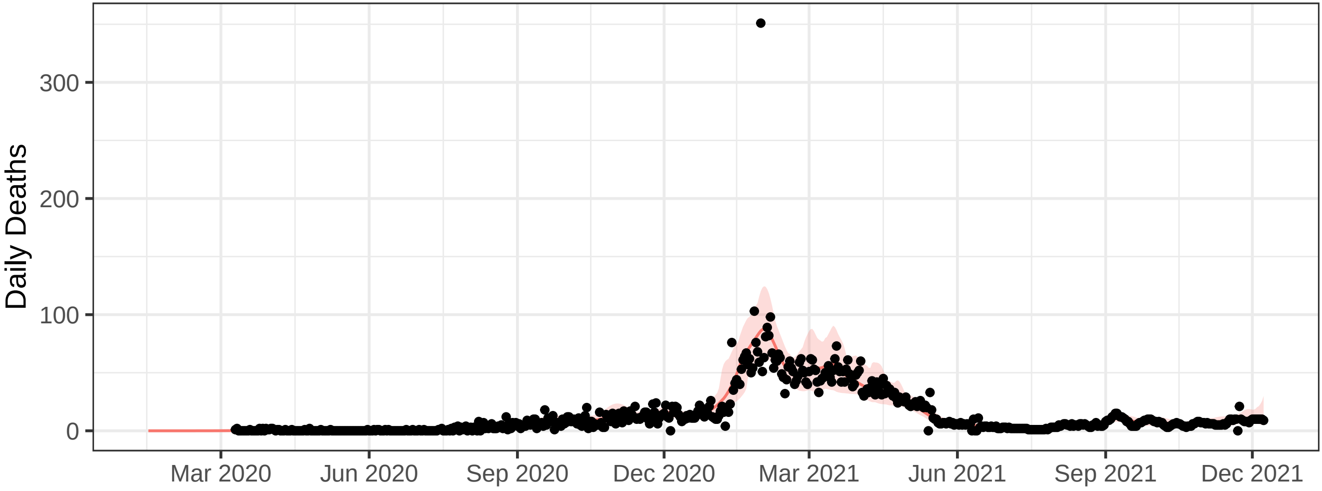

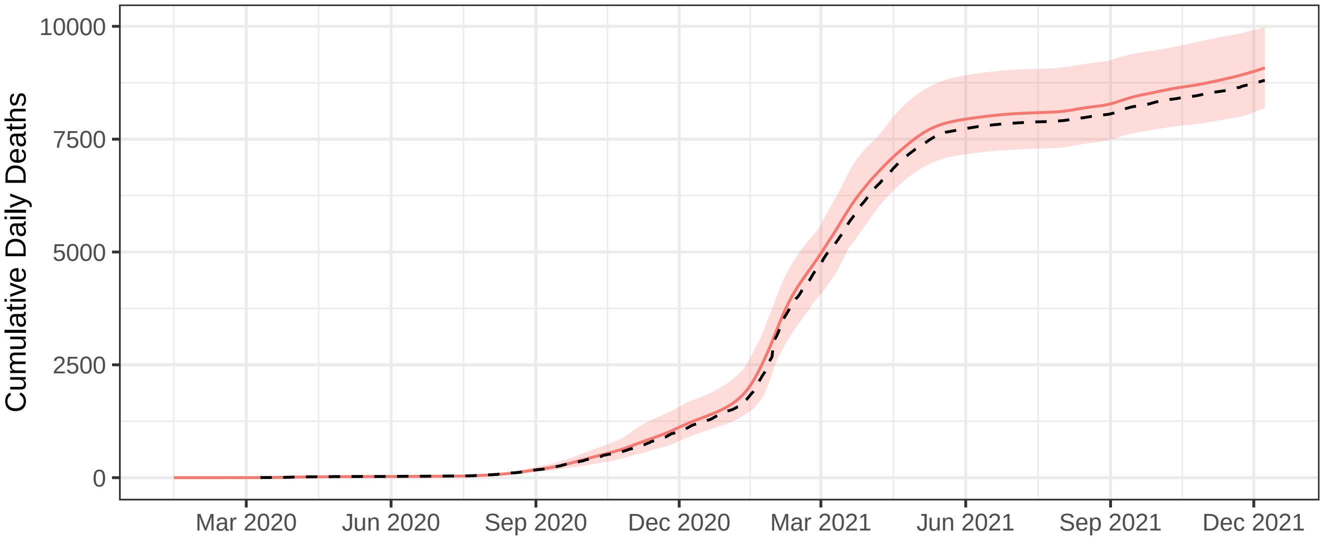

Daily deaths are included with reported COVID−19 deaths shown as points. The red line represents median model estimates of daily deaths, and the shaded region represents the 95% quantiles of the model estimated deaths. Plots of the cumulative estimated deaths are also shown, with the cumulative number of reported deaths represented by a dashed black line.

Liberia

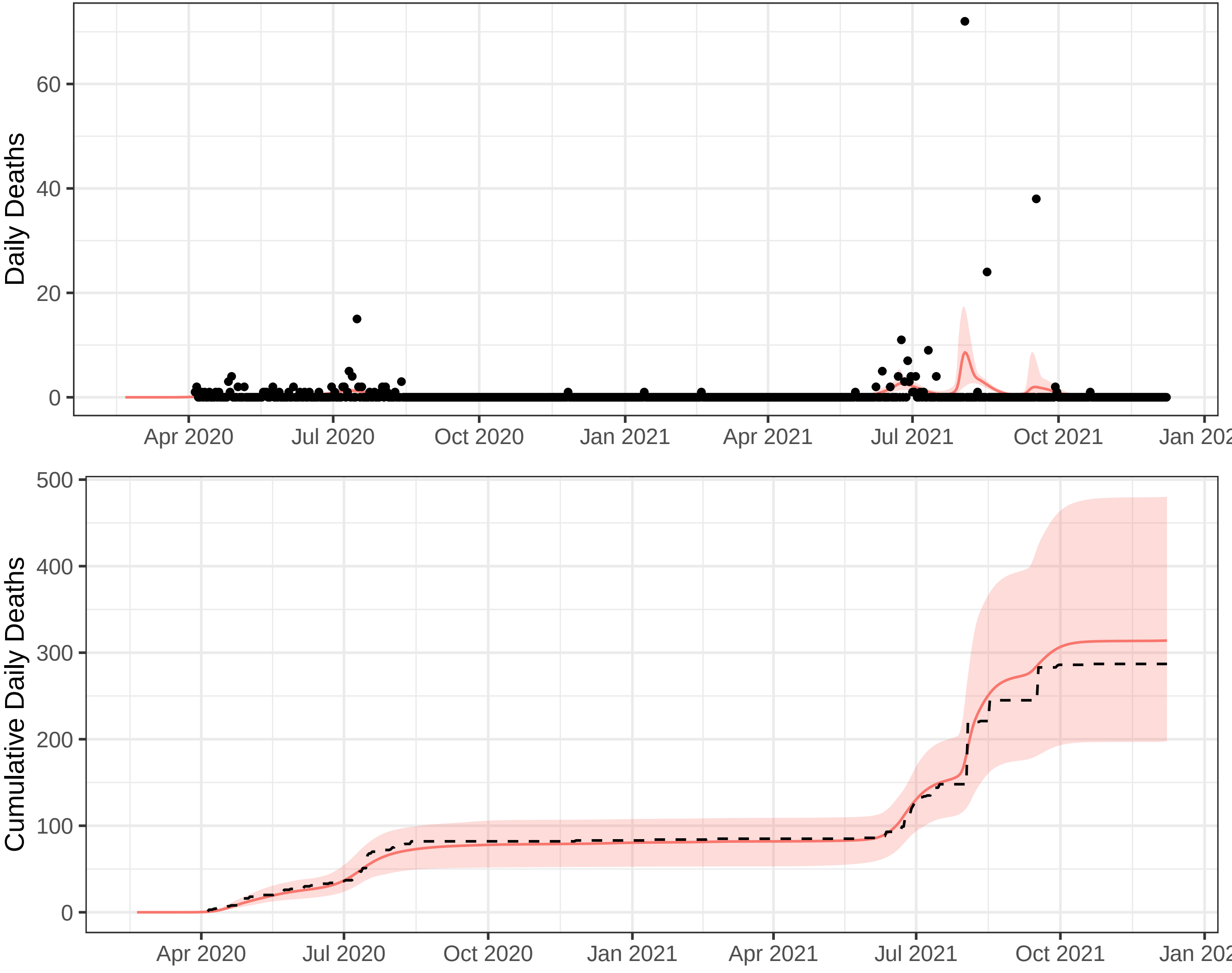

Daily deaths are included with reported COVID−19 deaths shown as points. The red line represents median model estimates of daily deaths, and the shaded region represents the 95% quantiles of the model estimated deaths. Plots of the cumulative estimated deaths are also shown, with the cumulative number of reported deaths represented by a dashed black line.

Libya

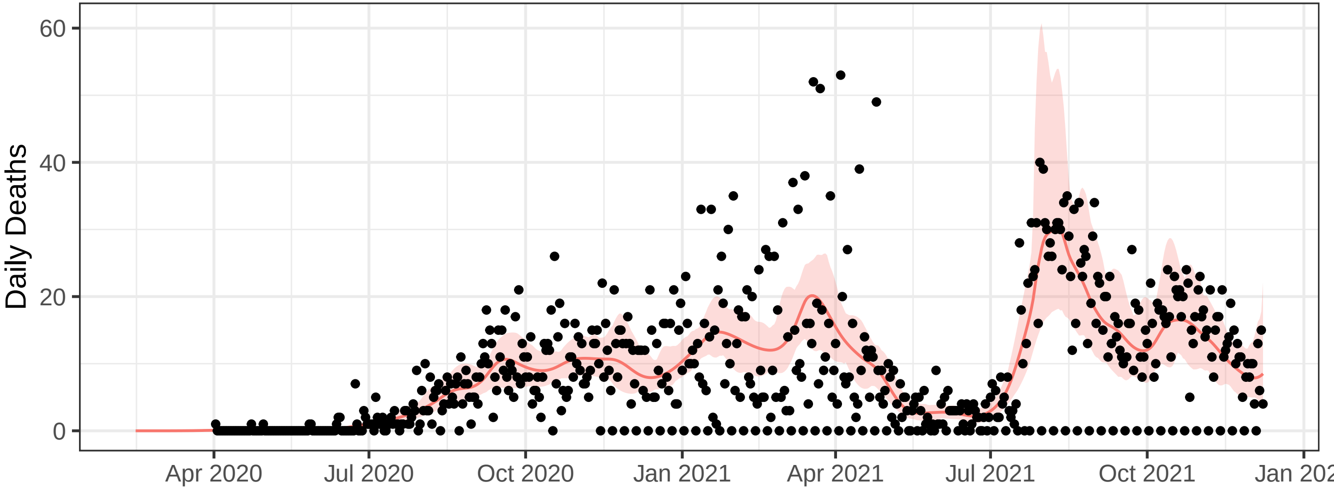

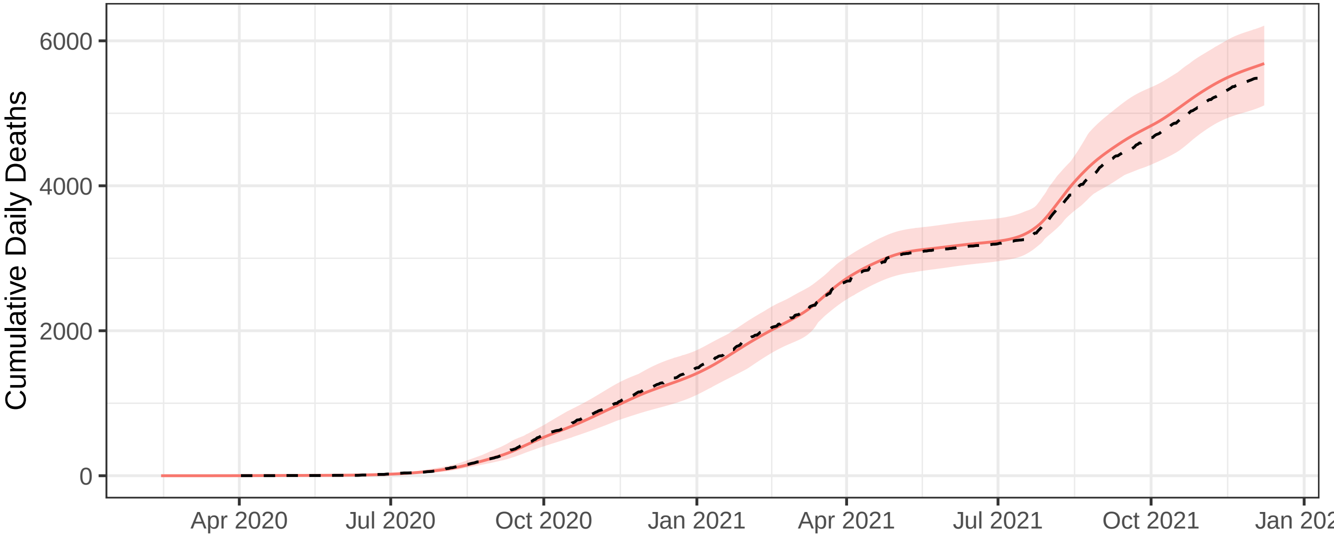

Daily deaths are included with reported COVID−19 deaths shown as points. The red line represents median model estimates of daily deaths, and the shaded region represents the 95% quantiles of the model estimated deaths. Plots of the cumulative estimated deaths are also shown, with the cumulative number of reported deaths represented by a dashed black line.

St. Lucia

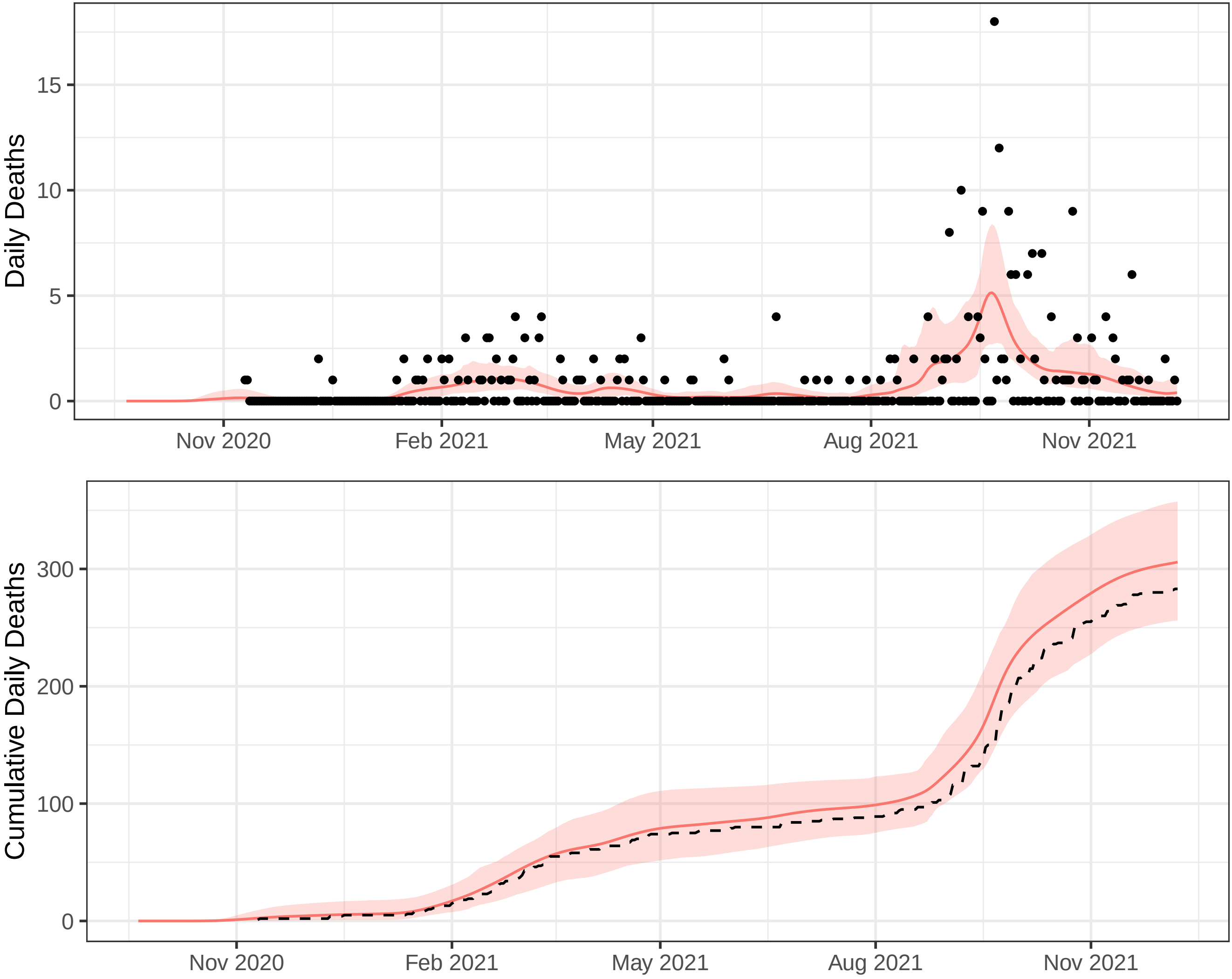

Daily deaths are included with reported COVID−19 deaths shown as points. The red line represents median model estimates of daily deaths, and the shaded region represents the 95% quantiles of the model estimated deaths. Plots of the cumulative estimated deaths are also shown, with the cumulative number of reported deaths represented by a dashed black line.

Sri Lanka

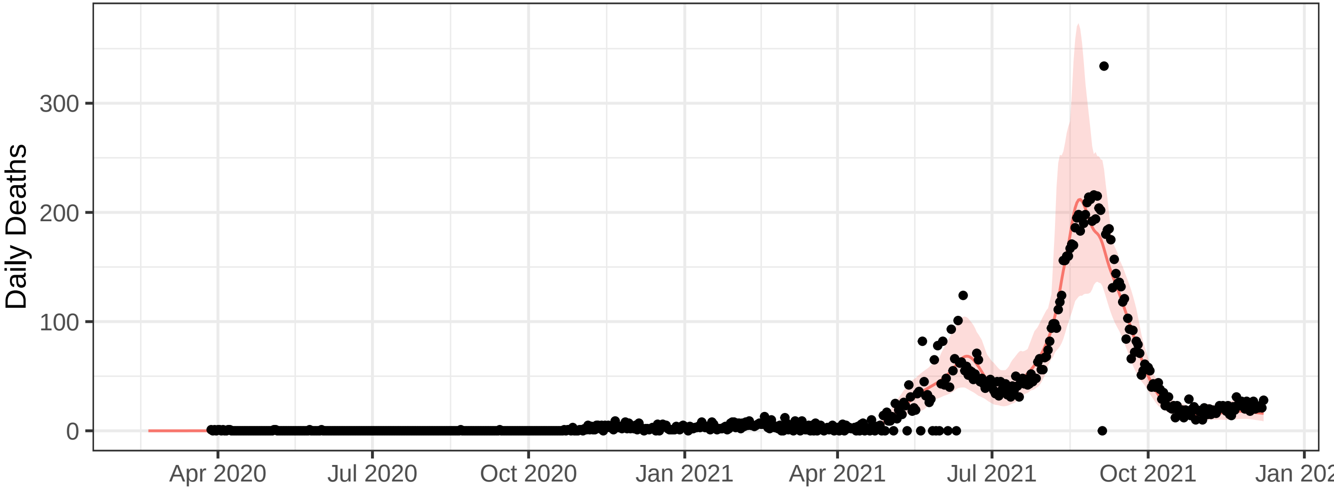

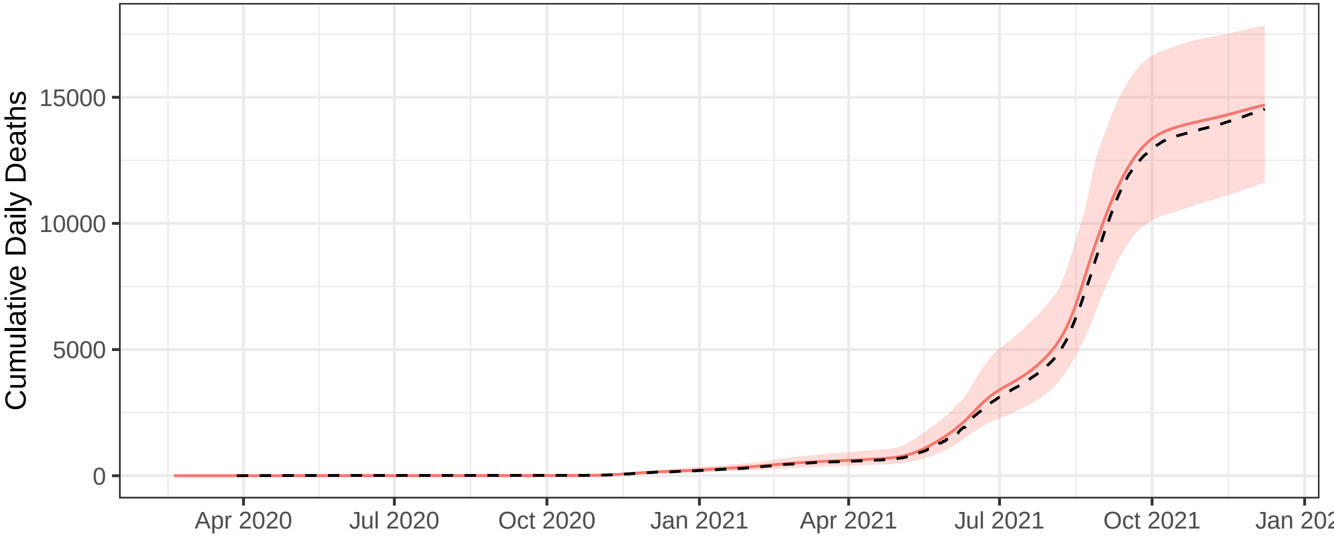

Daily deaths are included with reported COVID−19 deaths shown as points. The red line represents median model estimates of daily deaths, and the shaded region represents the 95% quantiles of the model estimated deaths. Plots of the cumulative estimated deaths are also shown, with the cumulative number of reported deaths represented by a dashed black line.

Lesotho

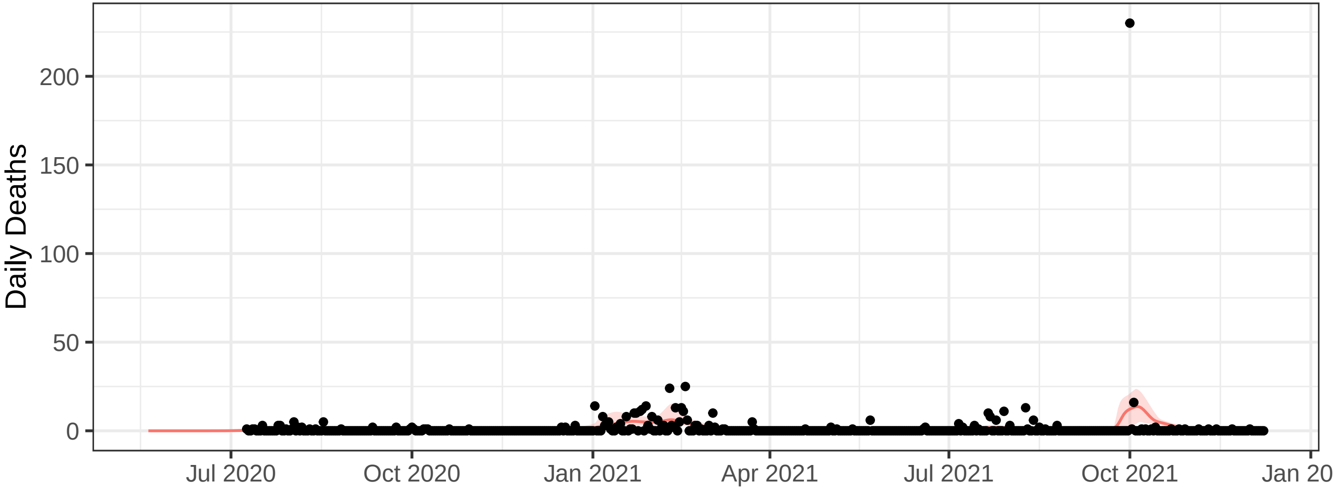

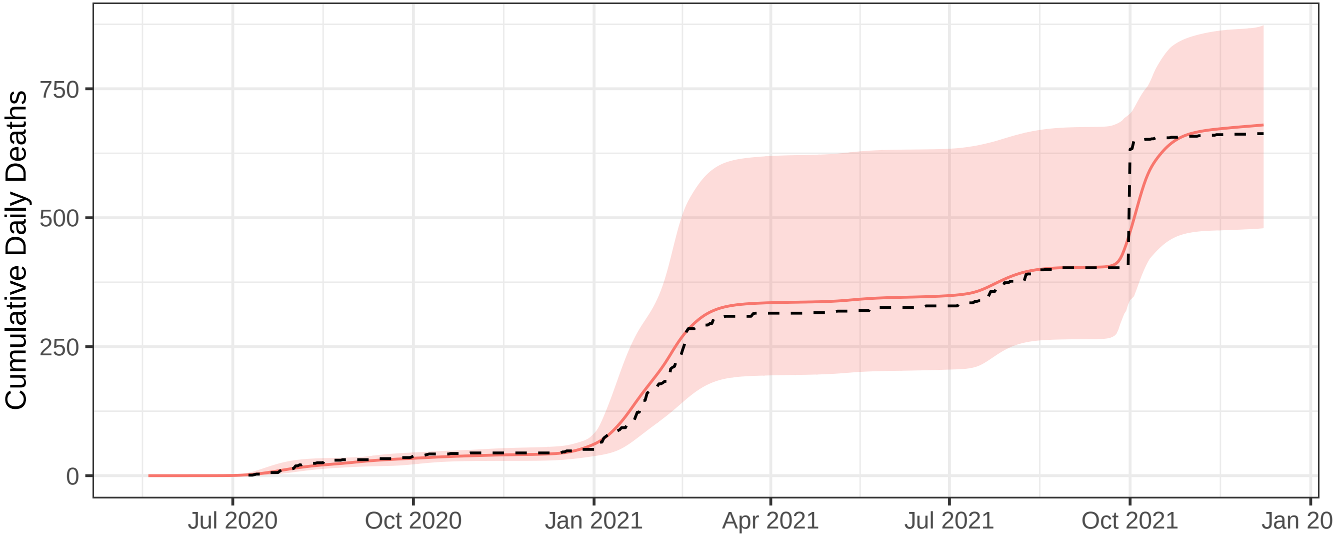

Daily deaths are included with reported COVID−19 deaths shown as points. The red line represents median model estimates of daily deaths, and the shaded region represents the 95% quantiles of the model estimated deaths. Plots of the cumulative estimated deaths are also shown, with the cumulative number of reported deaths represented by a dashed black line.

Lithuania

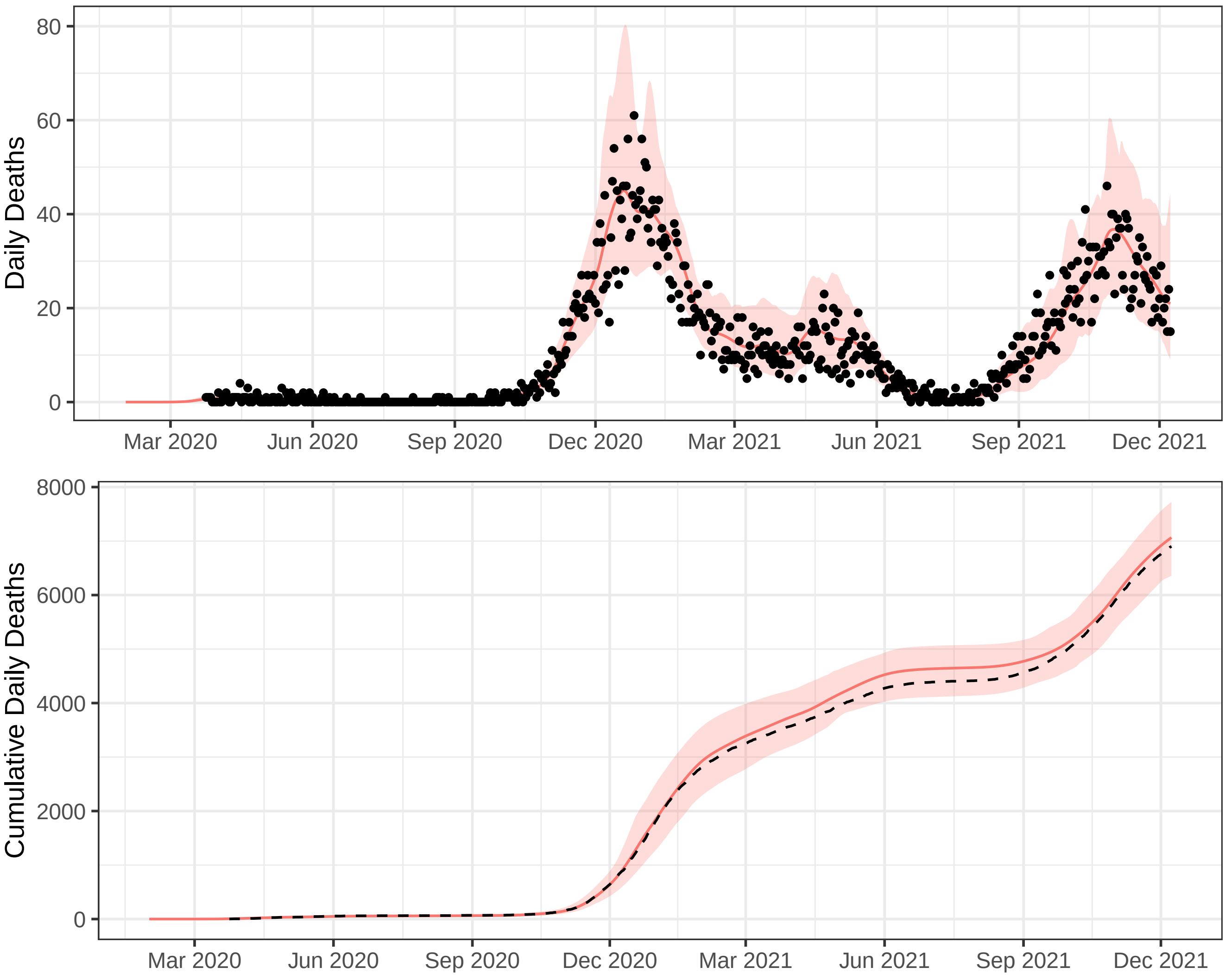

Daily deaths are included with reported COVID−19 deaths shown as points. The red line represents median model estimates of daily deaths, and the shaded region represents the 95% quantiles of the model estimated deaths. Plots of the cumulative estimated deaths are also shown, with the cumulative number of reported deaths represented by a dashed black line.

Luxembourg

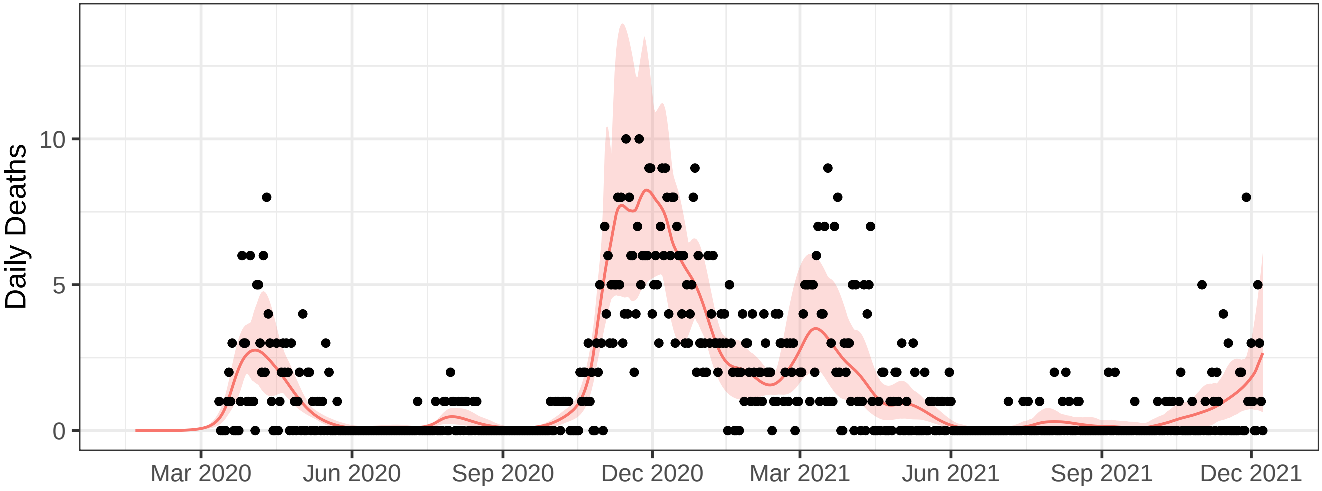

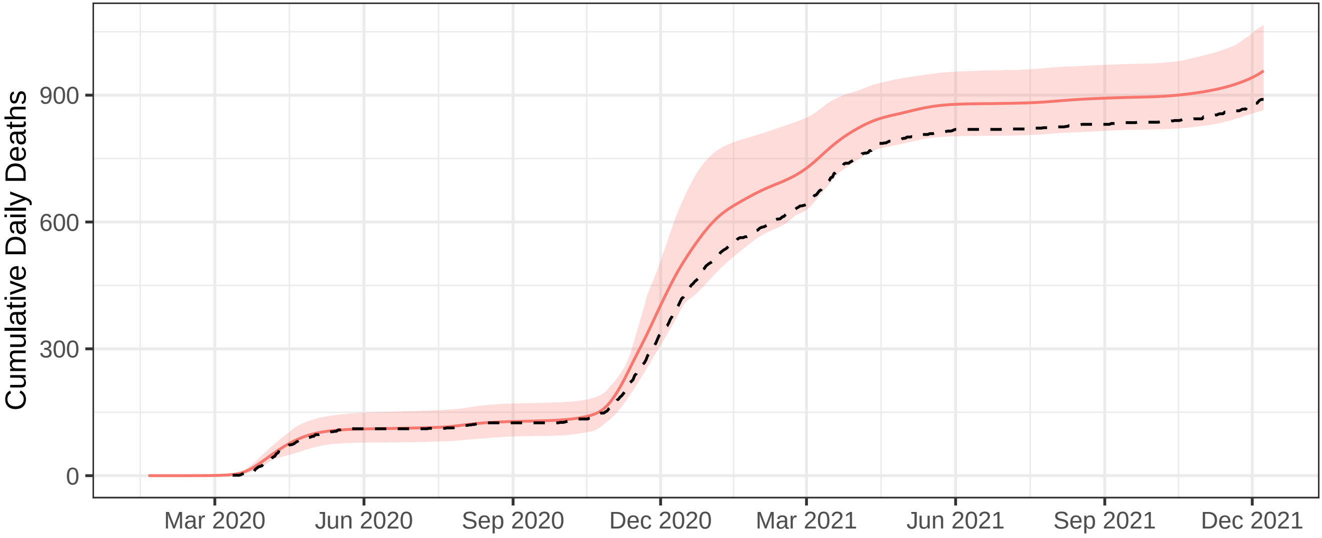

Daily deaths are included with reported COVID−19 deaths shown as points. The red line represents median model estimates of daily deaths, and the shaded region represents the 95% quantiles of the model estimated deaths. Plots of the cumulative estimated deaths are also shown, with the cumulative number of reported deaths represented by a dashed black line.

Latvia

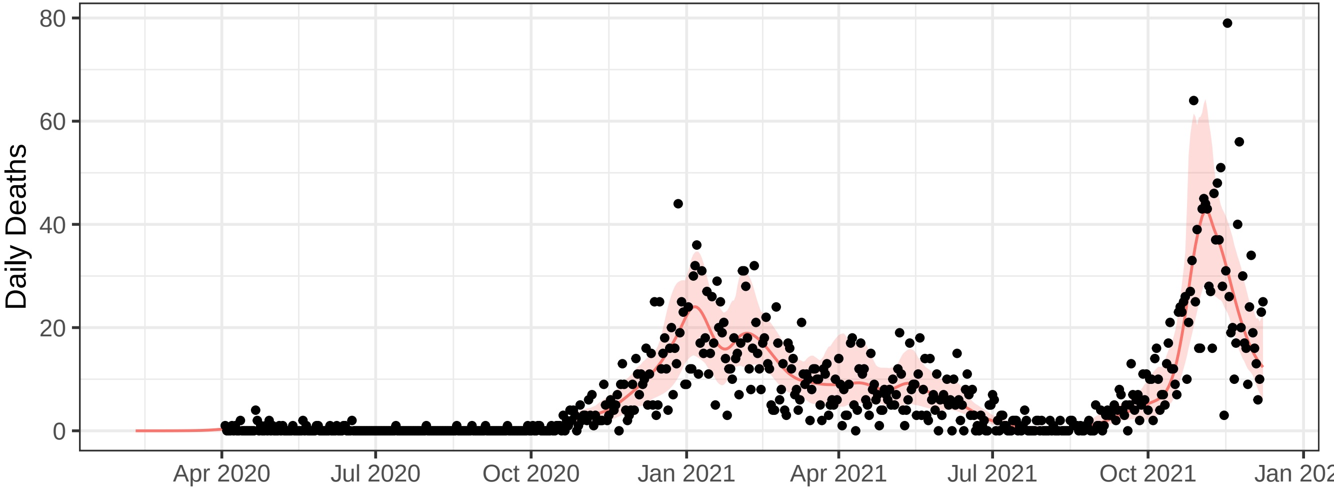

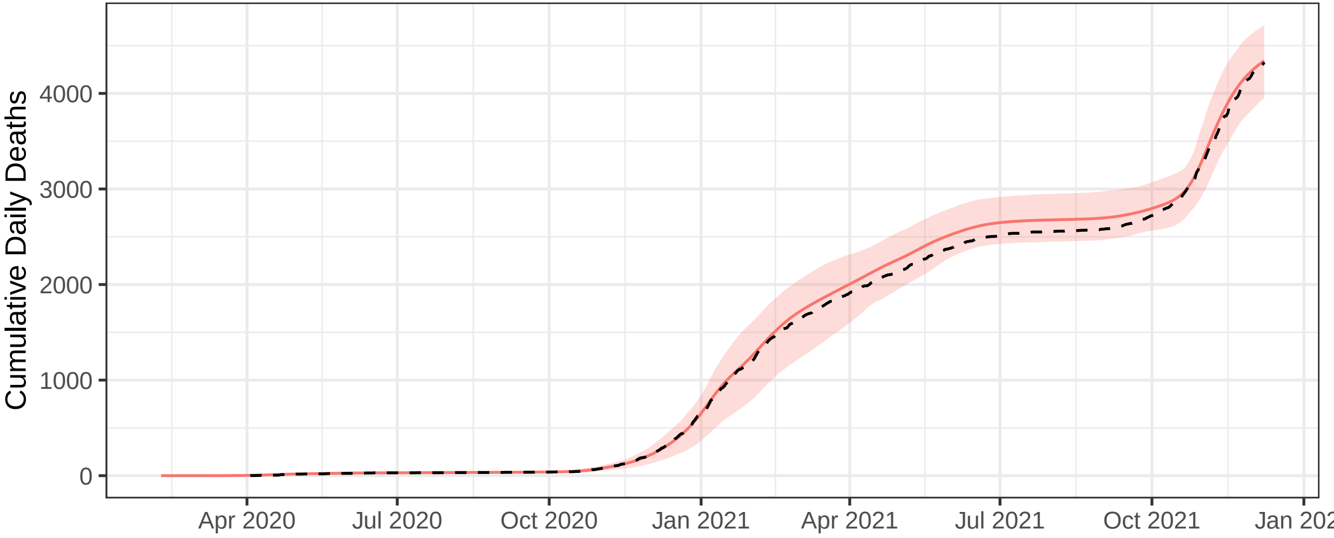

Daily deaths are included with reported COVID−19 deaths shown as points. The red line represents median model estimates of daily deaths, and the shaded region represents the 95% quantiles of the model estimated deaths. Plots of the cumulative estimated deaths are also shown, with the cumulative number of reported deaths represented by a dashed black line.

Morocco

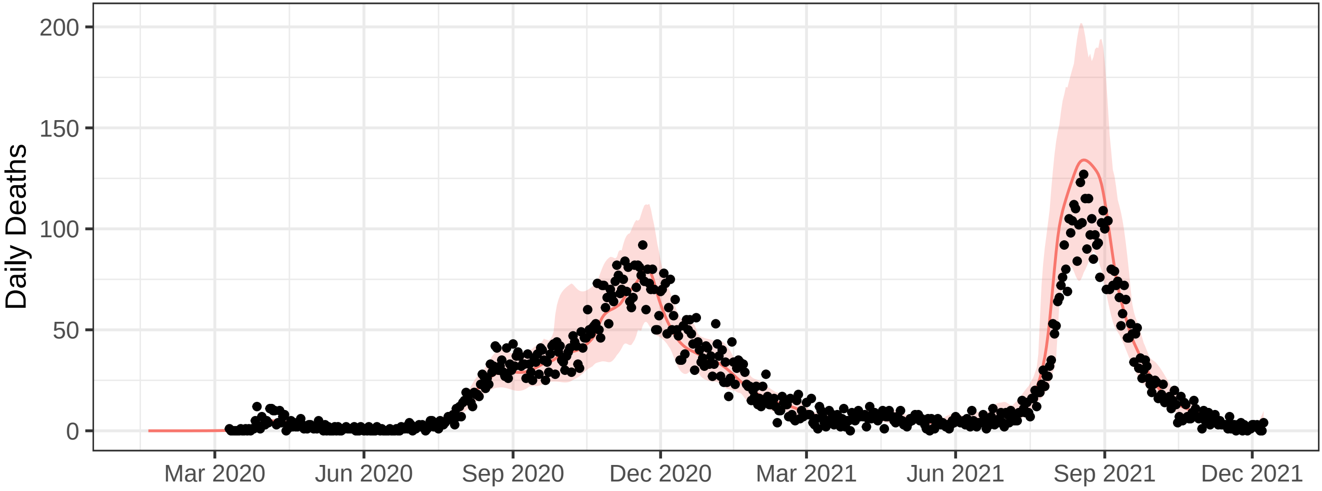

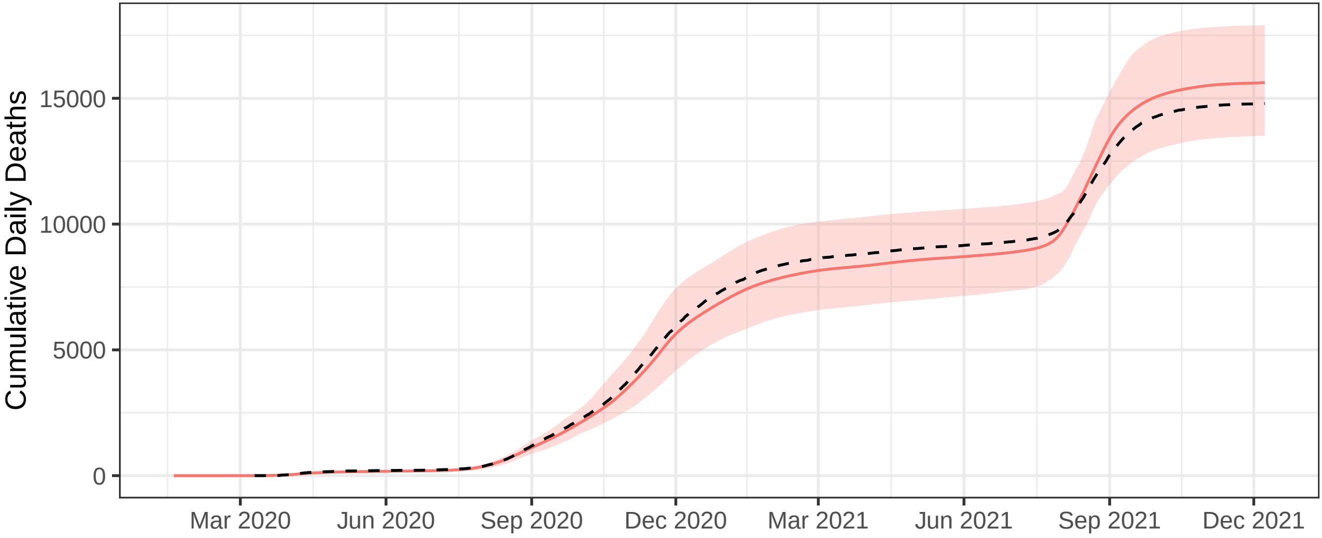

Daily deaths are included with reported COVID−19 deaths shown as points. The red line represents median model estimates of daily deaths, and the shaded region represents the 95% quantiles of the model estimated deaths. Plots of the cumulative estimated deaths are also shown, with the cumulative number of reported deaths represented by a dashed black line.

Moldova

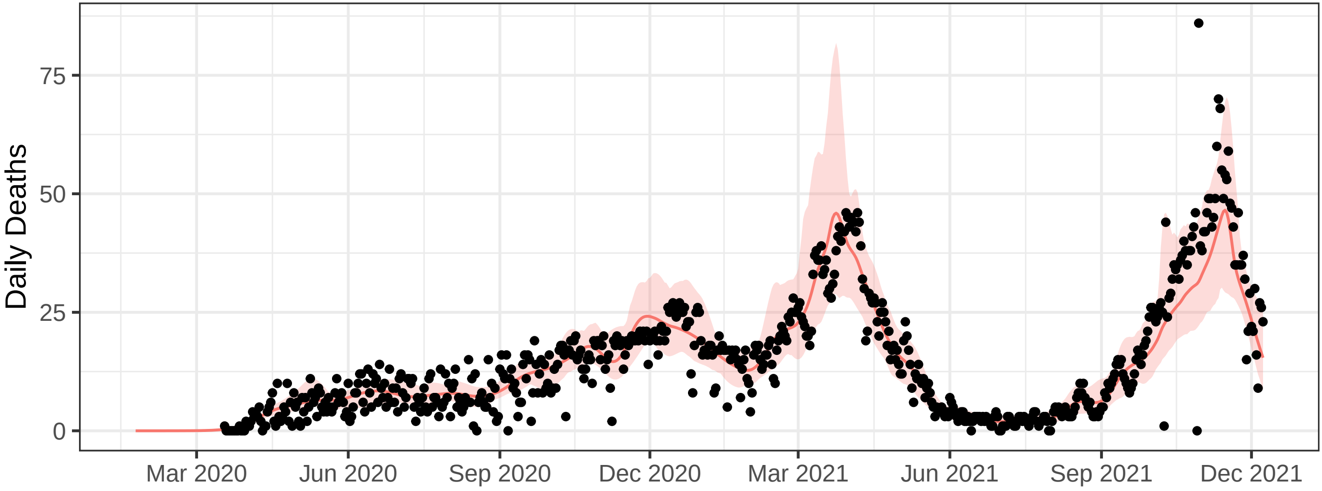

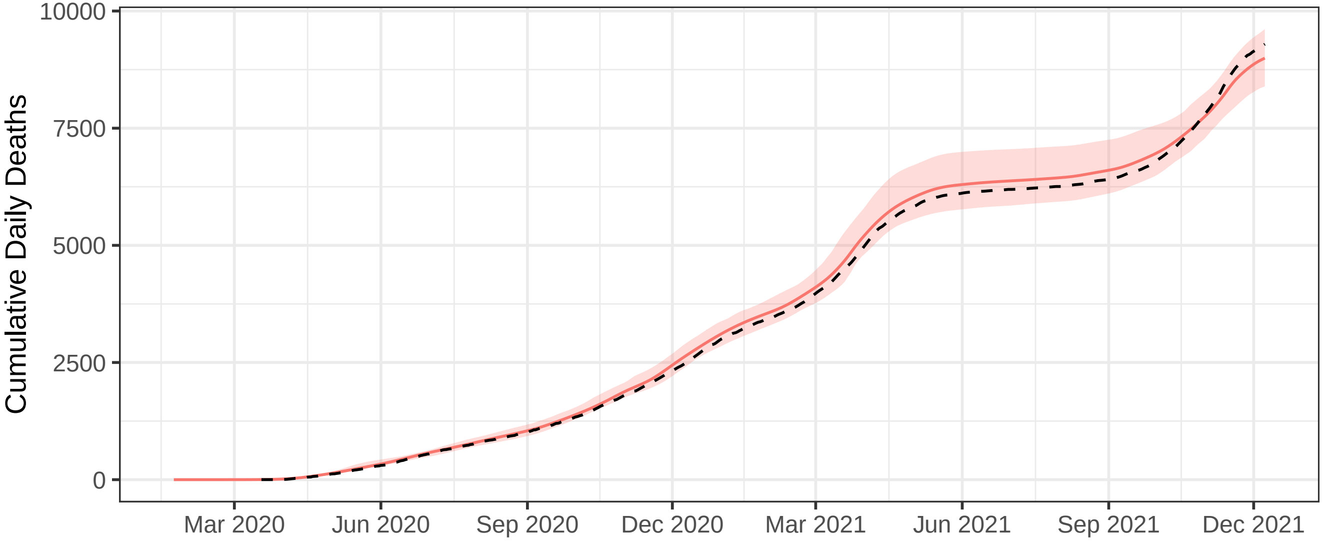

Daily deaths are included with reported COVID−19 deaths shown as points. The red line represents median model estimates of daily deaths, and the shaded region represents the 95% quantiles of the model estimated deaths. Plots of the cumulative estimated deaths are also shown, with the cumulative number of reported deaths represented by a dashed black line.

Madagascar

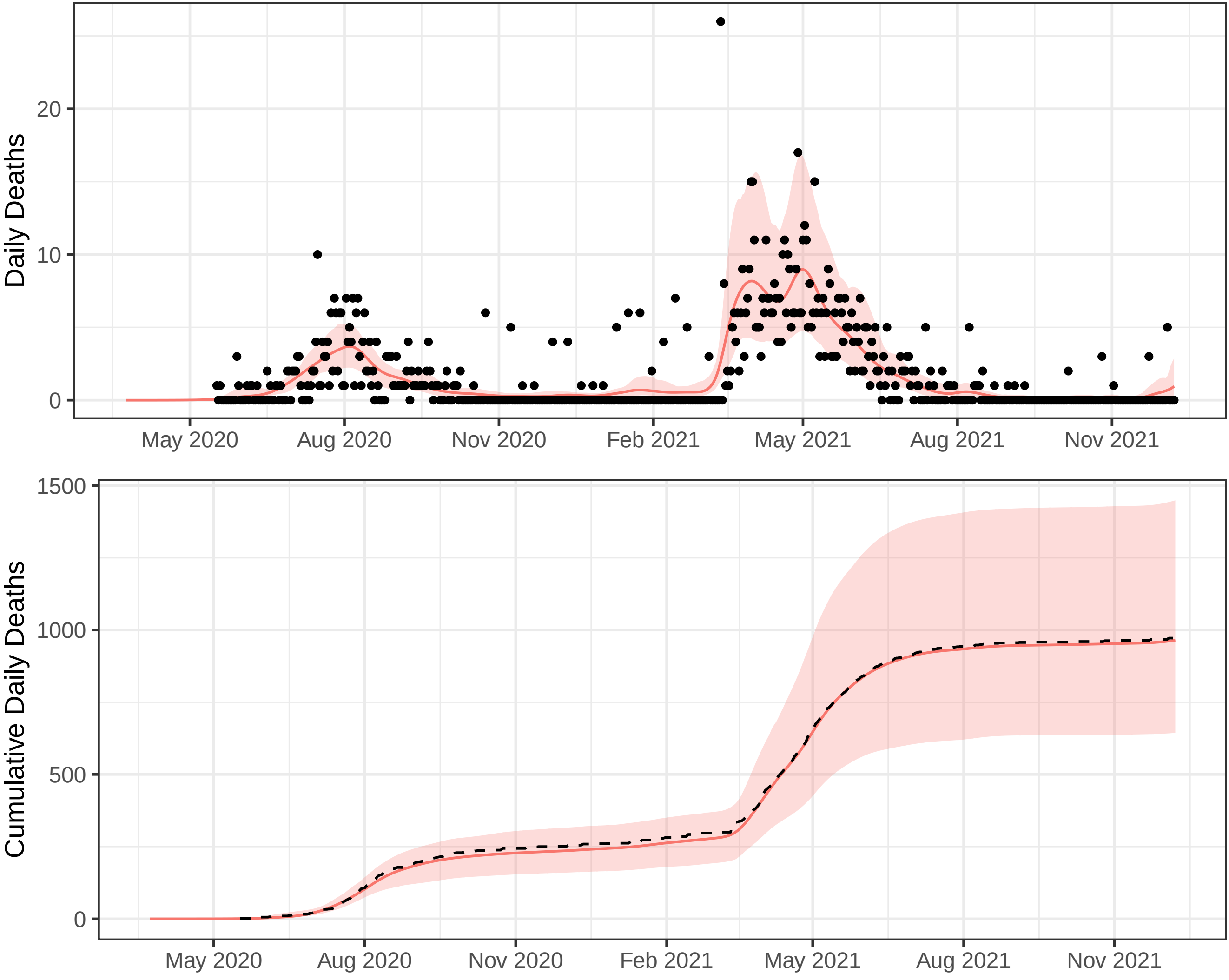

Daily deaths are included with reported COVID−19 deaths shown as points. The red line represents median model estimates of daily deaths, and the shaded region represents the 95% quantiles of the model estimated deaths. Plots of the cumulative estimated deaths are also shown, with the cumulative number of reported deaths represented by a dashed black line.

Maldives

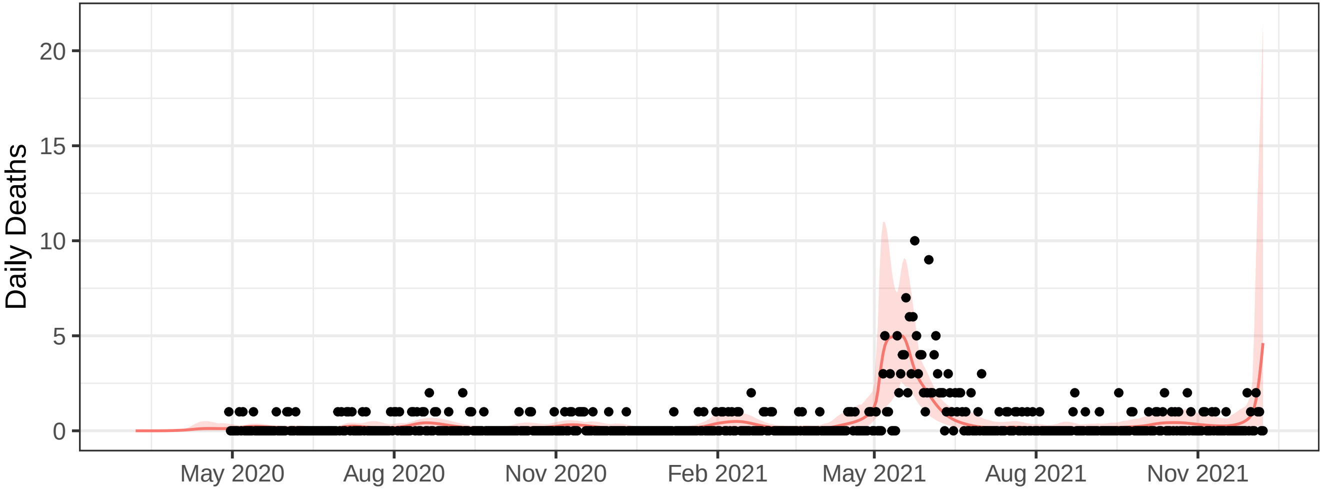

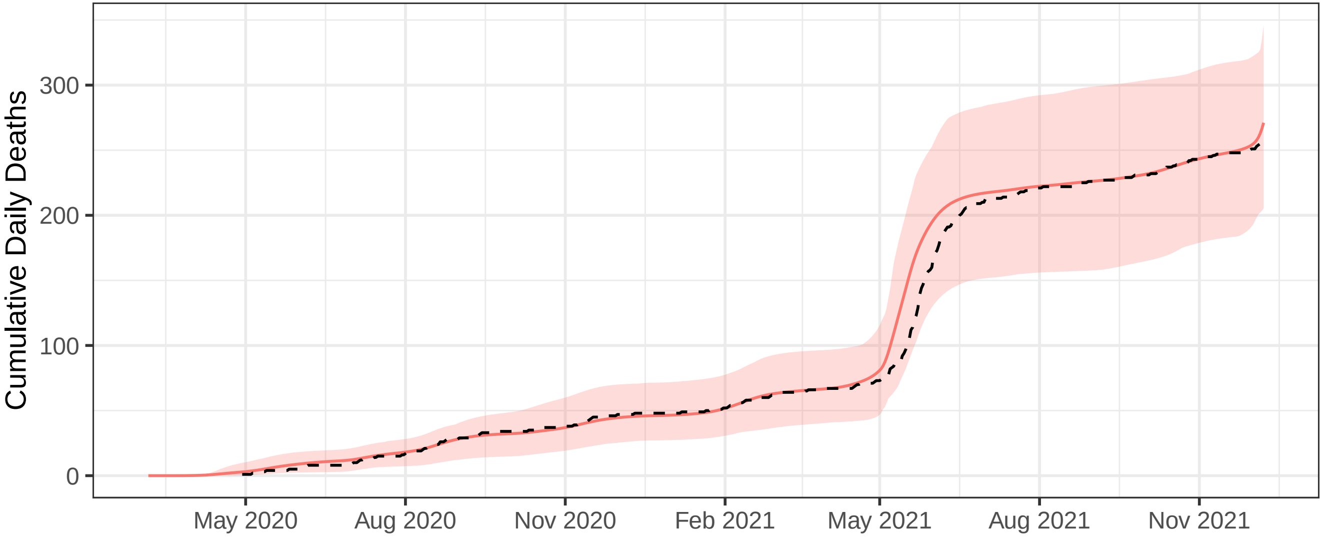

Daily deaths are included with reported COVID−19 deaths shown as points. The red line represents median model estimates of daily deaths, and the shaded region represents the 95% quantiles of the model estimated deaths. Plots of the cumulative estimated deaths are also shown, with the cumulative number of reported deaths represented by a dashed black line.

Mexico

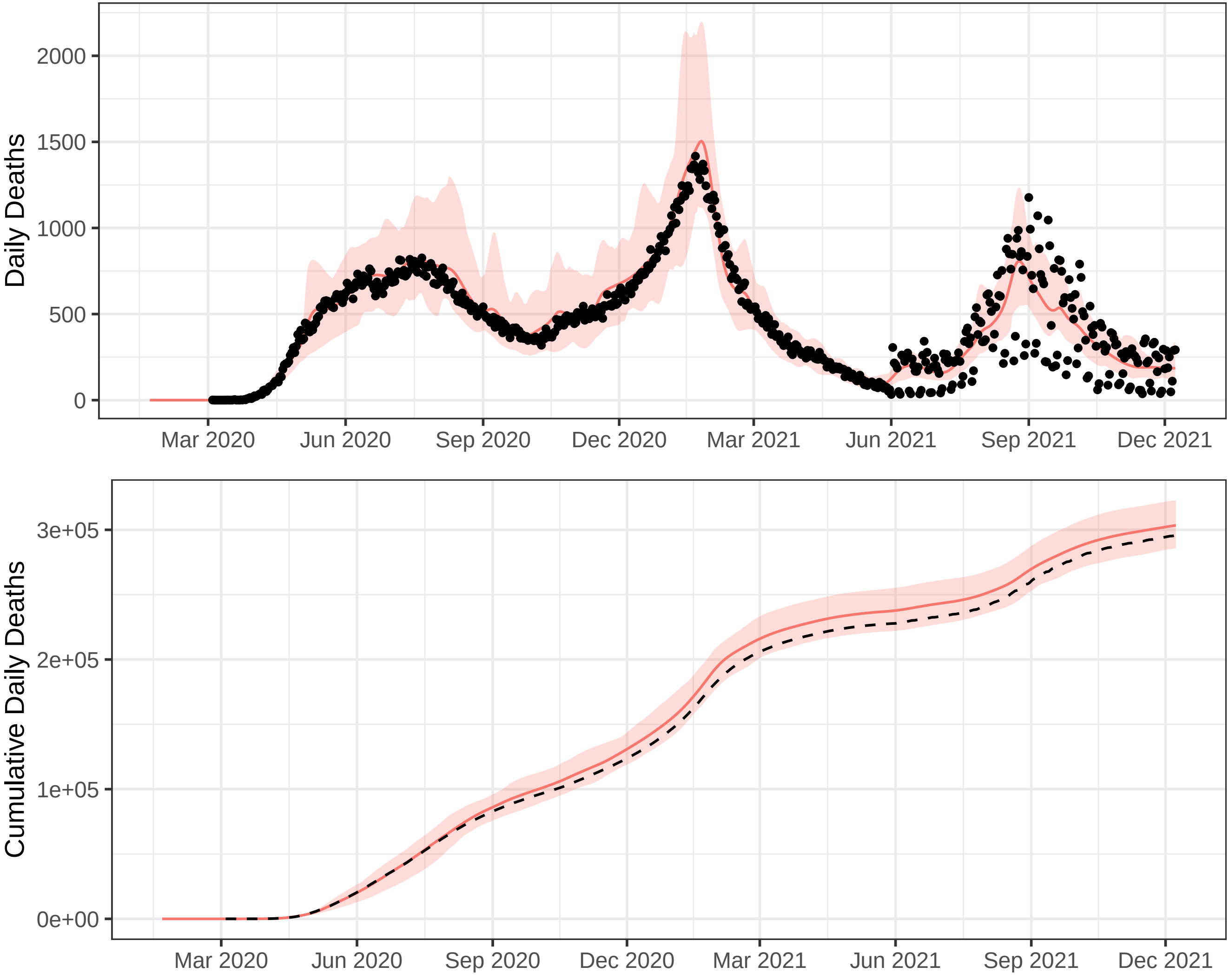

Daily deaths are included with reported COVID−19 deaths shown as points. The red line represents median model estimates of daily deaths, and the shaded region represents the 95% quantiles of the model estimated deaths. Plots of the cumulative estimated deaths are also shown, with the cumulative number of reported deaths represented by a dashed black line.

North Macedonia

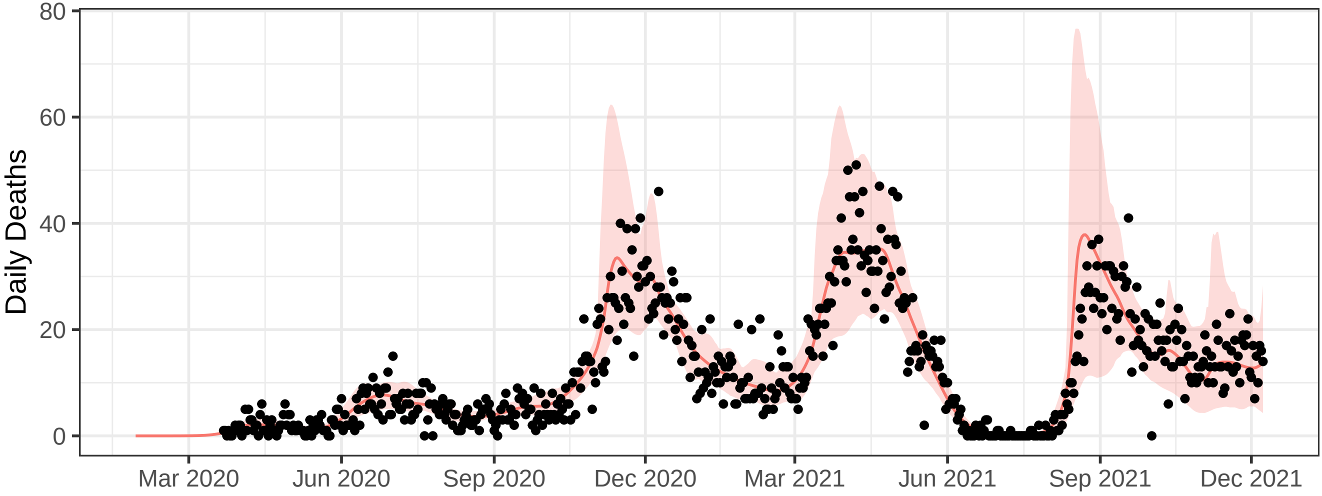

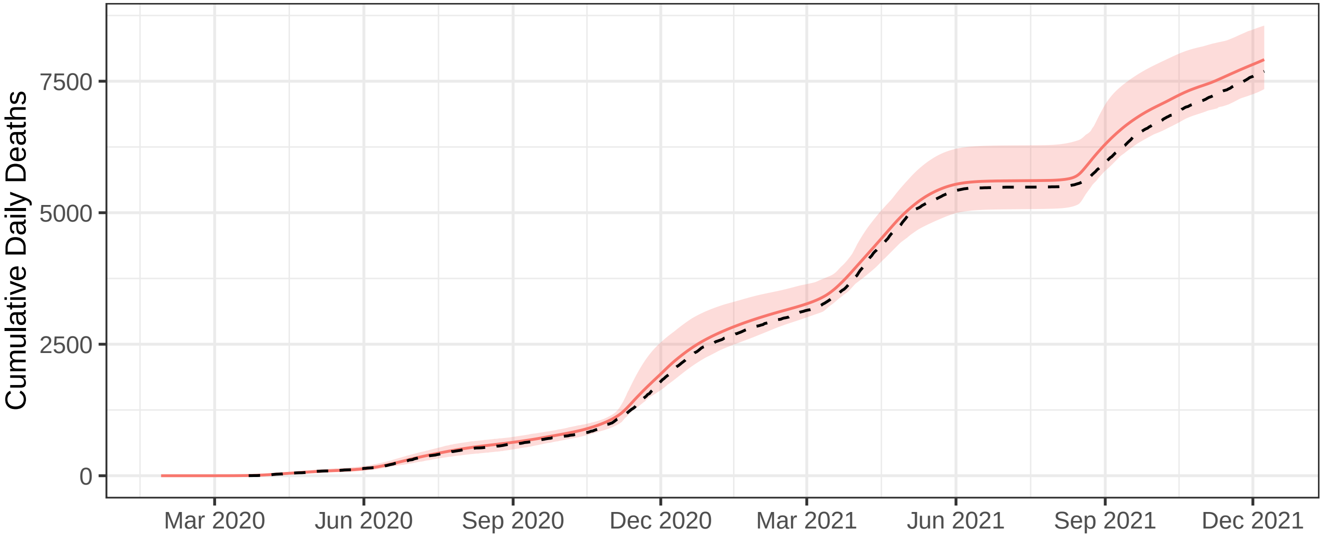

Daily deaths are included with reported COVID−19 deaths shown as points. The red line represents median model estimates of daily deaths, and the shaded region represents the 95% quantiles of the model estimated deaths. Plots of the cumulative estimated deaths are also shown, with the cumulative number of reported deaths represented by a dashed black line.

Mali

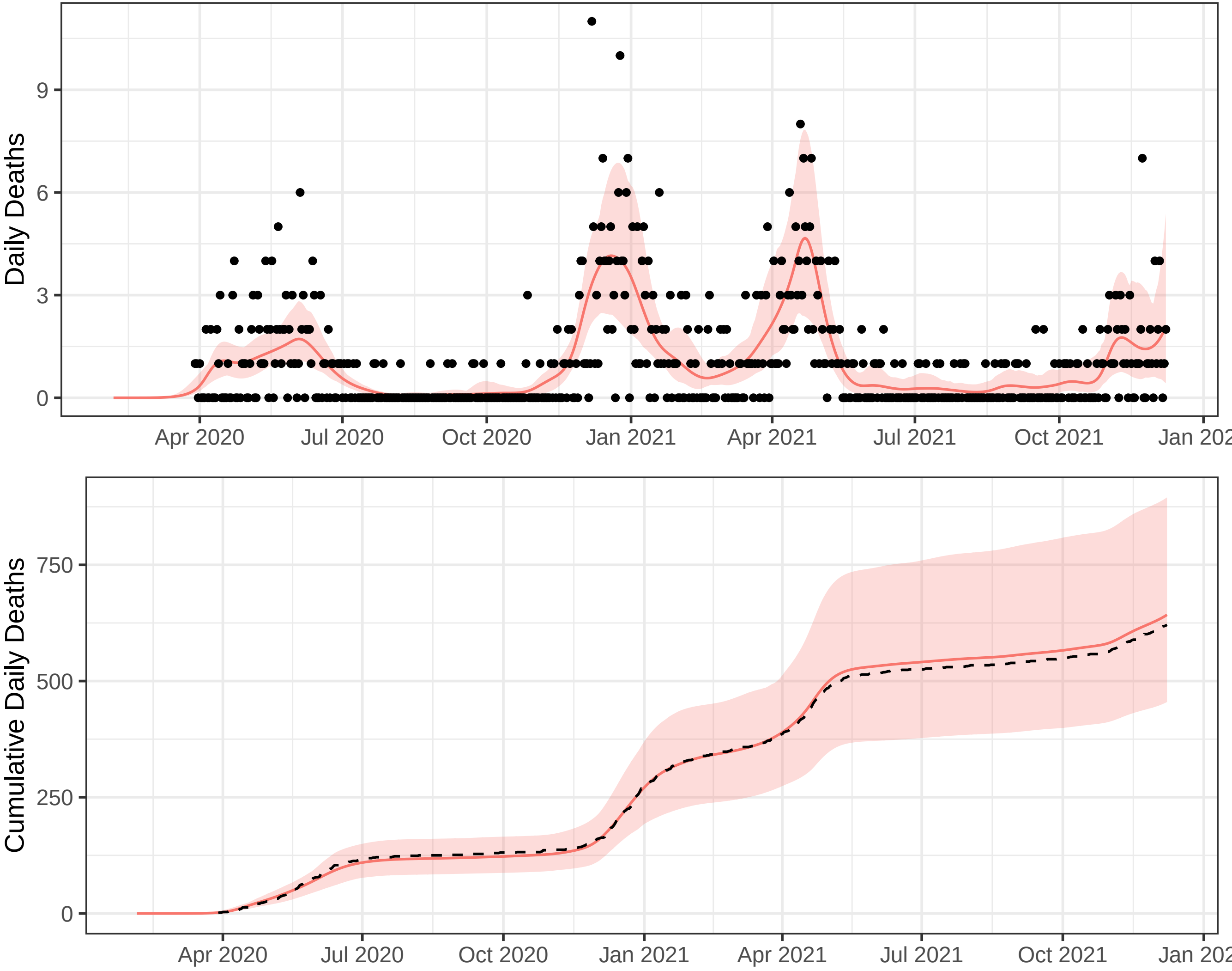

Daily deaths are included with reported COVID−19 deaths shown as points. The red line represents median model estimates of daily deaths, and the shaded region represents the 95% quantiles of the model estimated deaths. Plots of the cumulative estimated deaths are also shown, with the cumulative number of reported deaths represented by a dashed black line.

Malta

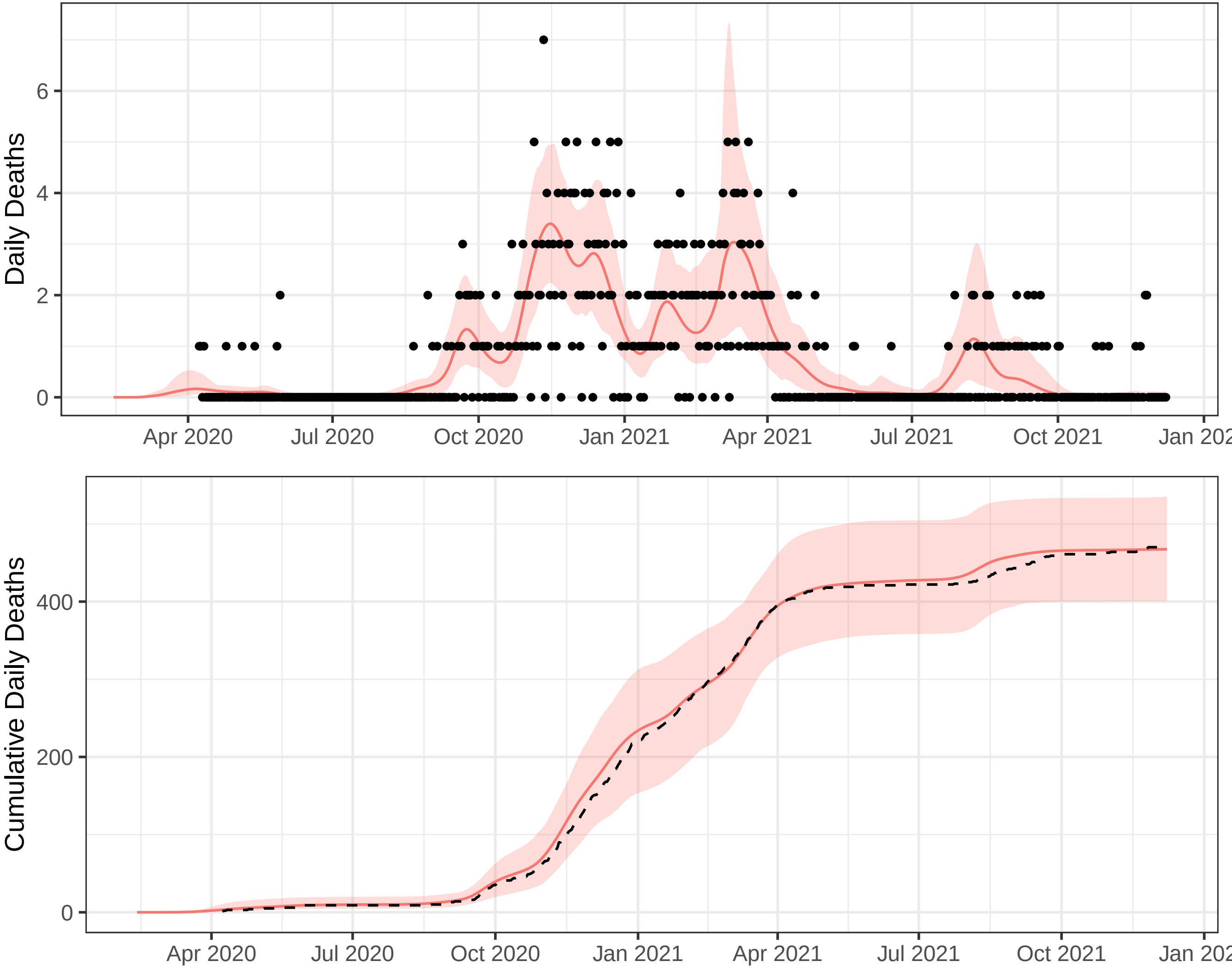

Daily deaths are included with reported COVID−19 deaths shown as points. The red line represents median model estimates of daily deaths, and the shaded region represents the 95% quantiles of the model estimated deaths. Plots of the cumulative estimated deaths are also shown, with the cumulative number of reported deaths represented by a dashed black line.

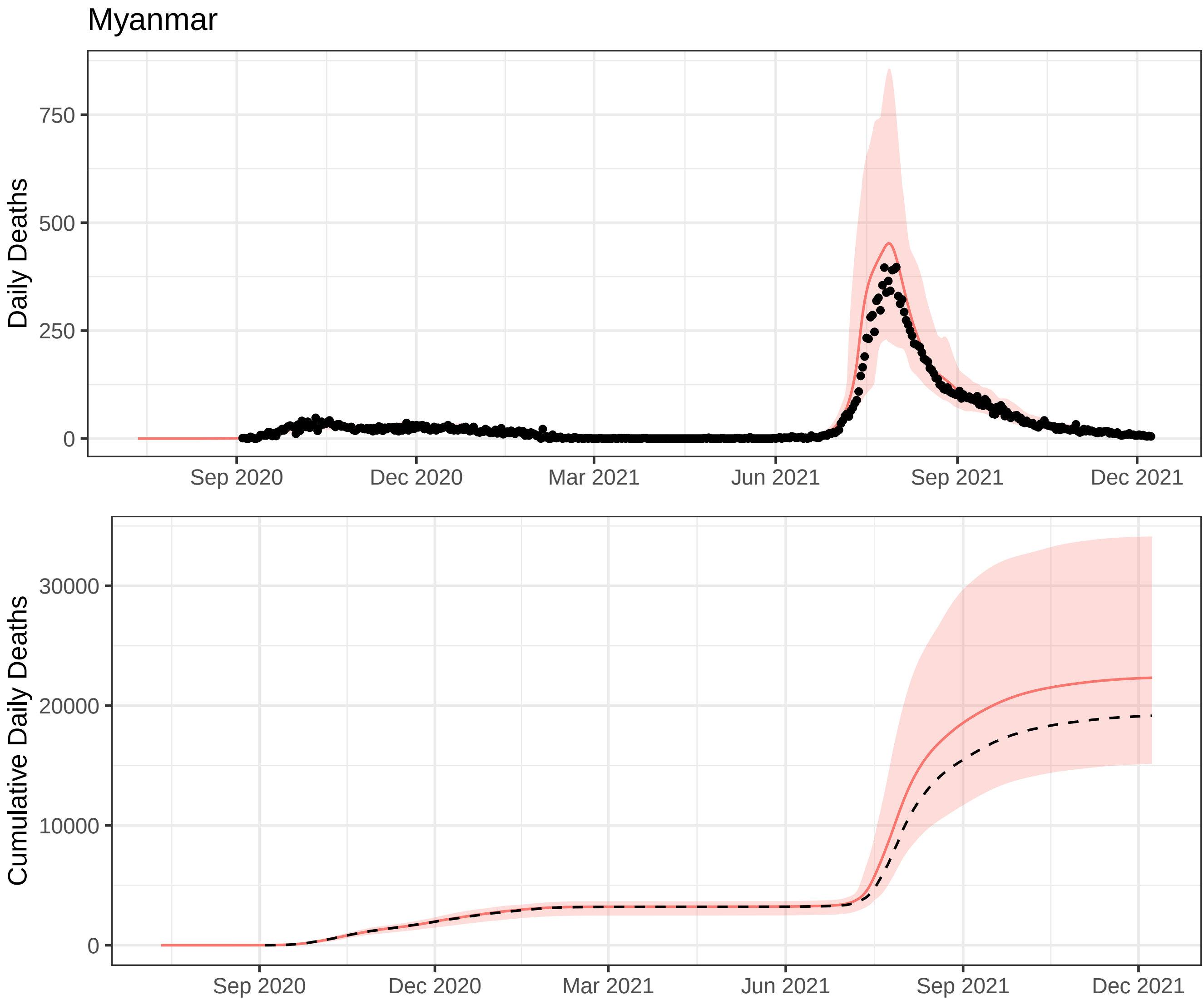

Daily deaths are included with reported COVID−19 deaths shown as points. The red line represents median model estimates of daily deaths, and the shaded region represents the 95% quantiles of the model estimated deaths. Plots of the cumulative estimated deaths are also shown, with the cumulative number of reported deaths represented by a dashed black line.

Montenegro

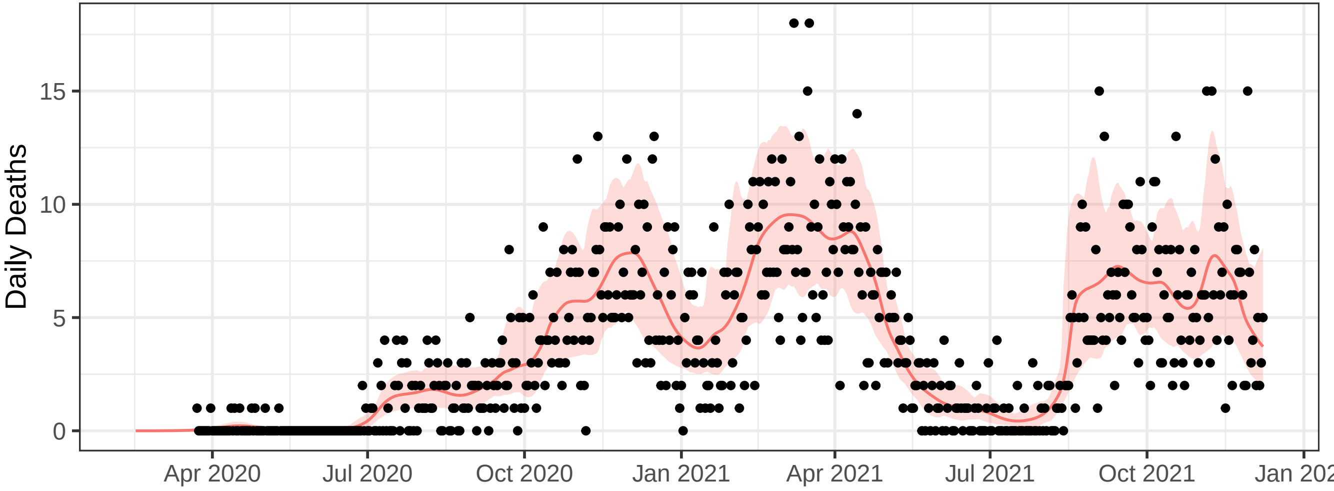

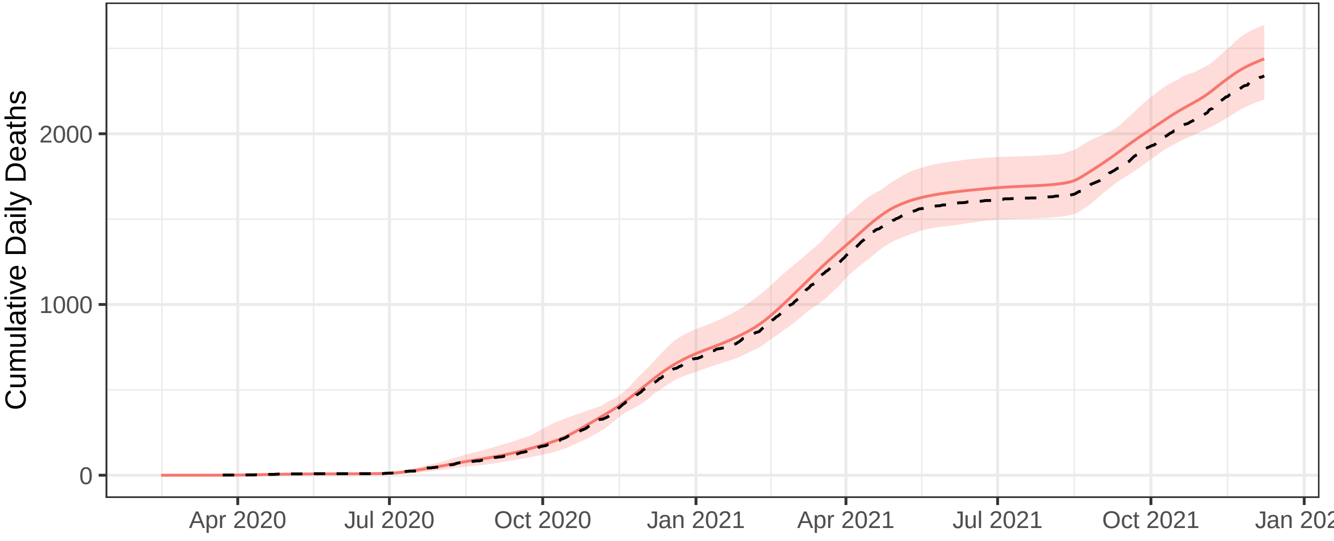

Daily deaths are included with reported COVID−19 deaths shown as points. The red line represents median model estimates of daily deaths, and the shaded region represents the 95% quantiles of the model estimated deaths. Plots of the cumulative estimated deaths are also shown, with the cumulative number of reported deaths represented by a dashed black line.

Mongolia

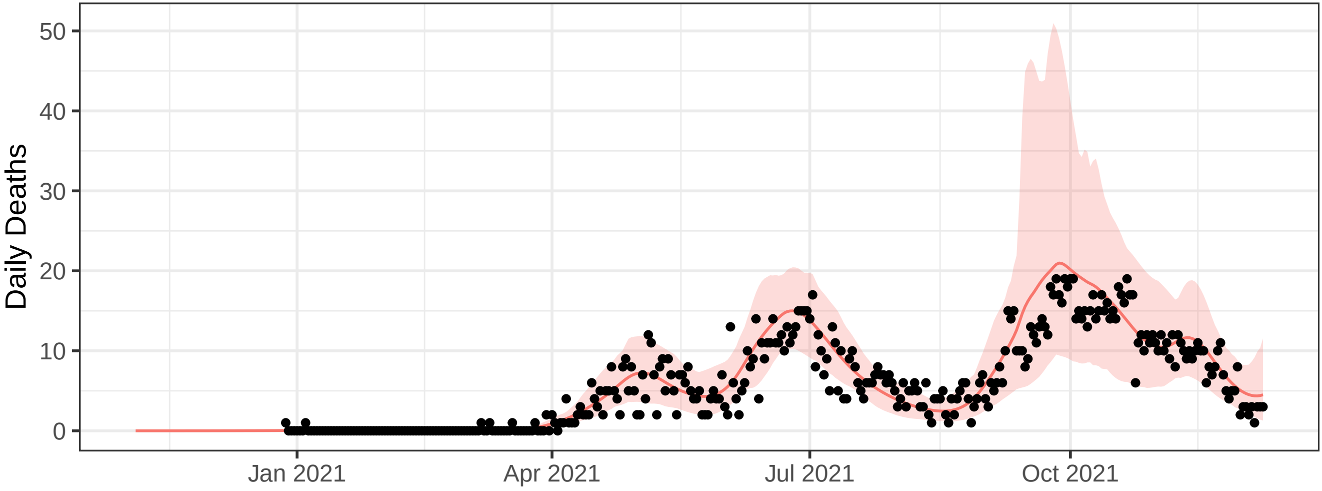

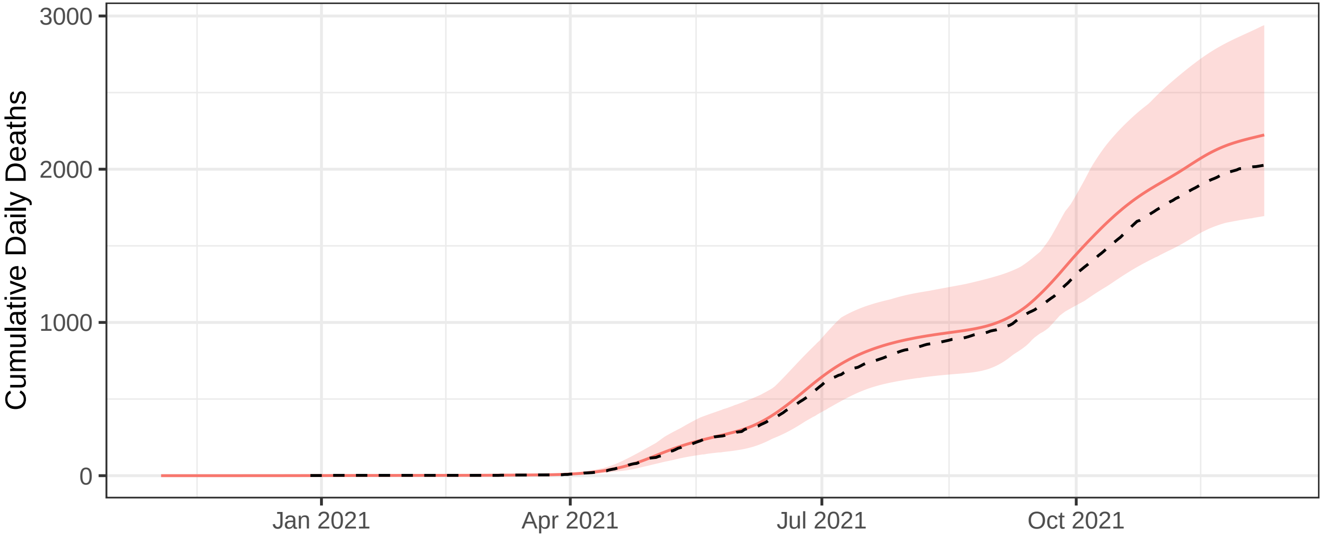

Daily deaths are included with reported COVID−19 deaths shown as points. The red line represents median model estimates of daily deaths, and the shaded region represents the 95% quantiles of the model estimated deaths. Plots of the cumulative estimated deaths are also shown, with the cumulative number of reported deaths represented by a dashed black line.

Mozambique

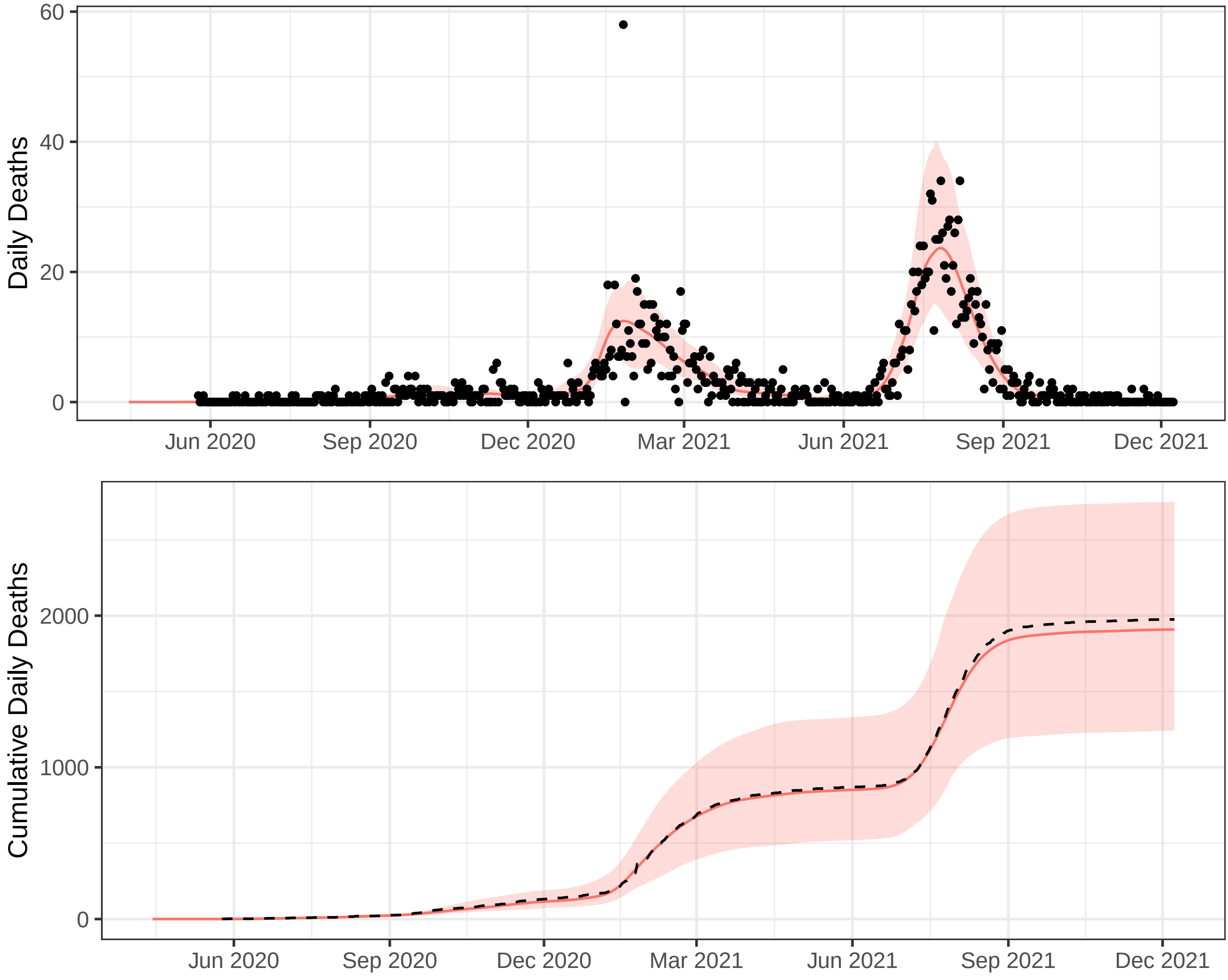

Daily deaths are included with reported COVID−19 deaths shown as points. The red line represents median model estimates of daily deaths, and the shaded region represents the 95% quantiles of the model estimated deaths. Plots of the cumulative estimated deaths are also shown, with the cumulative number of reported deaths represented by a dashed black line.

Mauritania

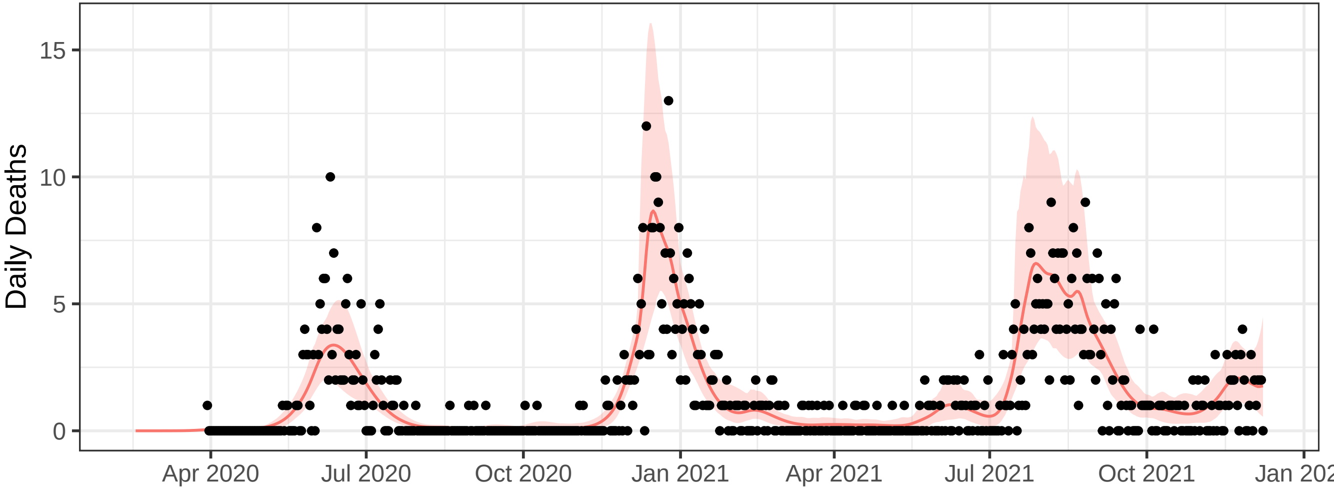

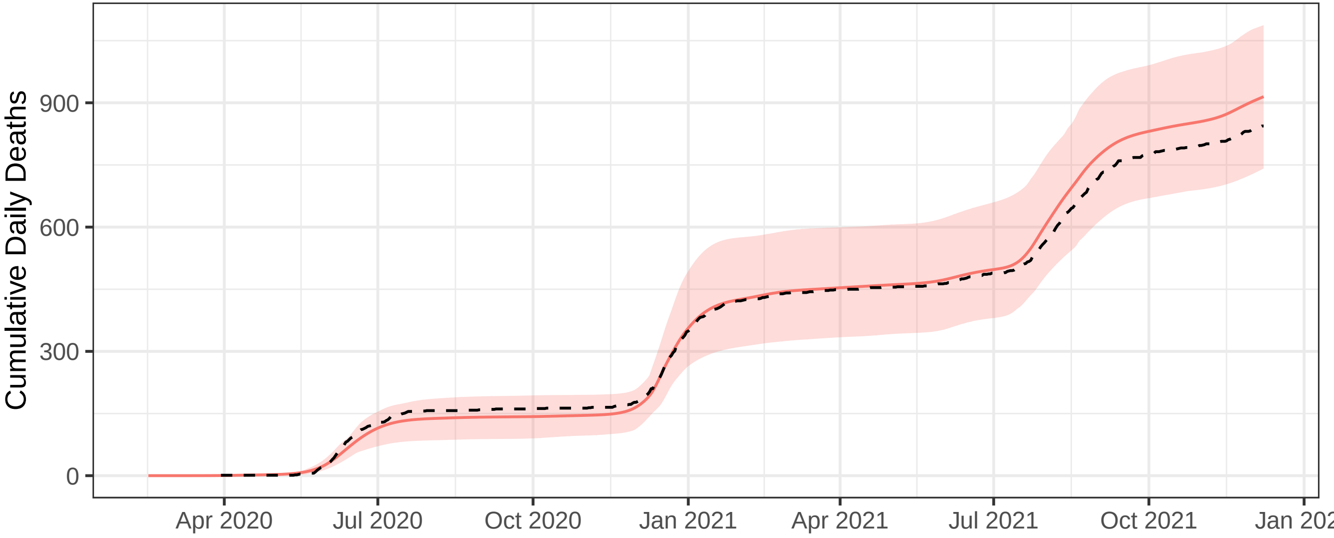

Daily deaths are included with reported COVID−19 deaths shown as points. The red line represents median model estimates of daily deaths, and the shaded region represents the 95% quantiles of the model estimated deaths. Plots of the cumulative estimated deaths are also shown, with the cumulative number of reported deaths represented by a dashed black line.

Mauritius

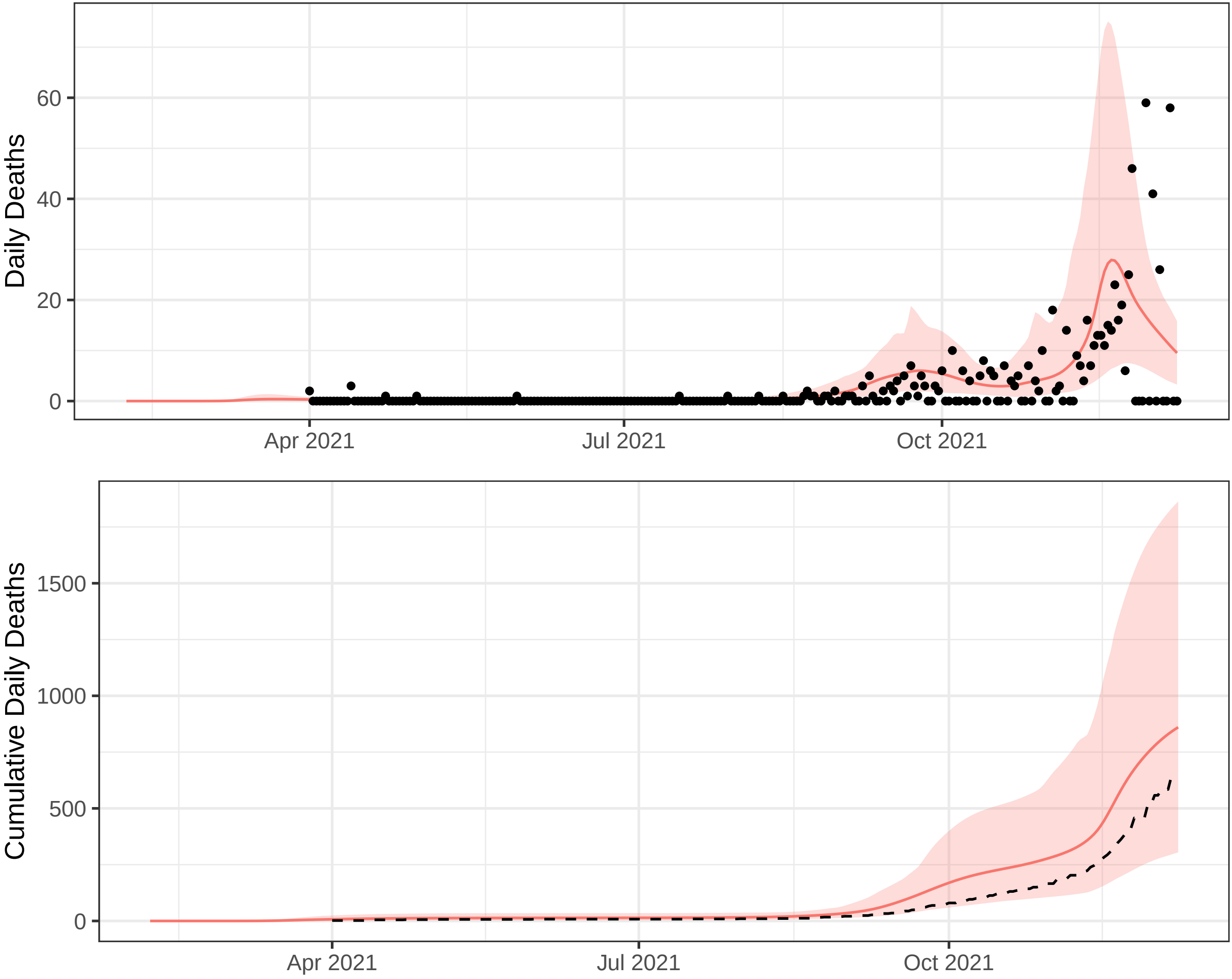

Daily deaths are included with reported COVID−19 deaths shown as points. The red line represents median model estimates of daily deaths, and the shaded region represents the 95% quantiles of the model estimated deaths. Plots of the cumulative estimated deaths are also shown, with the cumulative number of reported deaths represented by a dashed black line.

Malawi

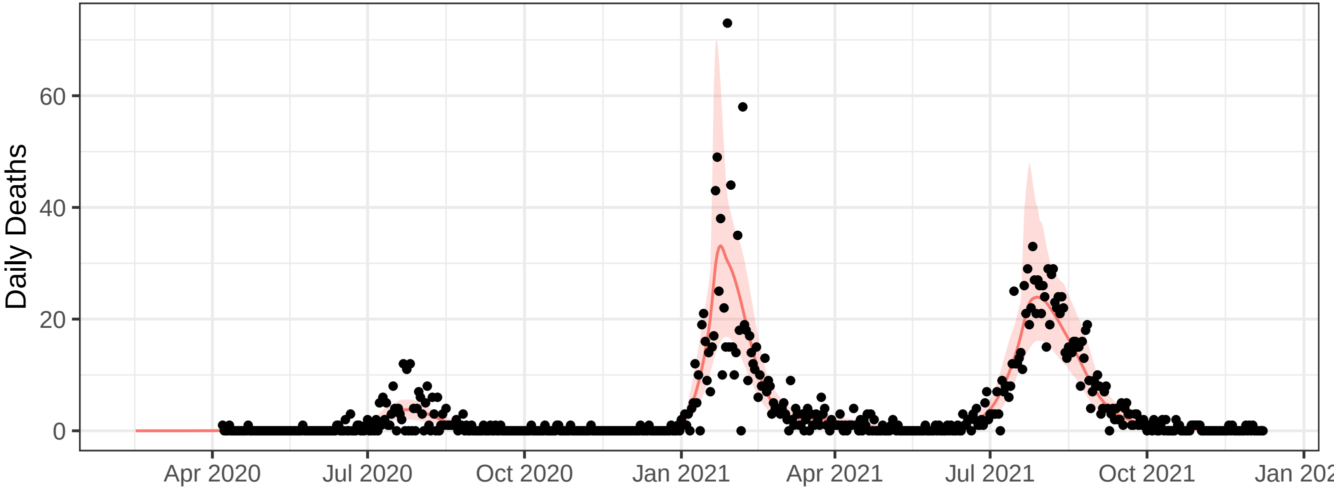

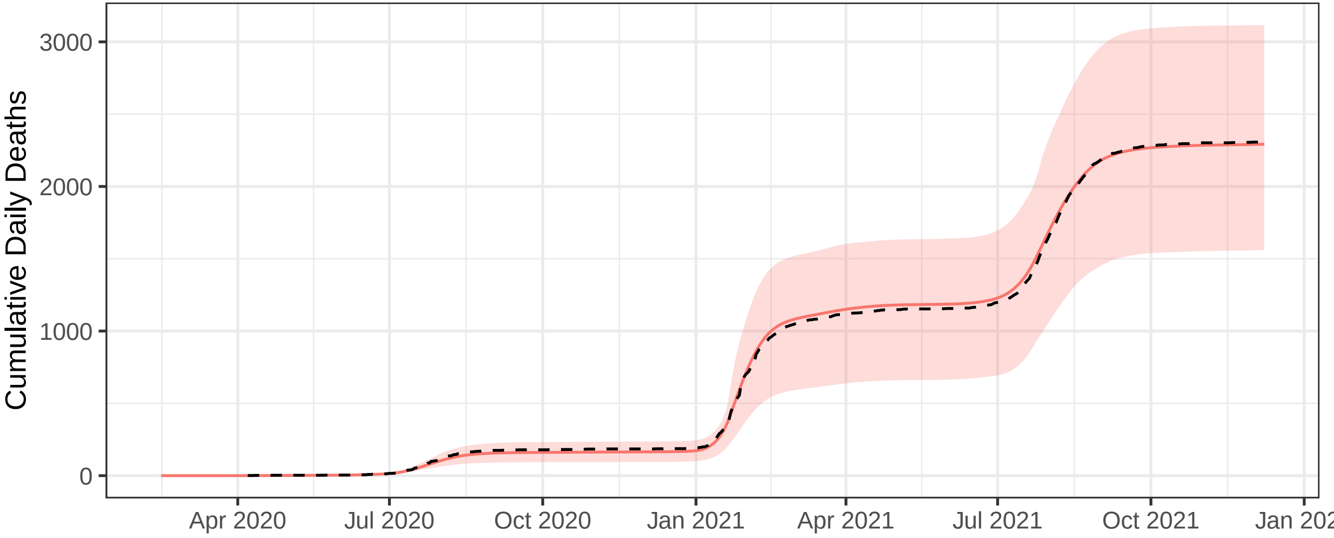

Daily deaths are included with reported COVID−19 deaths shown as points. The red line represents median model estimates of daily deaths, and the shaded region represents the 95% quantiles of the model estimated deaths. Plots of the cumulative estimated deaths are also shown, with the cumulative number of reported deaths represented by a dashed black line.

Malaysia

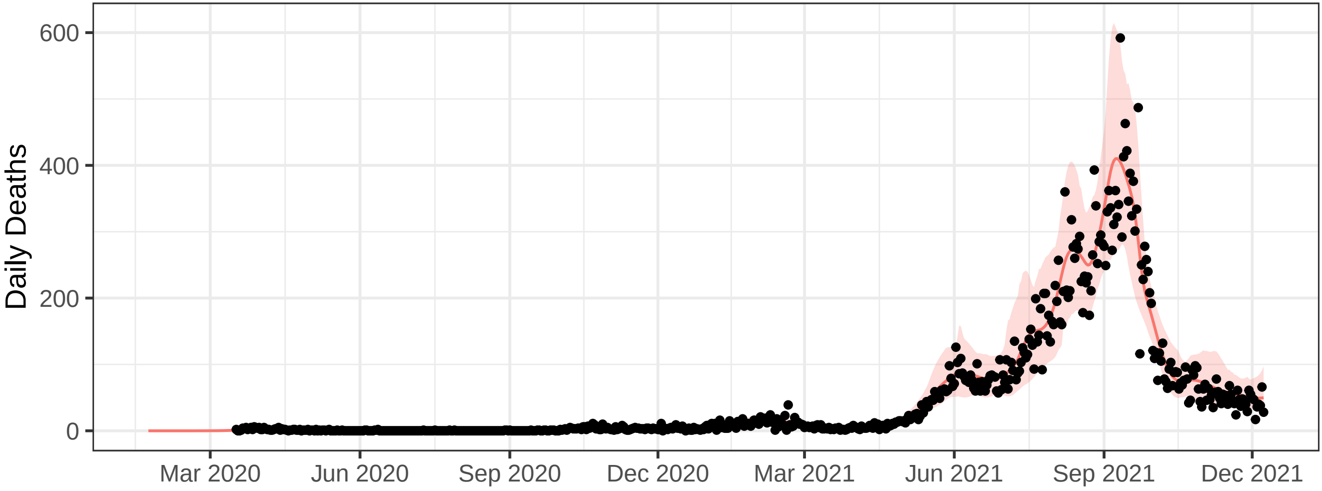

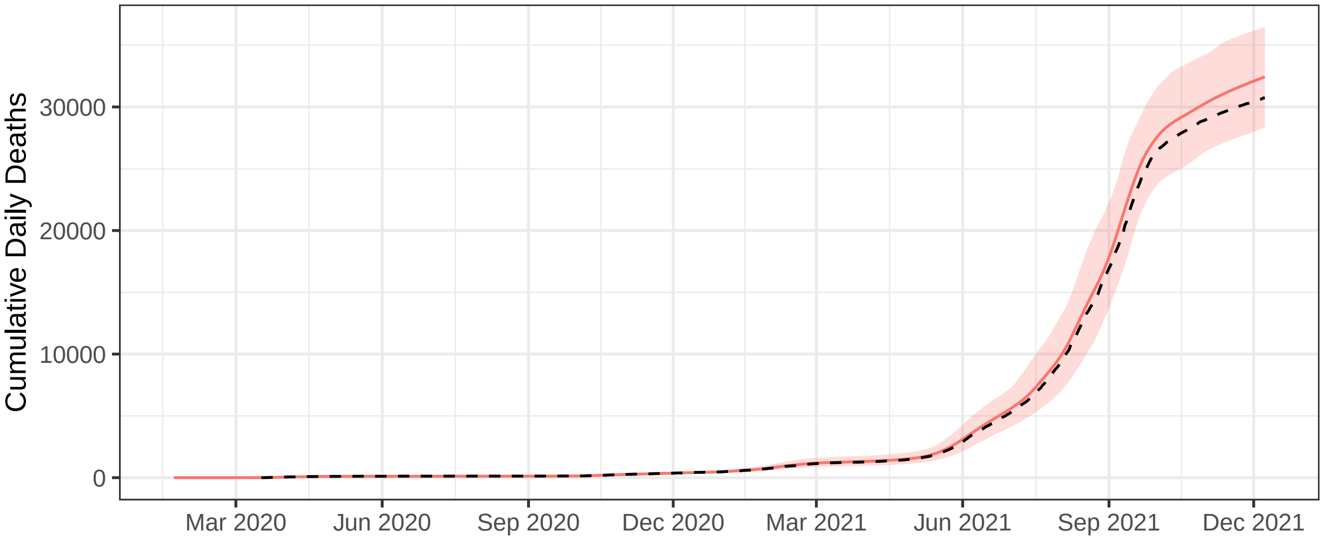

Daily deaths are included with reported COVID−19 deaths shown as points. The red line represents median model estimates of daily deaths, and the shaded region represents the 95% quantiles of the model estimated deaths. Plots of the cumulative estimated deaths are also shown, with the cumulative number of reported deaths represented by a dashed black line.

Namibia

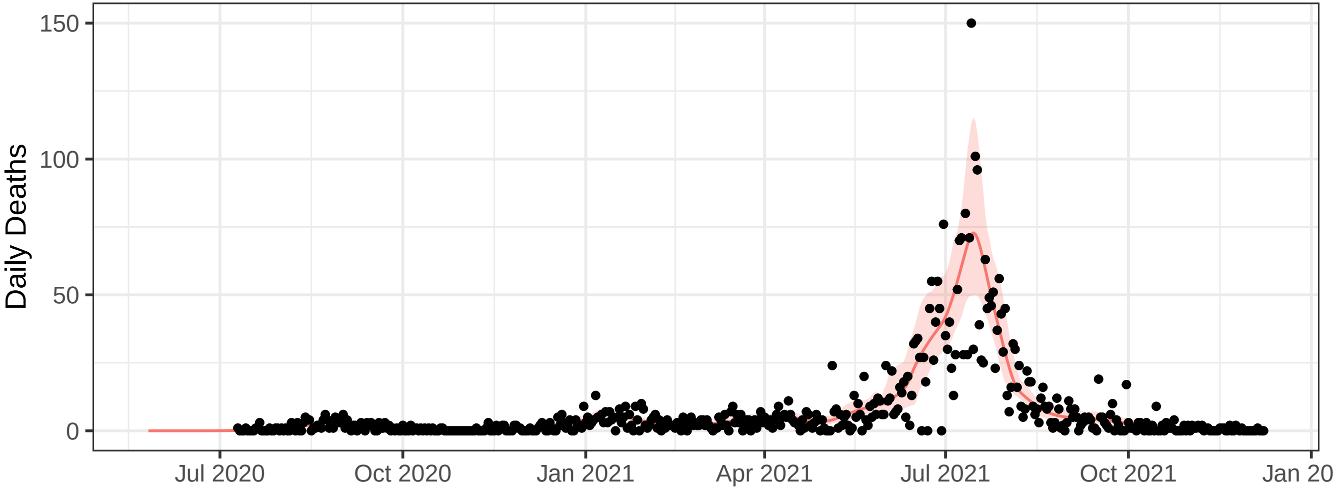

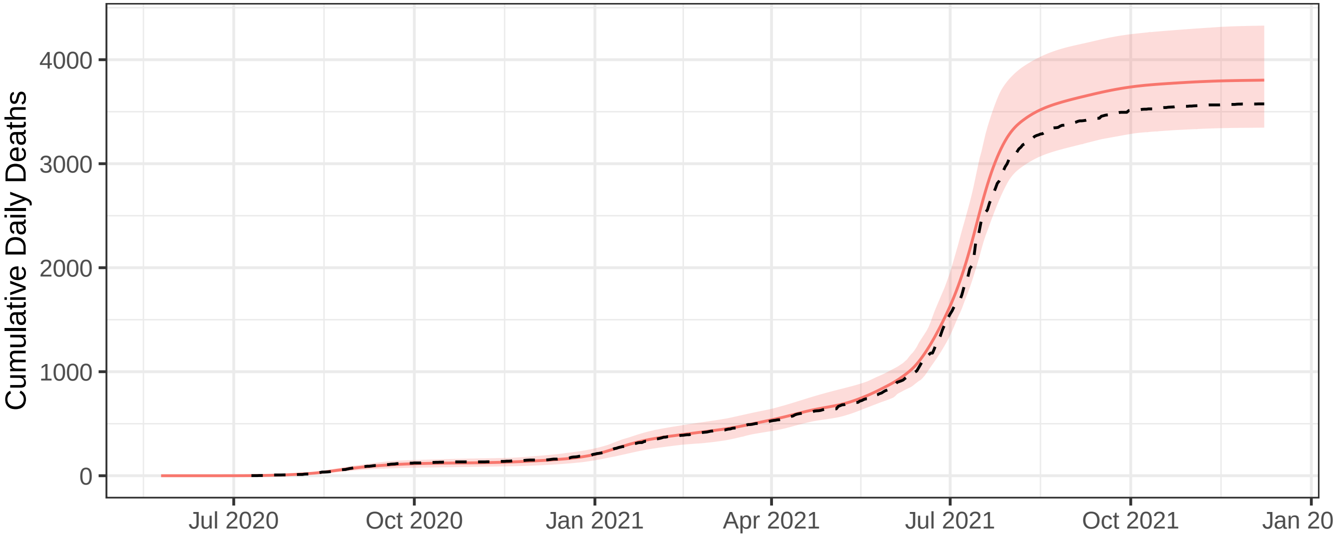

Daily deaths are included with reported COVID−19 deaths shown as points. The red line represents median model estimates of daily deaths, and the shaded region represents the 95% quantiles of the model estimated deaths. Plots of the cumulative estimated deaths are also shown, with the cumulative number of reported deaths represented by a dashed black line.

Niger

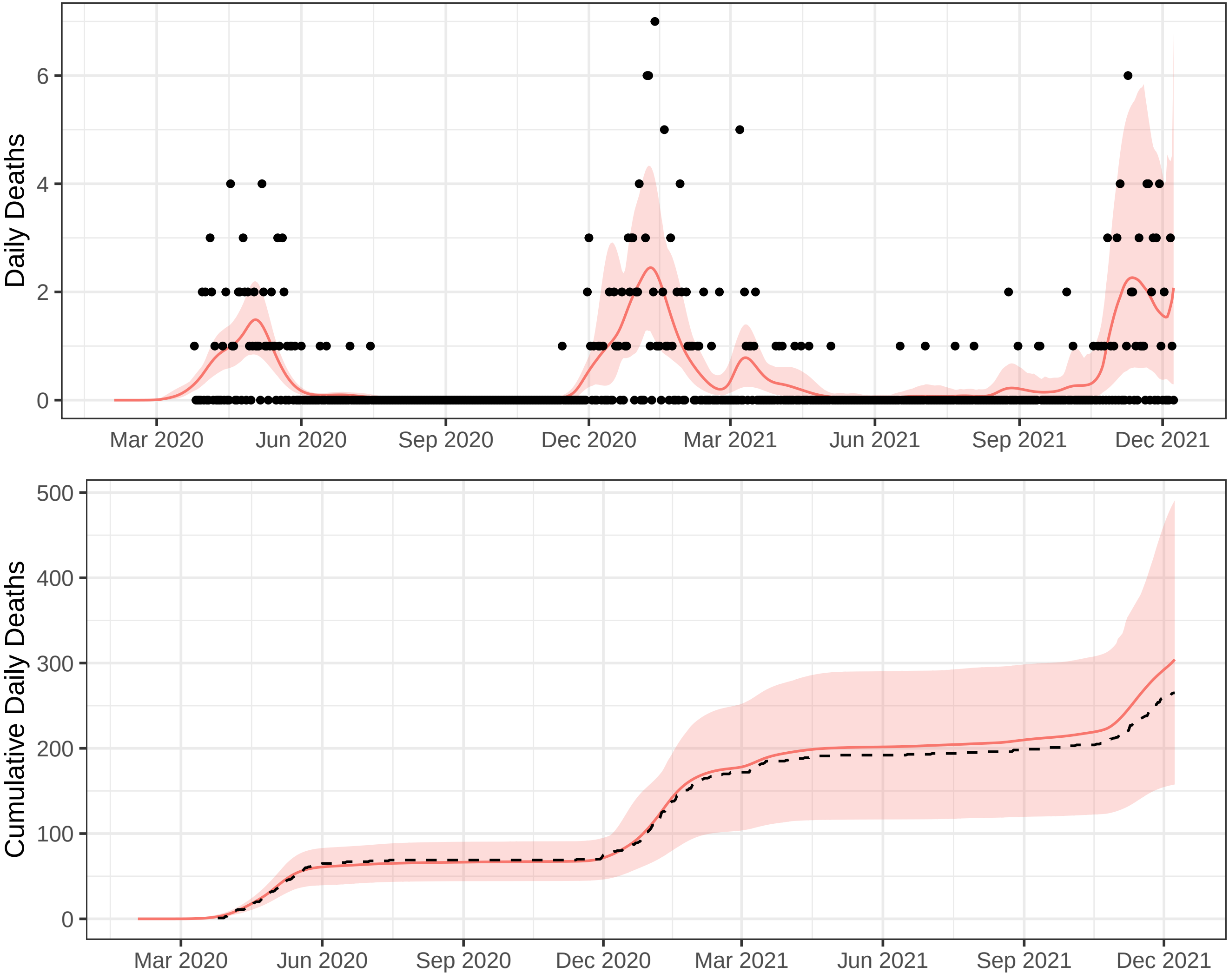

Daily deaths are included with reported COVID−19 deaths shown as points. The red line represents median model estimates of daily deaths, and the shaded region represents the 95% quantiles of the model estimated deaths. Plots of the cumulative estimated deaths are also shown, with the cumulative number of reported deaths represented by a dashed black line.

Nigeria

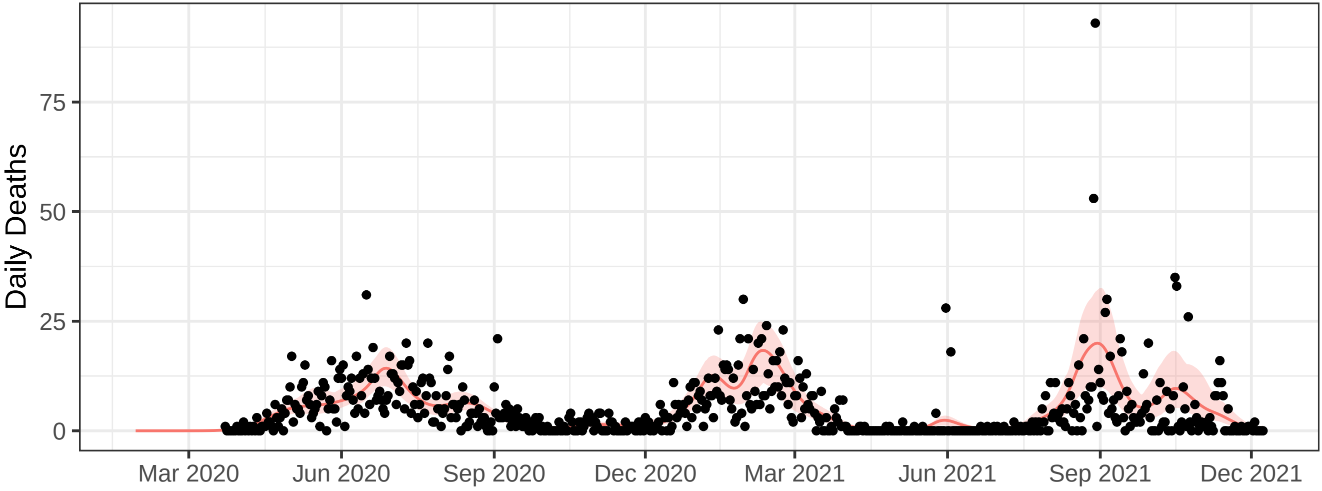

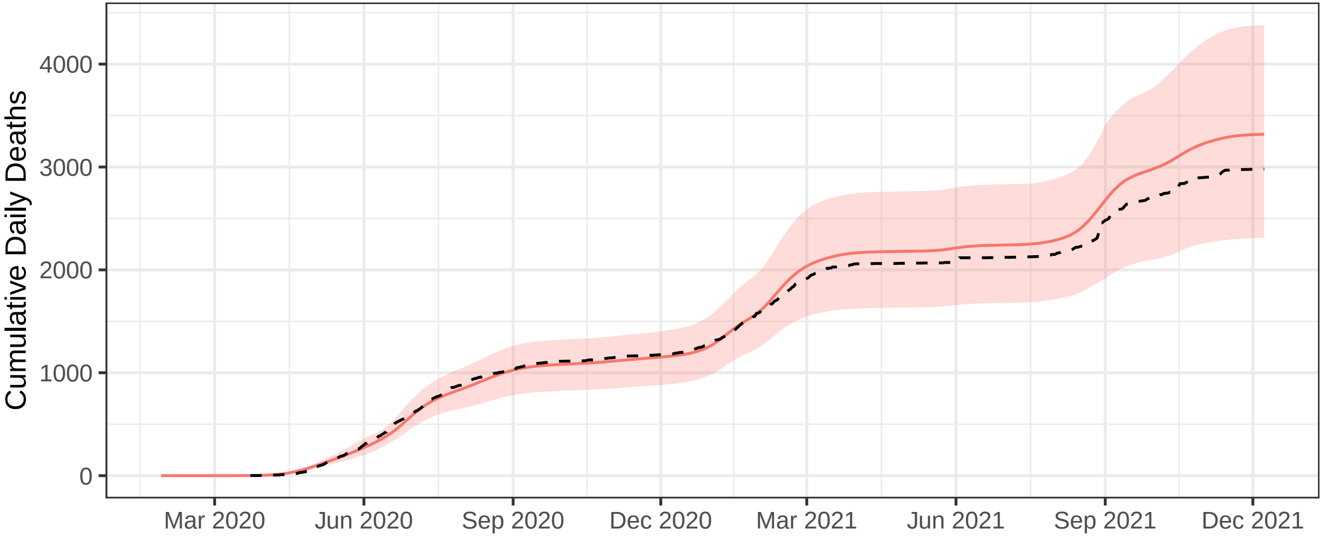

Daily deaths are included with reported COVID−19 deaths shown as points. The red line represents median model estimates of daily deaths, and the shaded region represents the 95% quantiles of the model estimated deaths. Plots of the cumulative estimated deaths are also shown, with the cumulative number of reported deaths represented by a dashed black line.

Nicaragua

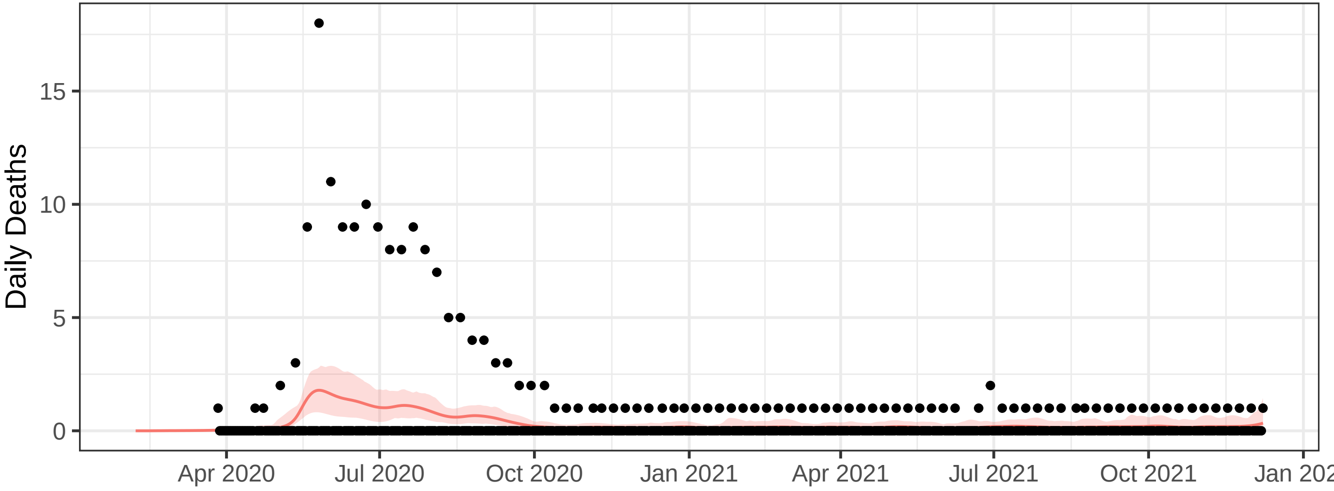

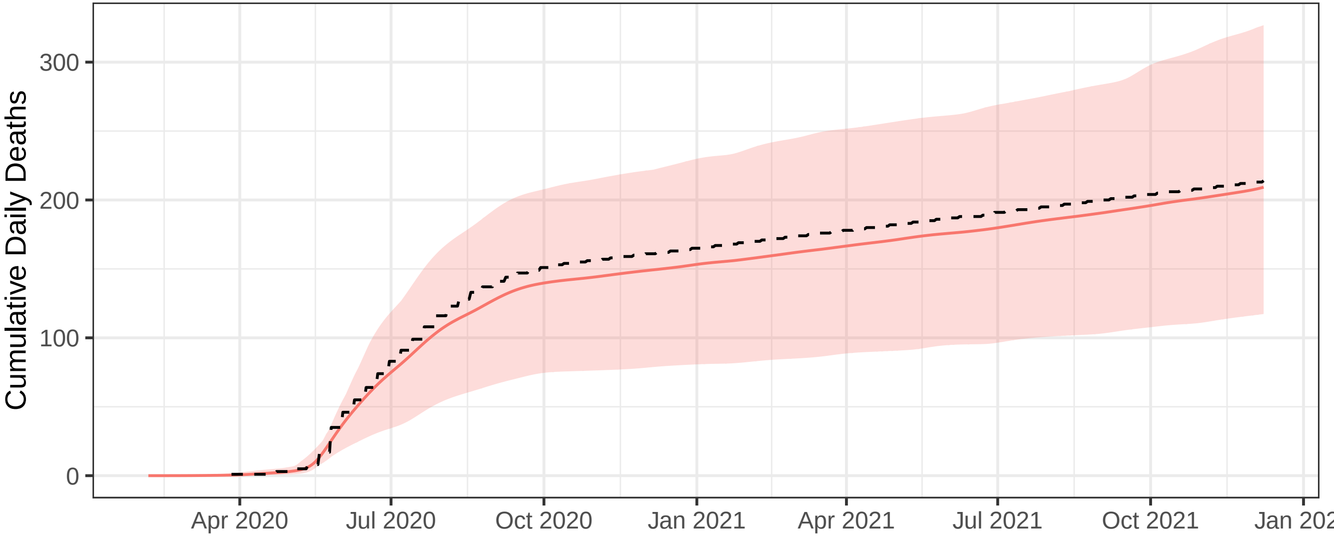

Daily deaths are included with reported COVID−19 deaths shown as points. The red line represents median model estimates of daily deaths, and the shaded region represents the 95% quantiles of the model estimated deaths. Plots of the cumulative estimated deaths are also shown, with the cumulative number of reported deaths represented by a dashed black line.

Netherlands

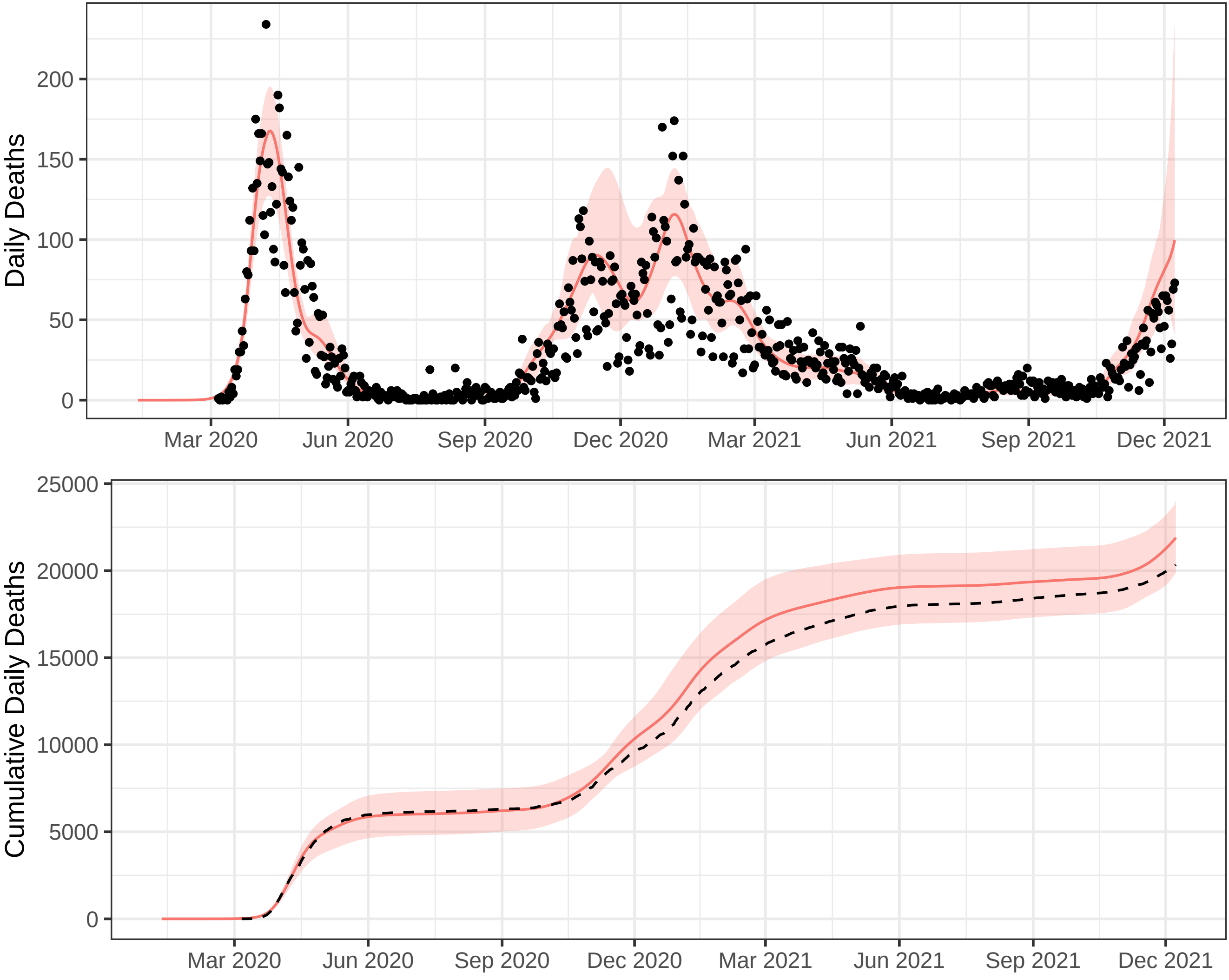

Daily deaths are included with reported COVID−19 deaths shown as points. The red line represents median model estimates of daily deaths, and the shaded region represents the 95% quantiles of the model estimated deaths. Plots of the cumulative estimated deaths are also shown, with the cumulative number of reported deaths represented by a dashed black line.

Norway

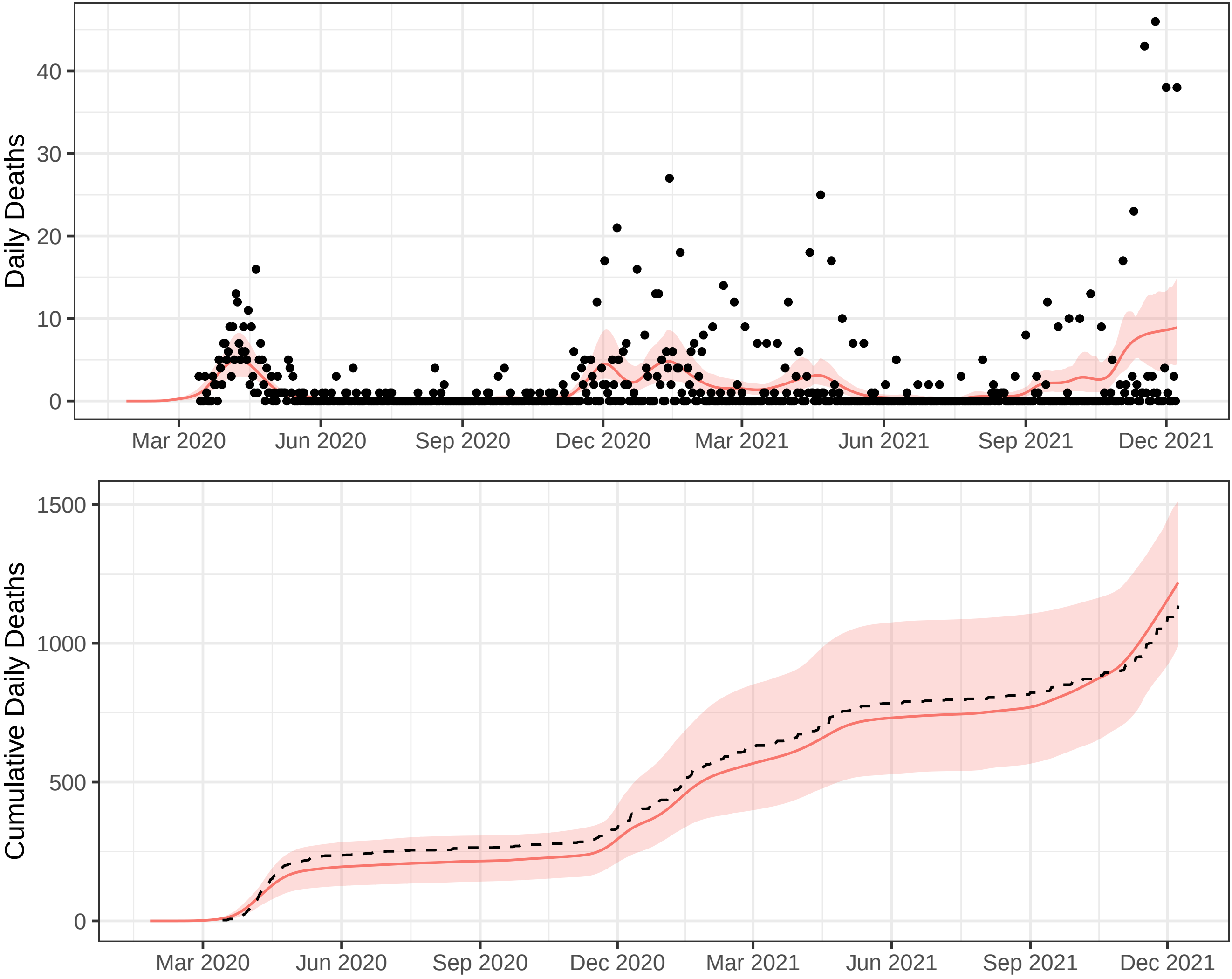

Daily deaths are included with reported COVID−19 deaths shown as points. The red line represents median model estimates of daily deaths, and the shaded region represents the 95% quantiles of the model estimated deaths. Plots of the cumulative estimated deaths are also shown, with the cumulative number of reported deaths represented by a dashed black line.

Nepal

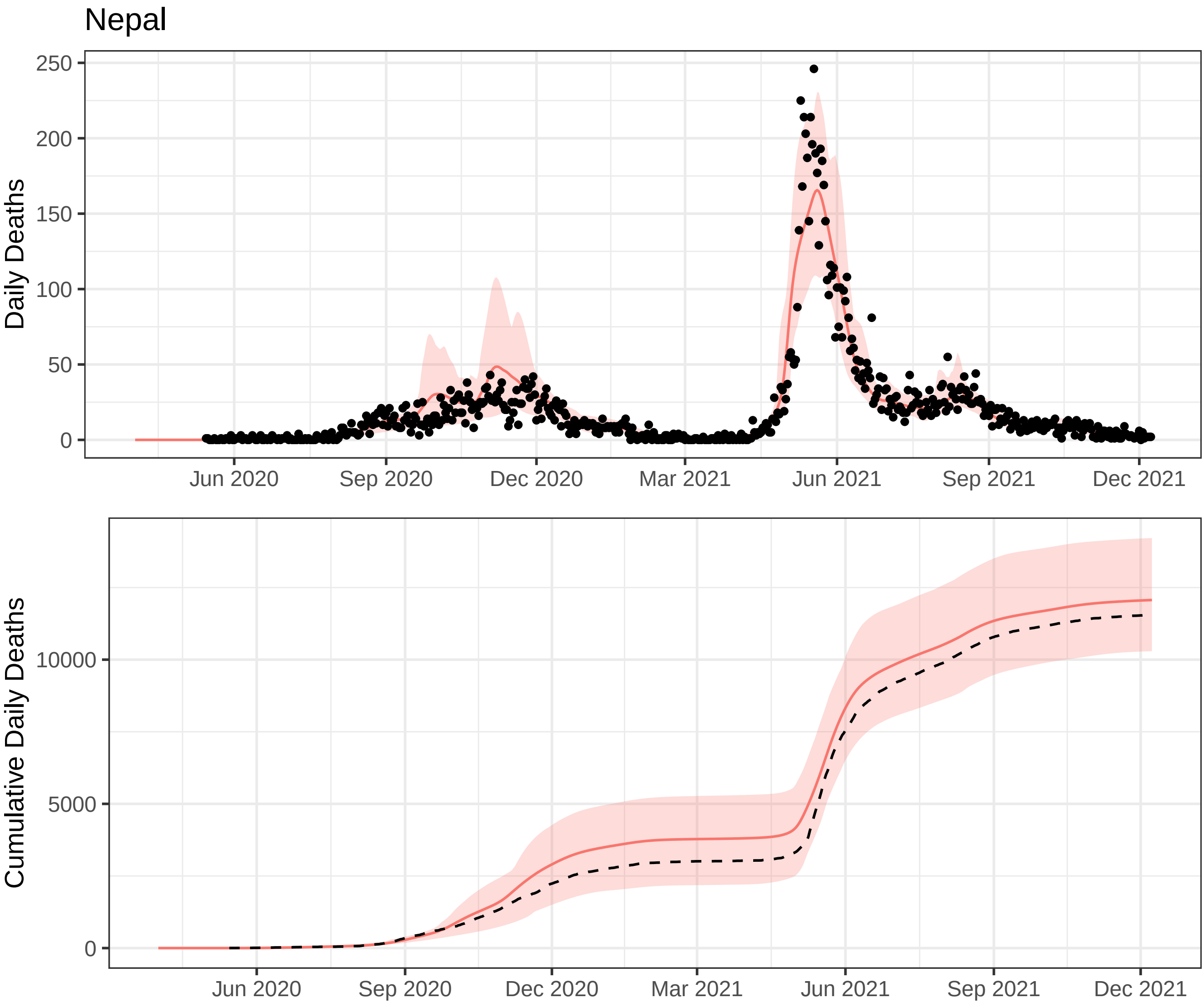

Daily deaths are included with reported COVID−19 deaths shown as points. The red line represents median model estimates of daily deaths, and the shaded region represents the 95% quantiles of the model estimated deaths. Plots of the cumulative estimated deaths are also shown, with the cumulative number of reported deaths represented by a dashed black line.

New Zealand

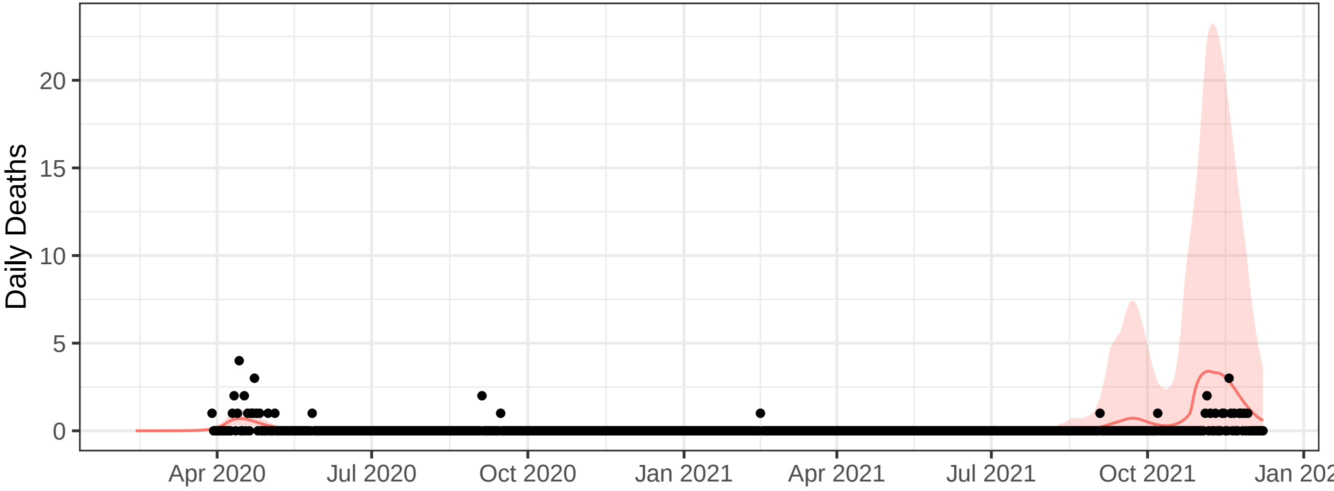

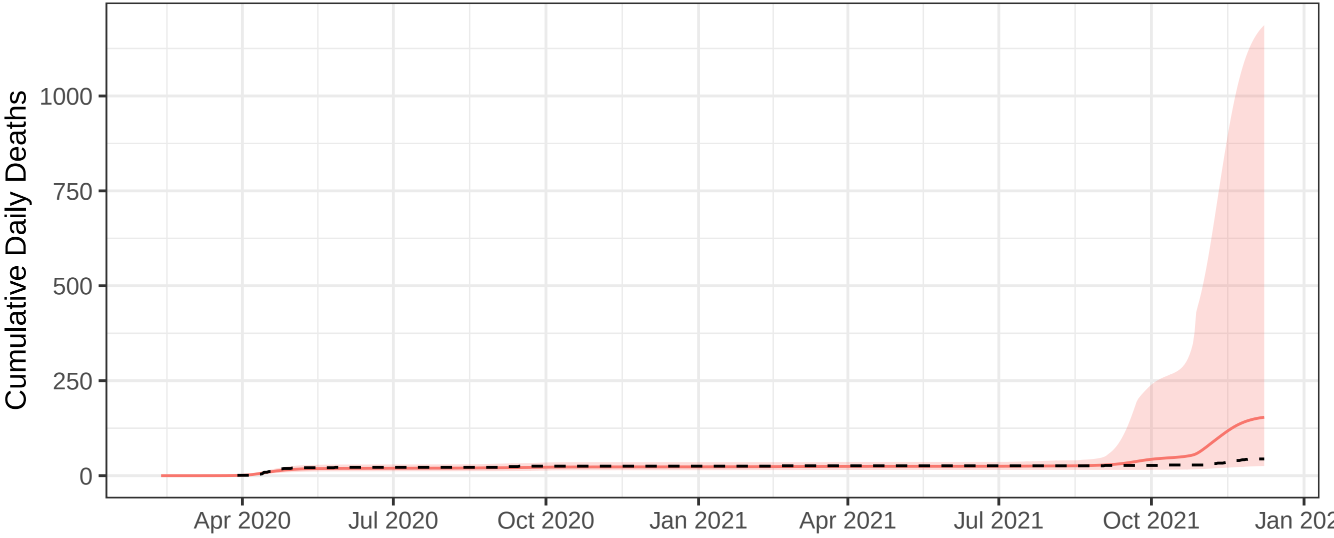

Daily deaths are included with reported COVID−19 deaths shown as points. The red line represents median model estimates of daily deaths, and the shaded region represents the 95% quantiles of the model estimated deaths. Plots of the cumulative estimated deaths are also shown, with the cumulative number of reported deaths represented by a dashed black line.

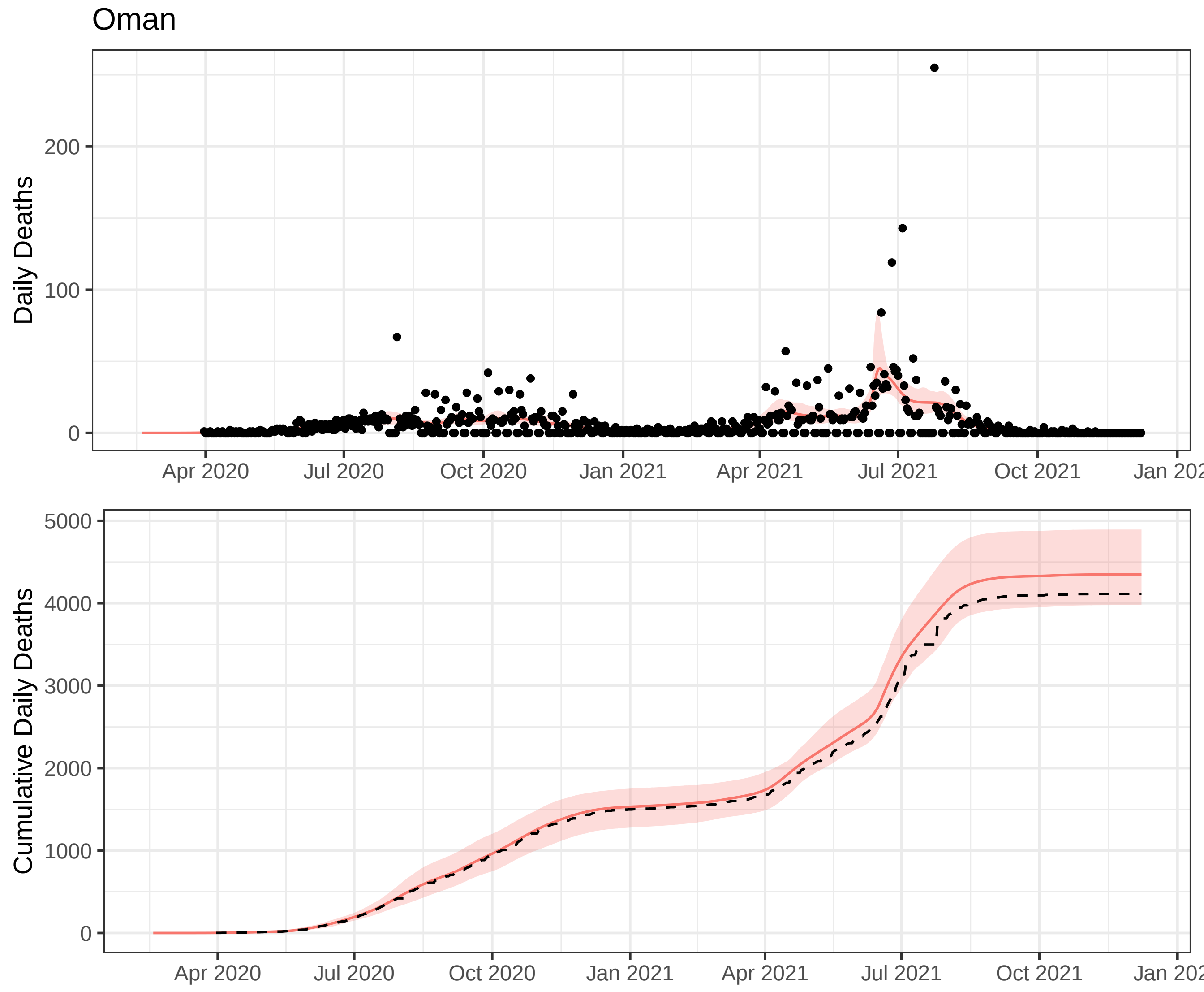

Daily deaths are included with reported COVID−19 deaths shown as points. The red line represents median model estimates of daily deaths, and the shaded region represents the 95% quantiles of the model estimated deaths. Plots of the cumulative estimated deaths are also shown, with the cumulative number of reported deaths represented by a dashed black line.

Pakistan

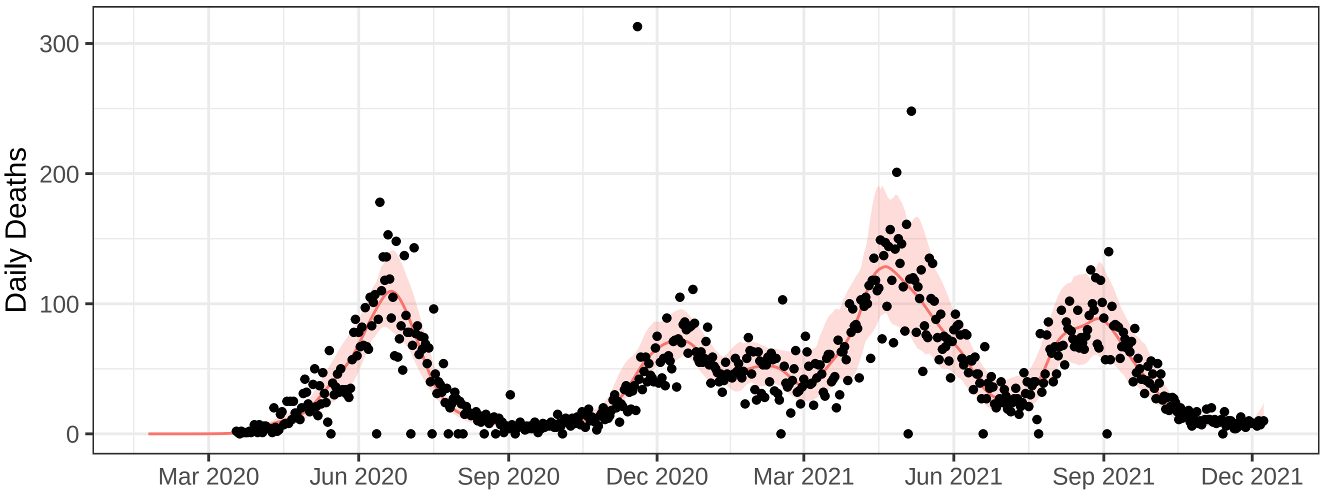

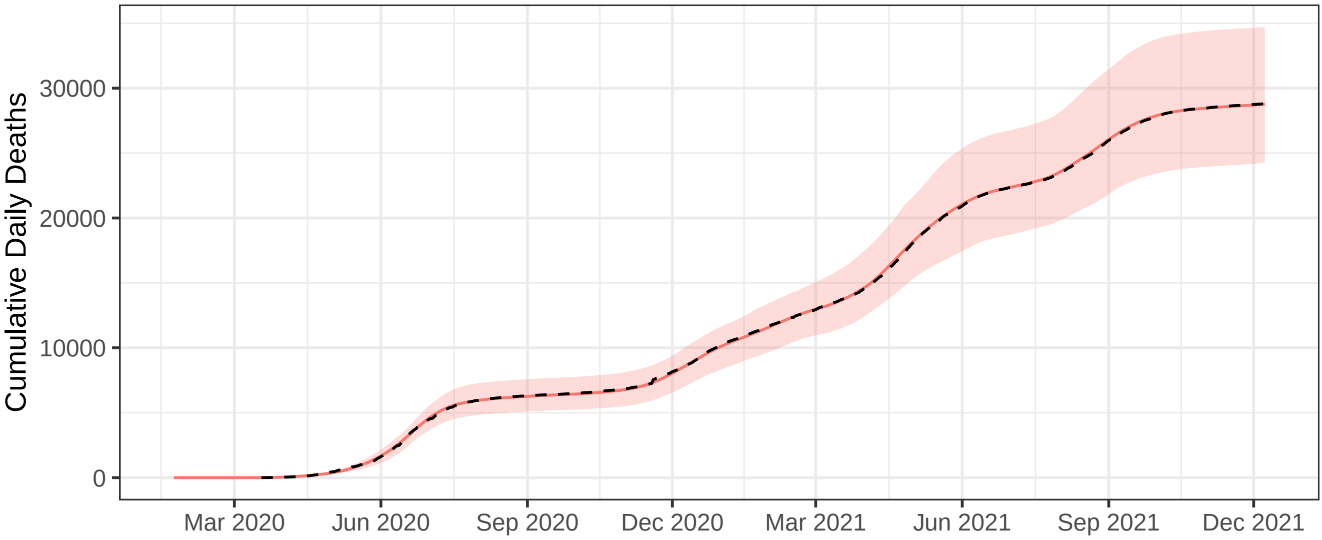

Daily deaths are included with reported COVID−19 deaths shown as points. The red line represents median model estimates of daily deaths, and the shaded region represents the 95% quantiles of the model estimated deaths. Plots of the cumulative estimated deaths are also shown, with the cumulative number of reported deaths represented by a dashed black line.

Panama

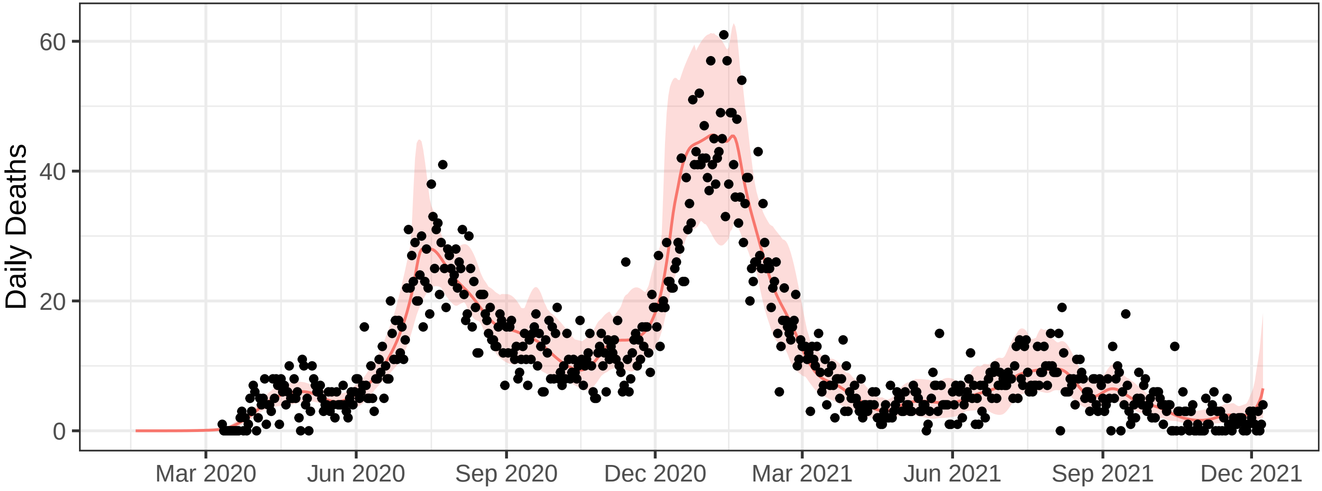

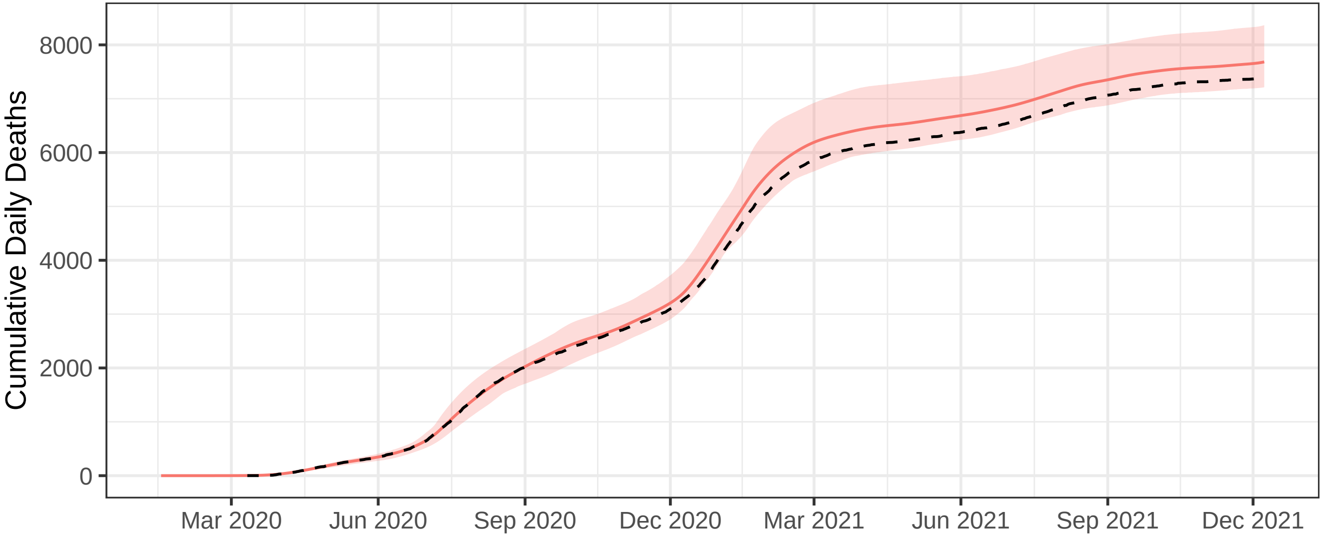

Daily deaths are included with reported COVID−19 deaths shown as points. The red line represents median model estimates of daily deaths, and the shaded region represents the 95% quantiles of the model estimated deaths. Plots of the cumulative estimated deaths are also shown, with the cumulative number of reported deaths represented by a dashed black line.

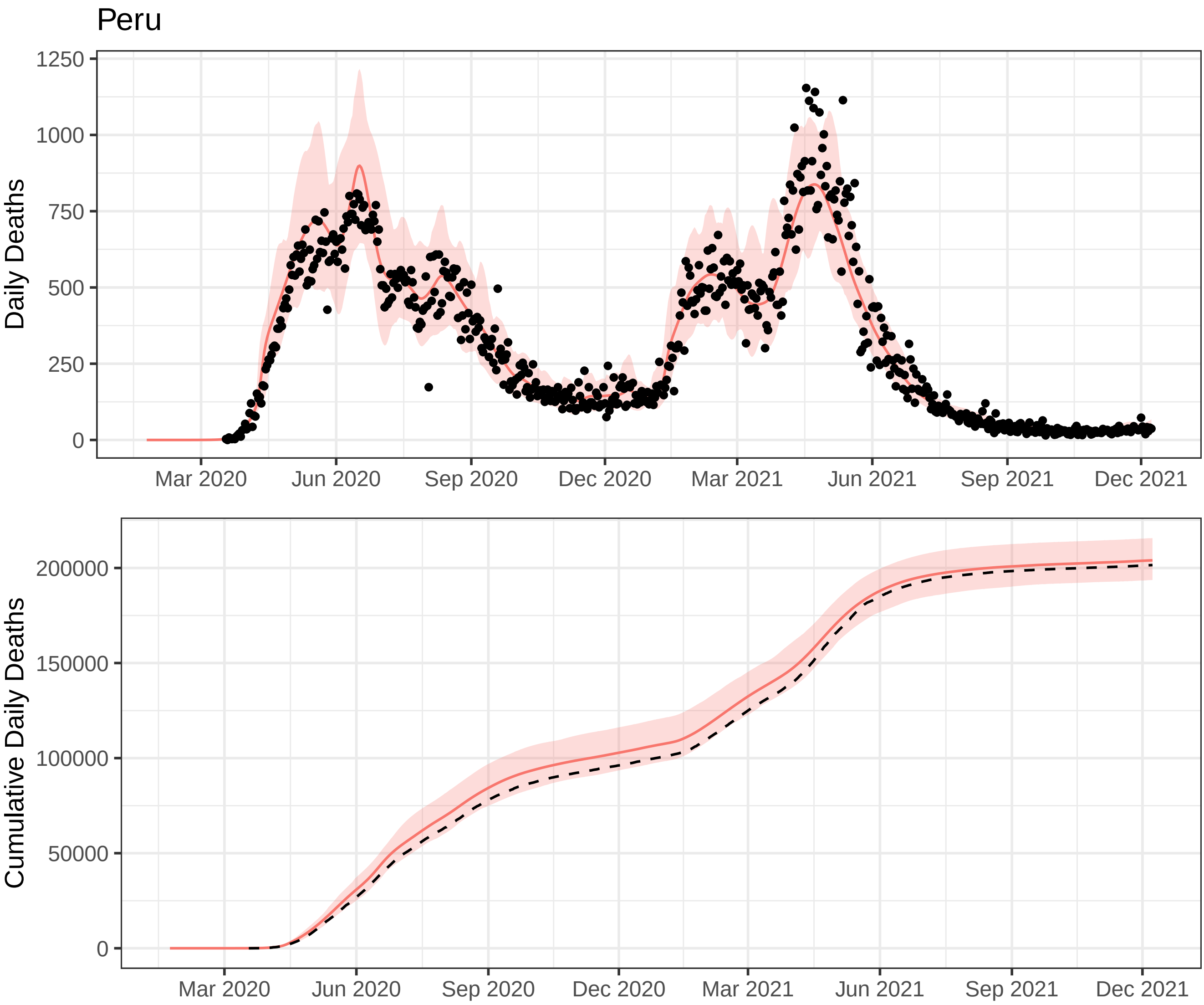

Daily deaths are included with reported COVID−19 deaths shown as points. The red line represents median model estimates of daily deaths, and the shaded region represents the 95% quantiles of the model estimated deaths. Plots of the cumulative estimated deaths are also shown, with the cumulative number of reported deaths represented by a dashed black line.

Philippines

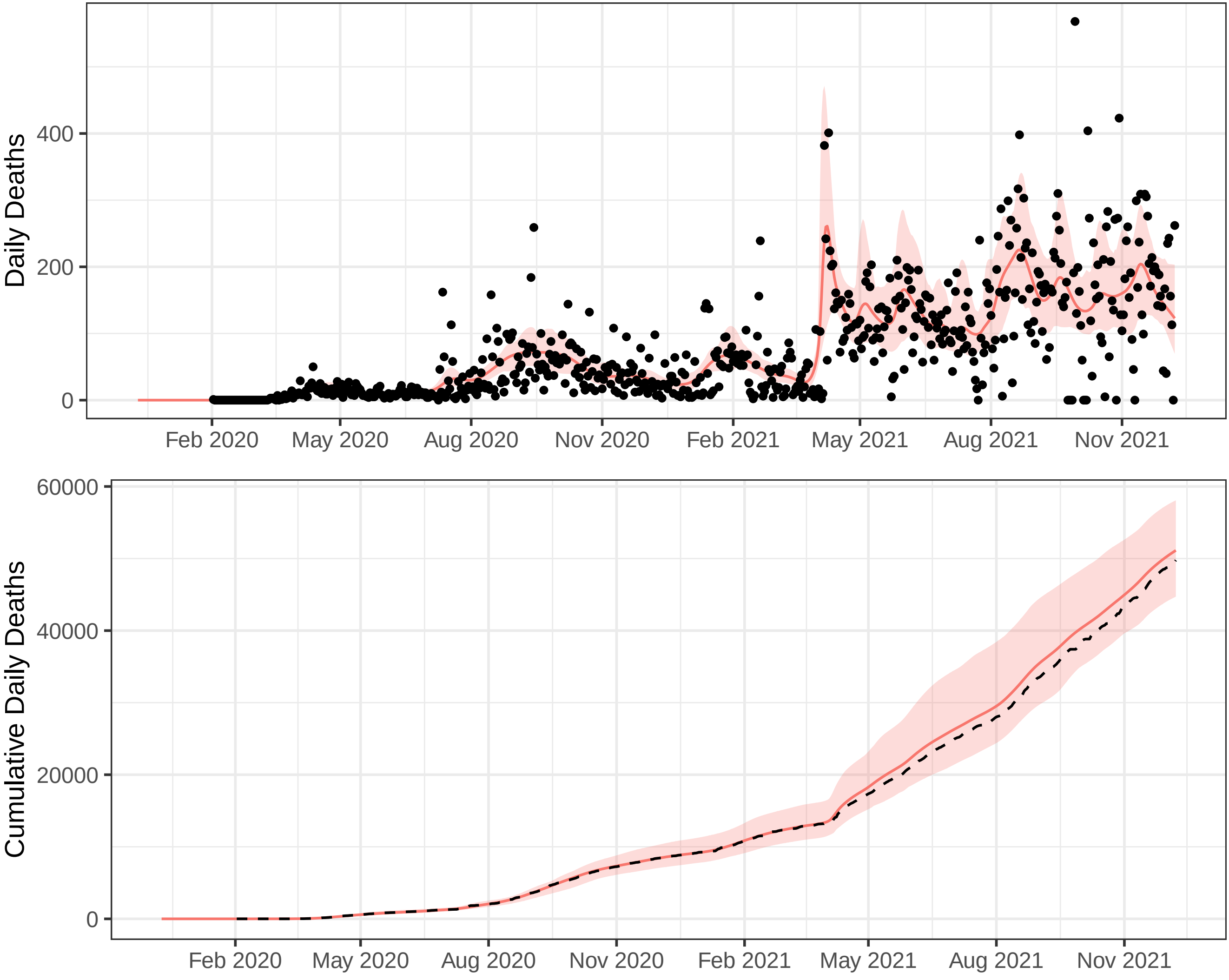

Daily deaths are included with reported COVID−19 deaths shown as points. The red line represents median model estimates of daily deaths, and the shaded region represents the 95% quantiles of the model estimated deaths. Plots of the cumulative estimated deaths are also shown, with the cumulative number of reported deaths represented by a dashed black line.

Papua New Guinea

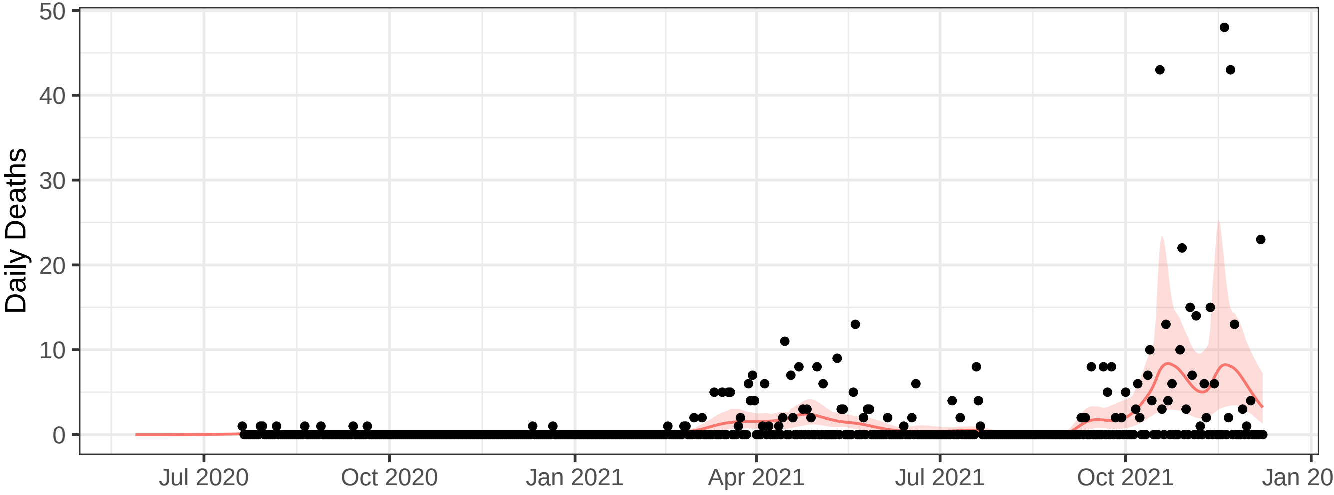

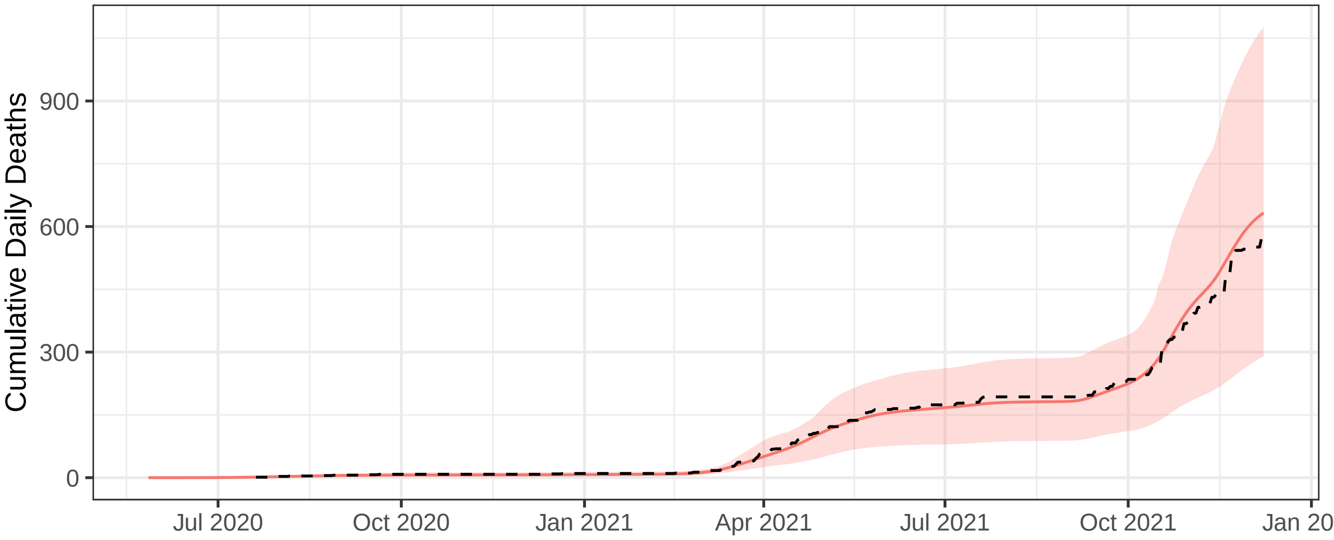

Daily deaths are included with reported COVID−19 deaths shown as points. The red line represents median model estimates of daily deaths, and the shaded region represents the 95% quantiles of the model estimated deaths. Plots of the cumulative estimated deaths are also shown, with the cumulative number of reported deaths represented by a dashed black line.

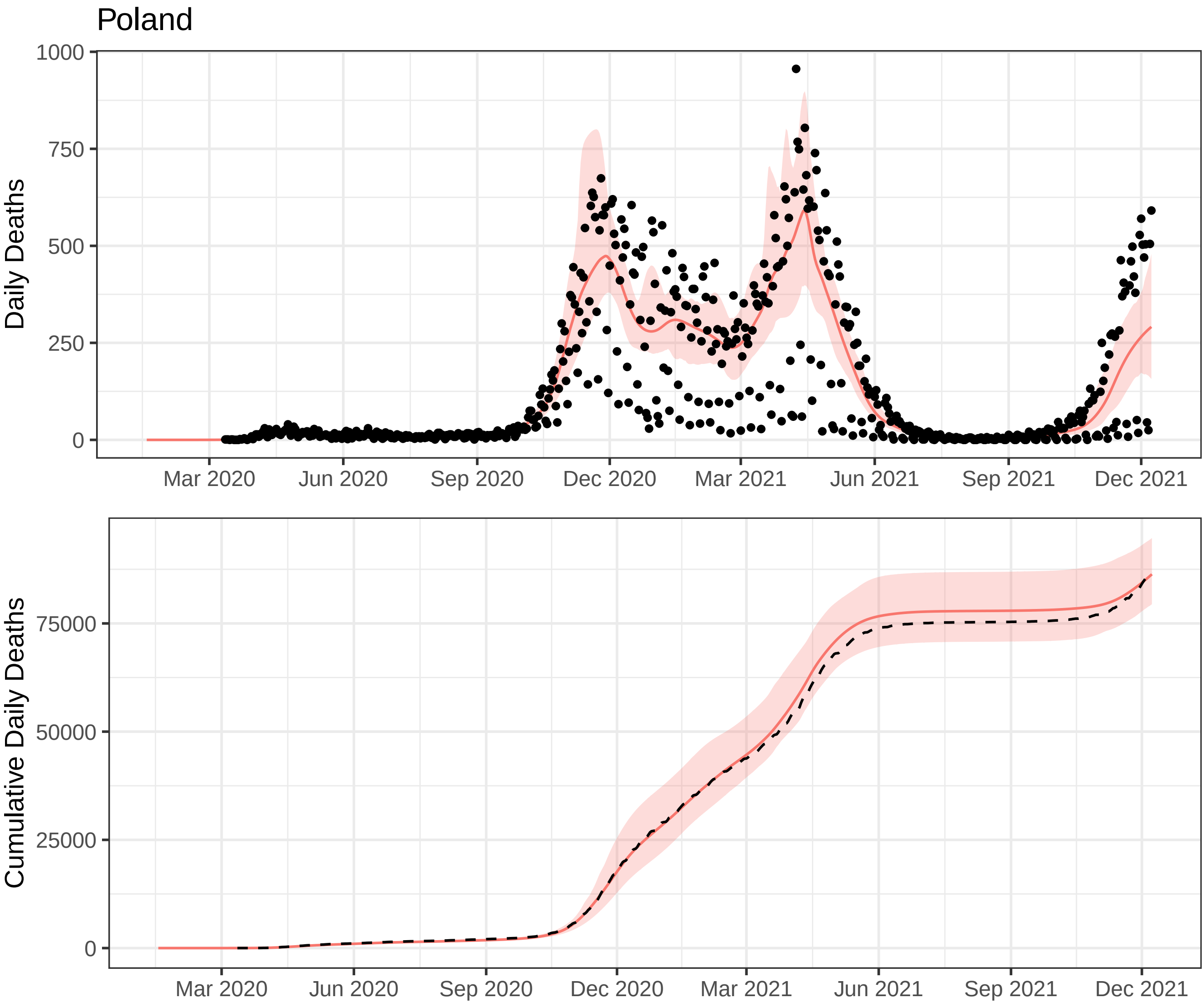

Daily deaths are included with reported COVID−19 deaths shown as points. The red line represents median model estimates of daily deaths, and the shaded region represents the 95% quantiles of the model estimated deaths. Plots of the cumulative estimated deaths are also shown, with the cumulative number of reported deaths represented by a dashed black line.

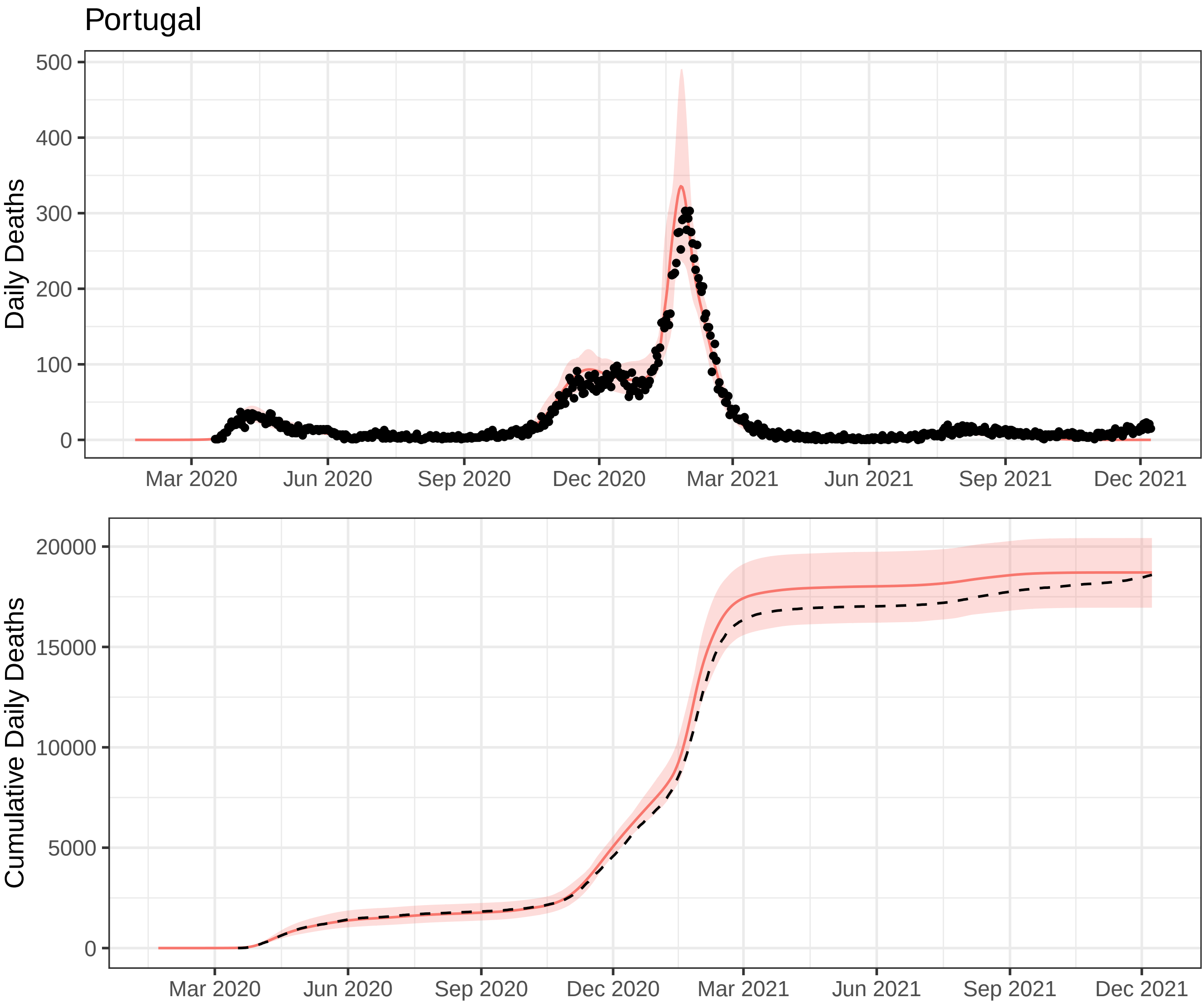

Daily deaths are included with reported COVID−19 deaths shown as points. The red line represents median model estimates of daily deaths, and the shaded region represents the 95% quantiles of the model estimated deaths. Plots of the cumulative estimated deaths are also shown, with the cumulative number of reported deaths represented by a dashed black line.

Paraguay

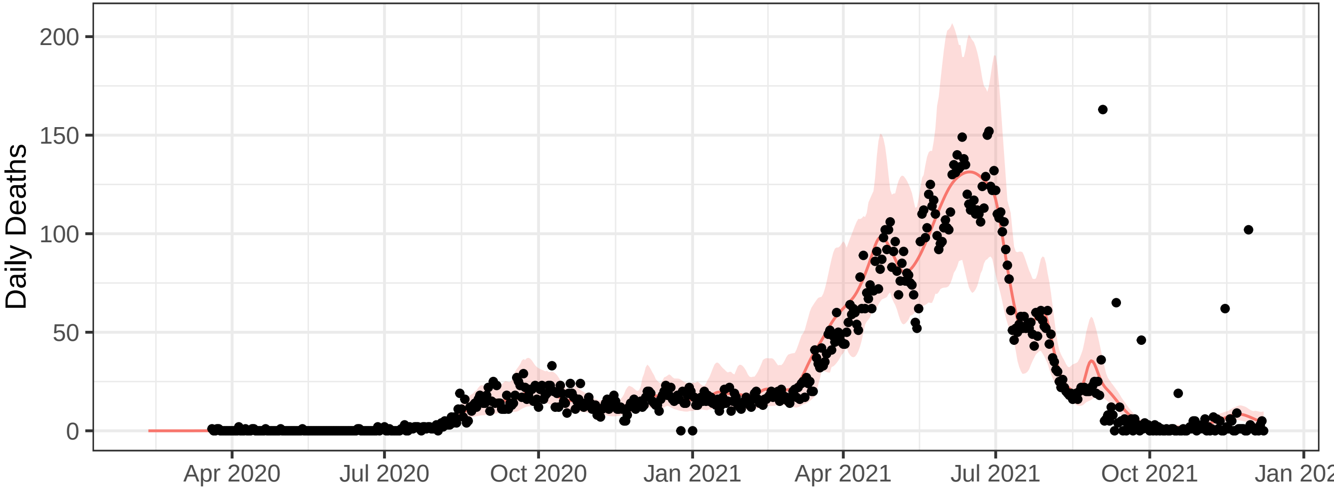

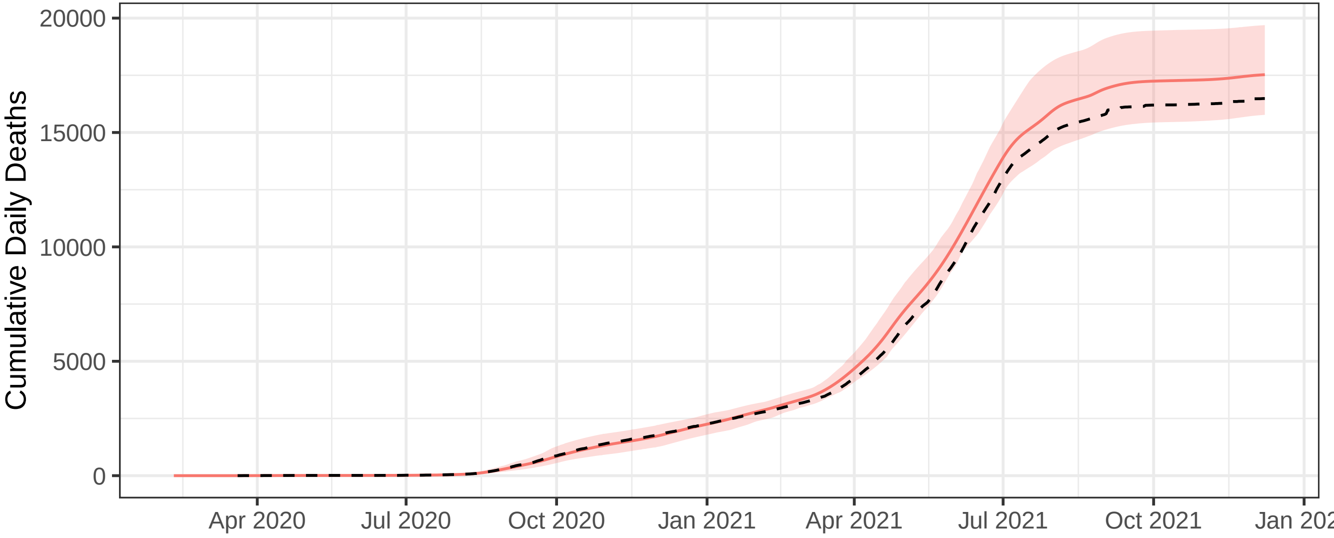

Daily deaths are included with reported COVID−19 deaths shown as points. The red line represents median model estimates of daily deaths, and the shaded region represents the 95% quantiles of the model estimated deaths. Plots of the cumulative estimated deaths are also shown, with the cumulative number of reported deaths represented by a dashed black line.

State of Palestine

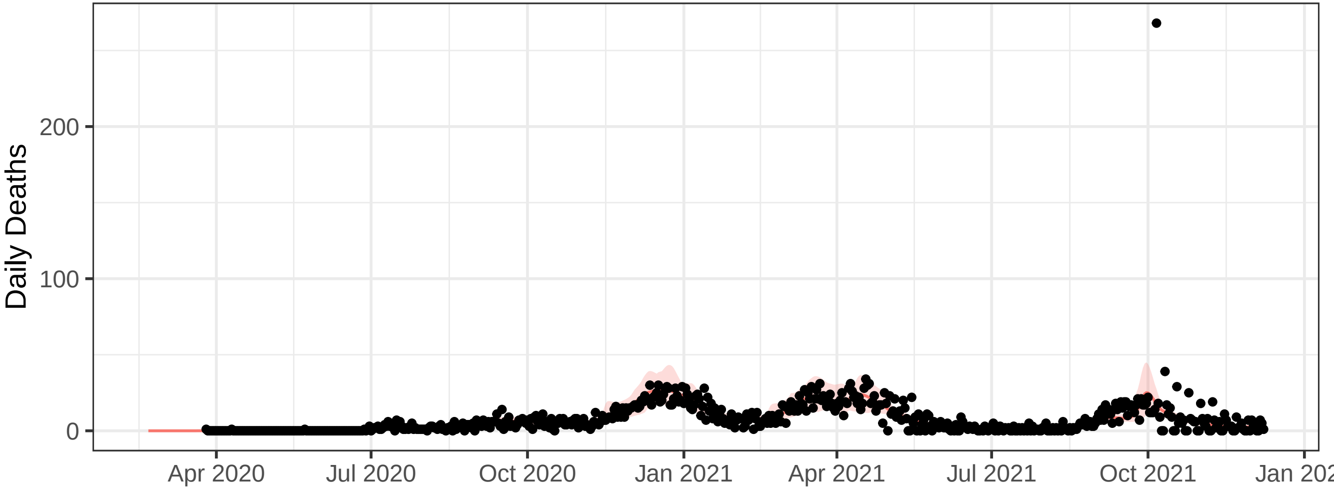

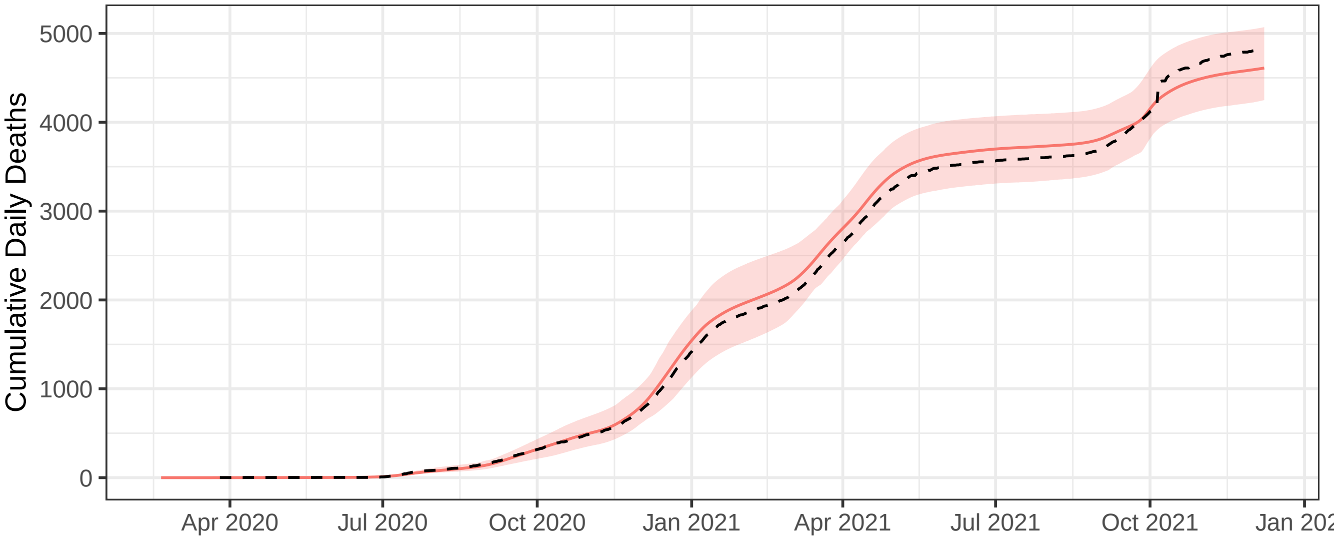

Daily deaths are included with reported COVID−19 deaths shown as points. The red line represents median model estimates of daily deaths, and the shaded region represents the 95% quantiles of the model estimated deaths. Plots of the cumulative estimated deaths are also shown, with the cumulative number of reported deaths represented by a dashed black line.

Qatar

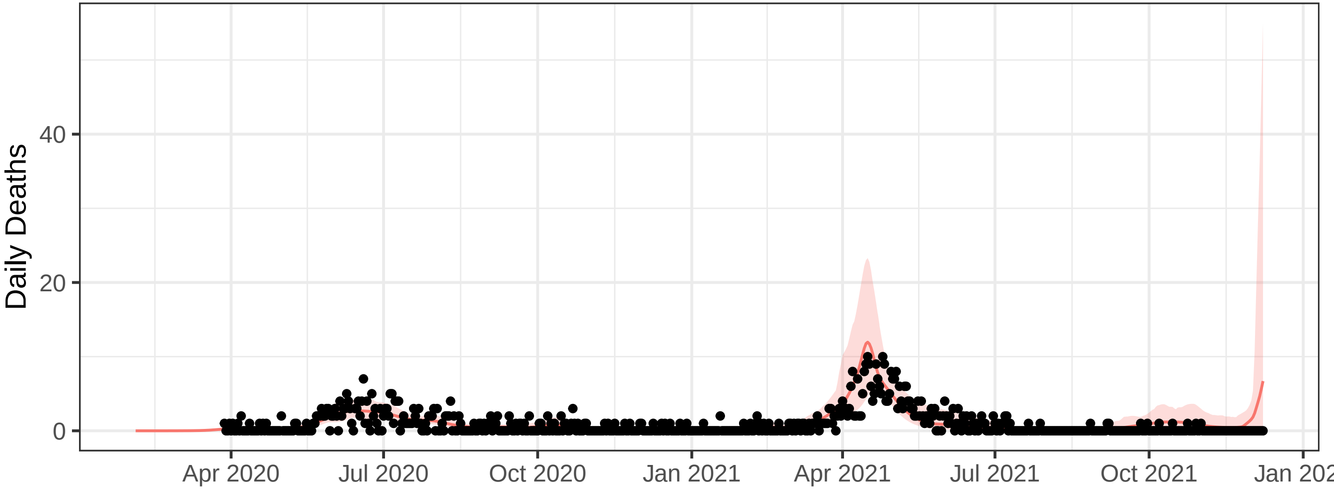

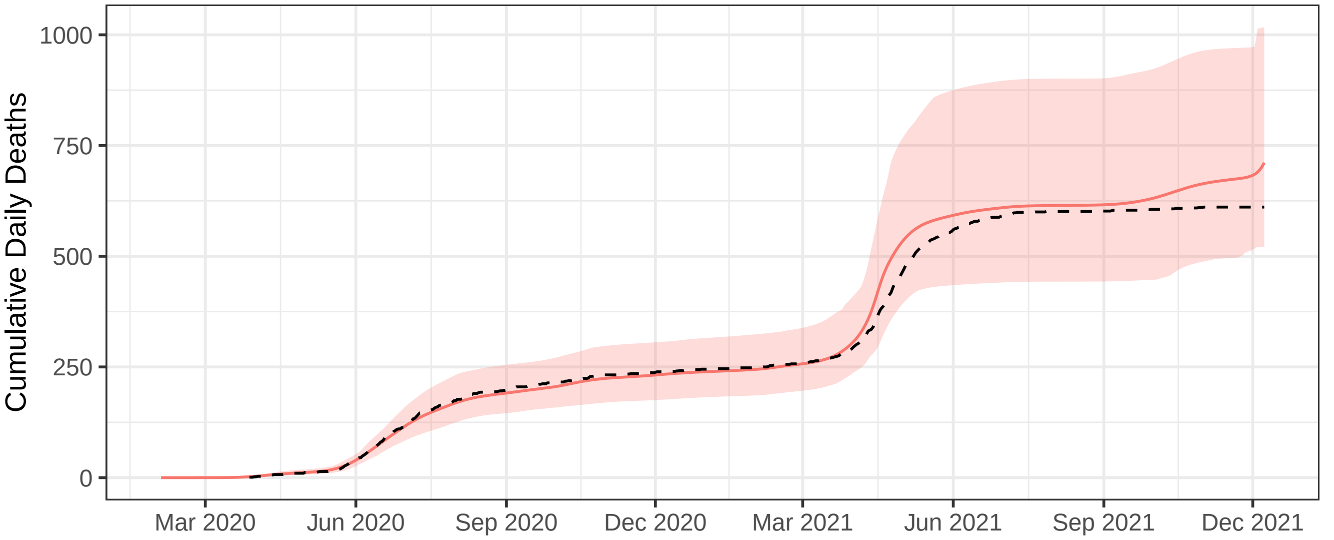

Daily deaths are included with reported COVID−19 deaths shown as points. The red line represents median model estimates of daily deaths, and the shaded region represents the 95% quantiles of the model estimated deaths. Plots of the cumulative estimated deaths are also shown, with the cumulative number of reported deaths represented by a dashed black line.

Romania

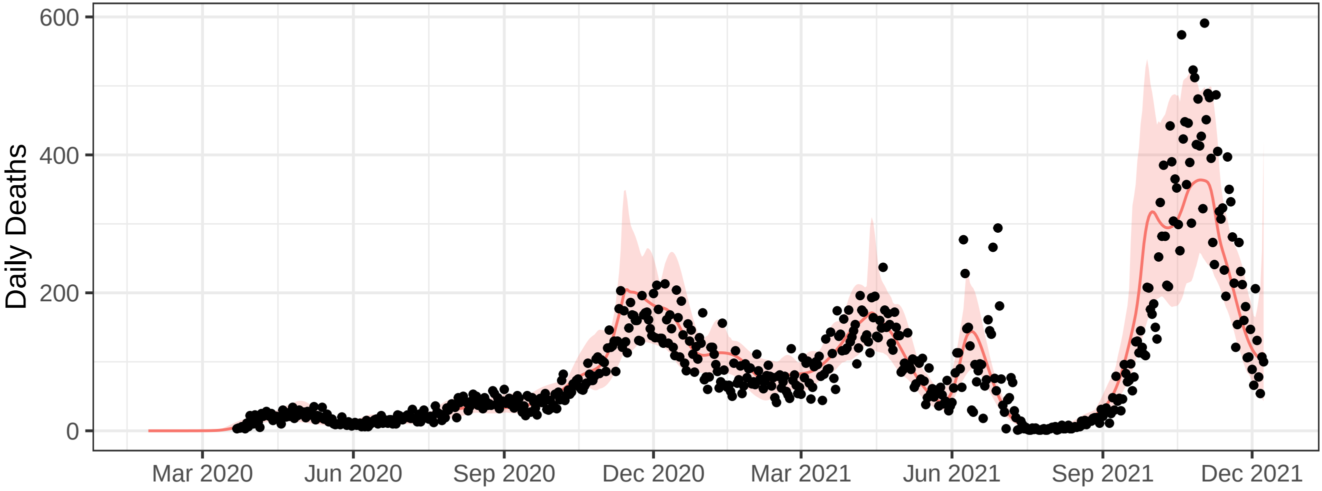

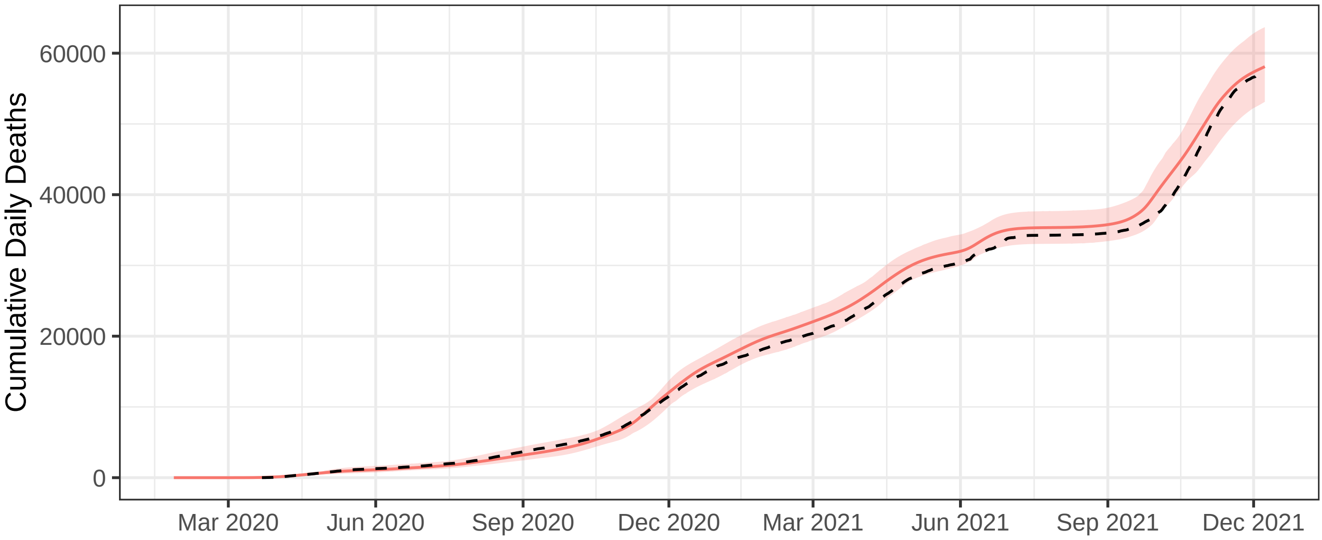

Daily deaths are included with reported COVID−19 deaths shown as points. The red line represents median model estimates of daily deaths, and the shaded region represents the 95% quantiles of the model estimated deaths. Plots of the cumulative estimated deaths are also shown, with the cumulative number of reported deaths represented by a dashed black line.

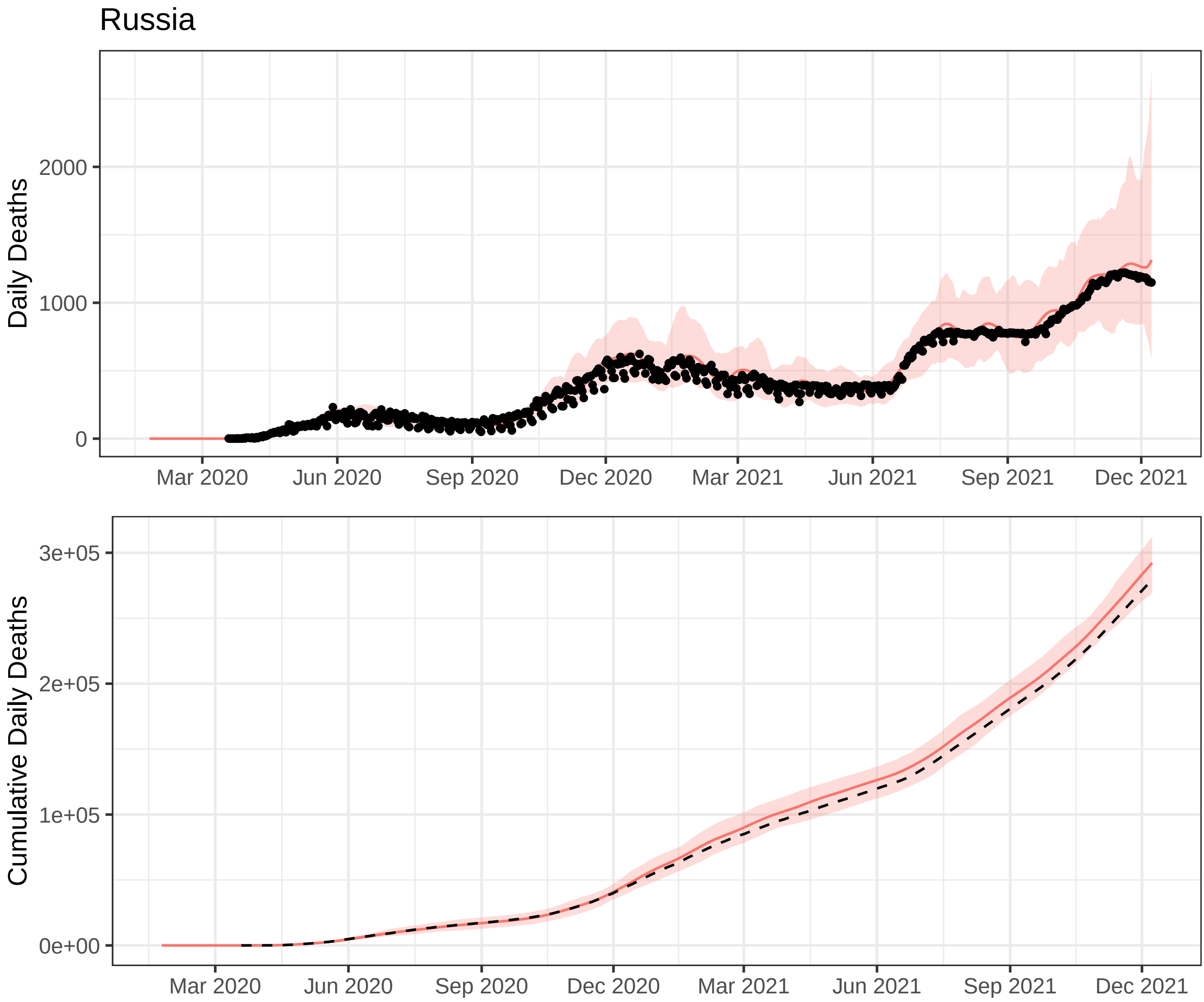

Daily deaths are included with reported COVID−19 deaths shown as points. The red line represents median model estimates of daily deaths, and the shaded region represents the 95% quantiles of the model estimated deaths. Plots of the cumulative estimated deaths are also shown, with the cumulative number of reported deaths represented by a dashed black line.

Rwanda

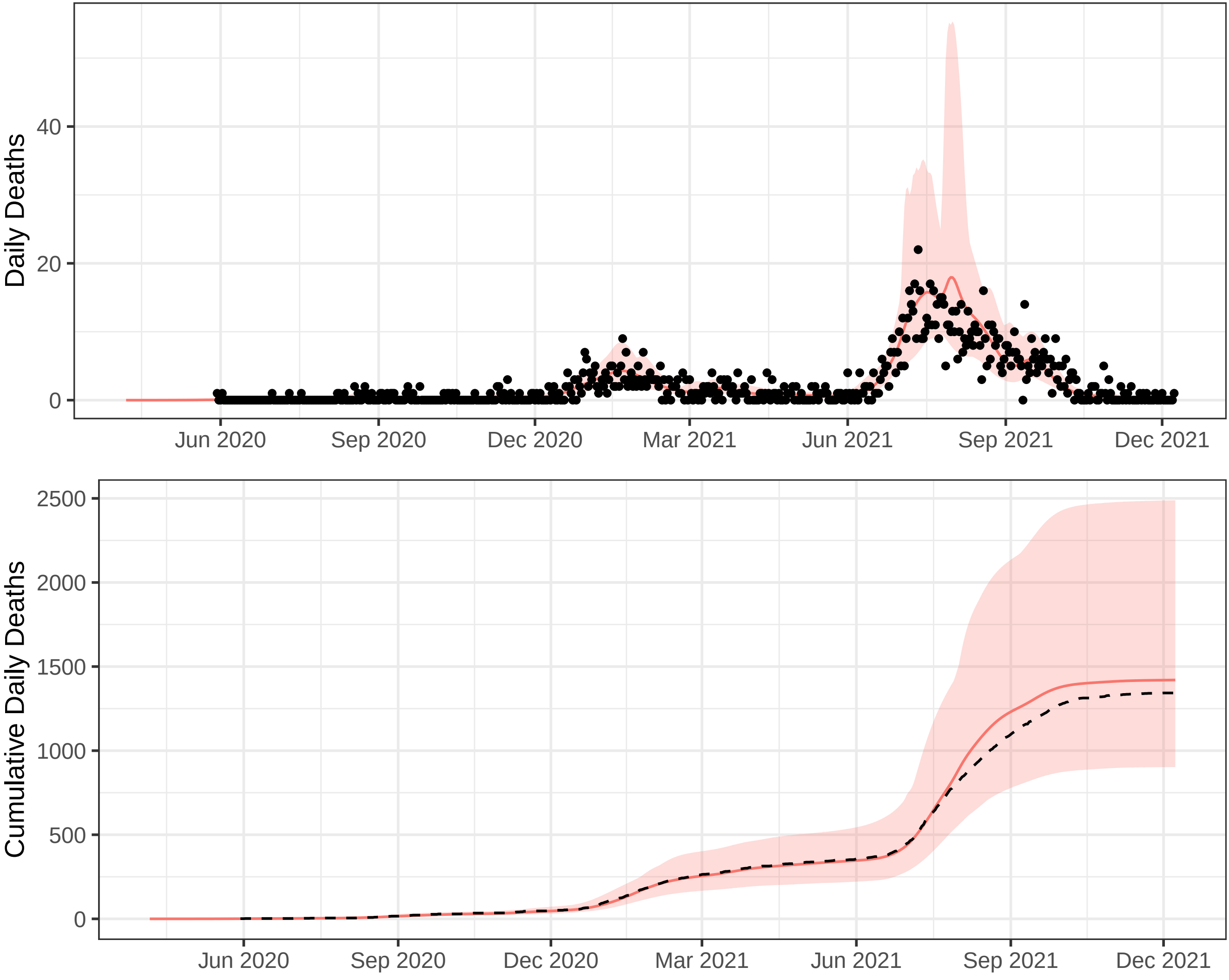

Daily deaths are included with reported COVID−19 deaths shown as points. The red line represents median model estimates of daily deaths, and the shaded region represents the 95% quantiles of the model estimated deaths. Plots of the cumulative estimated deaths are also shown, with the cumulative number of reported deaths represented by a dashed black line.

Saudi Arabia

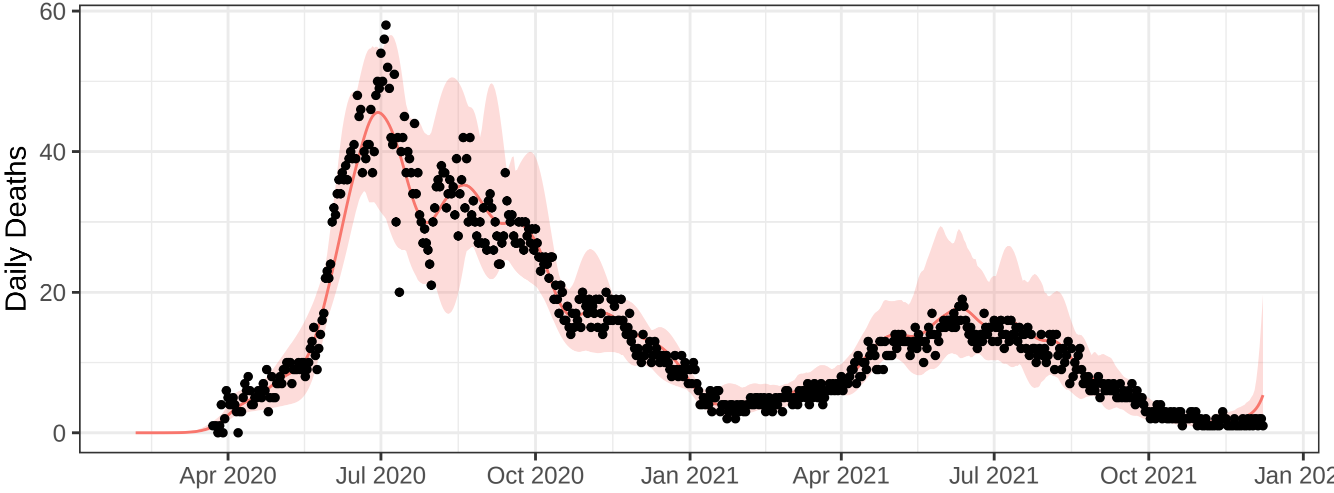

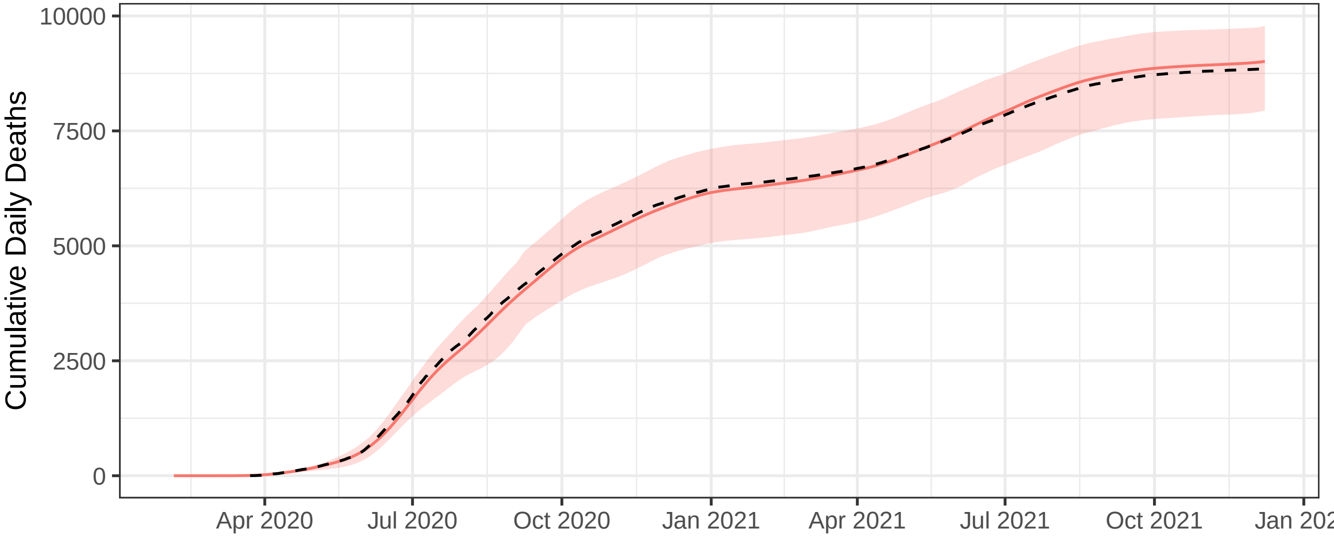

Daily deaths are included with reported COVID−19 deaths shown as points. The red line represents median model estimates of daily deaths, and the shaded region represents the 95% quantiles of the model estimated deaths. Plots of the cumulative estimated deaths are also shown, with the cumulative number of reported deaths represented by a dashed black line.

Sudan

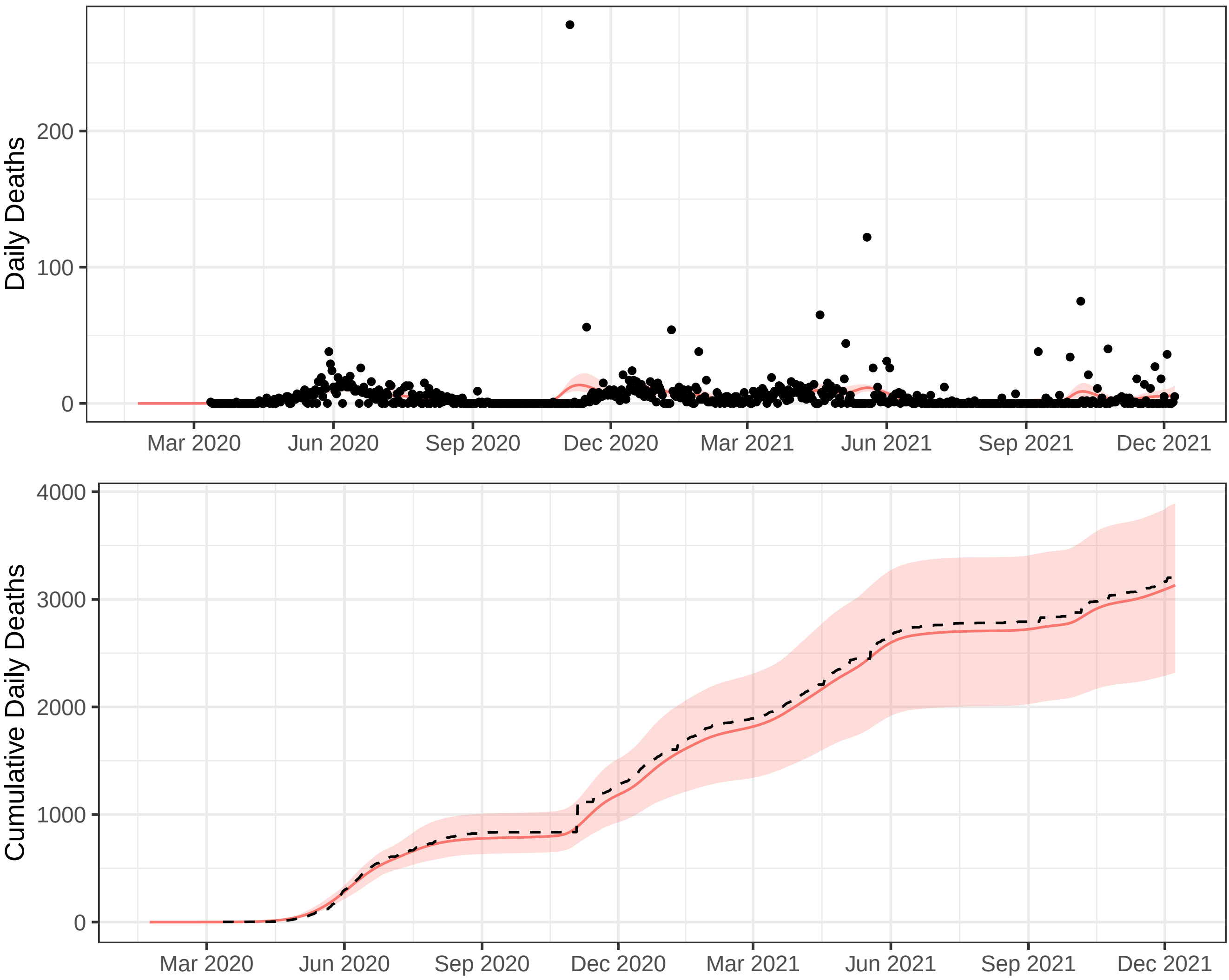

Daily deaths are included with reported COVID−19 deaths shown as points. The red line represents median model estimates of daily deaths, and the shaded region represents the 95% quantiles of the model estimated deaths. Plots of the cumulative estimated deaths are also shown, with the cumulative number of reported deaths represented by a dashed black line.

Senegal

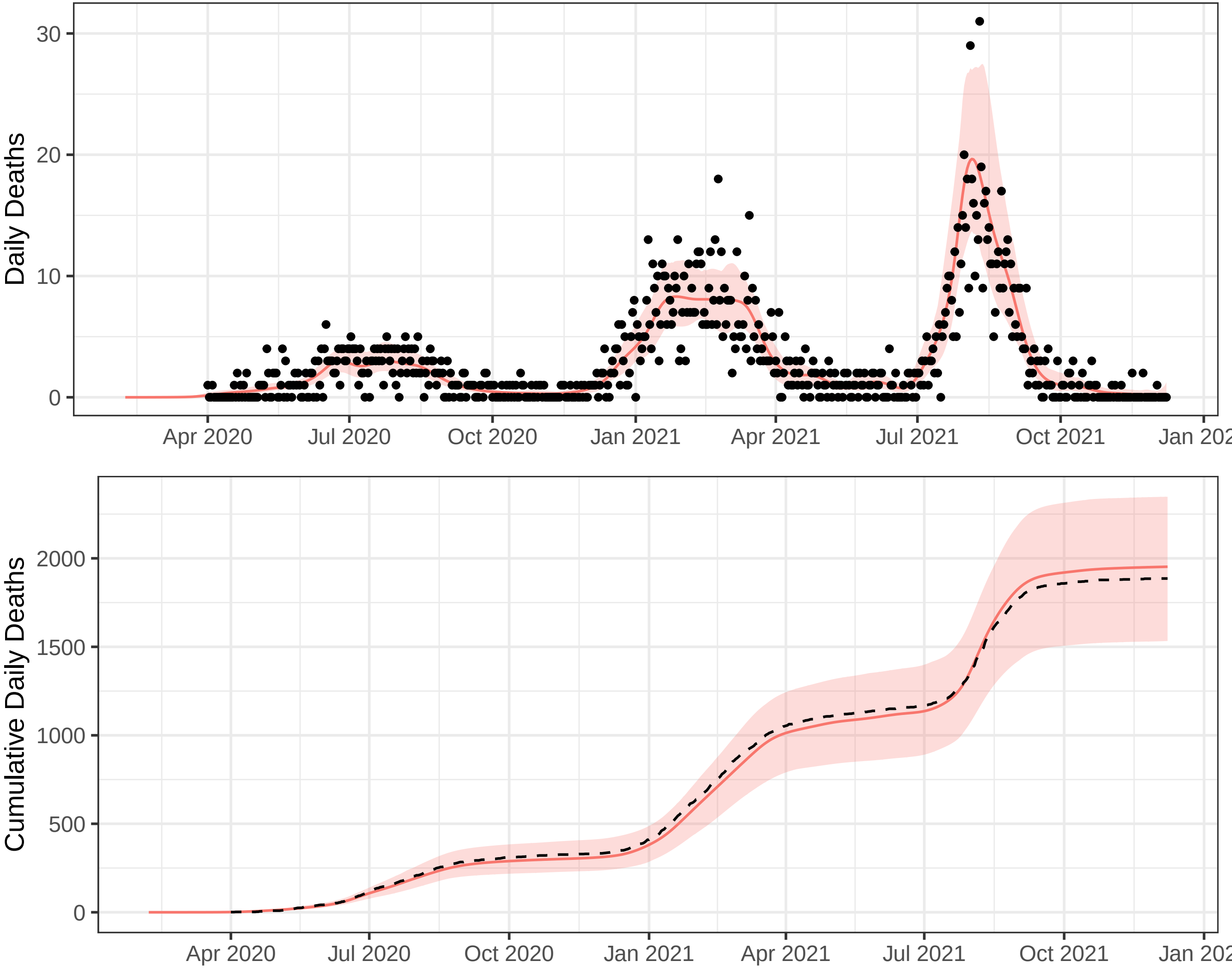

Daily deaths are included with reported COVID−19 deaths shown as points. The red line represents median model estimates of daily deaths, and the shaded region represents the 95% quantiles of the model estimated deaths. Plots of the cumulative estimated deaths are also shown, with the cumulative number of reported deaths represented by a dashed black line.

Singapore

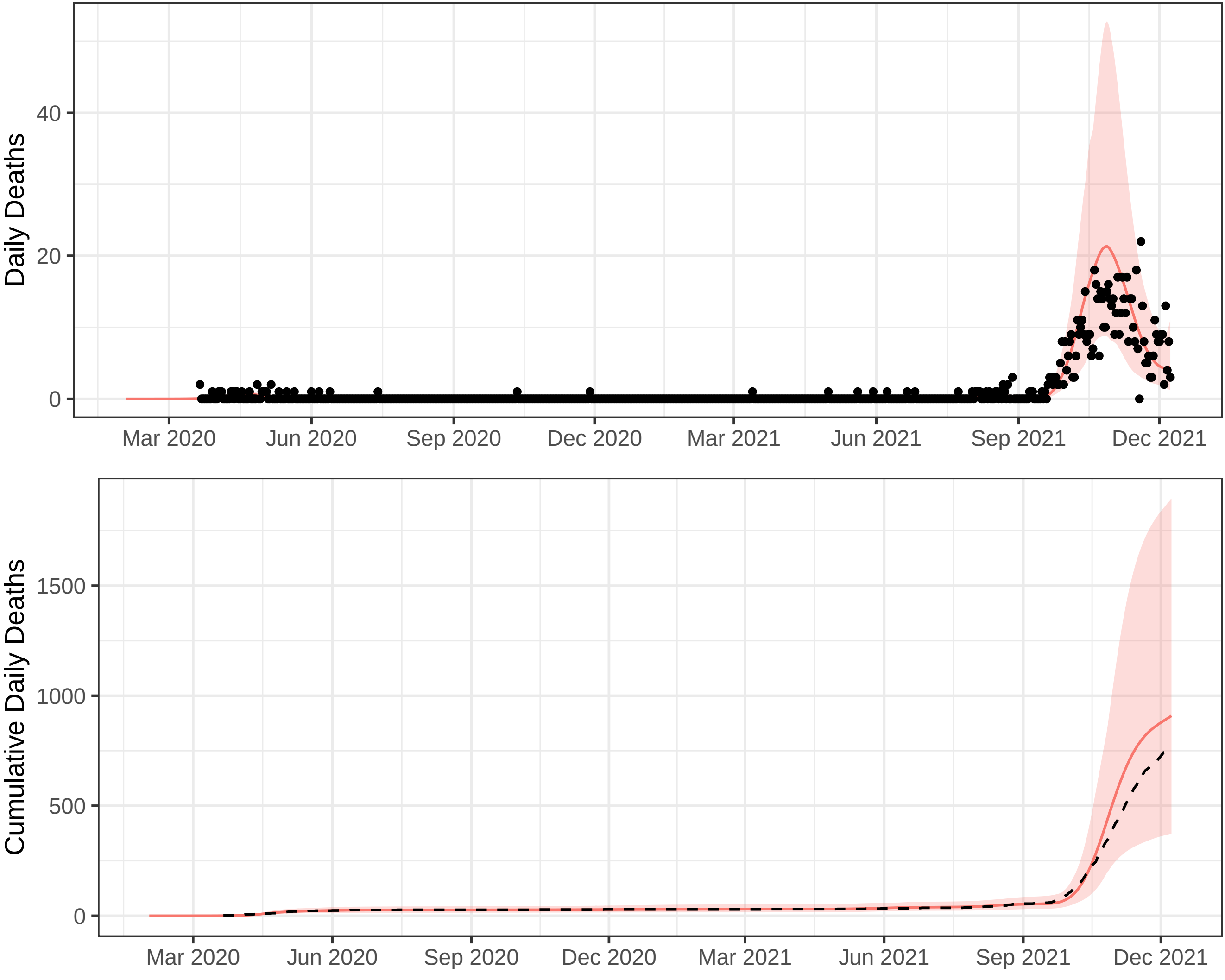

Daily deaths are included with reported COVID−19 deaths shown as points. The red line represents median model estimates of daily deaths, and the shaded region represents the 95% quantiles of the model estimated deaths. Plots of the cumulative estimated deaths are also shown, with the cumulative number of reported deaths represented by a dashed black line.

Sierra Leone

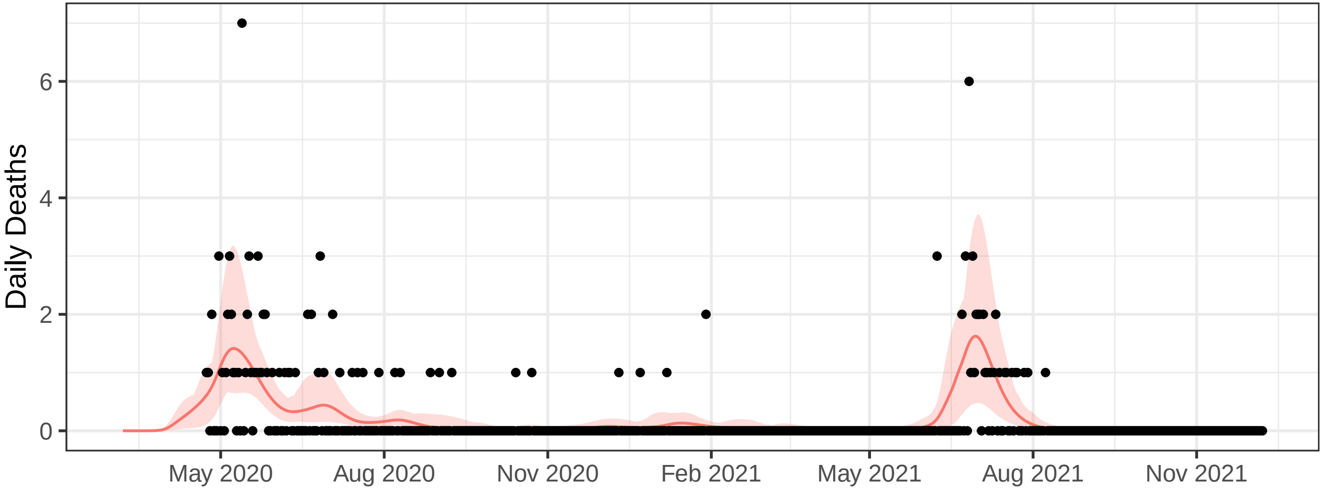

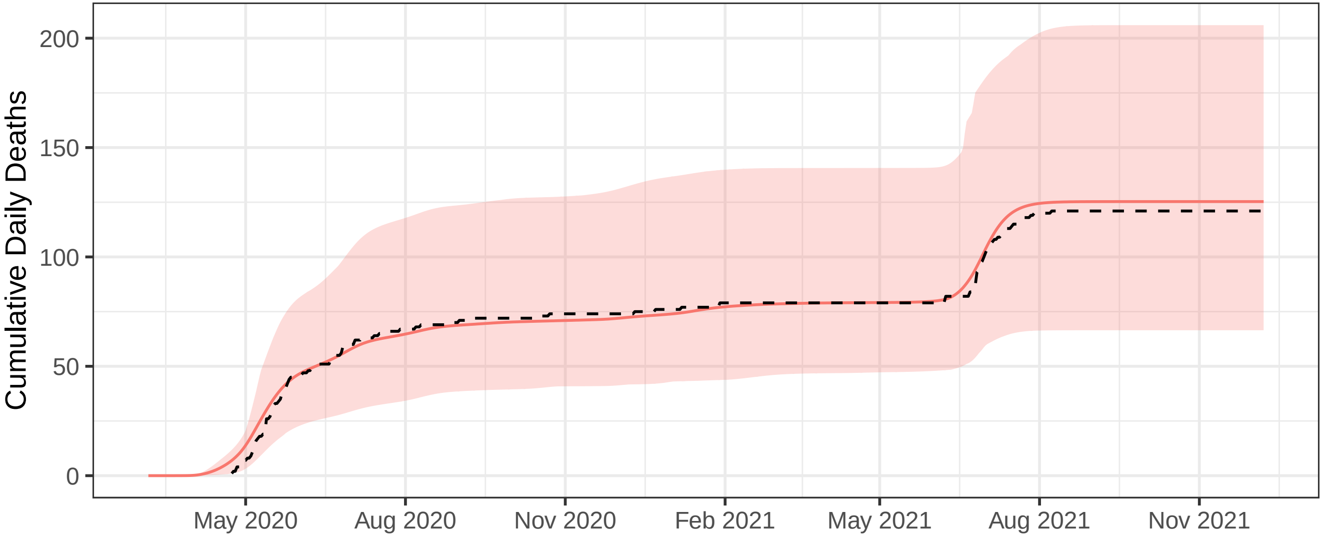

Daily deaths are included with reported COVID−19 deaths shown as points. The red line represents median model estimates of daily deaths, and the shaded region represents the 95% quantiles of the model estimated deaths. Plots of the cumulative estimated deaths are also shown, with the cumulative number of reported deaths represented by a dashed black line.

El Salvador

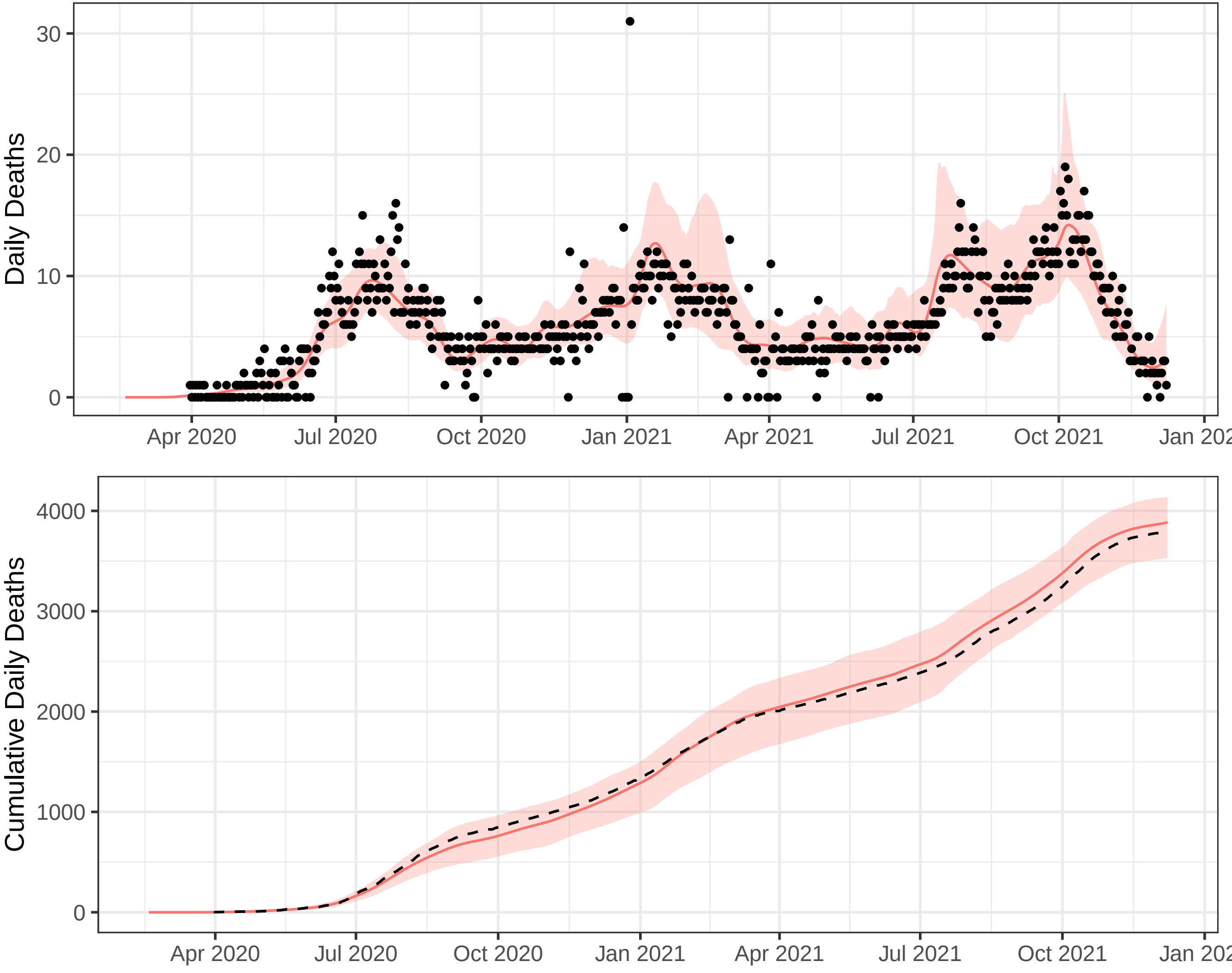

Daily deaths are included with reported COVID−19 deaths shown as points. The red line represents median model estimates of daily deaths, and the shaded region represents the 95% quantiles of the model estimated deaths. Plots of the cumulative estimated deaths are also shown, with the cumulative number of reported deaths represented by a dashed black line.

Somalia

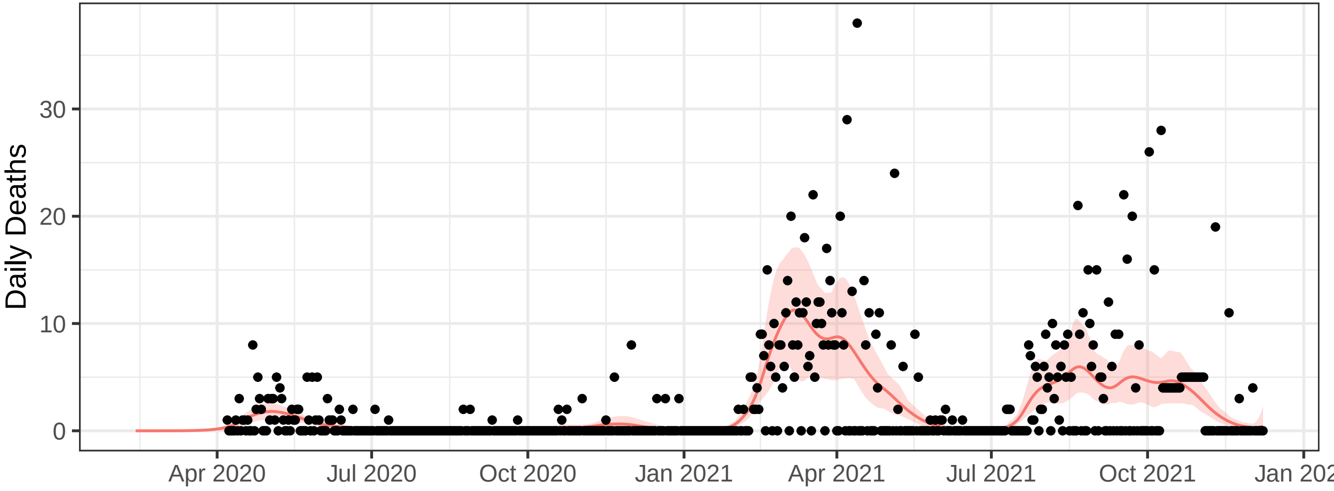

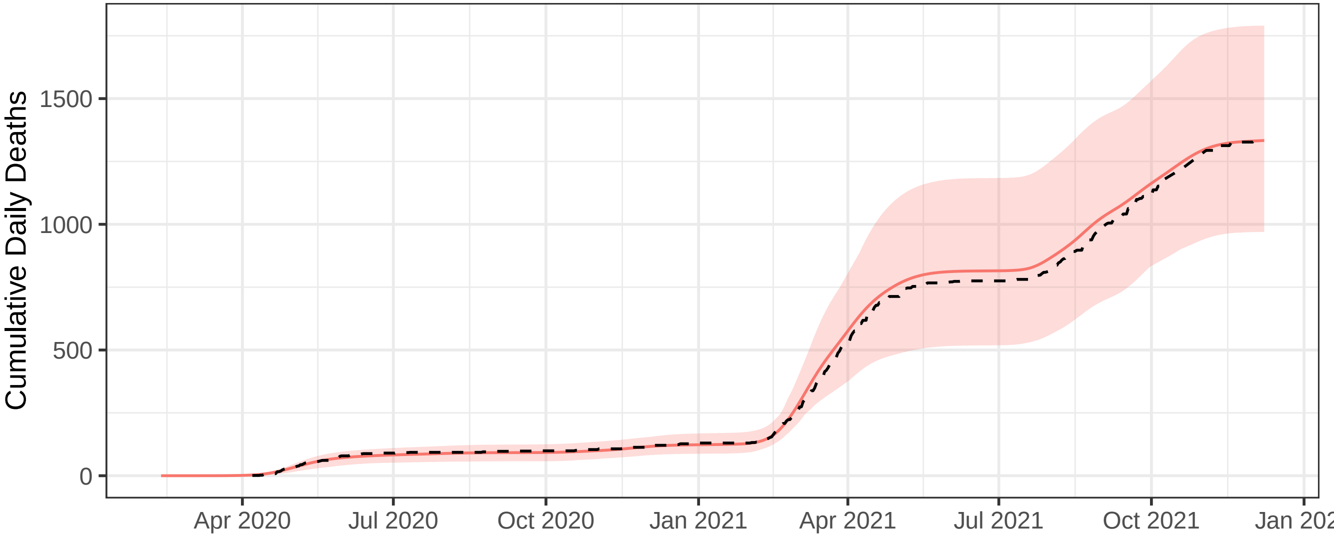

Daily deaths are included with reported COVID−19 deaths shown as points. The red line represents median model estimates of daily deaths, and the shaded region represents the 95% quantiles of the model estimated deaths. Plots of the cumulative estimated deaths are also shown, with the cumulative number of reported deaths represented by a dashed black line.

Serbia

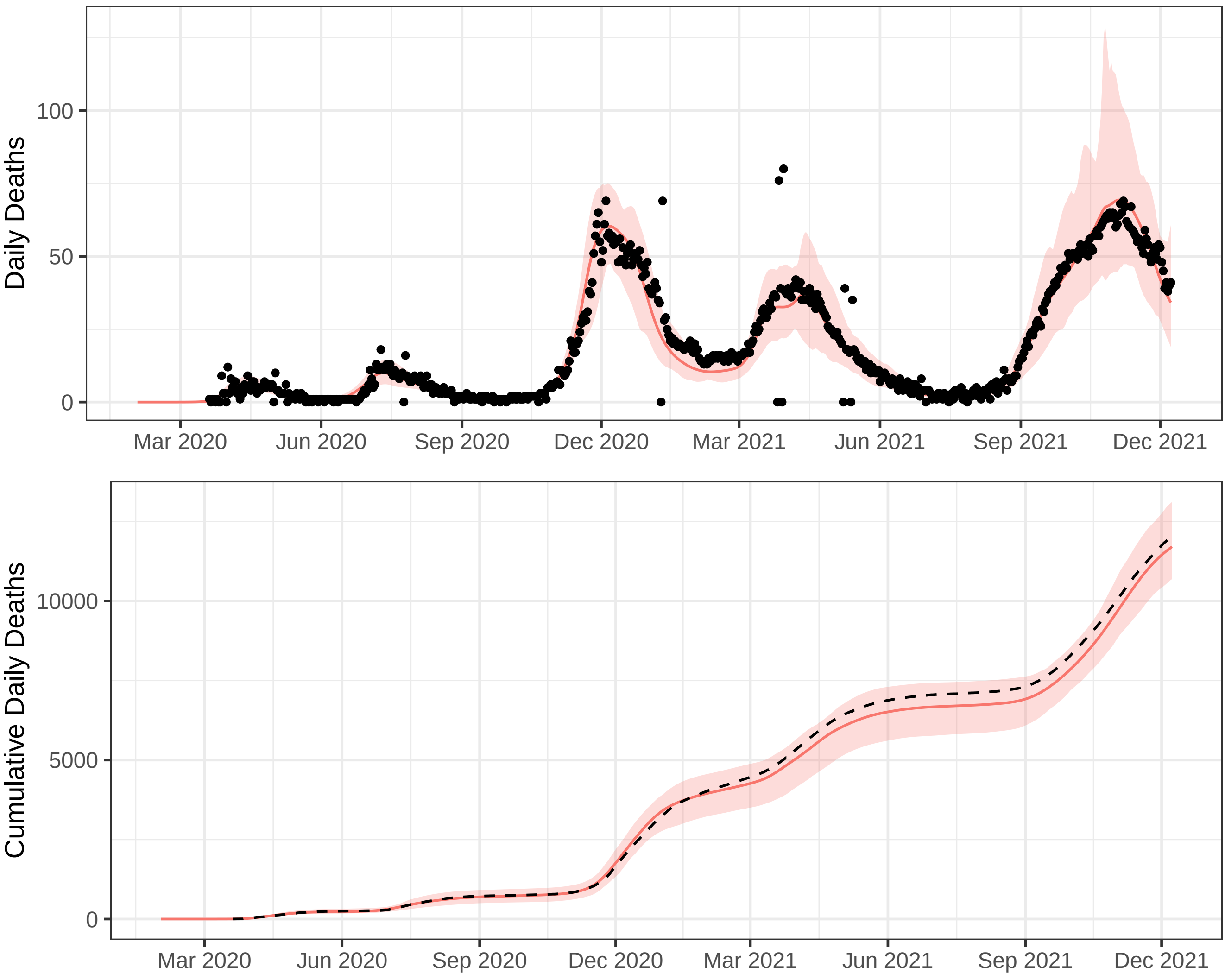

Daily deaths are included with reported COVID−19 deaths shown as points. The red line represents median model estimates of daily deaths, and the shaded region represents the 95% quantiles of the model estimated deaths. Plots of the cumulative estimated deaths are also shown, with the cumulative number of reported deaths represented by a dashed black line.

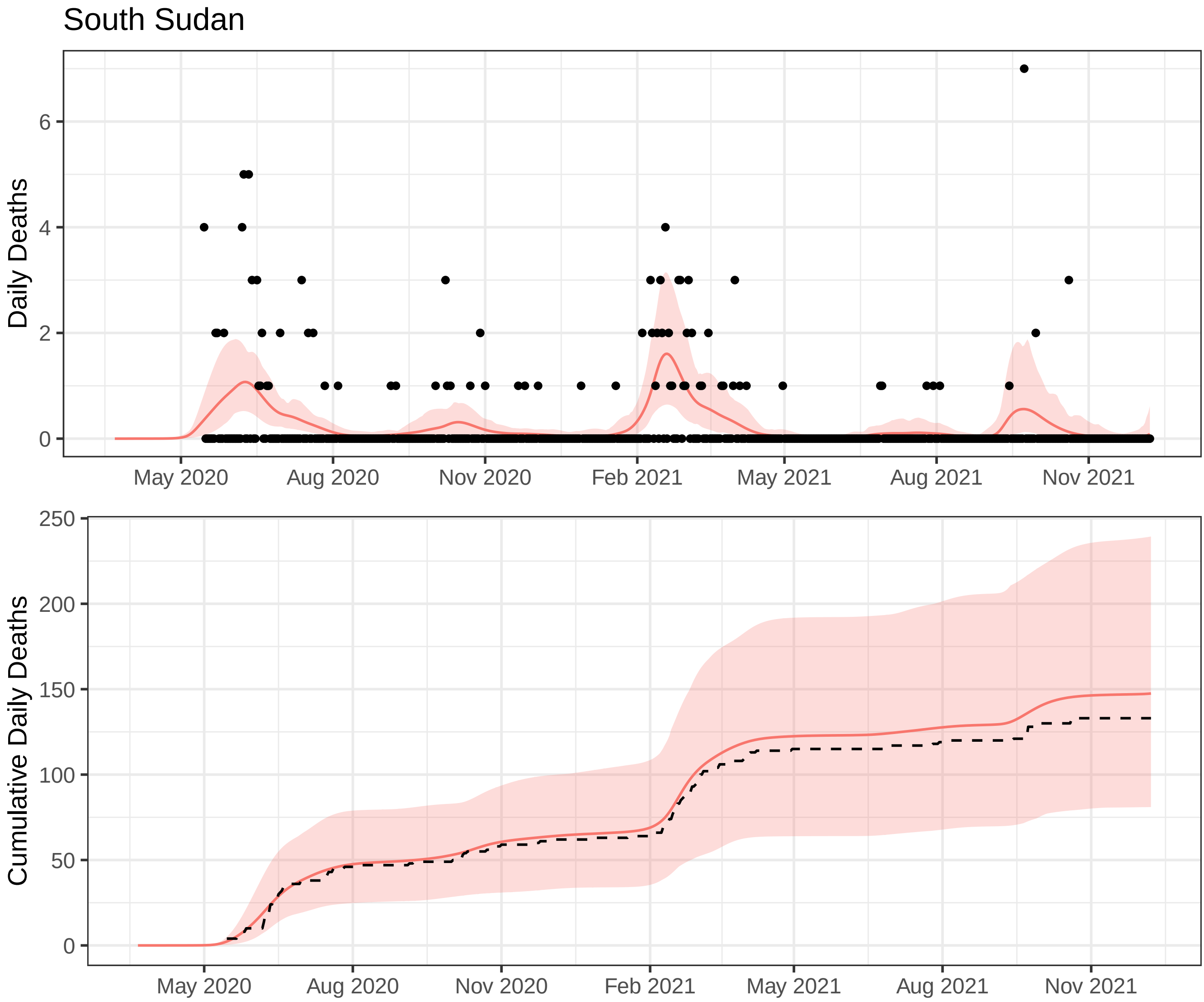

Daily deaths are included with reported COVID−19 deaths shown as points. The red line represents median model estimates of daily deaths, and the shaded region represents the 95% quantiles of the model estimated deaths. Plots of the cumulative estimated deaths are also shown, with the cumulative number of reported deaths represented by a dashed black line.

Sao Tome and Principe

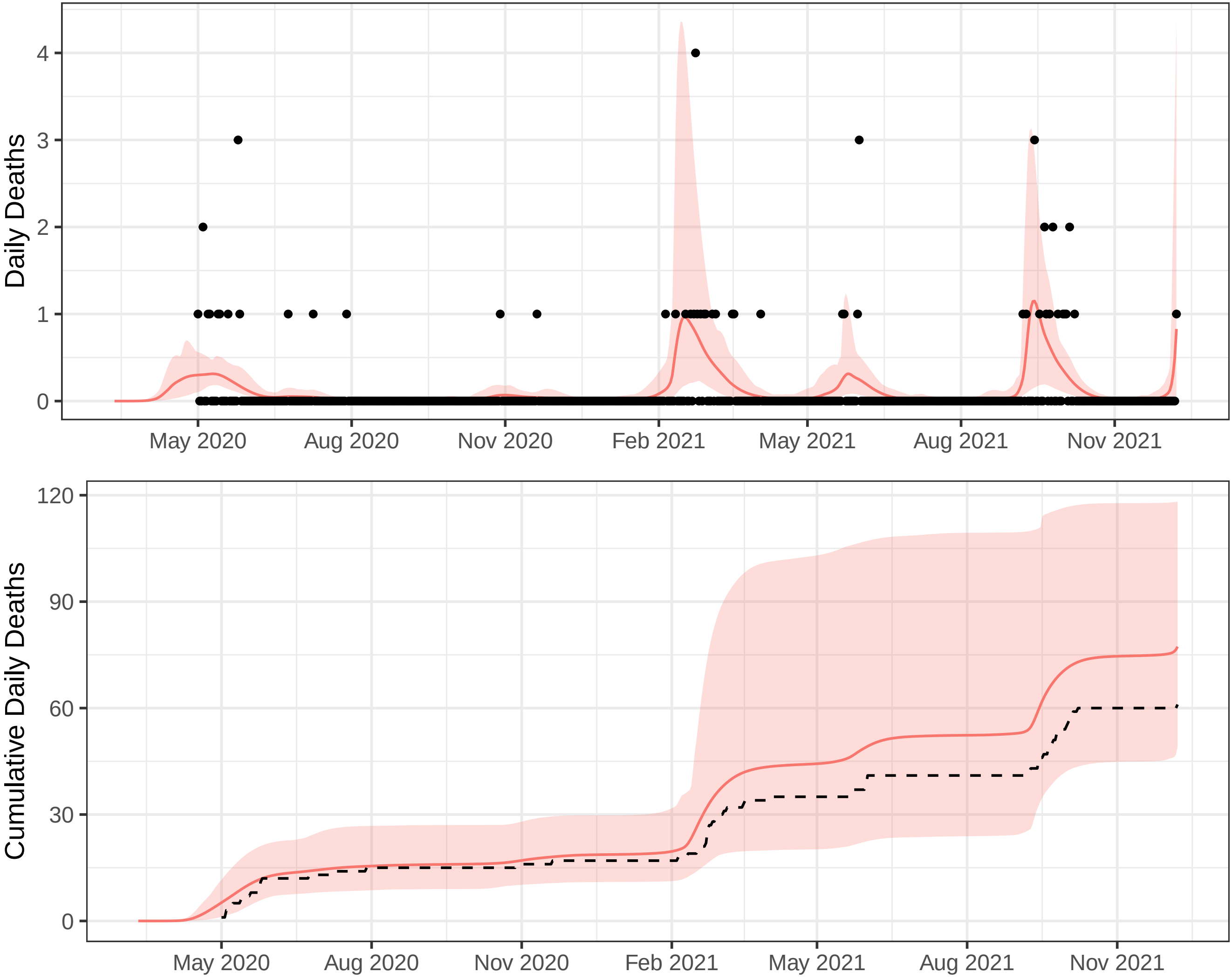

Daily deaths are included with reported COVID−19 deaths shown as points. The red line represents median model estimates of daily deaths, and the shaded region represents the 95% quantiles of the model estimated deaths. Plots of the cumulative estimated deaths are also shown, with the cumulative number of reported deaths represented by a dashed black line.

Suriname

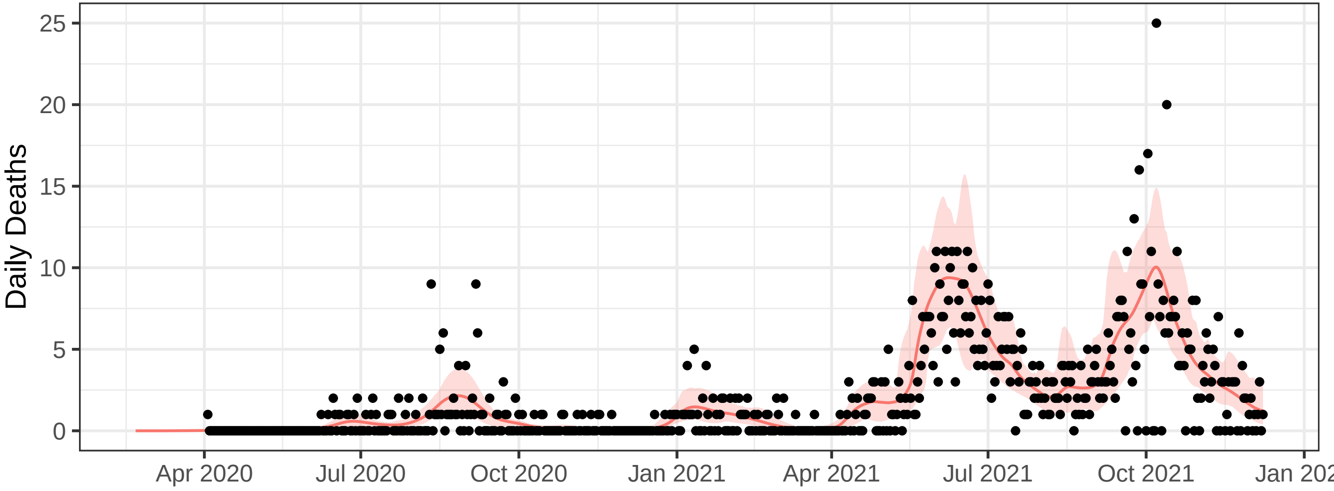

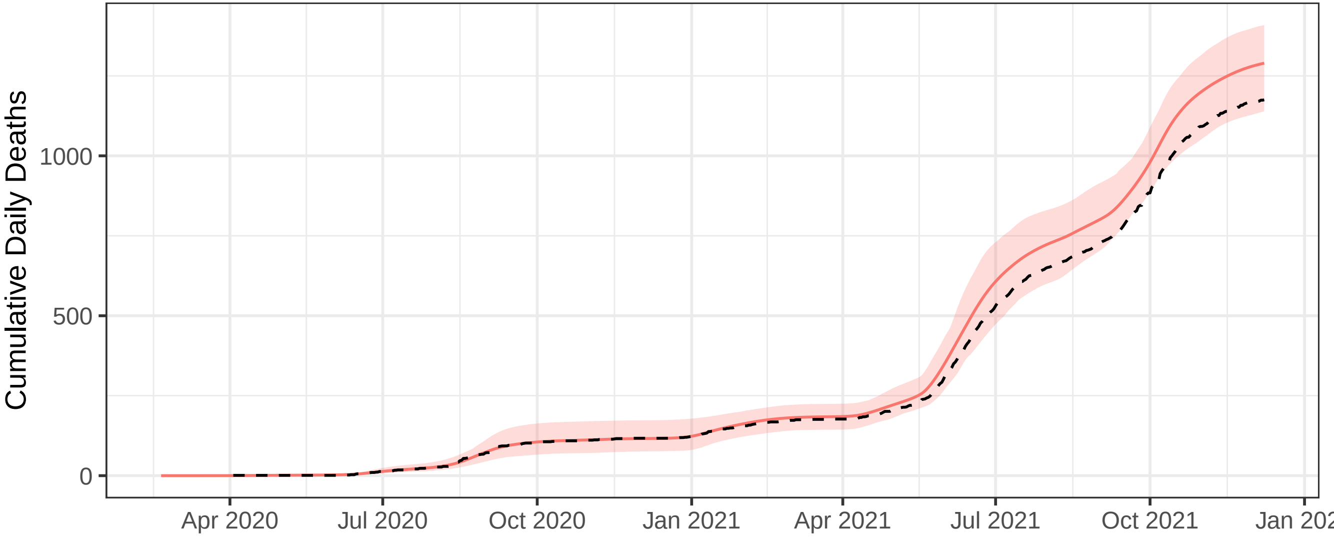

Daily deaths are included with reported COVID−19 deaths shown as points. The red line represents median model estimates of daily deaths, and the shaded region represents the 95% quantiles of the model estimated deaths. Plots of the cumulative estimated deaths are also shown, with the cumulative number of reported deaths represented by a dashed black line.

Slovakia

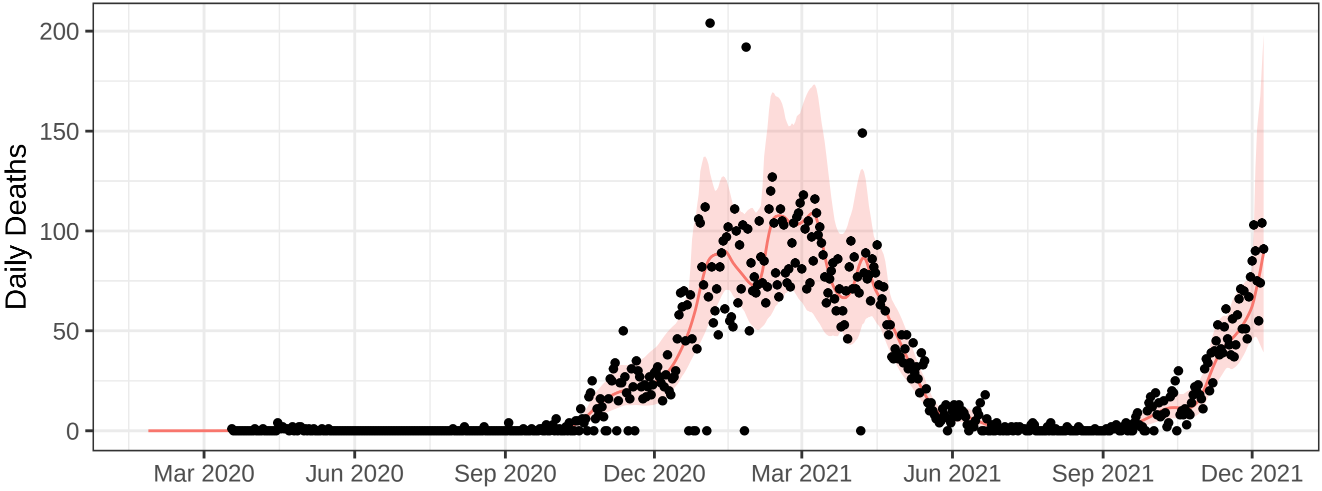

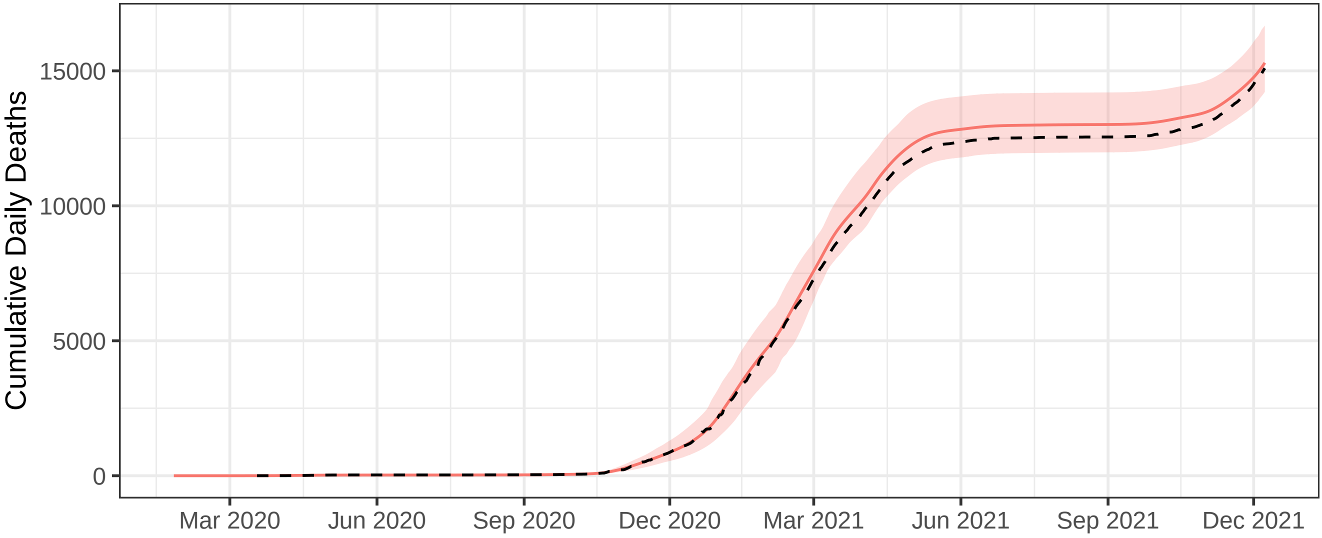

Daily deaths are included with reported COVID−19 deaths shown as points. The red line represents median model estimates of daily deaths, and the shaded region represents the 95% quantiles of the model estimated deaths. Plots of the cumulative estimated deaths are also shown, with the cumulative number of reported deaths represented by a dashed black line.

Slovenia

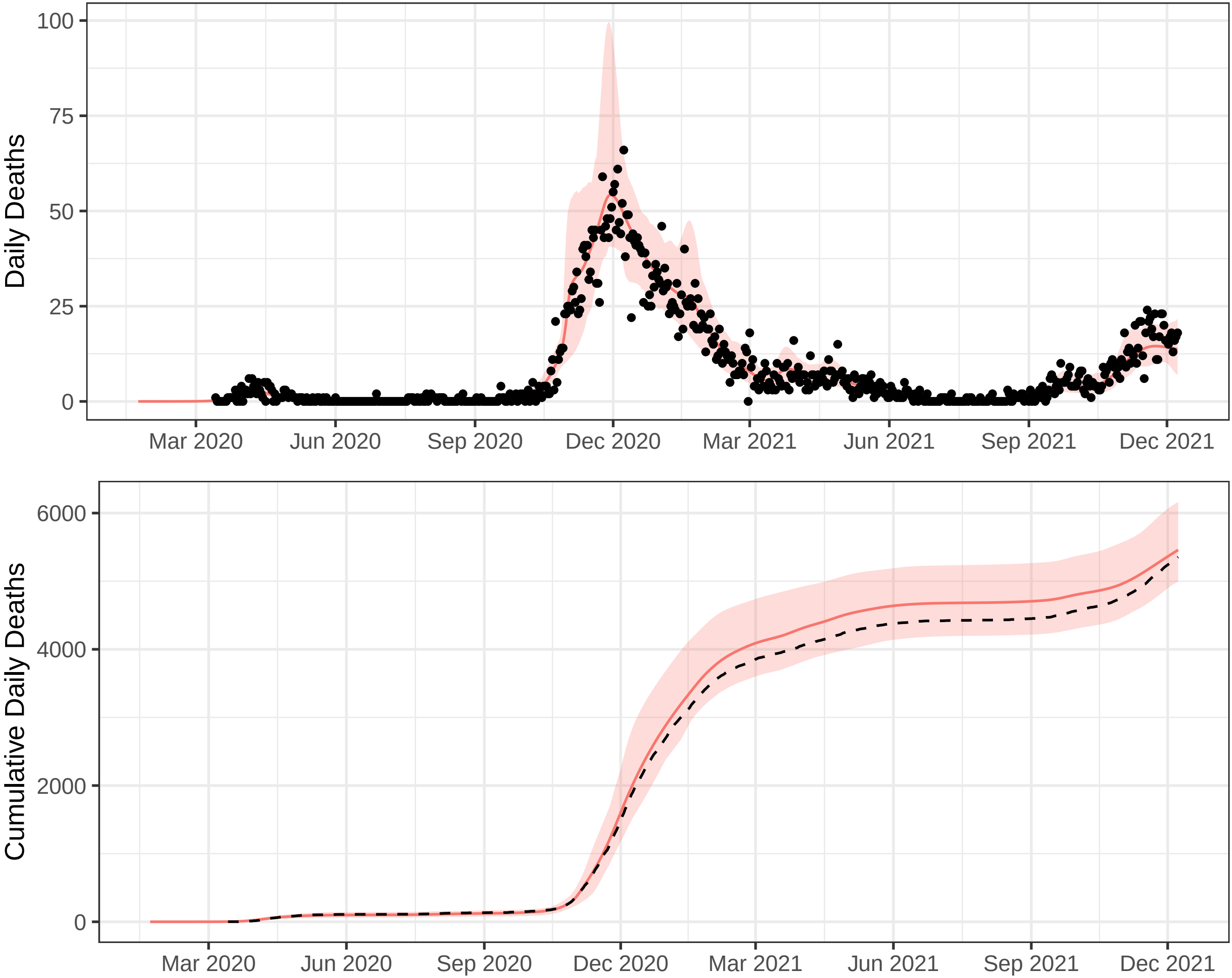

Daily deaths are included with reported COVID−19 deaths shown as points. The red line represents median model estimates of daily deaths, and the shaded region represents the 95% quantiles of the model estimated deaths. Plots of the cumulative estimated deaths are also shown, with the cumulative number of reported deaths represented by a dashed black line.

Sweden

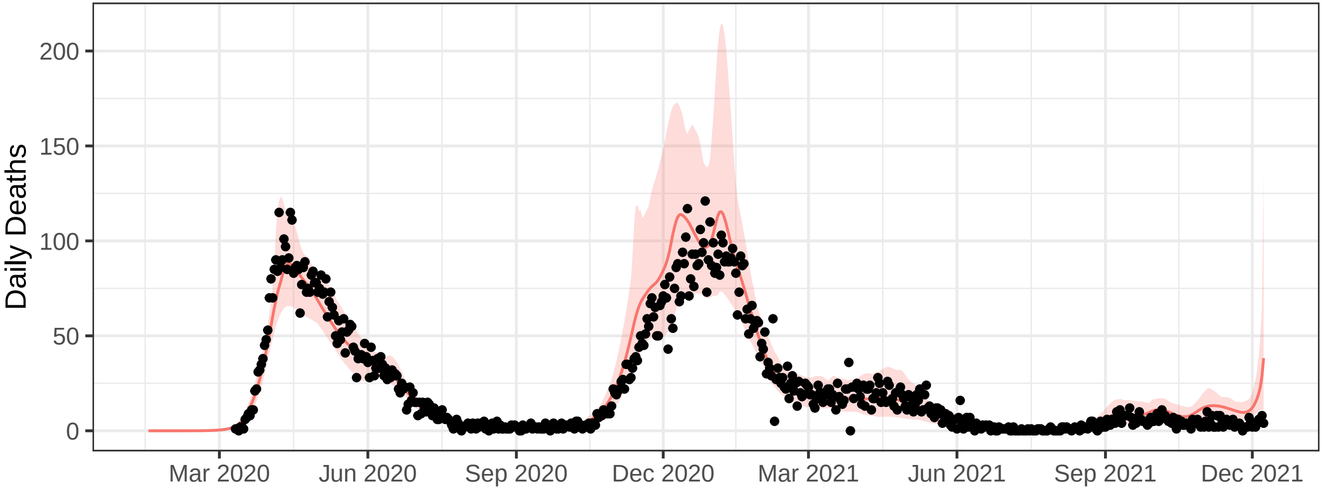

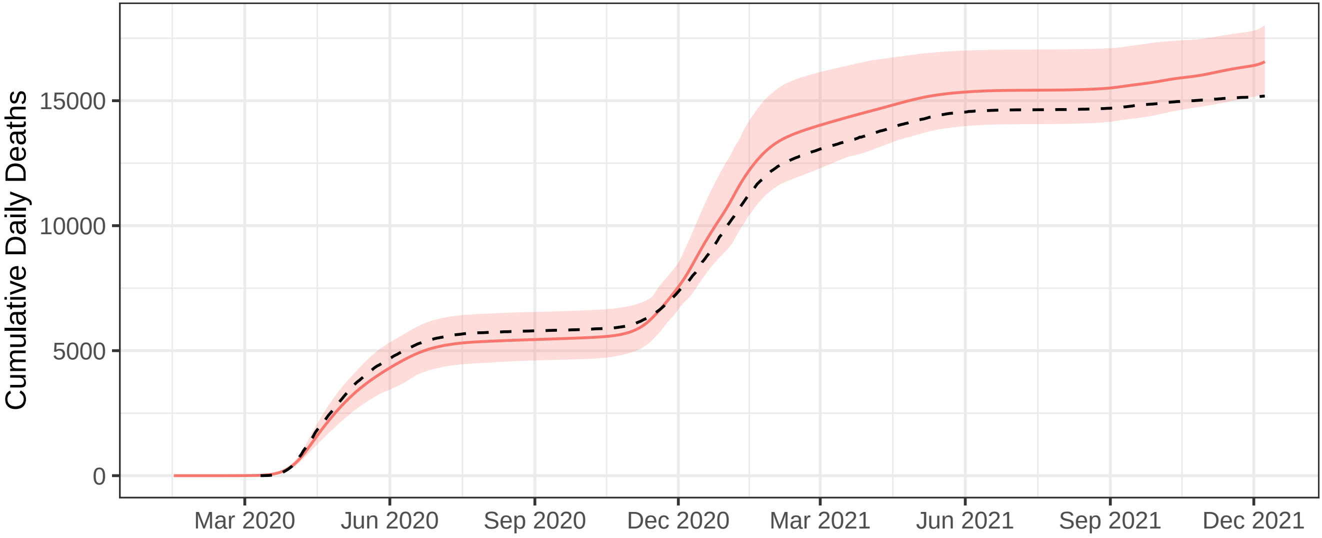

Daily deaths are included with reported COVID−19 deaths shown as points. The red line represents median model estimates of daily deaths, and the shaded region represents the 95% quantiles of the model estimated deaths. Plots of the cumulative estimated deaths are also shown, with the cumulative number of reported deaths represented by a dashed black line.

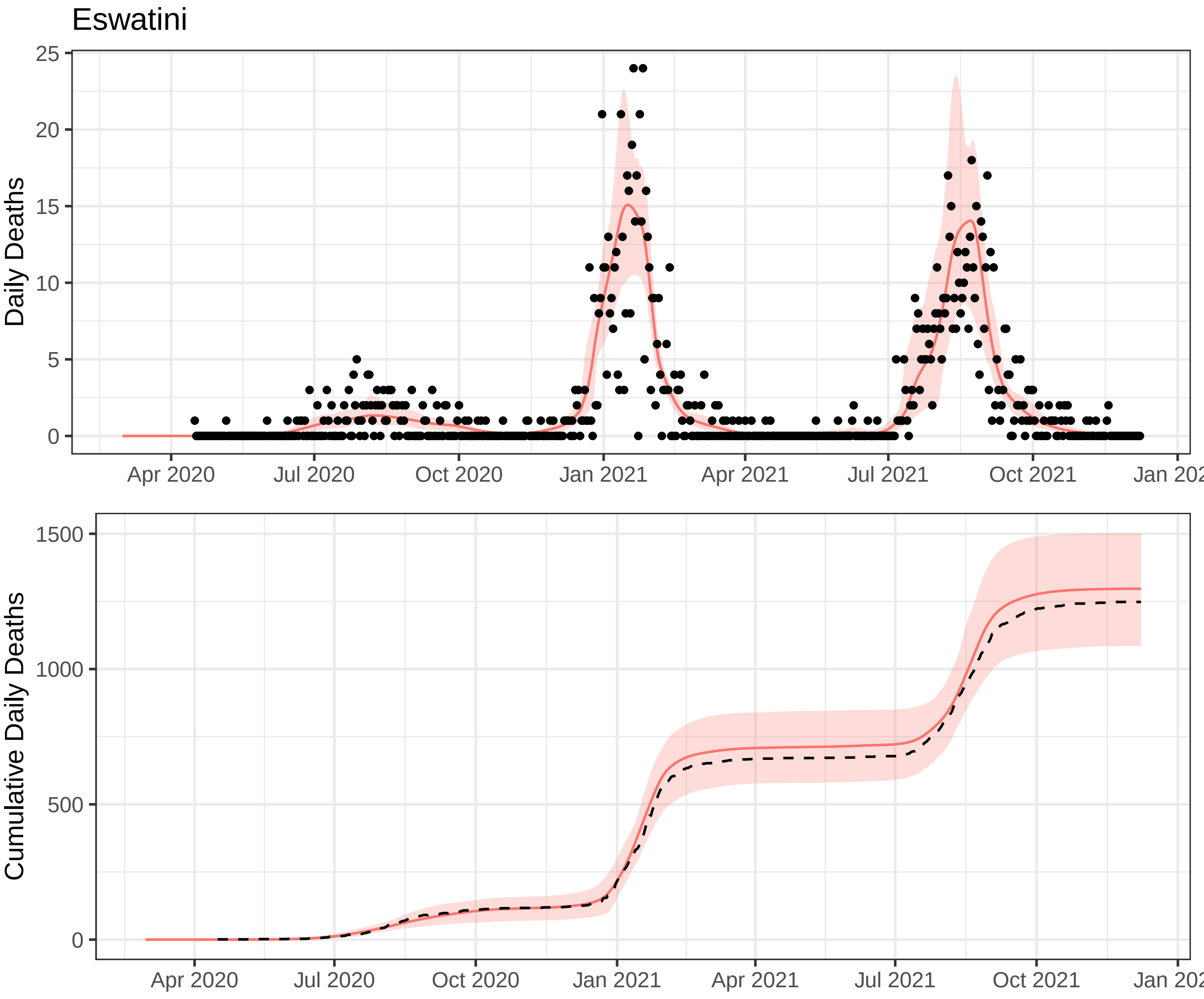

Daily deaths are included with reported COVID−19 deaths shown as points. The red line represents median model estimates of daily deaths, and the shaded region represents the 95% quantiles of the model estimated deaths. Plots of the cumulative estimated deaths are also shown, with the cumulative number of reported deaths represented by a dashed black line.

Seychelles

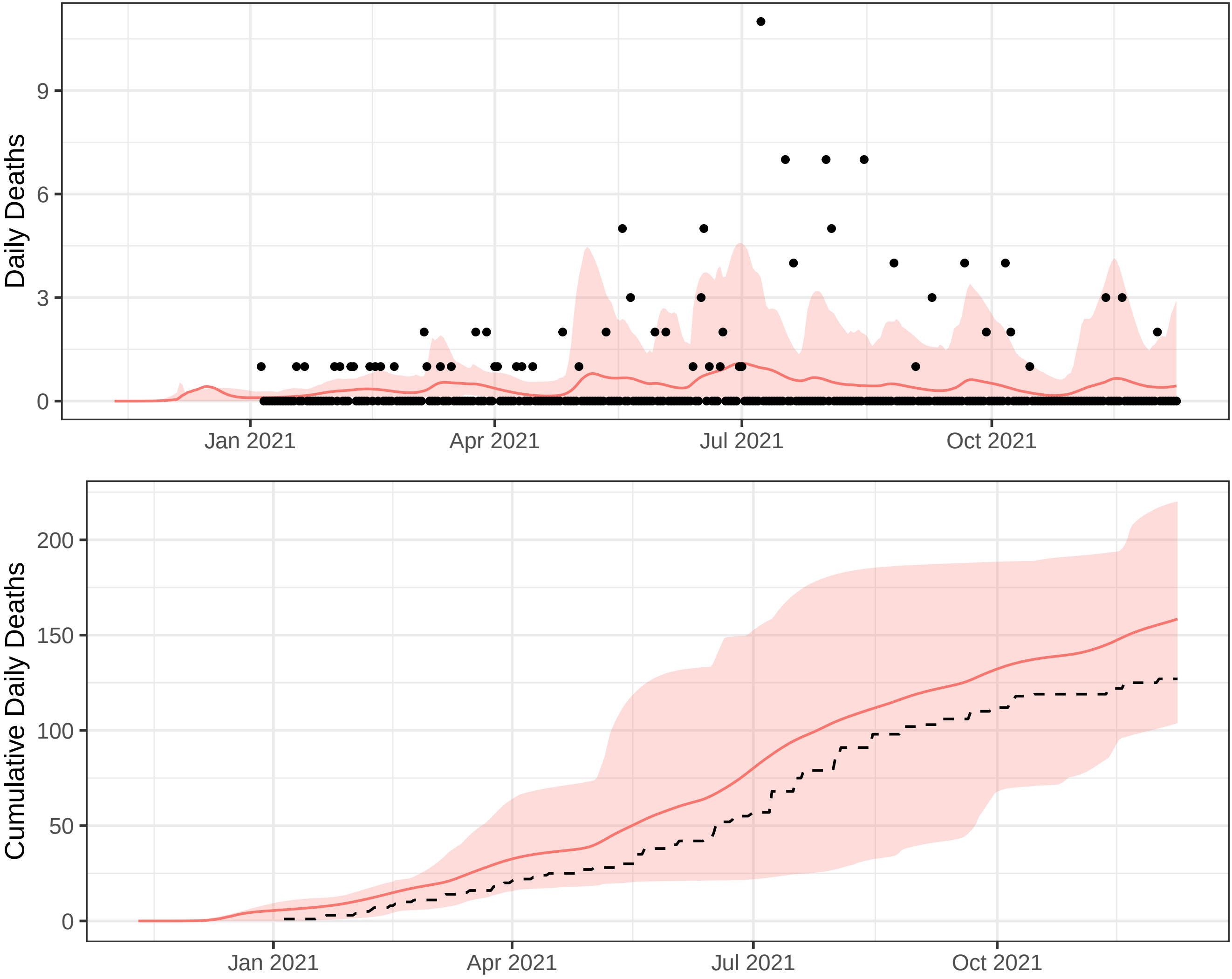

Daily deaths are included with reported COVID−19 deaths shown as points. The red line represents median model estimates of daily deaths, and the shaded region represents the 95% quantiles of the model estimated deaths. Plots of the cumulative estimated deaths are also shown, with the cumulative number of reported deaths represented by a dashed black line.

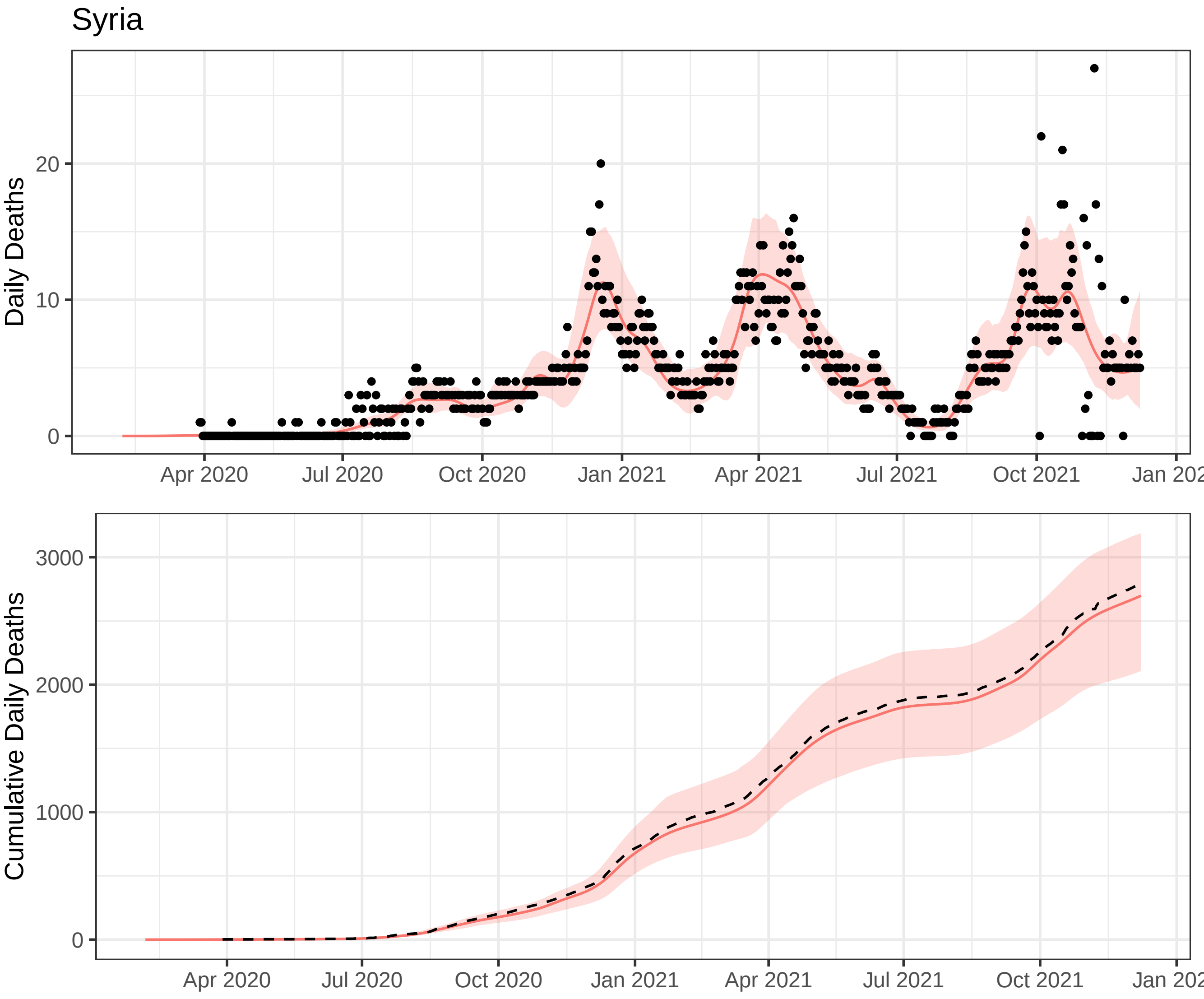

Daily deaths are included with reported COVID−19 deaths shown as points. The red line represents median model estimates of daily deaths, and the shaded region represents the 95% quantiles of the model estimated deaths. Plots of the cumulative estimated deaths are also shown, with the cumulative number of reported deaths represented by a dashed black line.

Chad

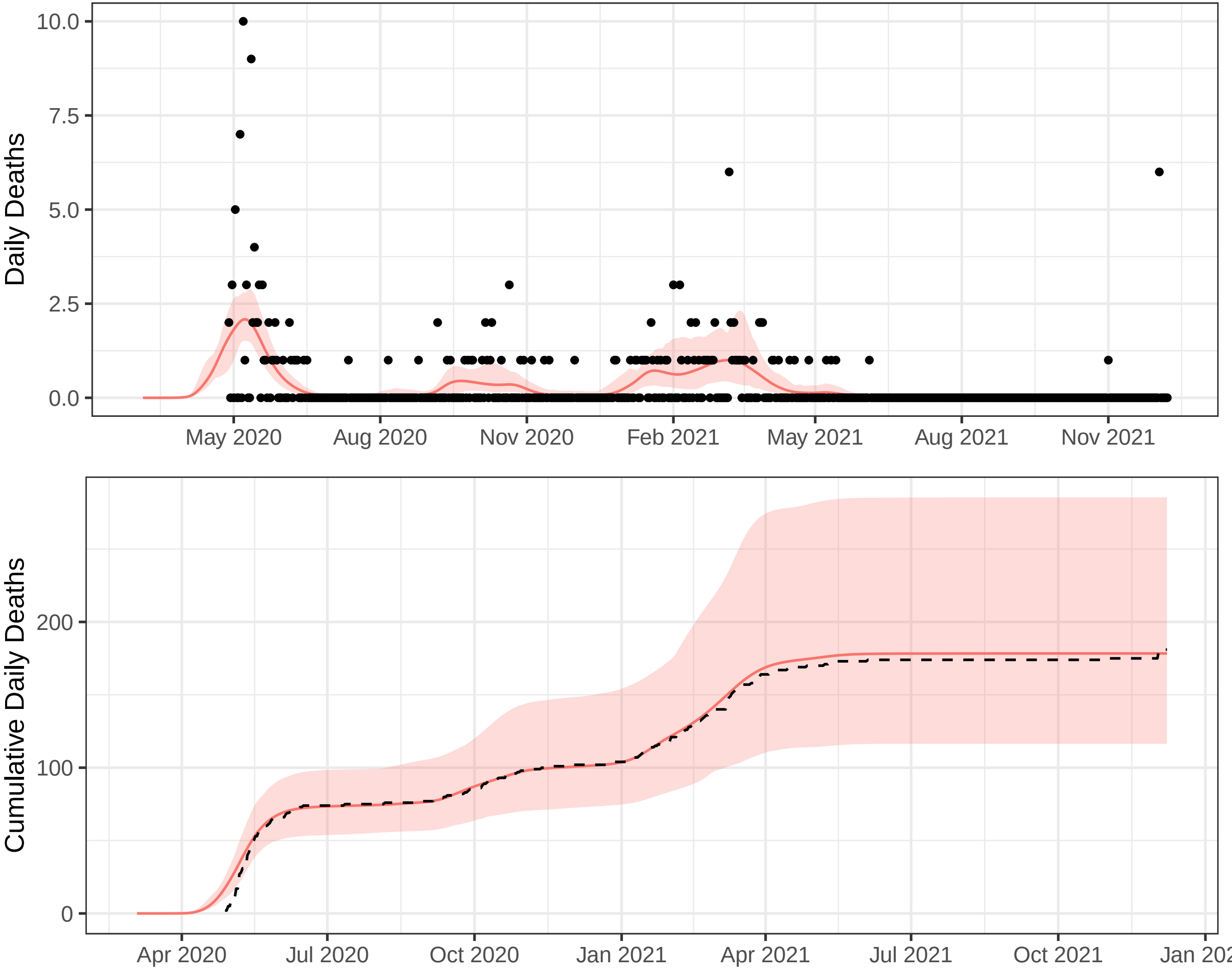

Daily deaths are included with reported COVID−19 deaths shown as points. The red line represents median model estimates of daily deaths, and the shaded region represents the 95% quantiles of the model estimated deaths. Plots of the cumulative estimated deaths are also shown, with the cumulative number of reported deaths represented by a dashed black line.

Togo

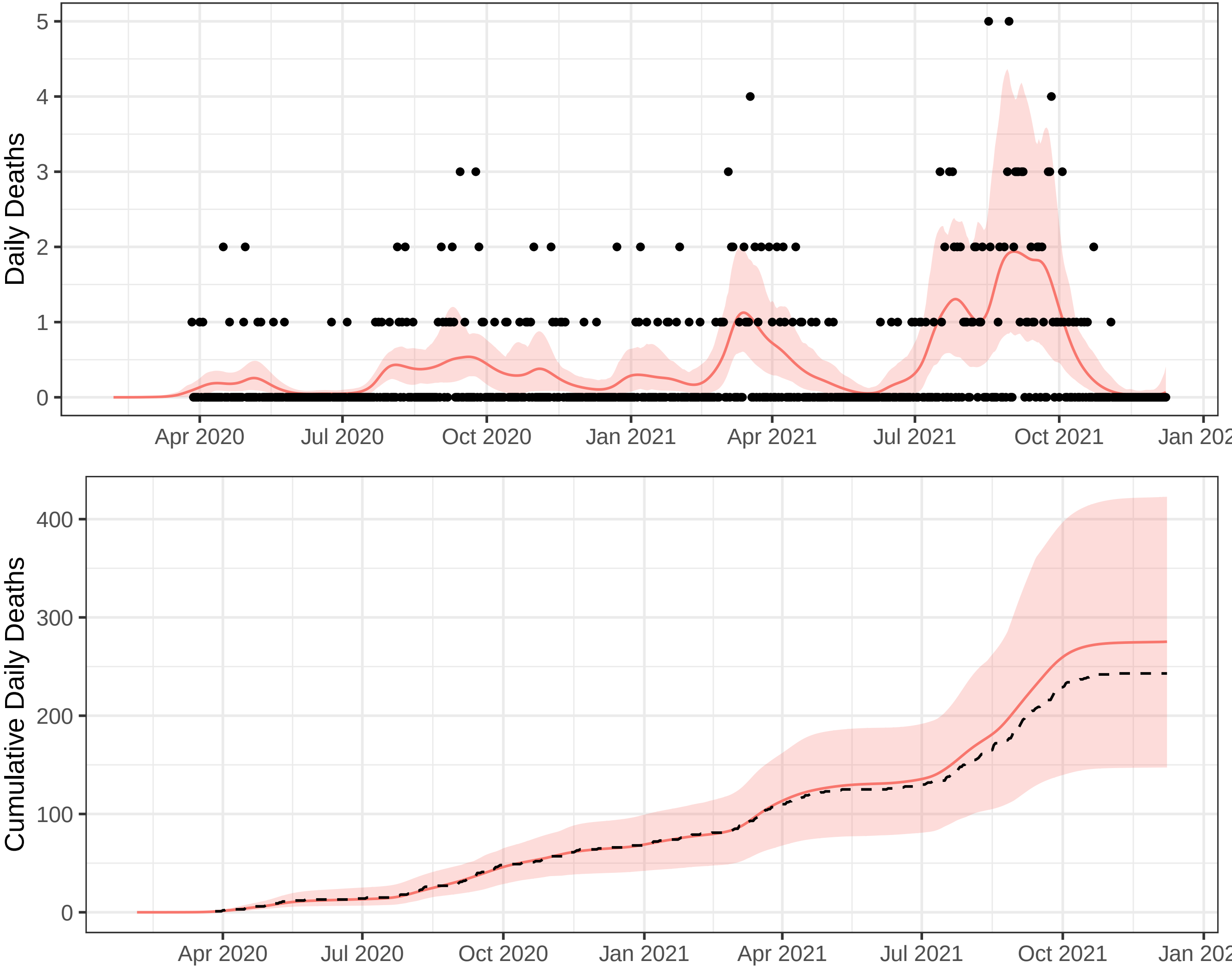

Daily deaths are included with reported COVID−19 deaths shown as points. The red line represents median model estimates of daily deaths, and the shaded region represents the 95% quantiles of the model estimated deaths. Plots of the cumulative estimated deaths are also shown, with the cumulative number of reported deaths represented by a dashed black line.

Thailand

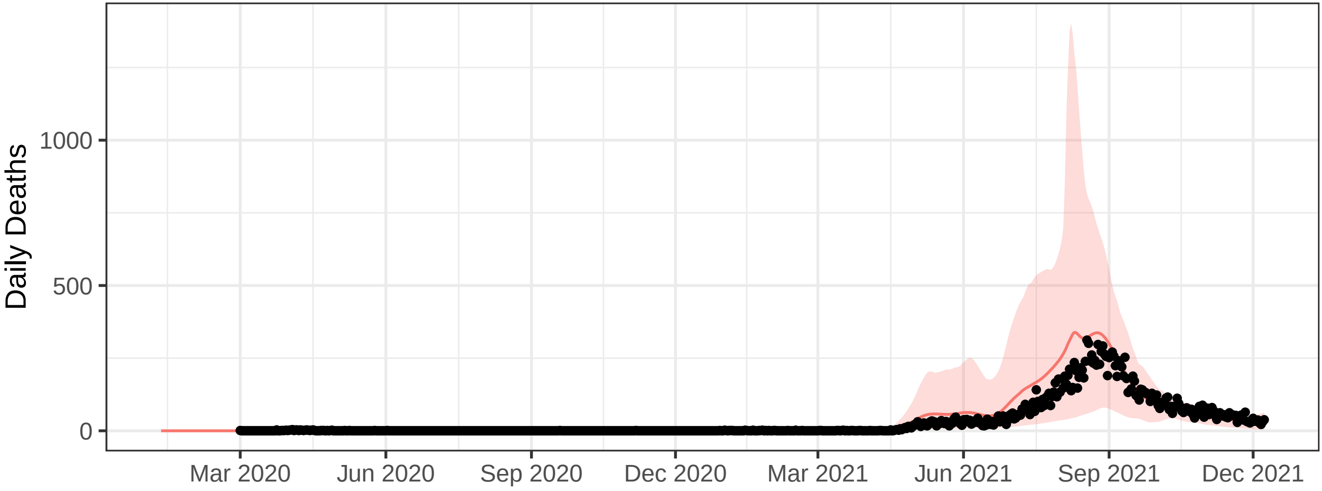

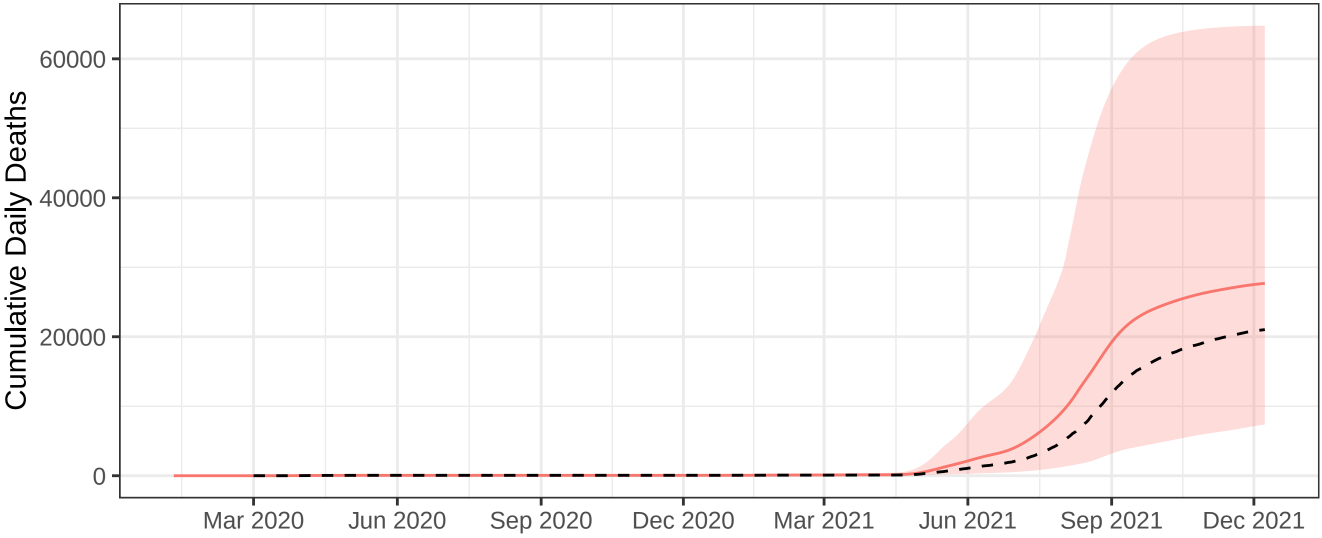

Daily deaths are included with reported COVID−19 deaths shown as points. The red line represents median model estimates of daily deaths, and the shaded region represents the 95% quantiles of the model estimated deaths. Plots of the cumulative estimated deaths are also shown, with the cumulative number of reported deaths represented by a dashed black line.

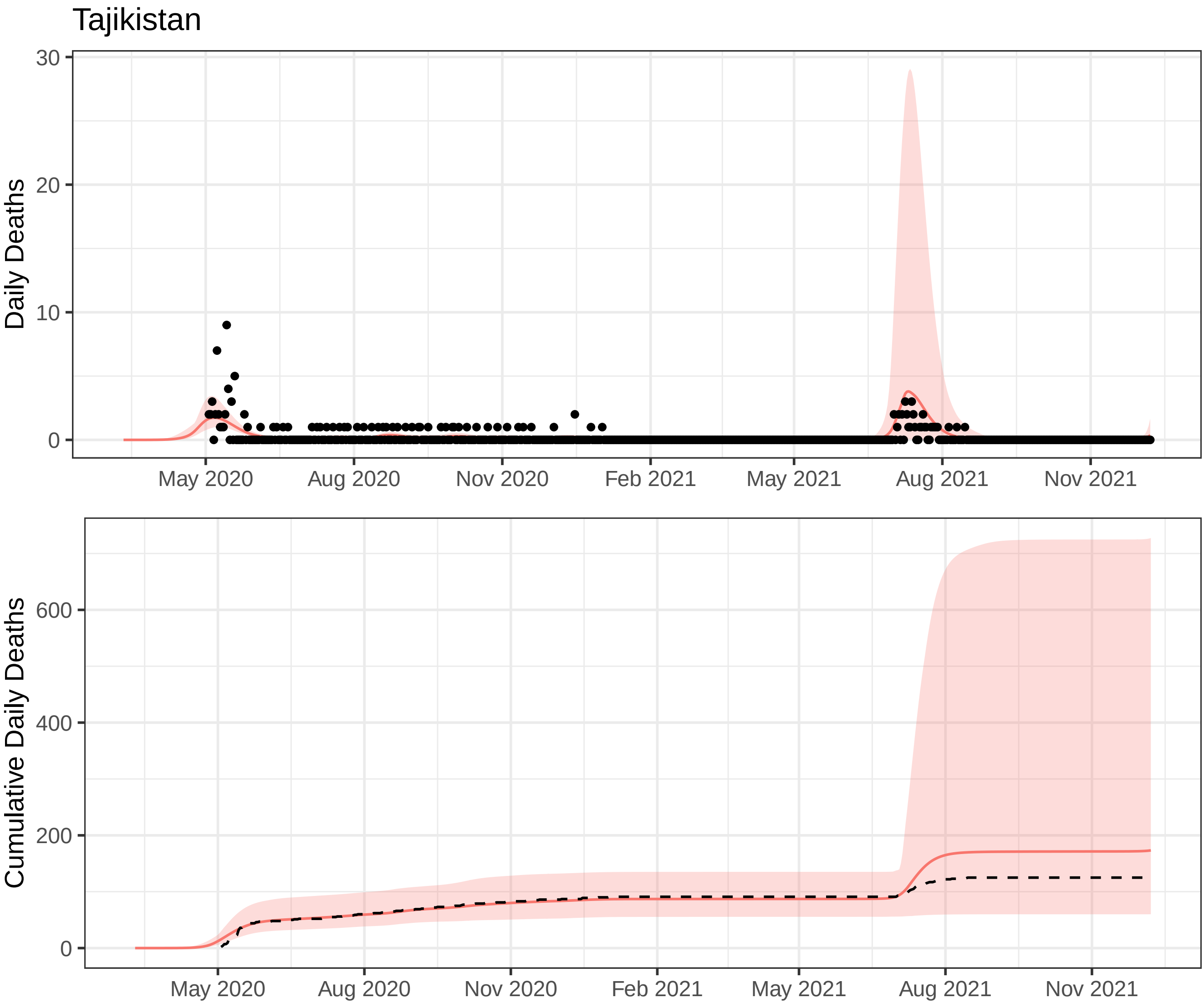

Daily deaths are included with reported COVID−19 deaths shown as points. The red line represents median model estimates of daily deaths, and the shaded region represents the 95% quantiles of the model estimated deaths. Plots of the cumulative estimated deaths are also shown, with the cumulative number of reported deaths represented by a dashed black line.

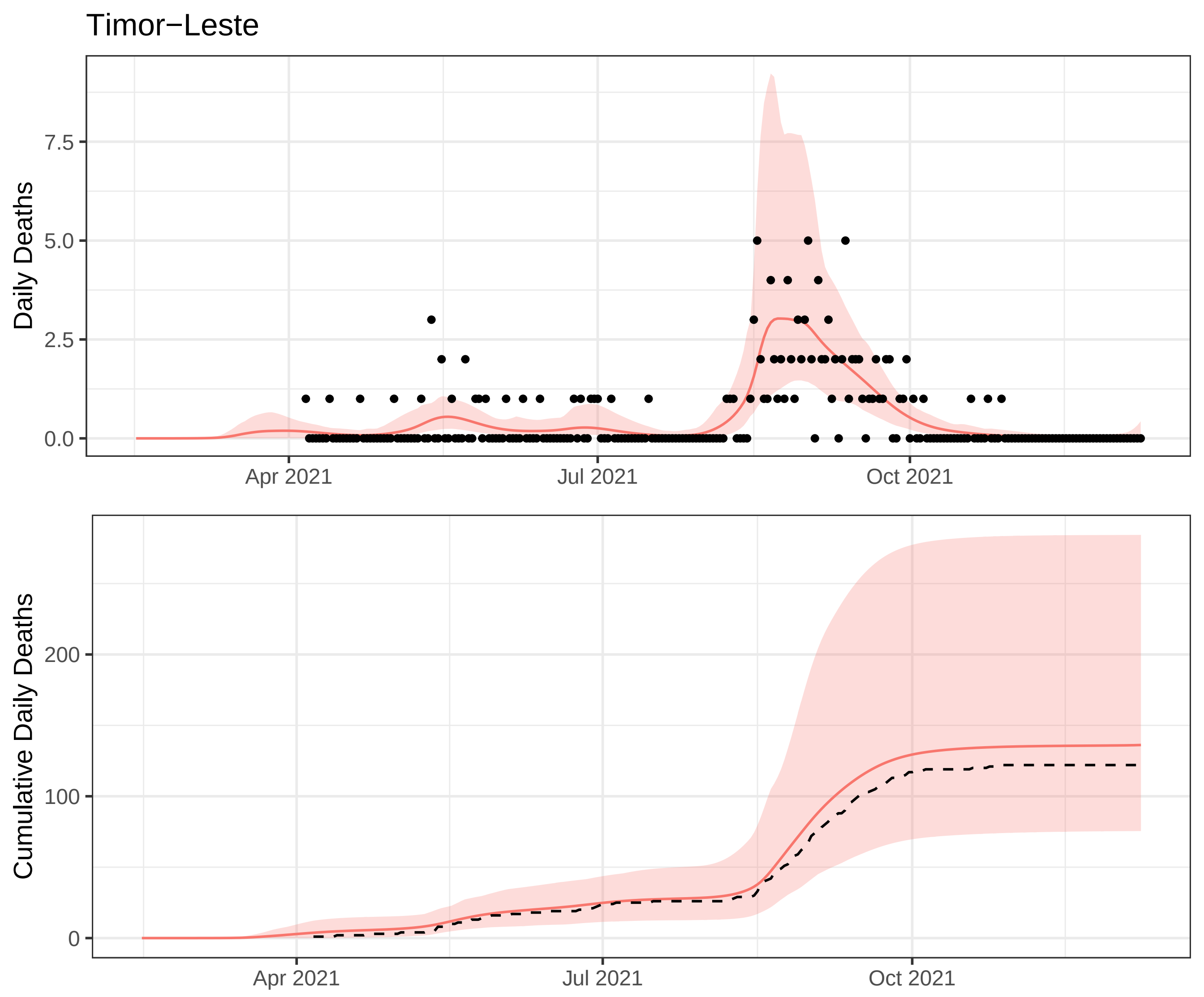

Daily deaths are included with reported COVID−19 deaths shown as points. The red line represents median model estimates of daily deaths, and the shaded region represents the 95% quantiles of the model estimated deaths. Plots of the cumulative estimated deaths are also shown, with the cumulative number of reported deaths represented by a dashed black line.

Trinidad and Tobago

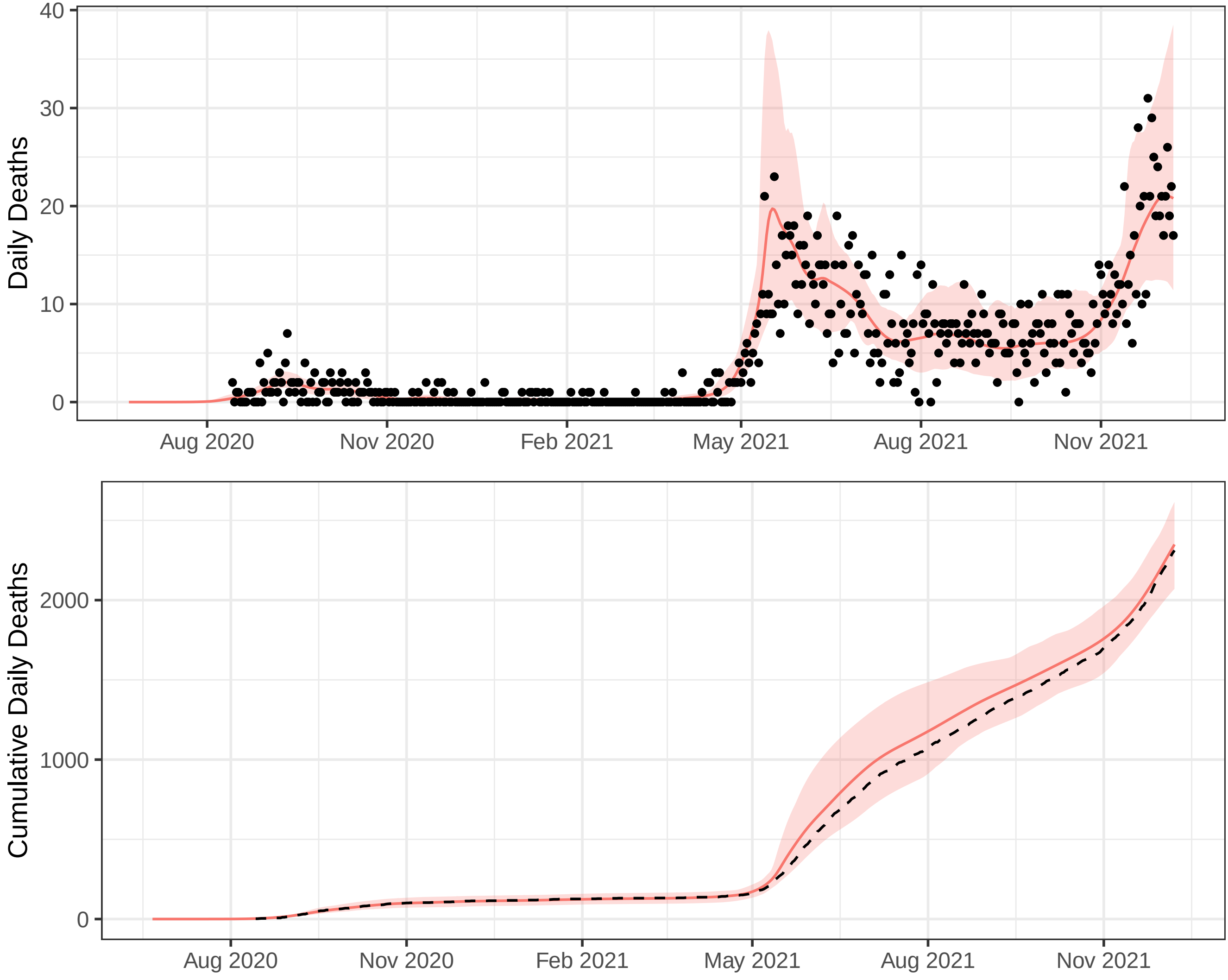

Daily deaths are included with reported COVID−19 deaths shown as points. The red line represents median model estimates of daily deaths, and the shaded region represents the 95% quantiles of the model estimated deaths. Plots of the cumulative estimated deaths are also shown, with the cumulative number of reported deaths represented by a dashed black line.

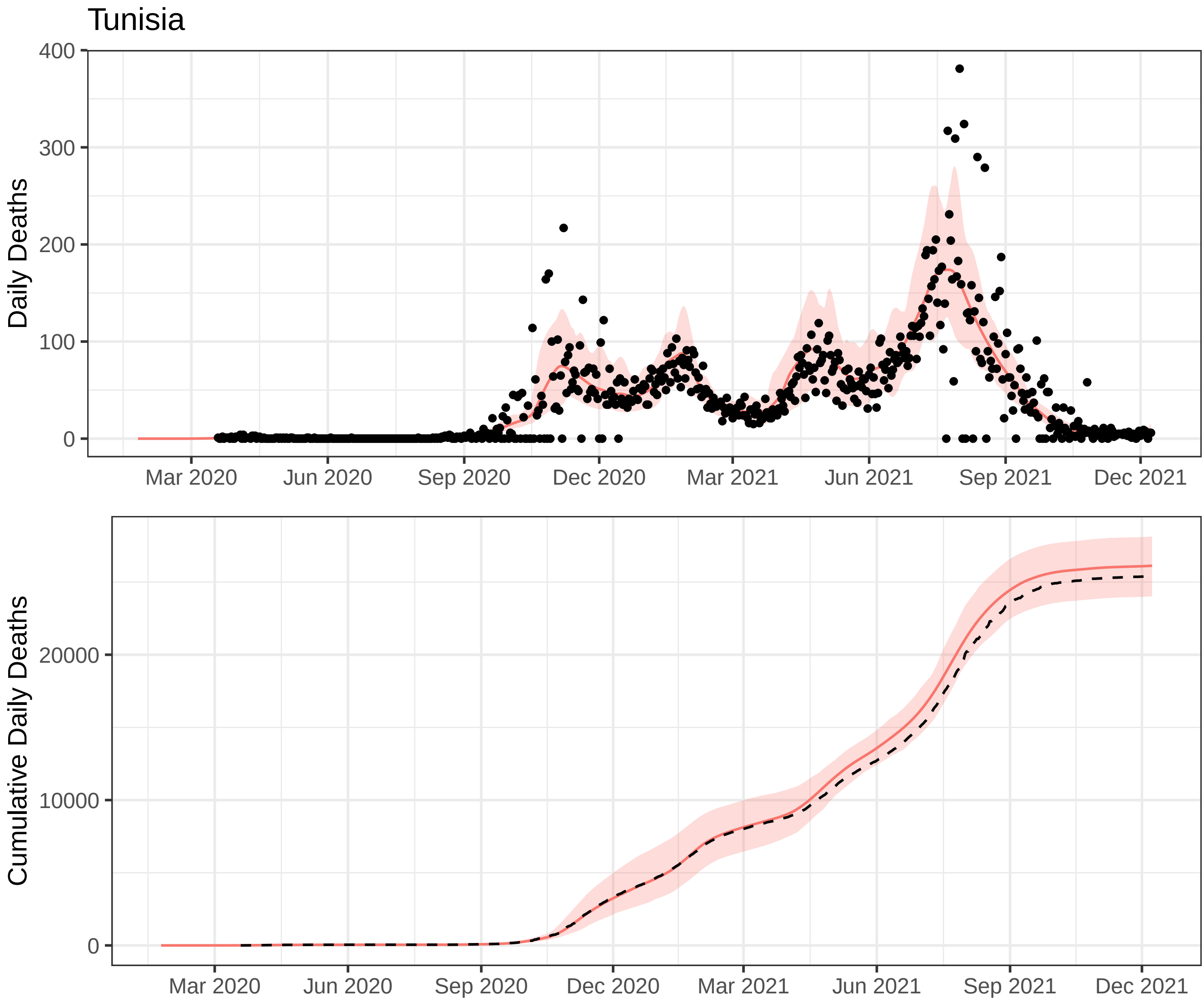

Daily deaths are included with reported COVID−19 deaths shown as points. The red line represents median model estimates of daily deaths, and the shaded region represents the 95% quantiles of the model estimated deaths. Plots of the cumulative estimated deaths are also shown, with the cumulative number of reported deaths represented by a dashed black line.

Turkey

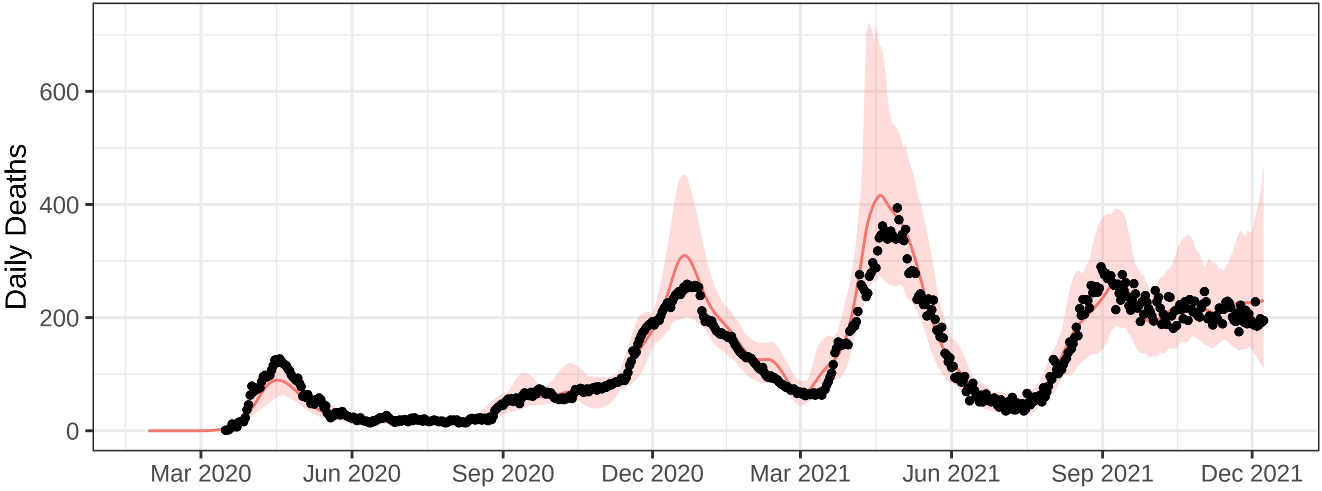

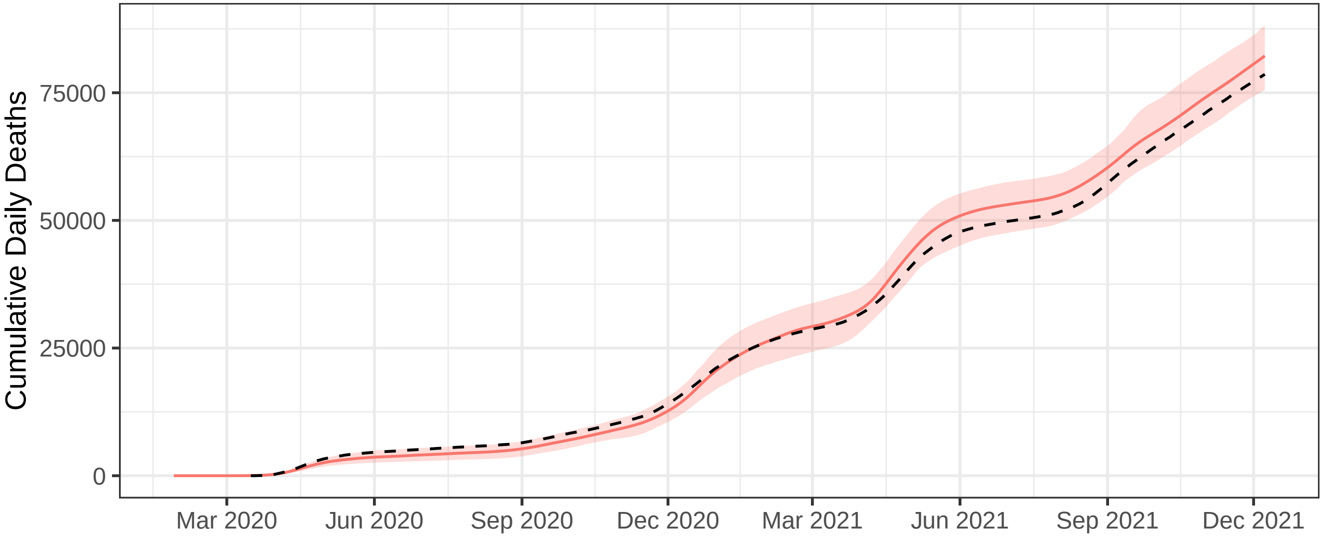

Daily deaths are included with reported COVID−19 deaths shown as points. The red line represents median model estimates of daily deaths, and the shaded region represents the 95% quantiles of the model estimated deaths. Plots of the cumulative estimated deaths are also shown, with the cumulative number of reported deaths represented by a dashed black line.

China, Taiwan Province of China

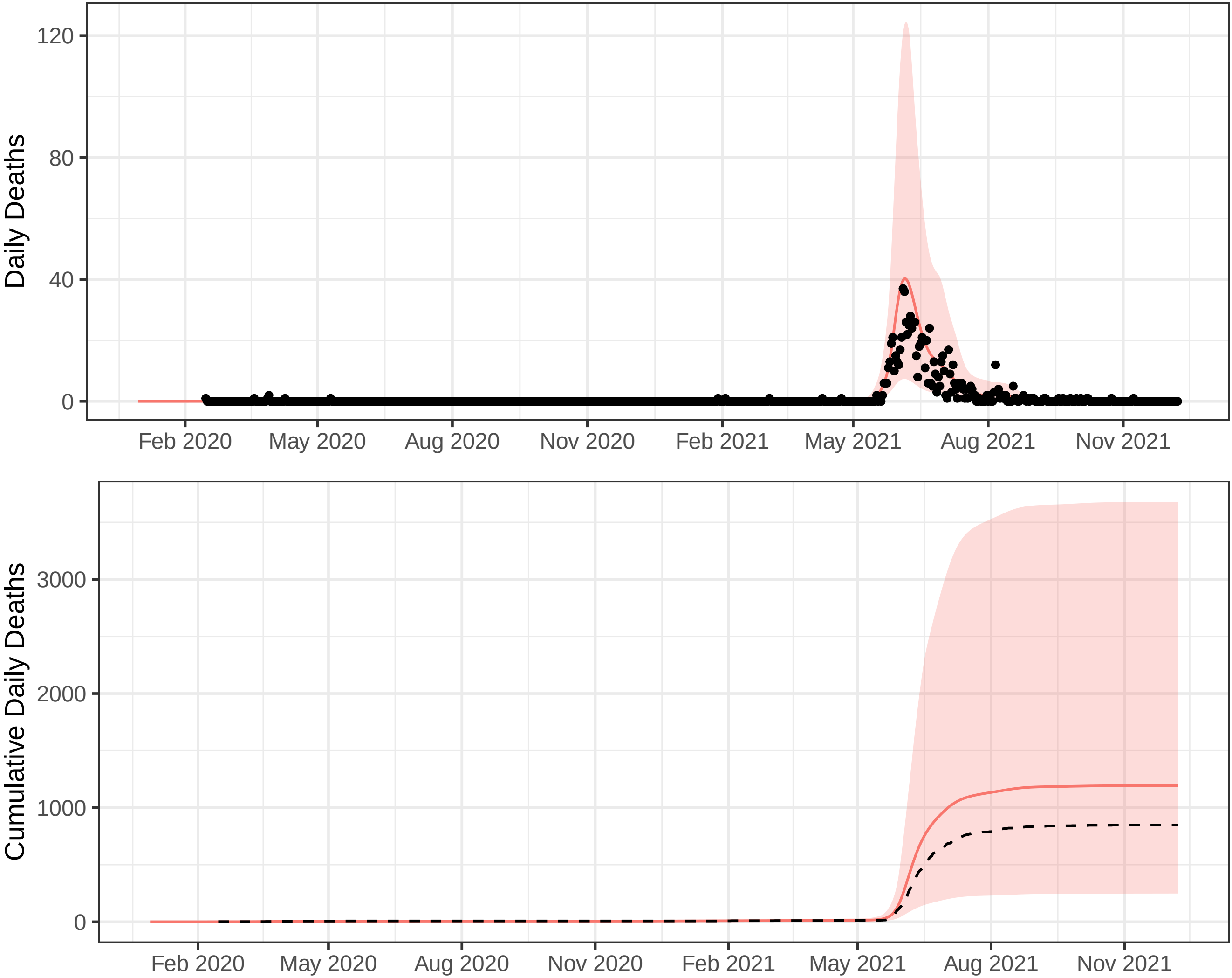

Daily deaths are included with reported COVID−19 deaths shown as points. The red line represents median model estimates of daily deaths, and the shaded region represents the 95% quantiles of the model estimated deaths. Plots of the cumulative estimated deaths are also shown, with the cumulative number of reported deaths represented by a dashed black line.

Tanzania

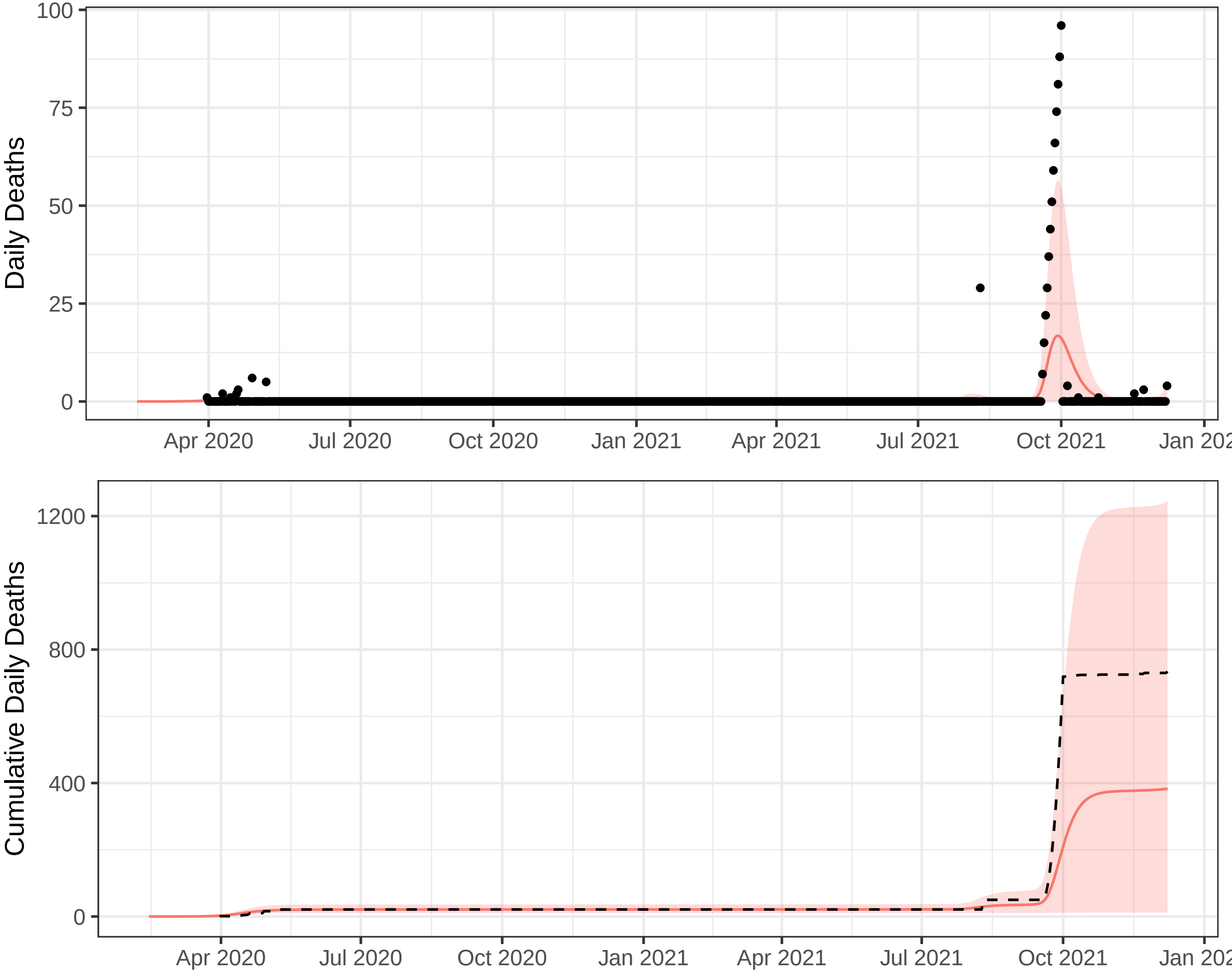

Daily deaths are included with reported COVID−19 deaths shown as points. The red line represents median model estimates of daily deaths, and the shaded region represents the 95% quantiles of the model estimated deaths. Plots of the cumulative estimated deaths are also shown, with the cumulative number of reported deaths represented by a dashed black line.

Uganda

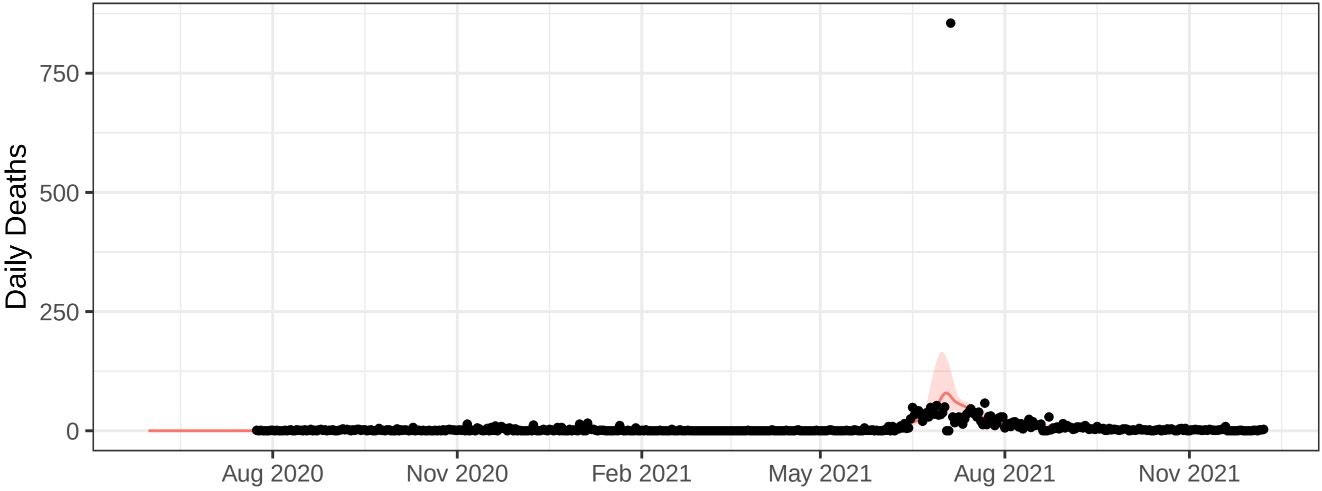

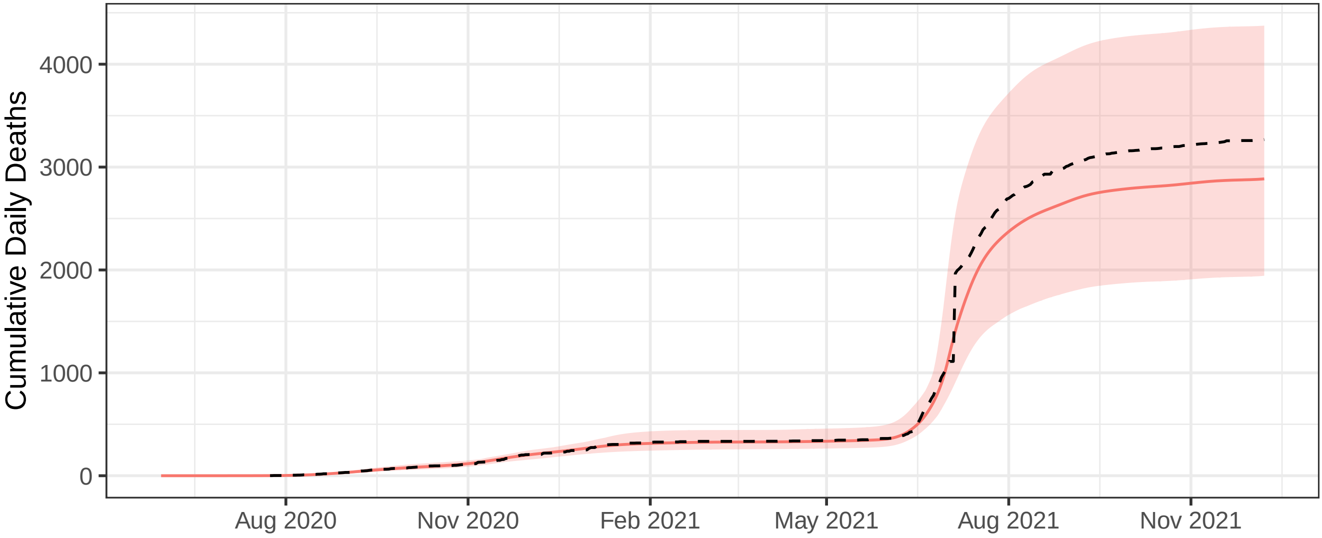

Daily deaths are included with reported COVID−19 deaths shown as points. The red line represents median model estimates of daily deaths, and the shaded region represents the 95% quantiles of the model estimated deaths. Plots of the cumulative estimated deaths are also shown, with the cumulative number of reported deaths represented by a dashed black line.

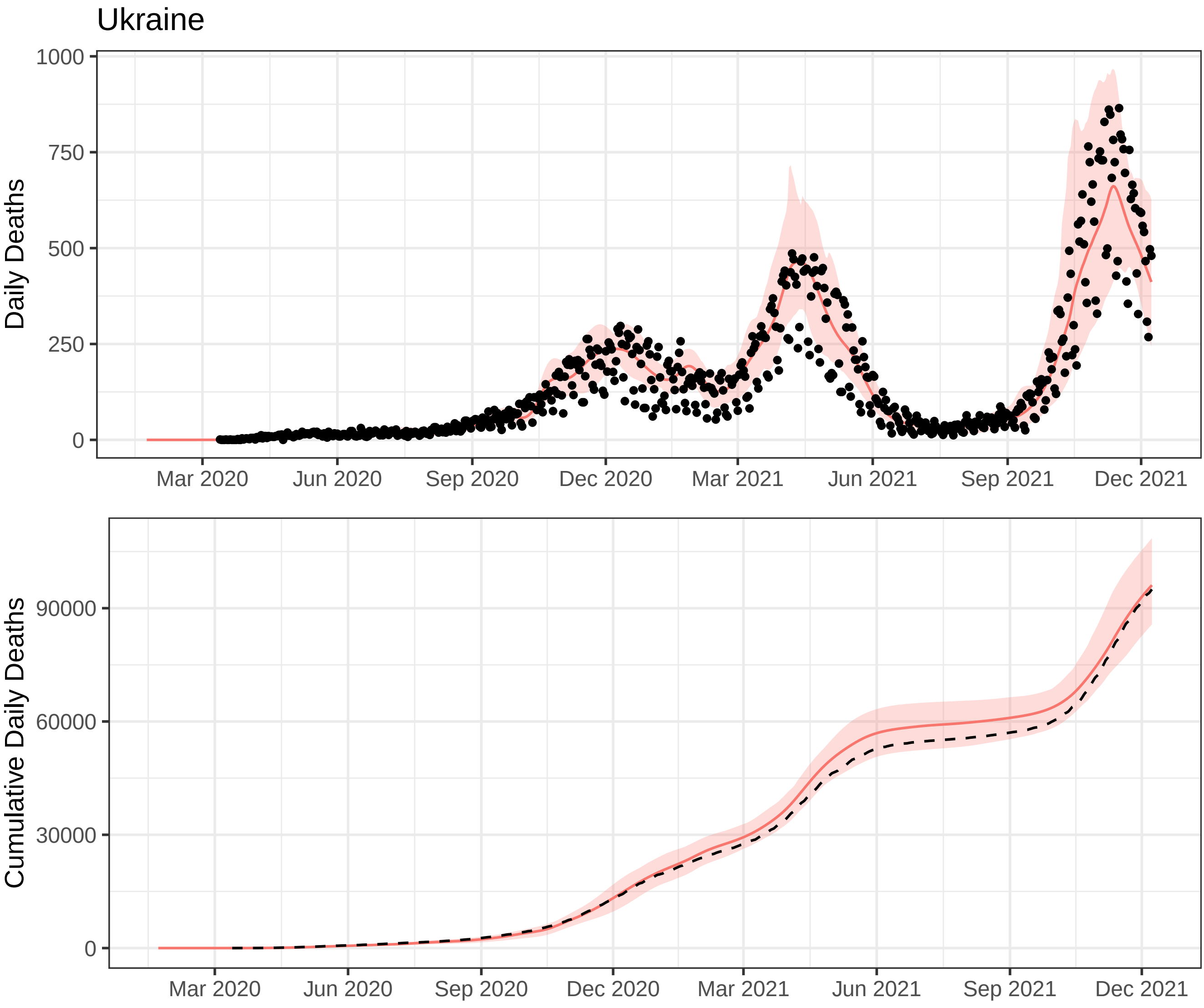

Daily deaths are included with reported COVID−19 deaths shown as points. The red line represents median model estimates of daily deaths, and the shaded region represents the 95% quantiles of the model estimated deaths. Plots of the cumulative estimated deaths are also shown, with the cumulative number of reported deaths represented by a dashed black line.

Uruguay

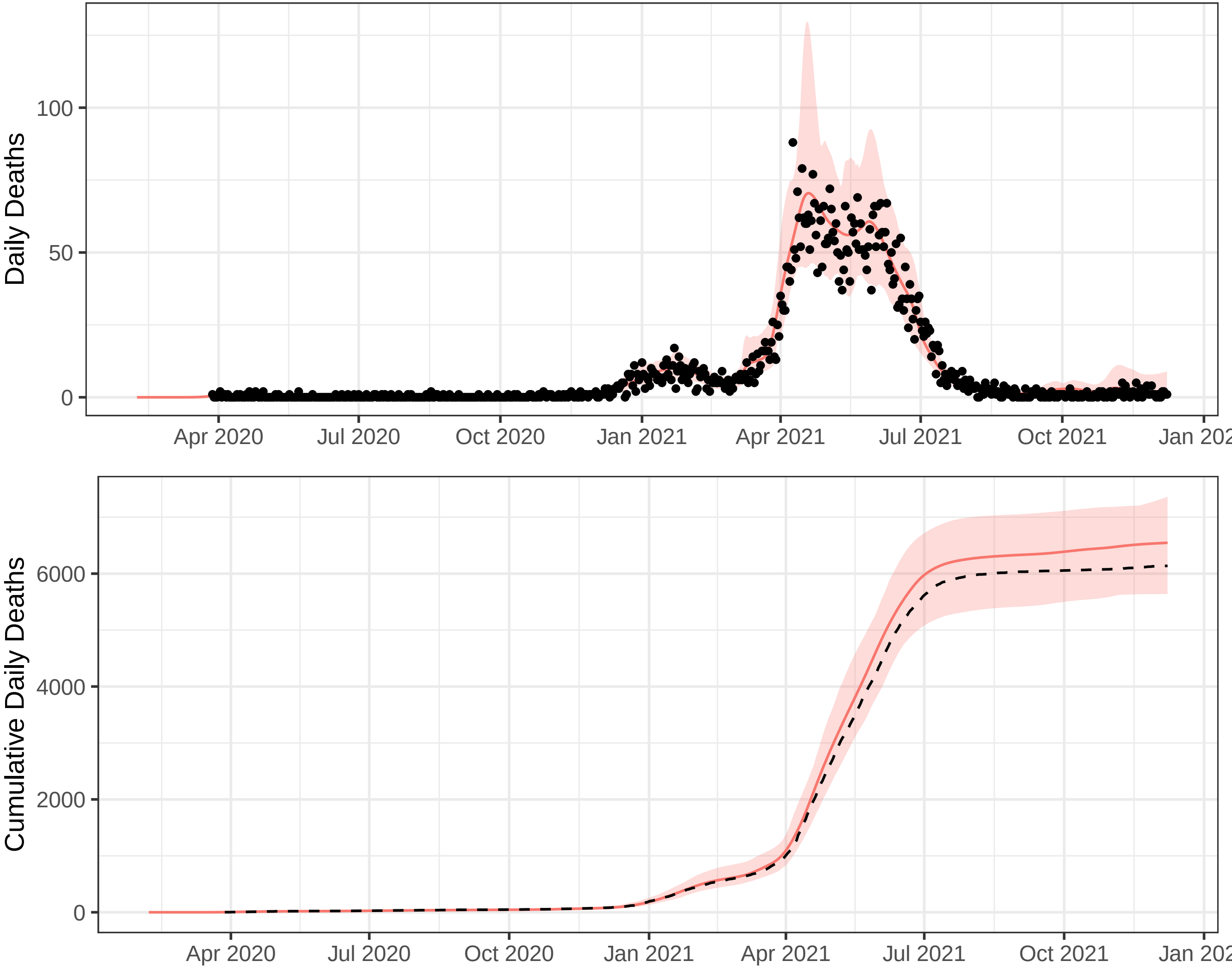

Daily deaths are included with reported COVID−19 deaths shown as points. The red line represents median model estimates of daily deaths, and the shaded region represents the 95% quantiles of the model estimated deaths. Plots of the cumulative estimated deaths are also shown, with the cumulative number of reported deaths represented by a dashed black line.

United States

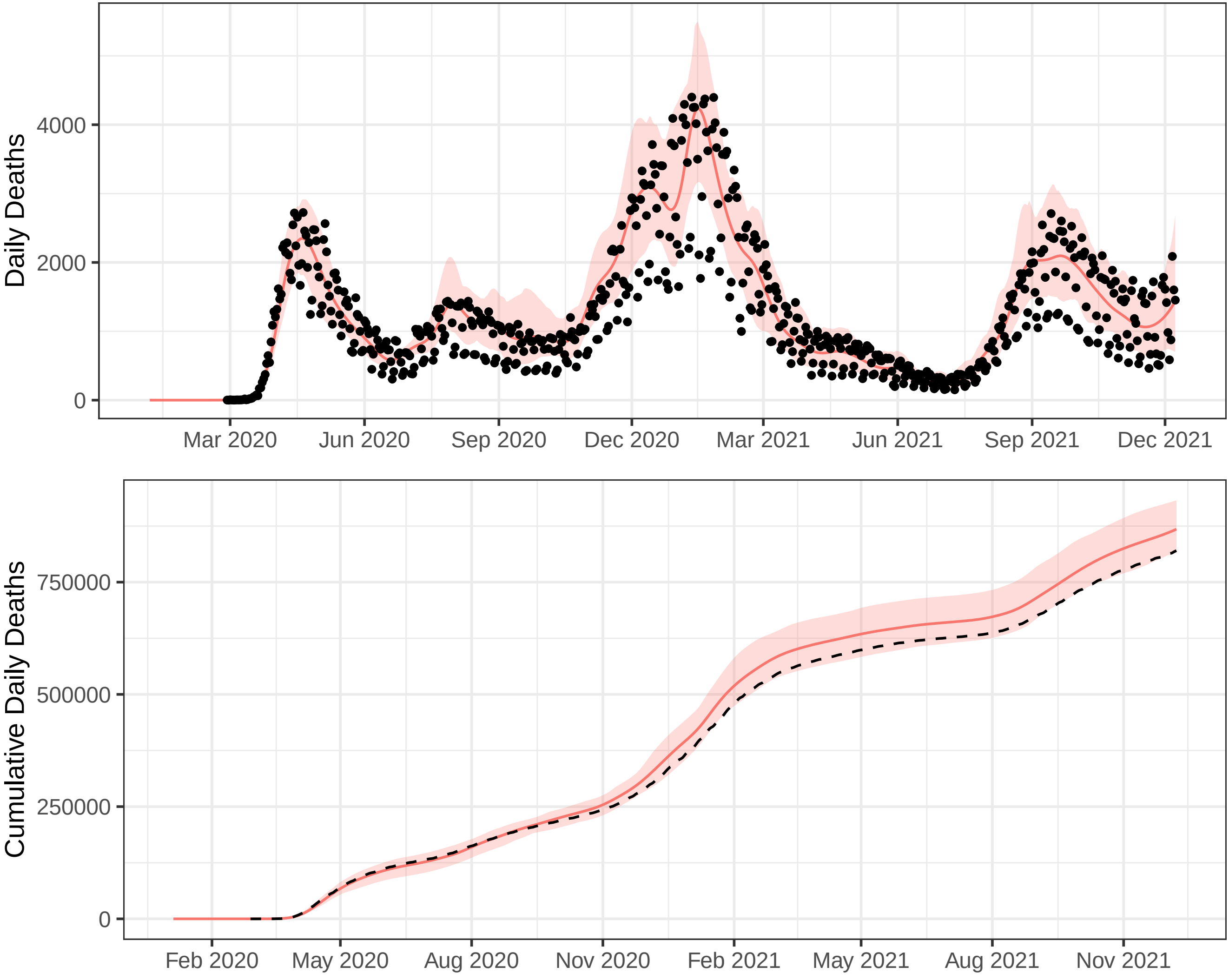

Daily deaths are included with reported COVID−19 deaths shown as points. The red line represents median model estimates of daily deaths, and the shaded region represents the 95% quantiles of the model estimated deaths. Plots of the cumulative estimated deaths are also shown, with the cumulative number of reported deaths represented by a dashed black line.

Uzbekistan

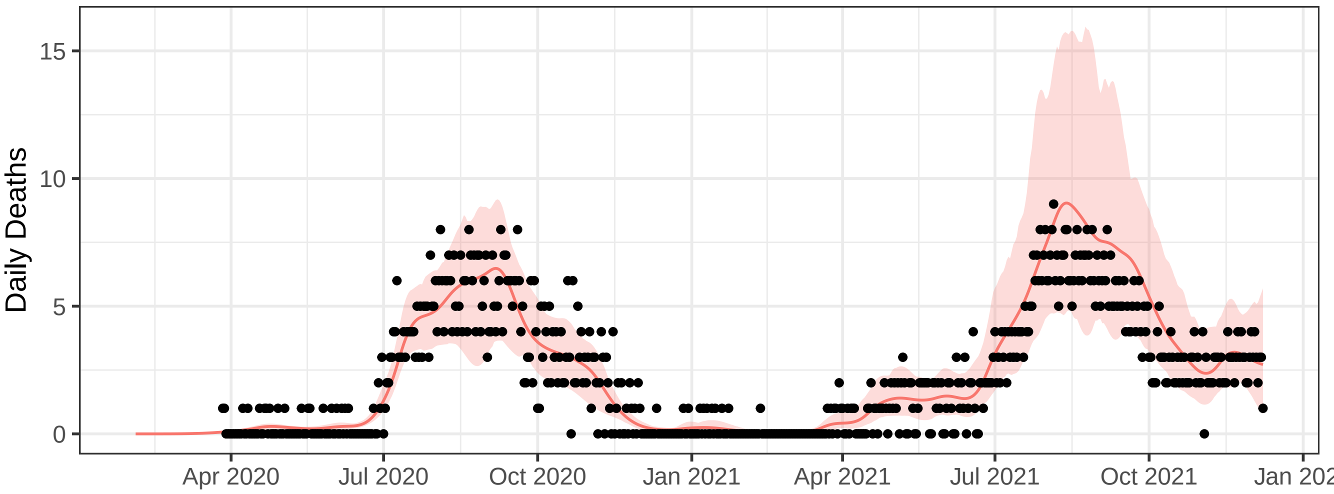

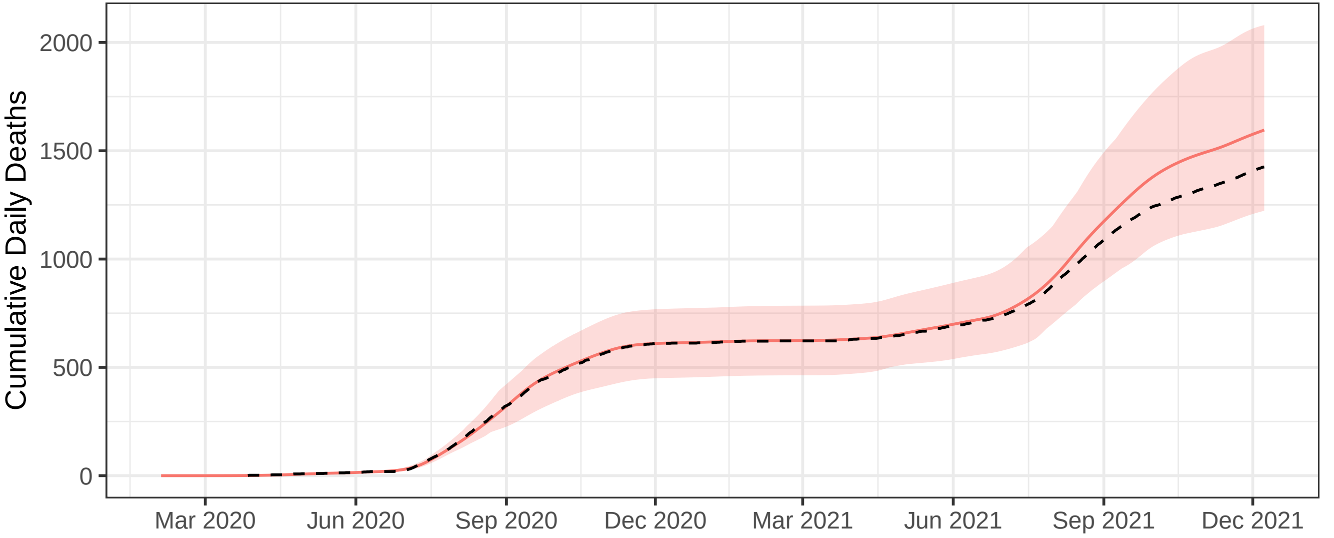

Daily deaths are included with reported COVID−19 deaths shown as points. The red line represents median model estimates of daily deaths, and the shaded region represents the 95% quantiles of the model estimated deaths. Plots of the cumulative estimated deaths are also shown, with the cumulative number of reported deaths represented by a dashed black line.

St. Vincent and the Grenadines

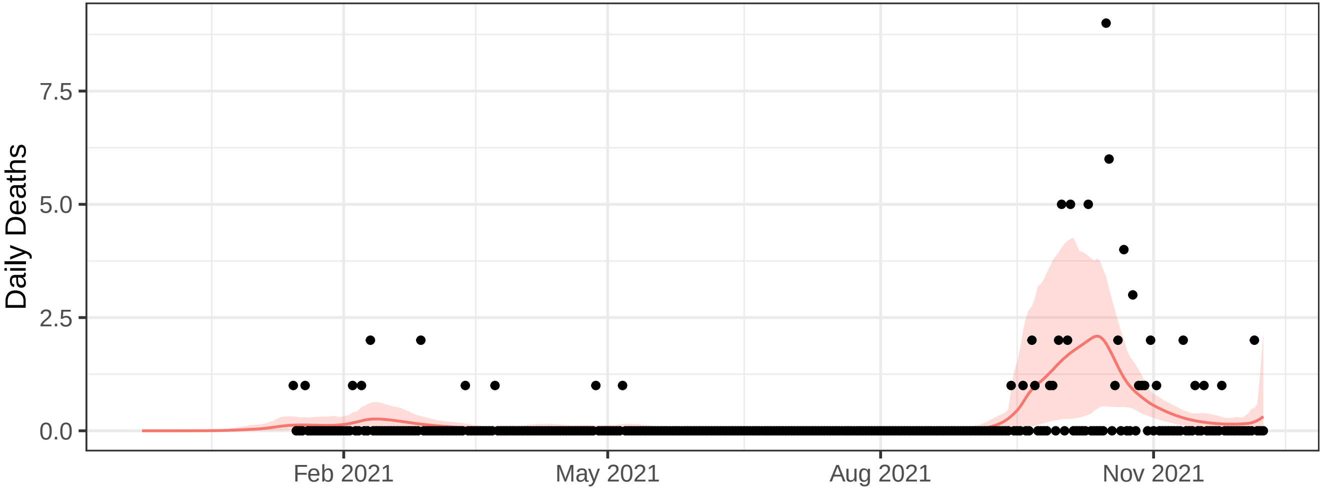

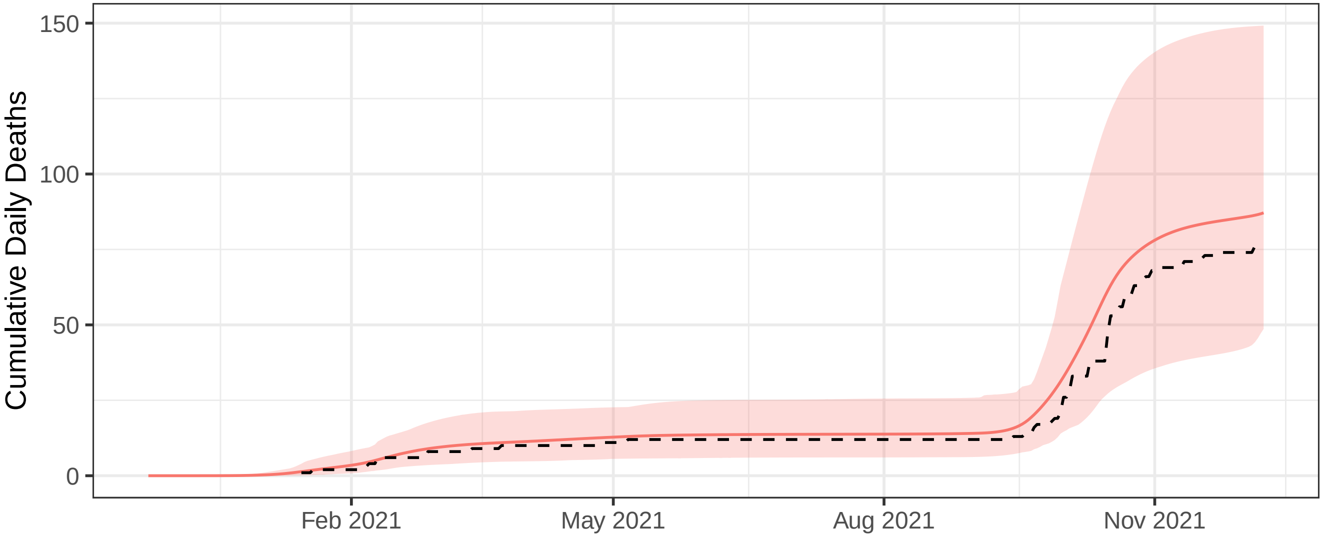

Daily deaths are included with reported COVID−19 deaths shown as points. The red line represents median model estimates of daily deaths, and the shaded region represents the 95% quantiles of the model estimated deaths. Plots of the cumulative estimated deaths are also shown, with the cumulative number of reported deaths represented by a dashed black line.

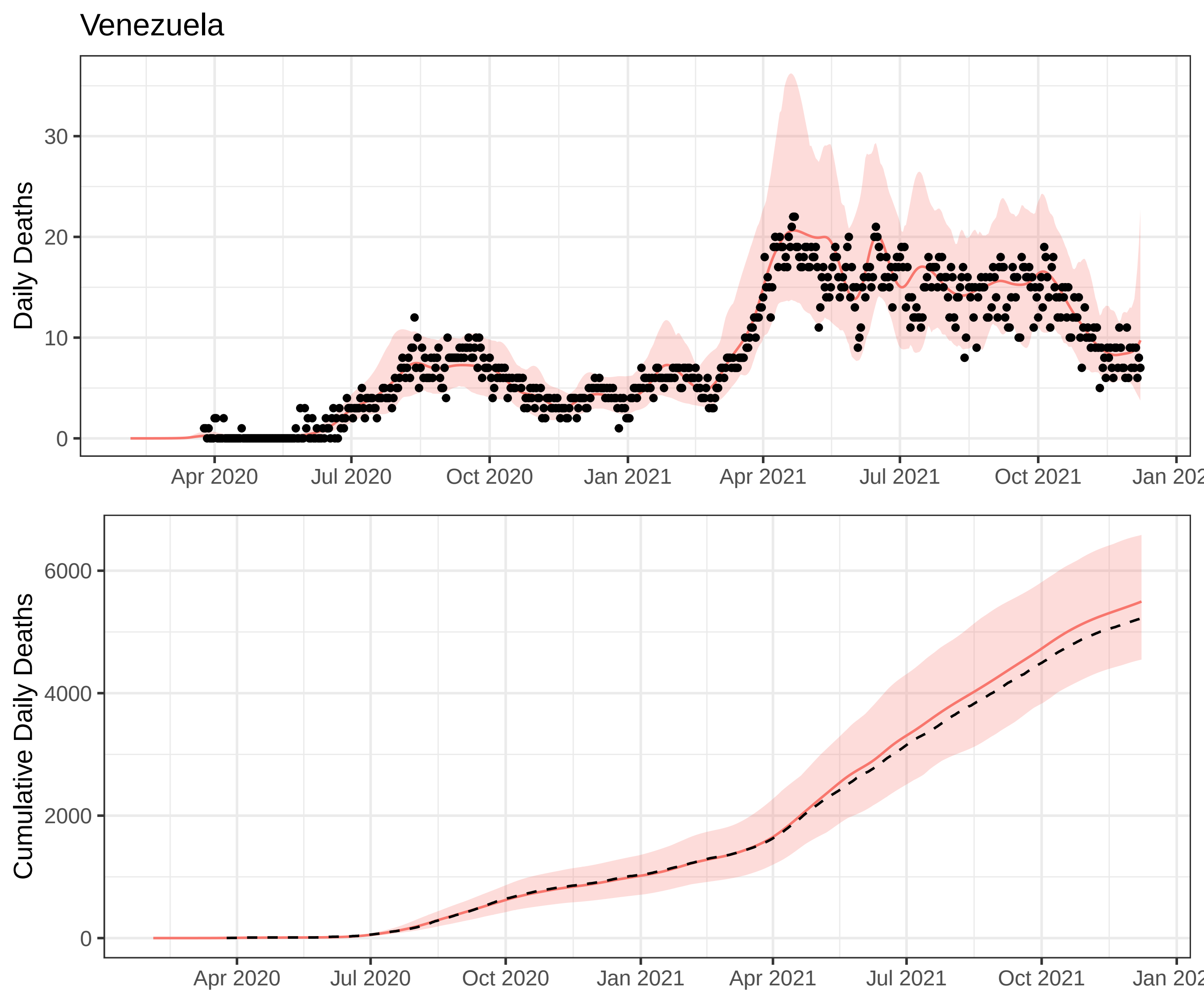

Daily deaths are included with reported COVID−19 deaths shown as points. The red line represents median model estimates of daily deaths, and the shaded region represents the 95% quantiles of the model estimated deaths. Plots of the cumulative estimated deaths are also shown, with the cumulative number of reported deaths represented by a dashed black line.

Vietnam

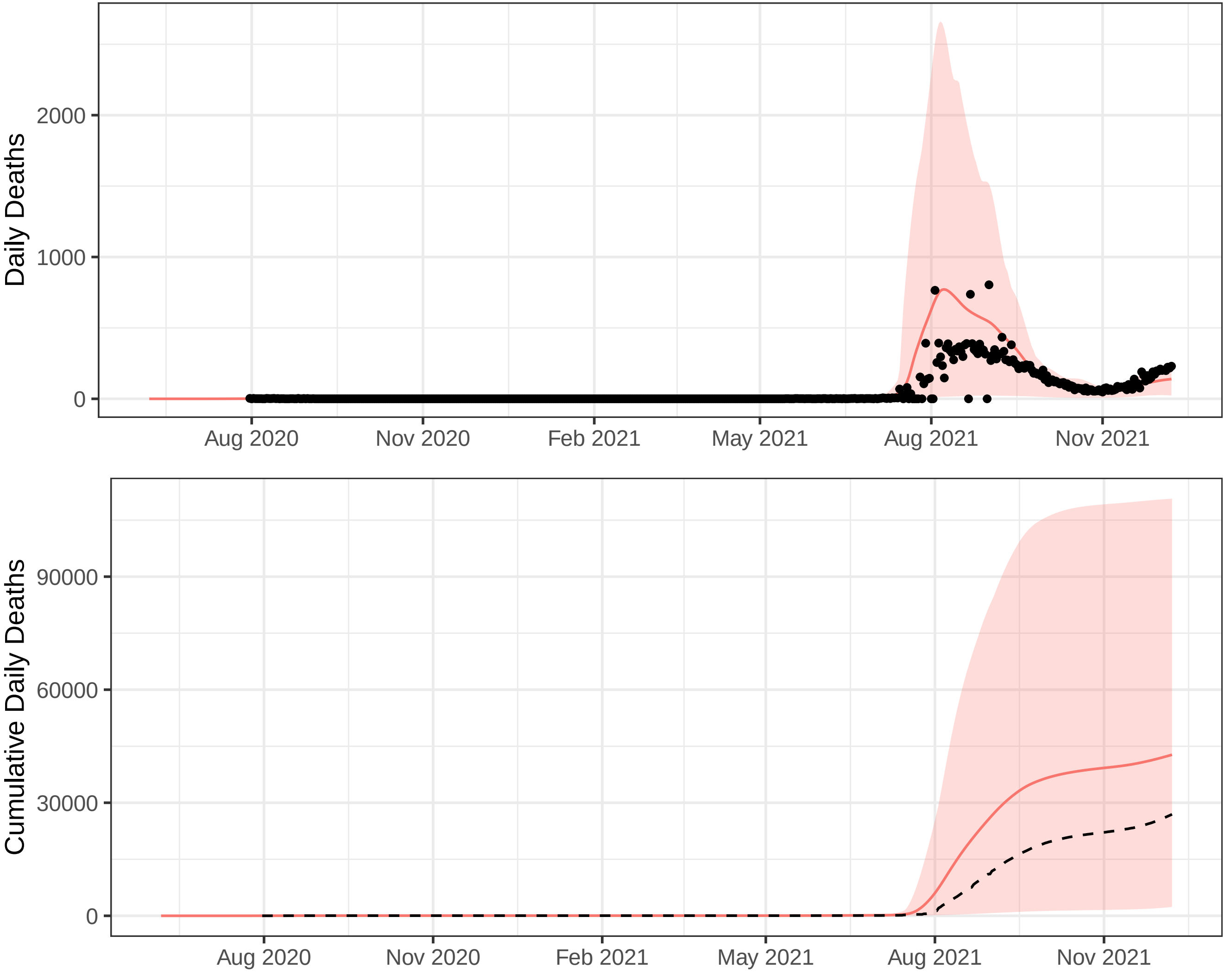

Daily deaths are included with reported COVID−19 deaths shown as points. The red line represents median model estimates of daily deaths, and the shaded region represents the 95% quantiles of the model estimated deaths. Plots of the cumulative estimated deaths are also shown, with the cumulative number of reported deaths represented by a dashed black line.

Vanuatu

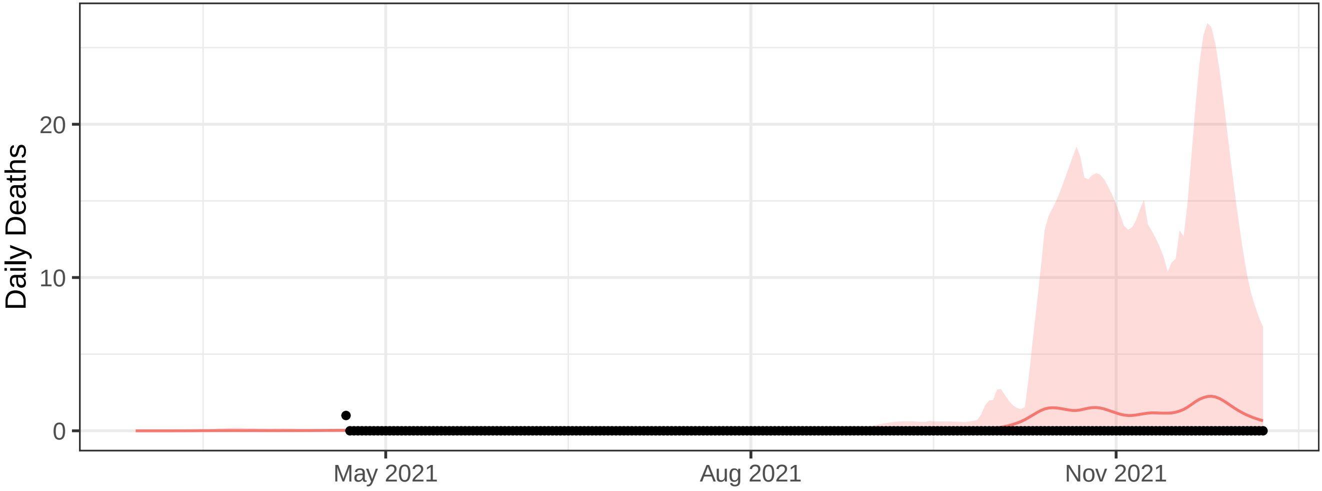

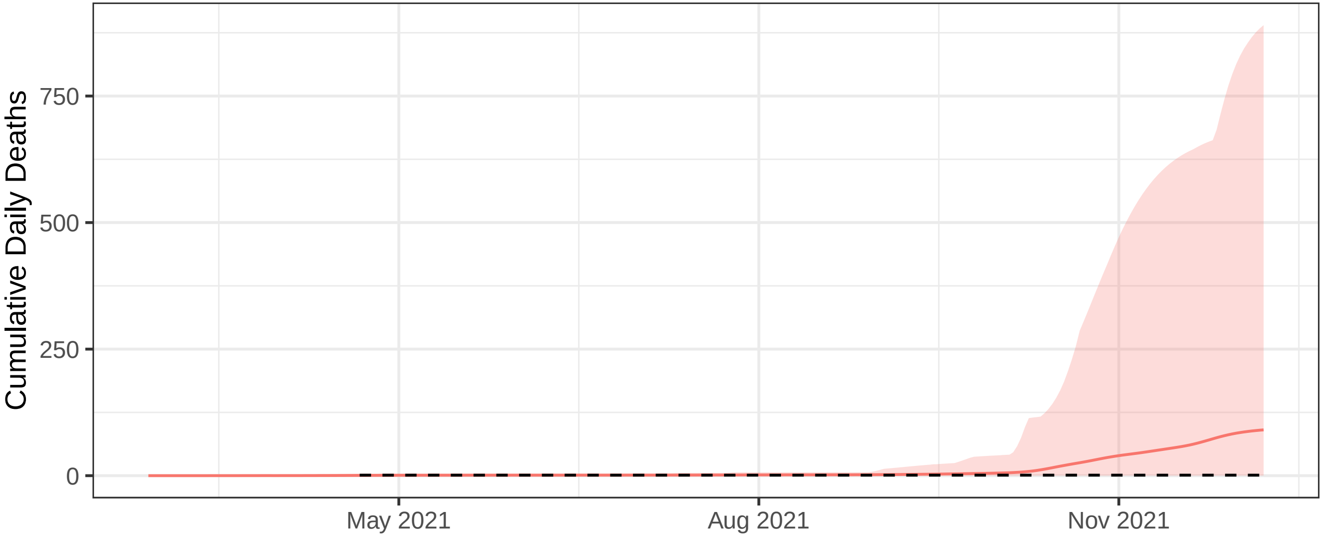

Daily deaths are included with reported COVID−19 deaths shown as points. The red line represents median model estimates of daily deaths, and the shaded region represents the 95% quantiles of the model estimated deaths. Plots of the cumulative estimated deaths are also shown, with the cumulative number of reported deaths represented by a dashed black line.

Yemen

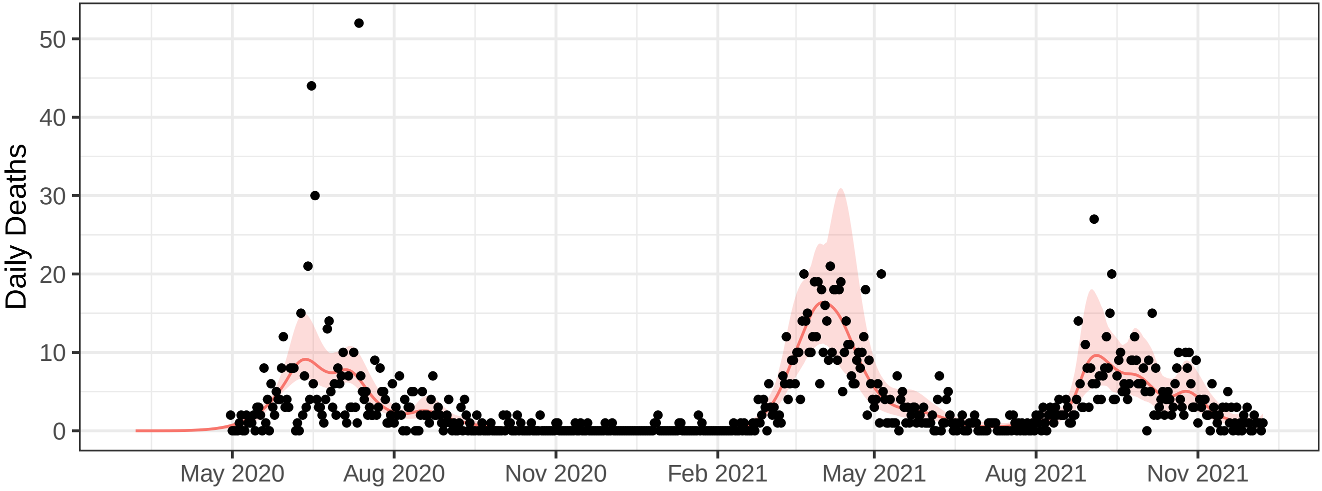

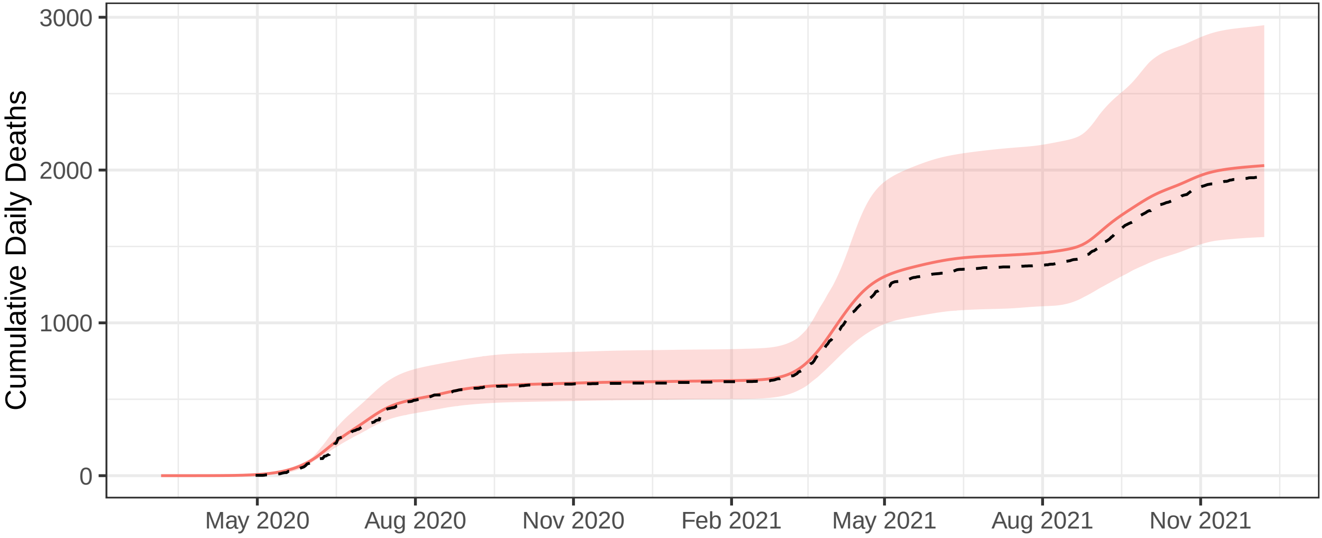

Daily deaths are included with reported COVID−19 deaths shown as points. The red line represents median model estimates of daily deaths, and the shaded region represents the 95% quantiles of the model estimated deaths. Plots of the cumulative estimated deaths are also shown, with the cumulative number of reported deaths represented by a dashed black line.

South Africa

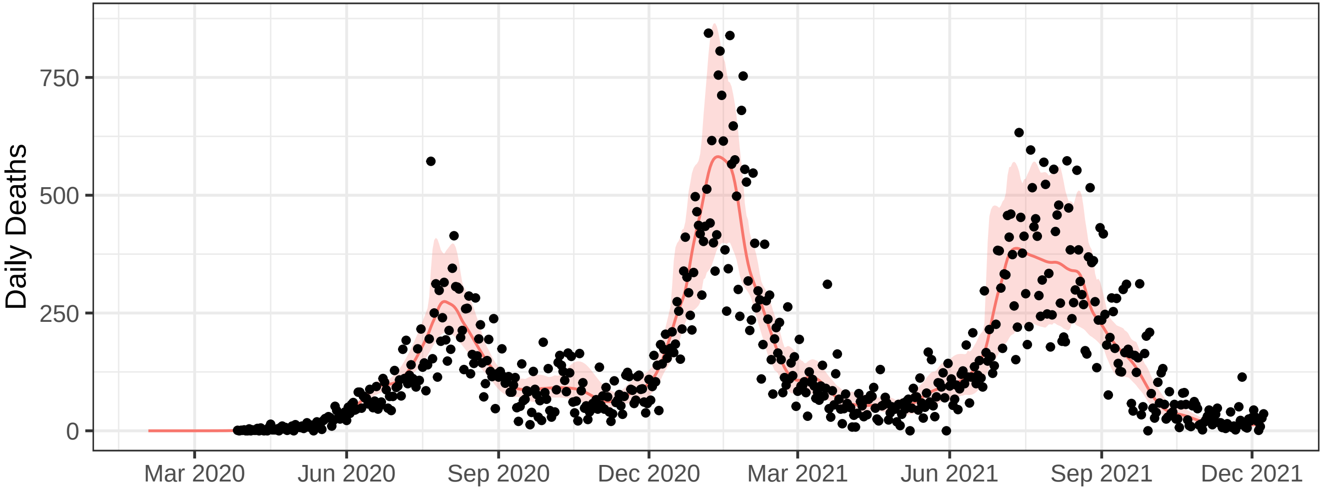

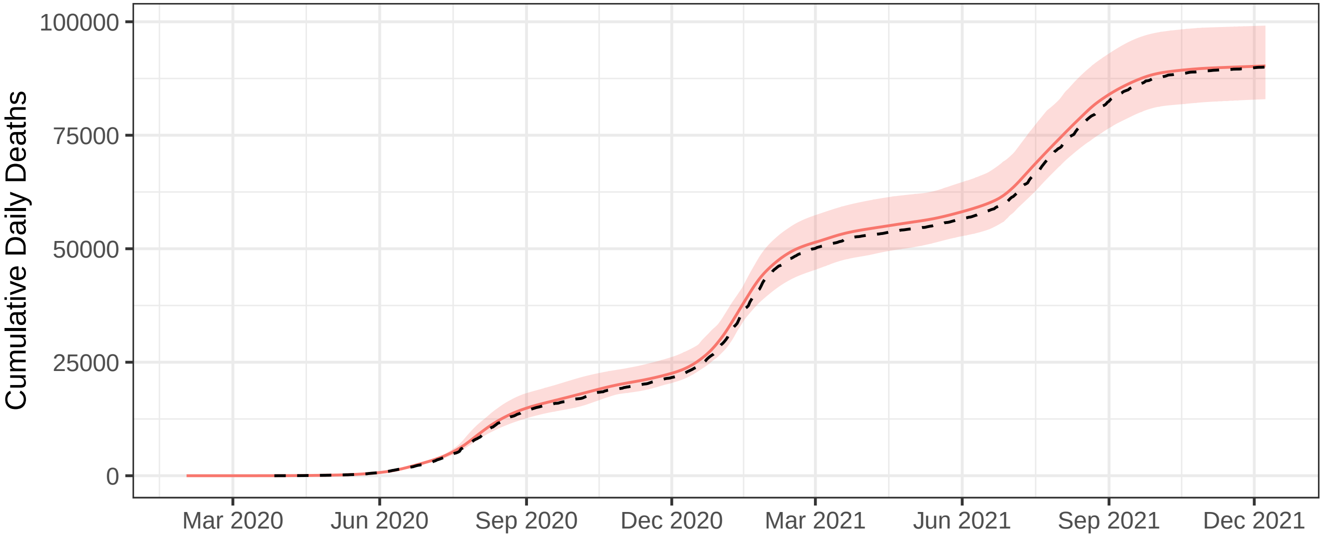

Daily deaths are included with reported COVID−19 deaths shown as points. The red line represents median model estimates of daily deaths, and the shaded region represents the 95% quantiles of the model estimated deaths. Plots of the cumulative estimated deaths are also shown, with the cumulative number of reported deaths represented by a dashed black line.

Zambia

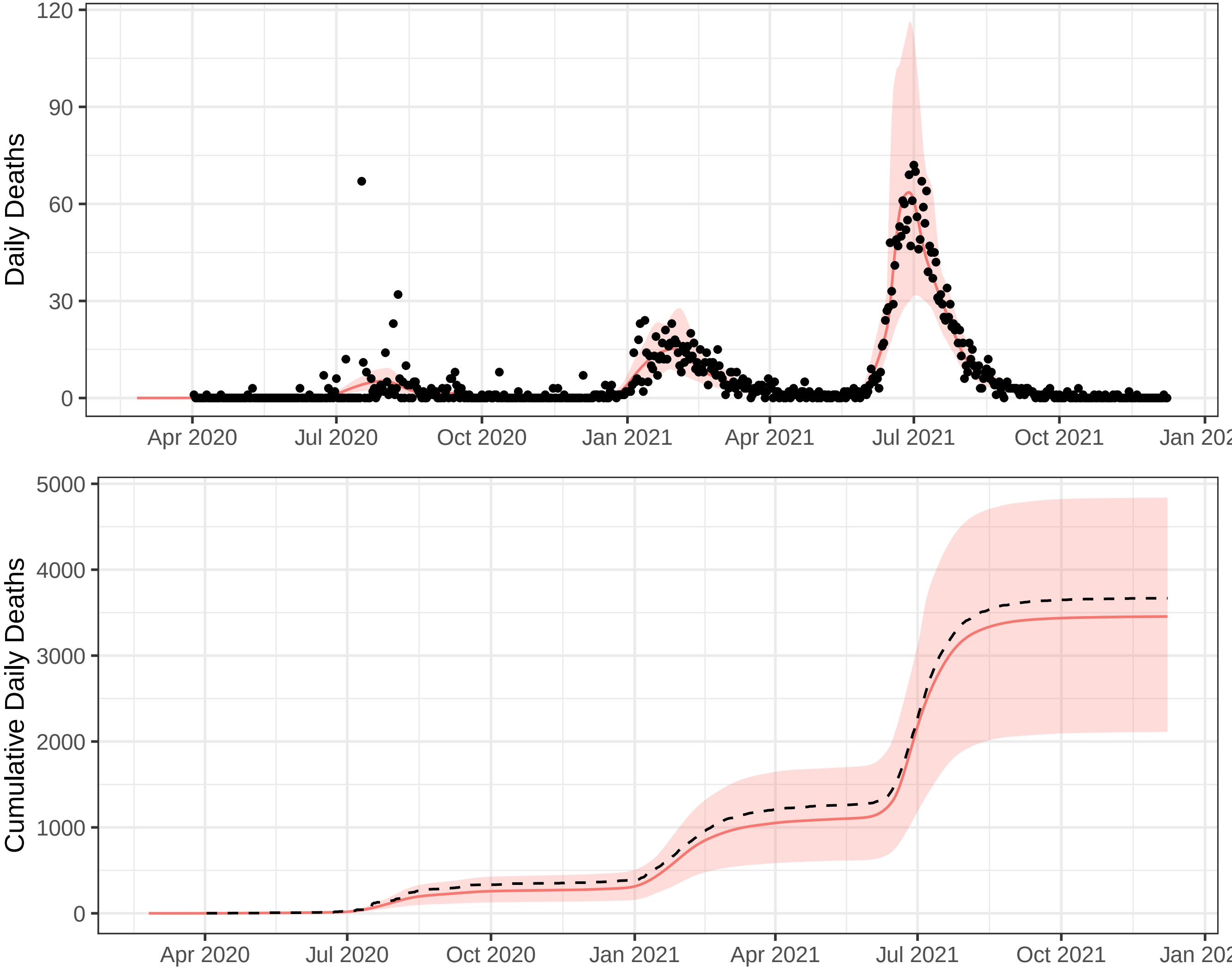

Daily deaths are included with reported COVID−19 deaths shown as points. The red line represents median model estimates of daily deaths, and the shaded region represents the 95% quantiles of the model estimated deaths. Plots of the cumulative estimated deaths are also shown, with the cumulative number of reported deaths represented by a dashed black line.

Zimbabwe

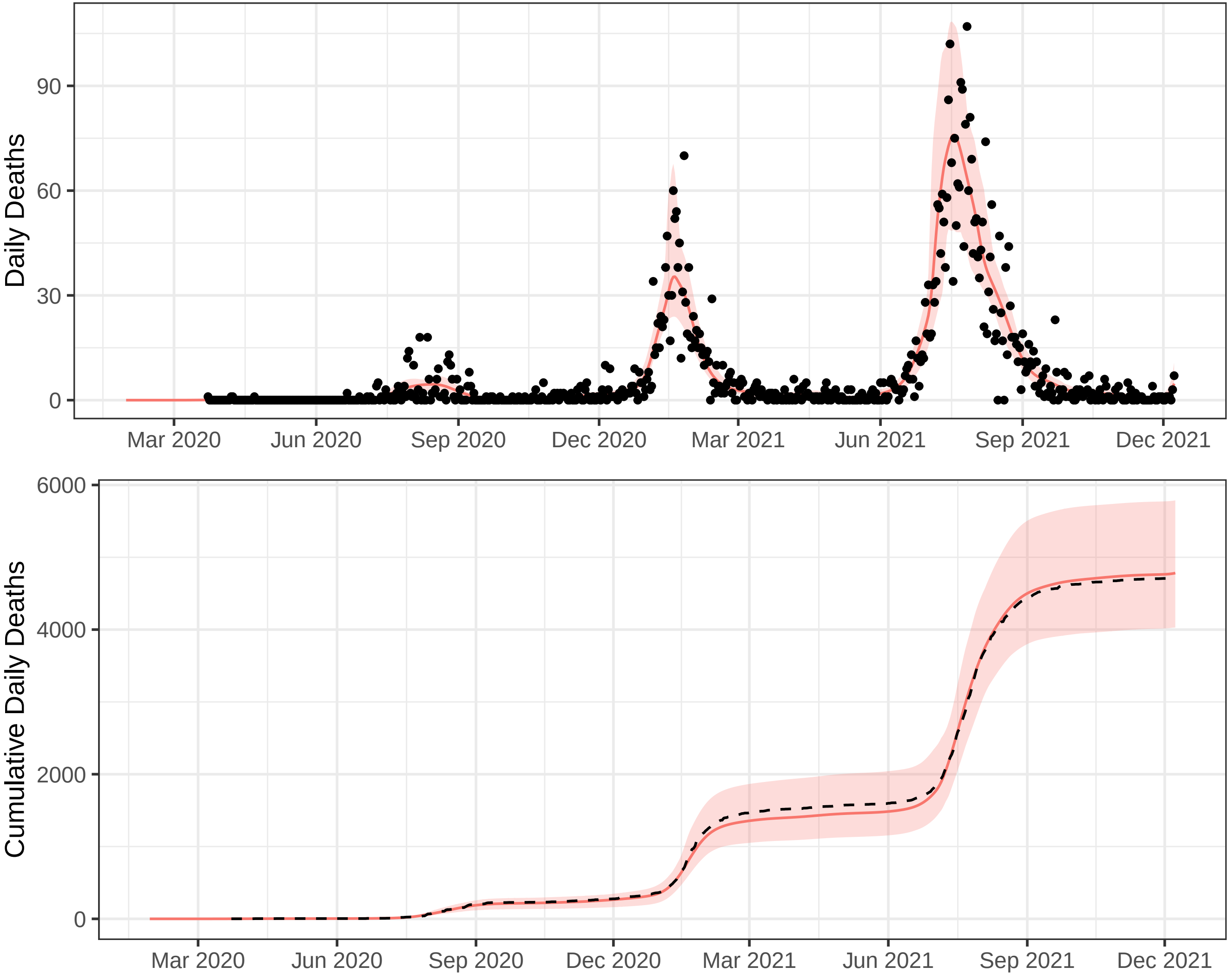

Daily deaths are included with reported COVID−19 deaths shown as points. The red line represents median model estimates of daily deaths, and the shaded region represents the 95% quantiles of the model estimated deaths. Plots of the cumulative estimated deaths are also shown, with the cumulative number of reported deaths represented by a dashed black line.
